# Supplementary material for: Neutron-encoded diubiquitins to profile linkage selectivity of deubiquitinating enzymes
Source: Nat Commun. 2023 Mar 25;14:1661. doi: 10.1038/s41467-023-37363-6 (PMC10039891; doi:10.1038/s41467-023-37363-6)
Supplement: Supplementary file 1 — Supplementary Information [file 41467_2023_37363_MOESM1_ESM.pdf]

Supplementary Information

Neutron-encoded diubiquitins to profile linkage selectivity of deubiquitinating enzymes

Bianca D. M. van Tol[1], Bjorn R. van Doodewaerd[1], Guinevere S. M. Lageveen-Kammeijer[2], Bas C. Jansen[2], Cami M. P. Talavera Ormeño[1], Paul J. M. Hekking[1], Aysegul Sapmaz[1], Robbert Q. Kim[1], Angeliki Moutsiopoulou[1], David Komander[3], Manfred Wuhrer[2], Gerbrand J. van der Heden van Noort[1], Huib Ovaa[1,†], Paul P. Geurink[1,*]

[1] Oncode Institute and Department of Cell and Chemical Biology, Chemical Biology and Drug Discovery, Leiden University Medical Center, 2333 ZC Leiden, The Netherlands

[2] Center for Proteomics and Metabolomics, Leiden University Medical Center, 2333 ZA Leiden, The Netherlands

[3] Ubiquitin Signalling Division, Walter and Eliza Hall Institute of Medical Research, 1G Royal Parade, Parkville 3052 Melbourne, Victoria, Australia

* Corresponding author. E-mail: p.p.geurink@lumc.nl

Table of contents

Supplementary Figures and Tables	Page
Supplementary Figure 1. Synthesis of all eight neutron-encoded diUbs	SI-4
Supplementary Figure 2. Full gel image of all eight neutron-encoded diUbs	SI-5
Supplementary Figure 3. Full MS spectrum of the mix of all eight neutron-encoded diUbs	SI-6
Supplementary Figure 4. USP21 mediated hydrolysis of all eight neutron-encoded diUbs	SI-7
Supplementary Figure 5. DUB mediated hydrolysis of synthetic and enzymatic prepared diUbs	SI-8
Supplementary Figure 6. Full gel images of Supplementary Figure 5	SI-9
Supplementary Figure 7. Assay window and linearity determination	SI-10
Supplementary Figure 8. Comparison between LaCyTools analysis versus manual data analysis	SI-11
Supplementary Figure 9. Fmoc-protection of neutron-encoded Val, Leu and Ile	SI-13
Supplementary Figure 10. Synthesis of internal standard non-hydrolyzable clicked Lys48 diUb	SI-14
Supplementary Figure 11. Synthesis of methyl-3-(glycylthio)-propionate	SI-15
Supplementary Table 1. Amino acid sequence of designed proximal ubiquitins	SI-16
Supplementary Table 2. Purified recombinant DUBs used in this work	SI-17
Supplementary Table 3. LaCyTools settings	SI-19
Supplementary Table 4. Alignment file/table	SI-20
Supplementary Table 5. Analytes and calibrants – LaCyTools	SI-21
Supplementary Table 6. AMSH expression: PCR reaction	SI-22
Supplementary Table 7. AMSH expression: PCR cycle condition	SI-23
Supplementary Table 8. Sequence of the cloning primers for AMSH	SI-24

Supplementary Methods

Chemical Synthesis

General	SI-25
Fmoc protection of neuron-encoded amino acids	SI-27
Methyl-3-(glycylthio)-propionate hydrochloride synthesis	SI-28
^{1}H NMR/ ^{13}C NMR	SI-29
LC-MS analysis	SI-33

Protein Synthesis

General	SI-37
Solid Phase Peptide Synthesis	SI-38
General procedures, analysis and purification	SI-40
Synthesis of neutron-encoded monoubiquitins	SI-44
Synthesis of monoubiquitin thioester	SI-46
Synthesis of neutron-encoded diubiquitins	SI-47
Synthesis of internal standards	SI-50

Supplementary References SI-53

Supplementary Figures and Tables

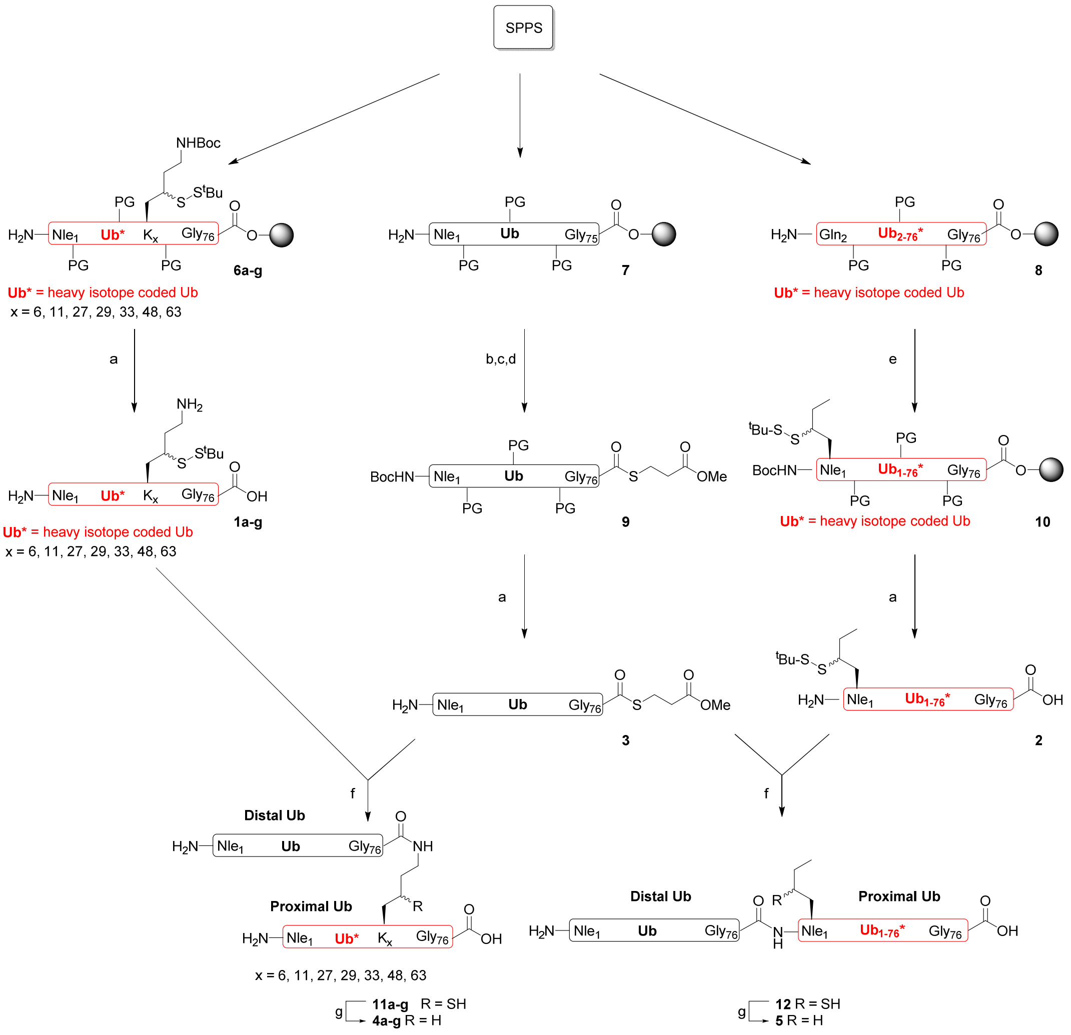

Supplementary Figure 1. Synthesis of all eight neutron-encoded diUbs. Reagents and conditions: (a) TFA/H_2O/PhOH/*i*Pr_3SiH (90.5/5/2.5/2); (b) Boc_2O, DiPEA, DCM (c) 20% HFIP/DCM; (d) HCl·H-Gly-$S(CH_2)_2CO_2Me$, EDC, HOBt, DCM; (e) S^tBu γ-thioNle[1], PyBOP, DiPEA, NMP; (f) 0.10 M TCEP, 0.25 M MPAA, 6 M Gnd·HCl, 0.15 M sodium phosphate, pH 7.6, 37°C; (g) 75 mM VA-044, 0.25 M TCEP, 100 mM GSH, 6 M Gnd·HCl, 0.15 M sodium phosphate, pH 7.0, 37°C. Monoubiquitins **6a-g**, **7** and **8** on resin were synthesized using solid phase peptide synthesis (SPPS). Monoubiquitins **6a-g** were liberated from the resin and deprotected using 90% TFA, yielding neutron-encoded $Ub_{1\text{-}76}$ containing γ-thioLys **1a-g**. The N-terminus of monoubiquitin **7** was protected with a Boc-protection group whereafter the protein was liberated from the resin using mild acidic conditions (20% HFIP/DCM), while protecting groups on the amino acid side chains remain intact. Methyl-3-(glycylthio)-propionate (**26**) was coupled to the liberated C-terminal glycine yielding **9**. Acid-mediated deprotection yielded $Ub_{1\text{-}76}$-thioester **3.** Resin-bound monoubiquitin **8** was elongated with γ-thioNle using the SPPS coupling conditions resulting in resin-bound monoubiquitin **10**. Neutron-encoded $Ub_{1\text{-}76}$ containing γ-thioNle **2** could be obtained after resin liberation and amino acid side chain deprotection using 90% TFA. Native chemical ligation (NCL) reactions between $Ub_{1\text{-}76}$-thioester **3** and neutron-encoded $Ub_{1\text{-}76}$ containing γ-thioLys **1a-g** or neutron-encoded $Ub_{1\text{-}76}$ containing γ-thioNle **2** yielded neutron-encoded diUb **11a-g** and **12**. Finally, the remaining sulfur atom was removed using desulfurization under radical conditions[2] to obtain the native diUb sequences **4a-g** and **5**.

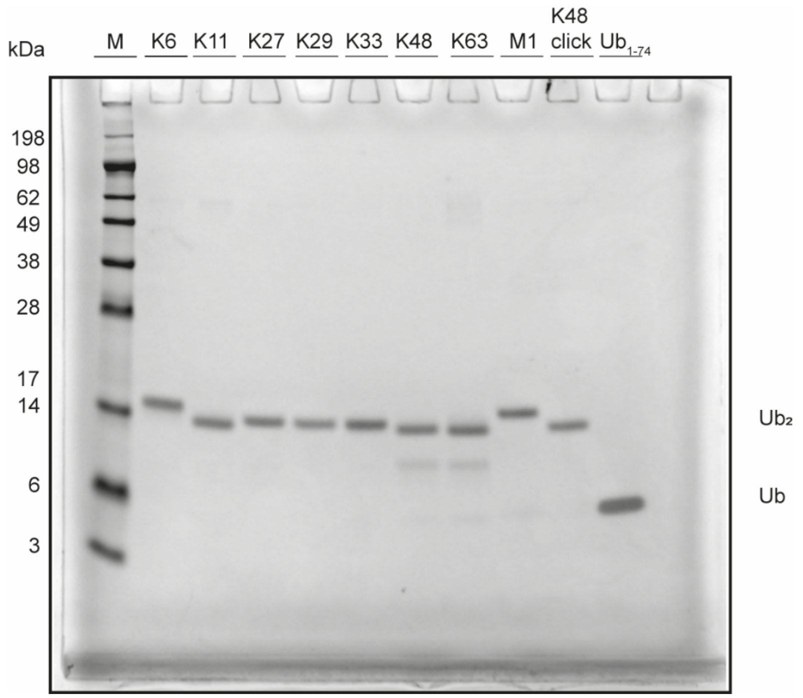

Supplementary Figure 2. Full gel image of all eight neutron-encoded diUbs (4a-g and 5), non-hydrolysable clicked Lys48 diUb (23) and Ub_{1-74} on 12% Bis-Tris gel related to Figure 2c in the main paper.

Protein marker = SeeBlue™ Plus2 Pre-stained Protein Standard. Loading: ~0.5 µg/lane. *Method*; Stock solutions of all Ubs were diluted in a buffer containing 50 mM TRIS and 100 mM NaCl to a concentration of 3.5 µM. 20 µL of sample buffer was added to 40 µL of diluted stock solution. The samples were boiled for 5 min at 95°C and 15 µL was loaded on a precast 12%Bis-Tris gel (Invitrogen). The samples were resolved by gel electrophoresis with MES running buffer and the gel was stained with InstantBlue™ staining (n=1).

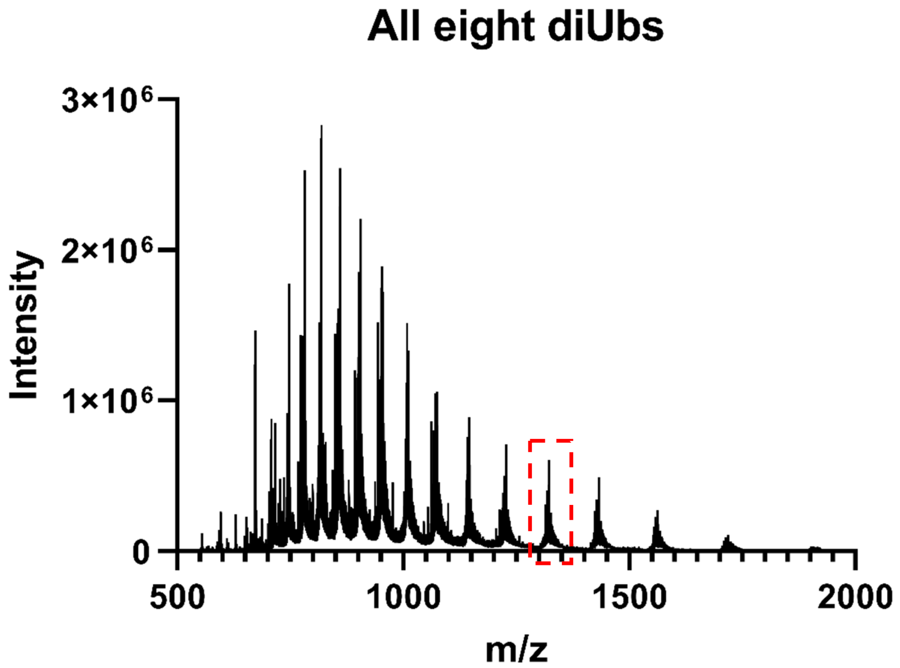

Supplementary Figure 3. Sum spectrum of the main peak of the chromatogram of the mix of all eight neutron-encoded diUbs (2.5 µM each). Full mass range of sum spectrum of the right panel of Figure 3a in the main paper.

Method; All eight neutron-encoded diUbs were mixed in equimolar amount (Final concentration = 2.5 µM for each diUb). This mixture was 16 times diluted with 0.1% FA in MQ and 8 µL of this solution was injected for a HPLC-MS run (LC-MS – System 2 – Gradient 3). Spectra 115:228 were combined for the sum spectrum. Source data are provided as a source data file.

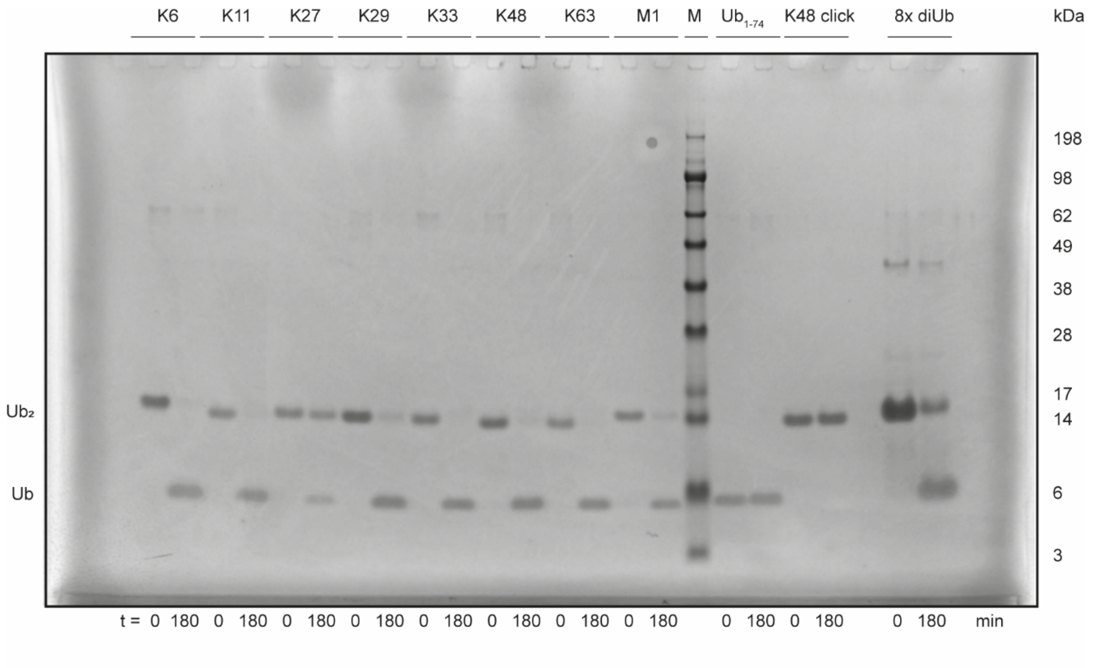

Supplementary Figure 4. USP21 mediated hydrolysis of all eight neutron-encoded diUb molecules (4a-g and 5) analysed by SDS-PAGE related to Figure 3b in the main paper. Protein marker = SeeBlue™ Plus2 Pre-stained Protein Standard. Loading: 10 µL reaction mixture. *Method*; Quenched samples of the reaction mixtures were boiled for 5 min. at 95°C and 15 µL was loaded on gel. The samples were resolved by gel electrophoresis on a 12% Bis-Tris gel (Invitrogen) with MES running buffer and the gel was stained with InstantBlue™ staining (n=1).

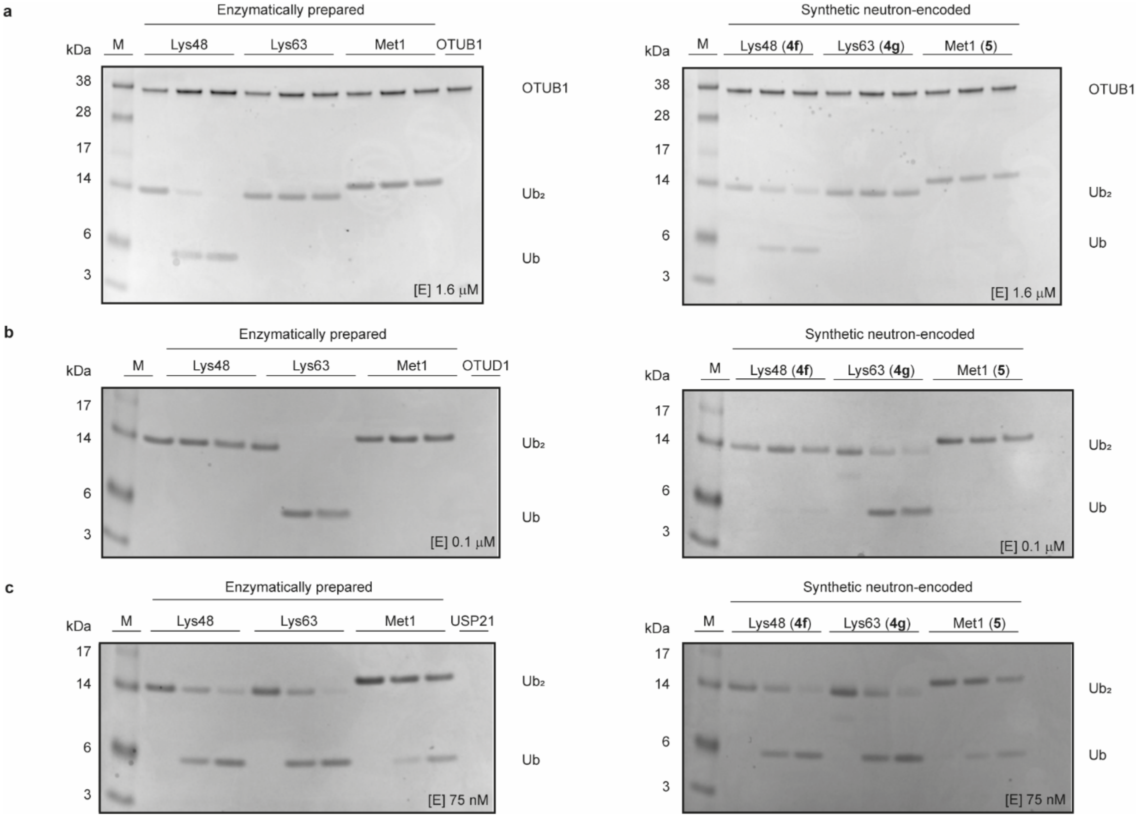

Supplementary Figure 5. DUB mediated hydrolysis of synthetic K48-, K63- and Met1-linked diUb molecules (4f, 4g and 5) and enzymatically prepared K48-, K63- and Met1-linked diUb molecules analysed by SDS-PAGE. a, OTUB1. **b,** OTUD1. **C,** USP21.

Protein marker = SeeBlue™ Plus2 Pre-stained Protein Standard. Loading: 5 µL reaction mixture. *Method*; Quenched samples of the reaction mixtures were boiled for 5 min. at 95°C and 7.5 µL was loaded on gel. The samples were resolved by gel electrophoresis on a 12% Bis-Tris gel (Invitrogen) with MES running buffer and the gel was stained with InstantBlue™ staining (n=1).

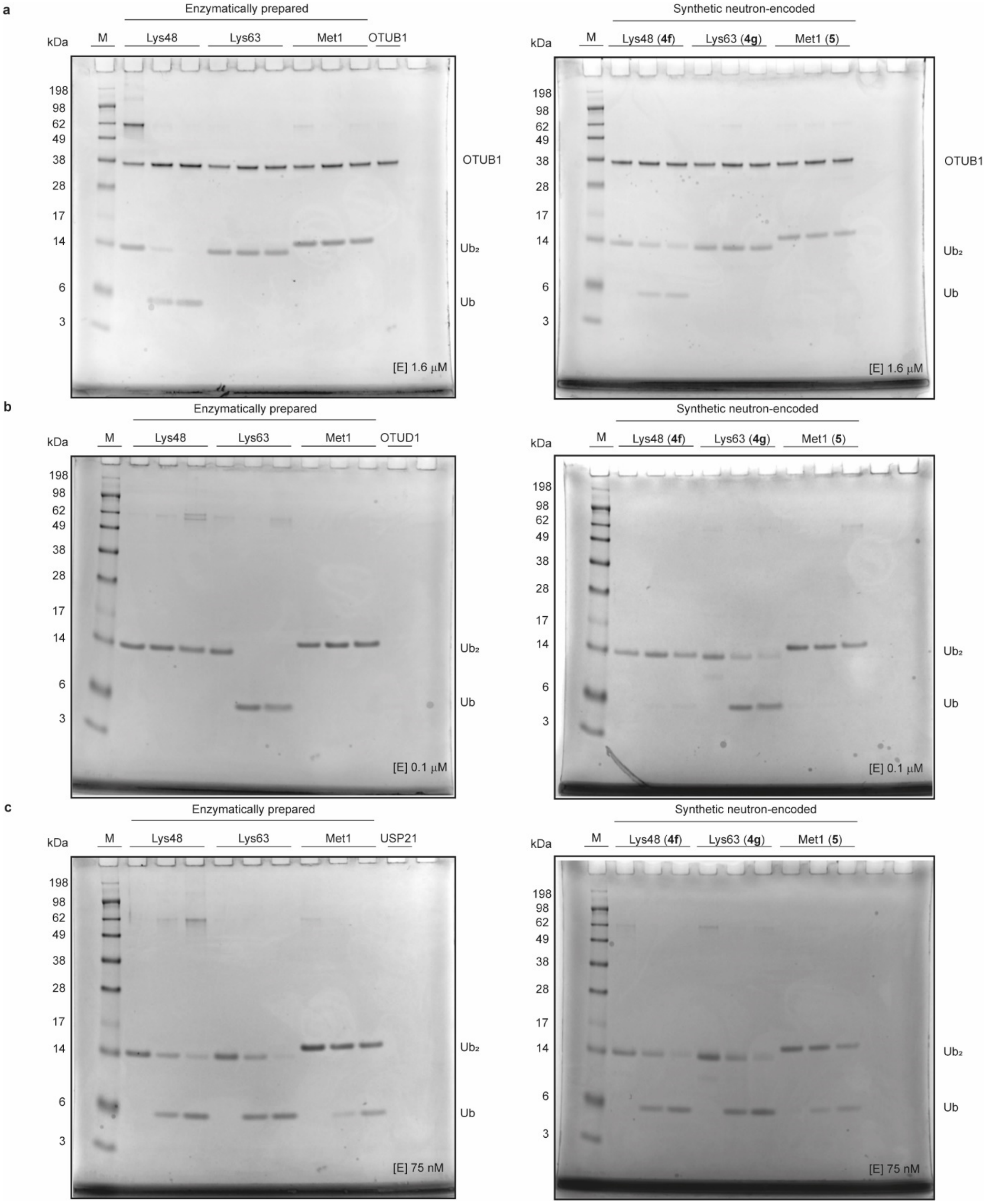

Supplementary Figure 6. Full gel images of DUB mediated hydrolysis of K48-, K63- and Met1-linked diUb molecules (4f, 4g and 5) and enzymatically prepared K48-, K63- and Met1-linked diUb molecules analysed by SDS-PAGE related to Supplementary Figure 5. a, OTUB1. **b,** OTUD1. **c,** USP21.

Protein marker = SeeBlue™ Plus2 Pre-stained Protein Standard. Loading: 5 µL reaction mixture. *Method*; Quenched samples of the reaction mixtures were boiled for 5 min. at 95°C and 7.5 µL was loaded on gel. The samples were resolved by gel electrophoresis on a 12% Bis-Tris gel (Invitrogen) with MES running buffer and the gel was stained with InstantBlue™ staining (n=1).

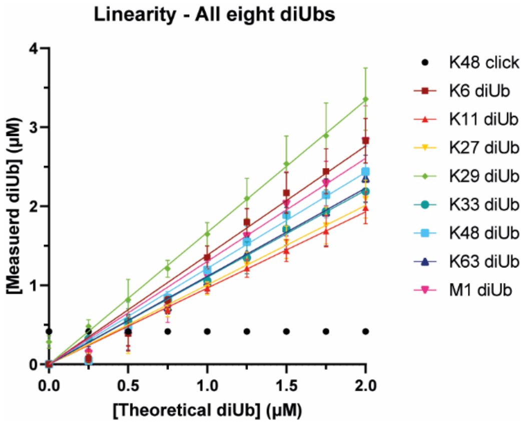

Supplementary Figure 7. Assay window and linearity determination. The linearity of the measured signal (signal height vs concentration) and the assay window were analysed by measuring standard curves of all eight neutron-encoded diUbs over the concentration range of 0.0-2.0 µM in the presence of non-hydrolysable clicked Lys48 diUb as internal standard in three separately performed experiments on three different days. Showing a reliable assay and measurement window between 0.5 and 2.0 µM. Concentrations of below 0.5 µM were divergent from the theoretically present amount of diUb, meaning these concentrations cannot be determined accurately. The graph represents the results as means ± SEM (n=3 individual experiments). Source data are provided as a source data file.

Method; All eight diUb solutions were mixed to obtain a solution containing equimolar amounts of these diUbs over a range of 0.0 – 2.0 µM diUbs (0.00, 0.25, 0.50, 0.75, 1.00, 1.25, 1.50, 1.75, 2.00 µM) in a buffer containing 50 mM TRIS and 20 mM NaCl, pH 7.55. These solution (2 µL) was diluted using a mixture containing 0.4 µL 10% TFA in MQ, 1.6 µL 0.1% FA in MQ and 12 µL of the internal standard solution (1 µL of 5.0 µM internal standard diluted with 11 µL of 0.1% FA in MQ) and 8 µL of these solutions were injected for an HPLC-MS run. The obtained data was quantified using LaCyTools and the standard data analysis protocol. The area under the curve for the internal standard was linked to the concentration of non-hydrolysable K48 present in the analysed solution. The measured concentrations of diUb analytes were calculated using their areas under the curve and plotted against the theoretical amount of diUb present in the mixture.

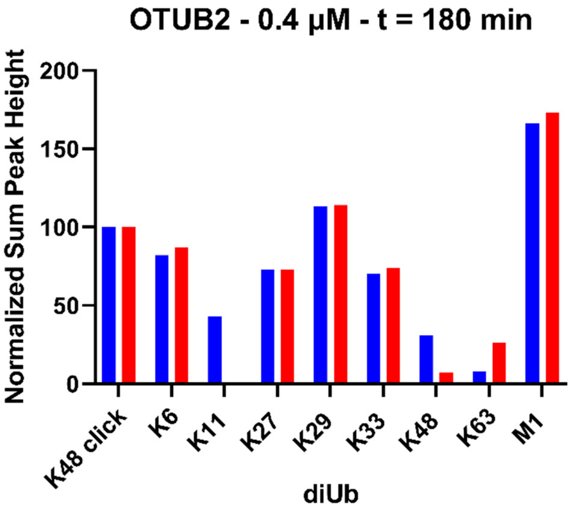
OTUB2 - 0.4 µM - t = 180 min
Normalized Sum Peak Height
0
50
100
150
200
K48 click
K6
K11
K27
K29
K33
K48
K63
M1
diUb

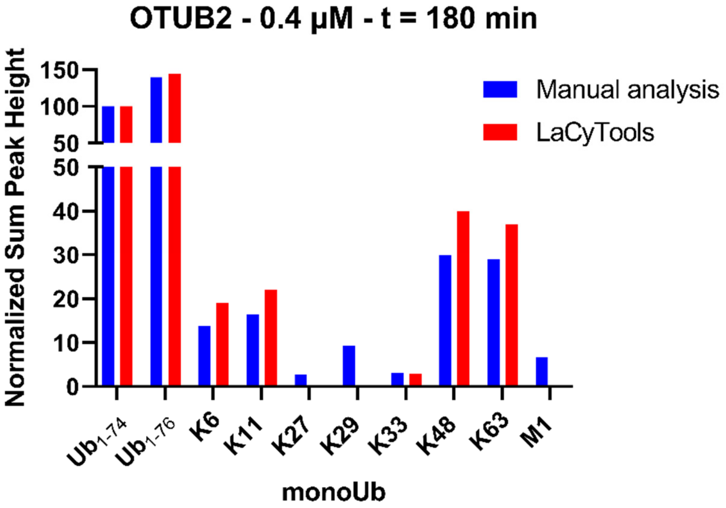
OTUB2 - 0.4 µM - t = 180 min
Normalized Sum Peak Height
150
100
50
50
40
30
20
10
0
Ub1-74
Ub1-76
K6
K11
K27
K29
K33
K48
K63
M1
monoUb
Manual analysis
LaCyTools

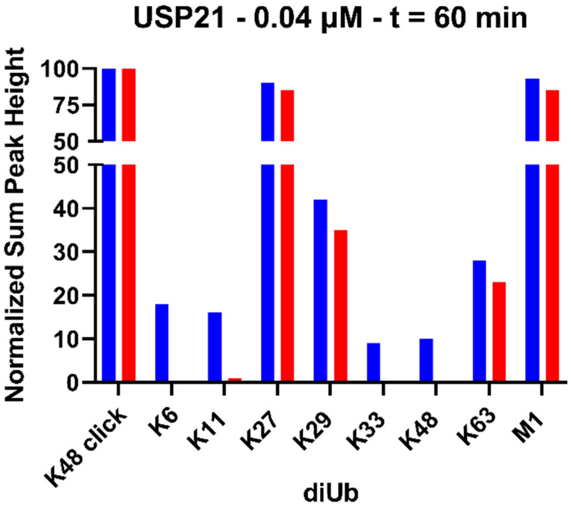
USP21 - 0.04 µM - t = 60 min
Normalized Sum Peak Height
100
75
50
50
40
30
20
10
0
K48 click
K6
K11
K27
K29
K33
K48
K63
M1
diUb

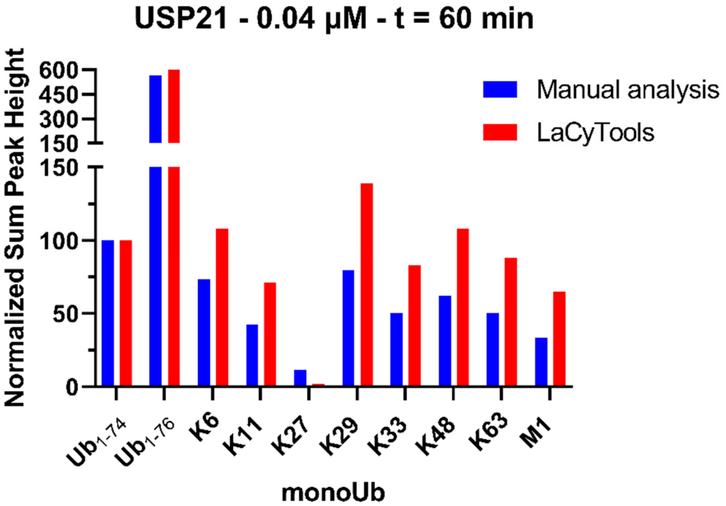
USP21 - 0.04 µM - t = 60 min
Normalized Sum Peak Height
600
450
300
150
150
100
50
0
Ub1-74
Ub1-76
K6
K11
K27
K29
K33
K48
K63
M1
monoUb
Manual analysis
LaCyTools

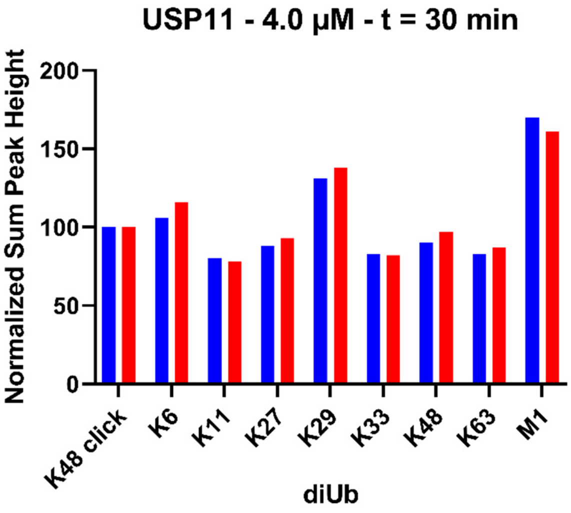
USP11 - 4.0 µM - t = 30 min
Normalized Sum Peak Height
0
50
100
150
200
K48 click
K6
K11
K27
K29
K33
K48
K63
M1
diUb

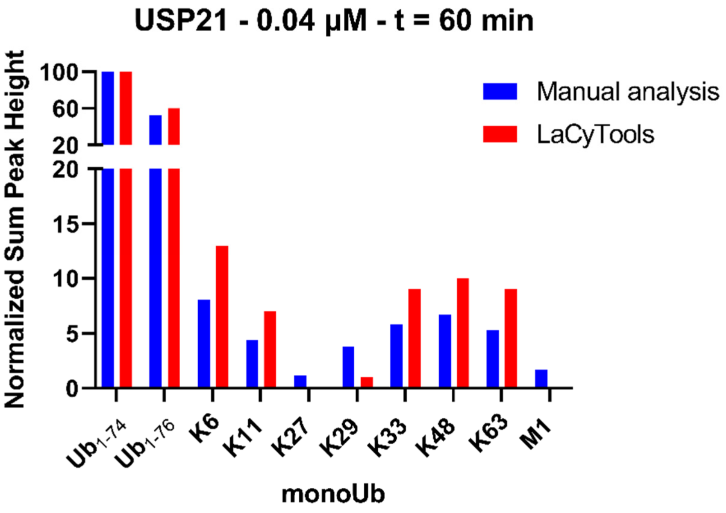
USP21 - 0.04 µM - t = 60 min
Normalized Sum Peak Height
100
60
20
20
15
10
5
0
Ub1-74
Ub1-76
K6
K11
K27
K29
K33
K48
K63
M1
monoUb
Manual analysis
LaCyTools

Supplementary Figure 8. Comparison between LaCyTools analysis versus manual data analysis. Three MS spectra from different DUBs cleavage assays, at different enzyme concentrations and different timepoints were selected to be analysed using LaCyTools analysis and manually. LaCyTools calculates the area under the curve of the relevant charge states. The areas under the curve of all relevant charge states (diUb $z=10^{+}$ to $z=25^{+}$ and monoUb $z=5^{+}$ to $z=13^{+}$) with the correct quality parameters were summed and normalized to the sum area of the internal standard. For the manual analysis the TIC count of the highest peak from an isotope pattern distribution was selected to serve as value for the peak height. The peak height of all relevant charge states (diUb $z=10^{+}$ to $z=25^{+}$ and monoUb $z=5^{+}$ to $z=13^{+}$) were summed and normalized to the sum peak height of the internal standard. For the manual analysis no quality control or background subtraction was performed (n=1). Source data are provided as a source data file.

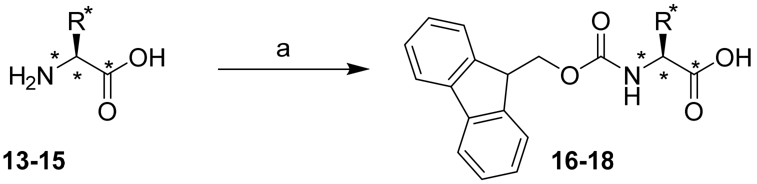

13-15
13 = R = $CH(CH_3)_2$ = Valine
14 = R = $CH_2CH(CH_3)_2$ = Leucine
15 = R = $CH_2(CH_3)(CH_2CH_3)$ = Isoleucine

16-18
16 = R = $CH(CH_3)_2$ = Fmoc -Val
17 = R = $CH_2CH(CH_3)_2$ = Fmoc-Leu
18 = R = $CH_2(CH_3)(CH_2CH_3)$ = Fmoc-Ile

Supplementary Figure 9. Fmoc-protection of neutron-encoded Val, Leu and Ile. Atoms marked with an asterisk (*) are ^{13}C or ^{15}N. Reagents and conditions: (a) 10% Na_2CO_3 in H_2O (11 mL/mmol AA) and 1.2 eq. FmocOSu in 1,4-dioxane (7 mL/mmol FmocOSu). Neutron-encoded Val, Leu and Ile were protected with an Fmoc-protection group using FmocOSu in an alkaline solution.

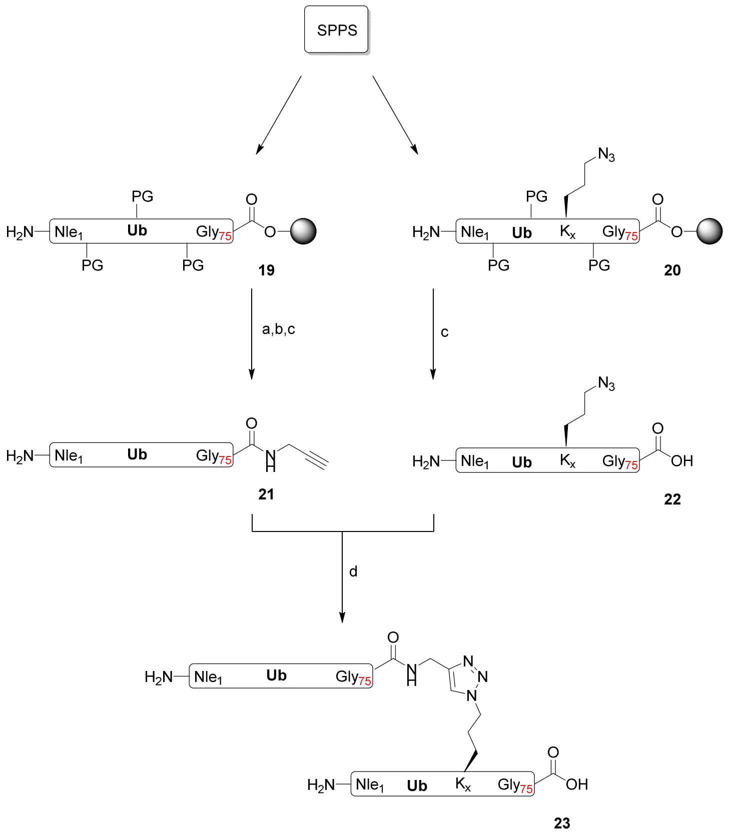

Supplementary Figure 10. Synthesis of internal standard non-hydrolyzable clicked Lys48 diUb. Reagents and conditions: (a) 20% HFIP/DCM; (b) Propargylamine (PA), PyBOP, DiPEA, DCM; (c) TFA/H_2O/PhOH/*i*Pr_3SiH (90.5/5/2.5/2); (d) $CuSO_4 \cdot 5H_2O$; Sodium Ascorbate; TBTA-analogue[3], 8M Urea, 100 mM phosphate buffer, pH 7. Monoubiquitins **19** and **20** on resin were synthesized using linear solid phase peptide synthesis (SPPS). Monoubiquitin **19** was liberated from the resin using mild acidic conditions (20% HFIP/DCM), while protecting groups on the amino acid side chains remain intact. Propargylamine was coupled to the liberated C-terminal glycine followed by acid-mediated deprotection yielded $Ub_{1\text{-}75}$-PA **21**. Monoubiquitin **20** was liberated from the resin and deprotected using 90% TFA, yielding $Ub_{1\text{-}75}$ (K48= ʟ-azido-ornithine) **22**. Subsequent CuAAC of alkyne **21** and azide **22** yields final K48 click diUb **23**.

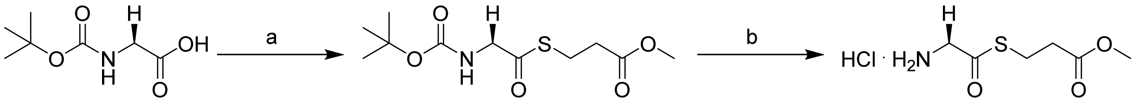

24 **25** **26**

Supplementary Figure 11. Synthesis of methyl-3-(glycylthio)-propionate. Reagents and conditions: (a) Methyl 3-mercaptopropionate, EDC, DMAP, DCM; (b) 4M HCl in dioxane. Boc-protected glycine **24** was converted into Boc-protected methyl-3-(glycylthio)-propionate **25** using methyl 3-mercaptopropionate together with the carboxyl activating carbodiimide EDC, and the nucleophilic catalyst DMAP. Hydrochloride salt of glycine thioester **26** was obtained by treatment of Boc-protected methyl-3-(glycylthio)-propionate **25** with hydrochloric acid in dioxane.

Supplementary Table 1. Amino acid sequence of designed proximal ubiquitins.

Sites of neutron-encoded amino acids, pseudoproline building blocks, dipeptides, thioLys and thioNle incorporation are indicated and relevant structures are shown below

Diubiquitin Linkage	Protein sequence for synthesizer	Amount of neutron-encoded amino acids	Mass difference in comparison with unlabeled ubiquitin	Mass difference in comparison with previous ubiquitin
K6	NleQIFVKTLTGKTITLEVEPSDTIENVKAKIQDKEGIPPDQQRLIFAGKQLEDGRTLSDYNIQKESTLHLVLRLRGG	1xV + 1xL	+13 Da	13 Da
K11	NleQIFVKTLTGKTITLEVEPSDTIENVKAKIQDKEGIPPDQQRLIFAGKQLEDGRTLSDYNIQKESTLHLVLRLRGG	3xV + 1xI	+25 Da	12 Da
K27	NleQIFVKTLTGKTITLEVEPSDTIENVKAKIQDKEGIPPDQQRLIFAGKQLEDGRTLSDYNIQKESTLHLVLRLRGG	3xV + 2xL + 1xI	+39 Da	14 Da
K29	NleQIFVKTLTGKTITLEVEPSDTIENVKAKIQDKEGIPPDQQRLIFAGKQLEDGRTLSDYNIQKESTLHLVLRLRGG	3xV + 2xL + 3xI	+53 Da	14 Da
K33	NleQIFVKTLTGKTITLEVEPSDTIENVKAKIQDKEGIPPDQQRLIFAGKQLEDGRTLSDYNIQKESTLHLVLRLRGG	3xV + 4xL + 3xI	+67 Da	14 Da
K48	NleQIFVKTLTGKTITLEVEPSDTIENVKAKIQDKEGIPPDQQRLIFAGKQLEDGRTLSDYNIQKESTLHLVLRLRGG	3xV + 4xL + 5xI	+81 Da	14 Da
K63	NleQIFVKTLTGKTITLEVEPSDTIENVKAKIQDKEGIPPDQQRLIFAGKQLEDGRTLSDYNIQKESTLHLVLRLRGG	3xV + 6xL + 5xI	+95 Da	14 Da
M1	NleQIFVKTLTGKTITLEVEPSDTIENVKAKIQDKEGIPPDQQRLIFAGKQLEDGRTLSDYNIQKESTLHLVLRLRGG	4xV + 6xL + 6xI	+108 Da	13 Da

K = thioLys   Nle = thioNle   XX = dipeptide building block   X = neutron-encoded amino acid

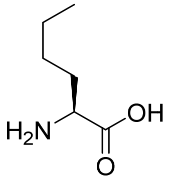

Nle

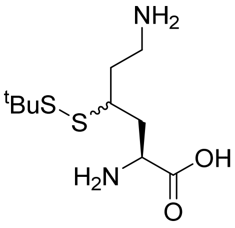

thioLys

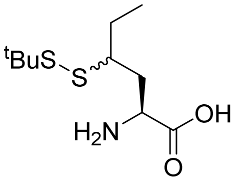

thioNle

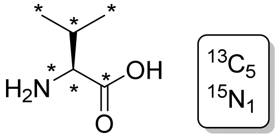

Isotope labelled Valine
$^{13}C_5H_{11}{}^{15}NO_2$
mw: 123,08 g/mol

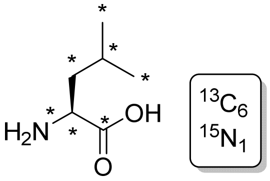

Isotope labelled Leucine
$^{13}C_6H_{13}{}^{15}NO_2$
mw: 138,09 g/mol

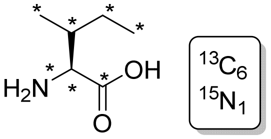

Isotope labelled Isoleucine
$^{13}C_6H_{13}{}^{15}NO_2$
mw: 138,09 g/mol

Supplementary Table 2. Purified recombinant DUBs used in this work.

	DUB family	DUB	Domain/ Length / Fragment	Tag	UniProt accession number	Species / Origin / Organism	Expression system / Host / Source	Stock concentration	Source or reference
1	Ubiquitin-specific proteases (USPs)	USP2	Full length (1-369)	GST-tagged	O75604-4	Human	E.coli	0.5 mg/mL, 73 kDa 6.9 µM	Obtained from Ubiquigent, #64-0014-050
2		USP7	Full length (1-1102)	GST-tagged, clvd	Q93009	Human	E.coli BL21(DE3) Rosetta2	10.13 µM	*In-house.* [Kim, 2019][4]
3		USP8	Full length (1-1118)	untagged	P40818-1	Human	E.coli	0.5 mg/mL, 128 kDa 3.9 µM	Obtained from Ubiquigent, # 64-0053-050
4		USP9x	CD (1554-1995)	GST-tagged	Q93008	Human	E.coli	0.5 mg/mL, 79 kDa 6.35 µM	Obtained from Ubiquigent, # 64-0017-050
5		USP9x	CD(1551-1970)	His-tagged, clvd	Q93008	Human	E.coli BL21(DE3) Rosetta2	228 µM	*In-house.* [Paudel, 2019][5]
6		USP10	Full length (1-798)	C-terminal 6-His tag	Q14694-1	Human	Sf21 Insect cells (baculovirus)	88 kDa 10 µM	Obtained from BioTechne, # E-592-050
7		USP11 Isoform 2	FL(1-920) Canocical (43-963)	N-terminal His-tag, clvd	G5E9A6 (P51784-2)	Human	E.coli	13.5 mg/mL 136 µM	*In-house*, [Luna-Vargas 2011][6]
8		USP16FL isoform 3	FL (22-823) Q141H, EY480DN	N-terminal His-tag	Q9Y5T5-3	Human	Sf9 Insect cells (baculovirus)	12 µM	*In-house*, [Mons, 2021][7]
9		USP21	CD(196-565)	N-terminal His-SUMO, clvd	Q9UK80	Human	E.coli BL21(DE3) Rosetta2	79.95 µM	*In-house*, [Ye, 2011][8]
10		USP32	Full-length (1-1604)	6-His tag N-terminus	Q8NFA0	Human	Sf9 Insect cells (baculovirus)	182 kDa 26 µM	*In-house*, [Sapmaz, 2019][9]
11		USP34	CD(1892-2241)	6-His tag	Q70CQ2	Human	E. coli	42 kDa 12.9 µM	Obtained from LifeSensors, # DB506
12	Ovarian tumour proteases (OTUs)	Cezanne	CD (129-438)	C-terminal 6-His tag and N-terminal GST tag, clvd	Q6GQQ6	Human	E.coli Rosetta2 (DE3) pLacI	362 µM	Gift from David Komander, [Mevissen, 2013][10]
13		OTULIN	Full length (1-352)	GST-tagged	Q96BN8-1	Human	E. coli	0.5 mg/mL, 67 kDa 7.46 µM	Obtained from Ubiquigent, # 64-0017-050
14		OTUB1	Full length (1-271)	N-terminal His6-GST tag, clvd	Q96FW1	Human	E.coli Rosetta2 pLacI	943 µM	Gift from David Komander, [Mevissen, 2013][10]
15		OTUB2	Full length (1-234)	6-His tag, clvd	Q96DC9	Human	E.coli BL21(DE3)	2.4 mg/mL 81 µM	*In house*, [Nanao, 2004][11]
16		OTUD1	CD (287-481)	N-terminal His6-GST tag, clvd	Q5VV17	Human	E.coli Rosetta2 pLacI	223 µM	Gift from David Komander, [Mevissen, 2013][10]
17		OTUD2	Full length (1-348)	N-terminal His6-GST tag, clvd	Q5VVQ6	Human	E. coli Rosetta2 pLacI	10 mg/mL 274 µM	Gift from David Komander, [Mevissen, 2013][10]

18		OTUD3	CD (52-209)	N-terminal His6-GST tag, clvd	Q5T2D3	Human	E. coli Rosetta2 pLacI	220 µM	Gift from David Komander, [Mevissen, 2013][10]
19	MJD+	JOSD1	1-202	6-His tagged	Q15040	Human	E.coli	0.5 mg/mL, 26 kDa 19 µM	Obtained from Ubiquigent, # 67-0006-001
20		JOSD2	1-188	6-His tagged	Q8TAC2-1	Human	E.coli	0.5 mg/mL, 23 kDa 21.7 µM	Obtained from Ubiquigent, # 67-0006-001
21		ATAXN3L	Full length (1-355)	6-His tagged	Q9H3M9	Human	E.coli	0.5 mg/mL, 43 kDa 11.6 µM	Obtained from Ubiquigent, # 67-0006-001
22	JAB/MPN/Mov34 metalloenzyme (JAMM)	AMSH	Full length (1-424)	GST-tagged, clvd	O95630	Human	E.coli	33 µM	*In-house,* This work
23		AMSH-LP	Full length (1-436)	6-His tagged	Q96FJ0	Human	E.coli	0.5 mg/mL, 52 kDa 9.6 µM	Obtained from Abcam, # ab139776
24		RPN11/ RPN8	RPN11(2-239)/ RPN8(1-179)	N-terminal 6-His tagged, C-terminal StrepII tagged	P43588/ Q08723	Saccharomyces cerevisiae	E.coli BL21 (DE3)	88 µM	*In-house,* [Hameed, 2019][12]

Supplementary Table 3. Alignment file/table

m/z	**Seconds**
599.3889	200
619.6895	197
767.6107	217
777.8871	218
803.0705	217
901.7223	230
905.3077	238
947.0356	226

Supplementary Table 4. LaCyTools settings

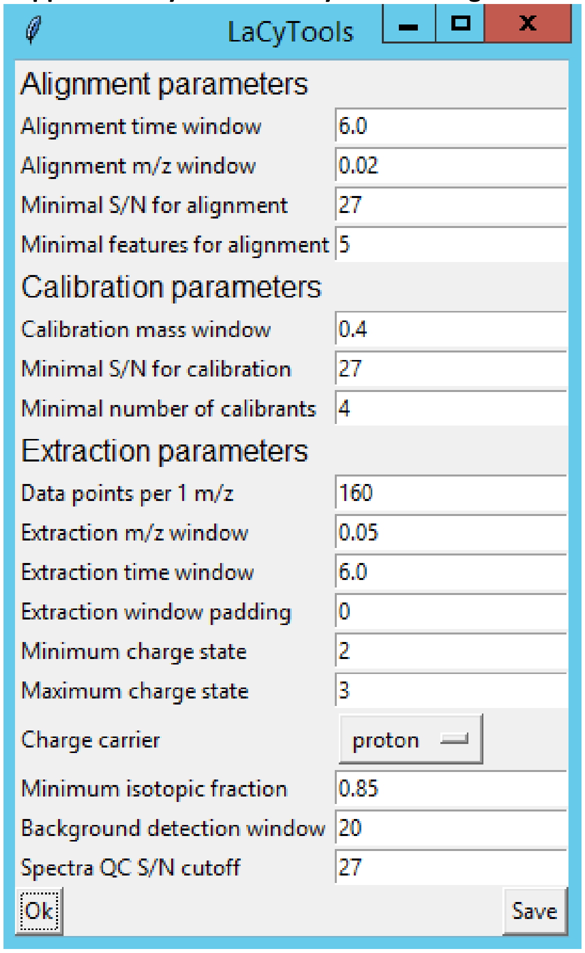

LaCyTools

**Alignment parameters**	
Alignment time window	6.0
Alignment m/z window	0.02
Minimal S/N for alignment	27
Minimal features for alignment	5
**Calibration parameters**	
Calibration mass window	0.4
Minimal S/N for calibration	27
Minimal number of calibrants	4
**Extraction parameters**	
Data points per 1 m/z	160
Extraction m/z window	0.05
Extraction time window	6.0
Extraction window padding	0
Minimum charge state	2
Maximum charge state	3
Charge carrier	proton
Minimum isotopic fraction	0.85
Background detection window	20
Spectra QC S/N cutoff	27

Ok Save

Supplementary Table 5. Analytes and calibrants – LaCyTools

Peak	RT	Mass Window	Time Window	Minimal charge state	Maximum charge state	Calibrant
_C756_H1255_N211_O233	227			10	25	X
_C747_hC11_H1260_N208_hN2_O235	230			10	25	X
_C737_hC21_H1260_N206_hN4_O235	230			10	25	X
_C725_hC33_H1260_N204_hN6_O235	230			10	25	X
_C713_hC45_H1260_N202_hN8_O235	230			10	25	X
_C701_hC57_H1260_N200_hN10_O235	230			10	25	X
_C689_hC69_H1260_N198_hN12_O235	230			10	25	X
_C677_hC81_H1260_N196_hN14_O235	230			10	25	X
_C666_hC92_H1260_N194_hN16_O235	237			10	25	X
_C375_H625_N103_O116	218			5	13	X
_C379_H631_N105_O118	218			5	13	X
_C368_hC11_H631_N103_hN2_O118	218			5	13	X
_C358_hC21_H631_N101_hN4_O118	218			5	13	X
_C346_hC33_H631_N99_hN6_O118	218			5	13	X
_C334_hC45_H631_N97_hN8_O118	218			5	13	X
_C322_hC57_H631_N95_hN10_O118	218			5	13	X
_C310_hC69_H631_N93_hN12_O118	218			5	13	X
_C298_hC81_H631_N91_hN14_O118	218			5	13	X
_C287_hC92_H631_N89_hN16_O118	218			5	13	X

_C = carbon atom = 12.00000
_hC = heavy carbon atom = 13.0033550
_H = hydrogen atom = 1.007825
_N = nitrogen atom = 14.003074
_hN = heavy nitrogen atom = 15.000109
_O = oxygen atom = 15.994915

Supplementary Table 6. AMSH expression: PCR reaction

PCR Reaction Component	Volume (µL)	Final Concentration
ThermoPol Reaction Buffer (10X)	2.5 µL	1X
Deoxynucleotide (dNTP) Solution Mix (10 mM)	1 µL	200 µM
Forward Primer (10 µM stock)	0.6 µL	0.24 µM
Reverse Primer (10 µM stock)	0.6 µL	0.24 µM
DNA Template (MelJuSo cDNA)	1 µL	N/A
Vent DNA Polymerase (New England Biolabs)	0.3 µL	0.6 unit
$MgCl_2$	0.6 µL	2 mM
Nuclease-free water	Bring reaction to a final volume of 30 µL	

Supplementary Table 7. AMSH expression: PCR cycle condition

Steps	Temperature	Time	# of cycle
Initial Denaturation	95 °C	2 min	
Denaturation	95 °C	30 sec	34
Annealing	56 °C	30 sec	
Elongation	72 °C	1 min 30sec	
Final Elongation	72 °C	5 min	

Supplementary Table 8. Sequence of the cloning primers for AMSH

**Name**	**Sequence**
AMSH Forward	cagggacccggtATGTCTGACCATGGAGATGTGAGCC
AMSH Reverse	cgaggagaagcccggttaTCGAAGGTCTGTGATGGTCACTGC

Supplementary Methods

Chemical Synthesis

General. All commercially available reagents and solvents were purchased from various suppliers (listed in Supplementary Table 6) and used as received.

Supplementary Table 6. Building blocks, reagents and solvents for chemical synthesis.

Compound	Abbreviation	CAS#	Source or reference
Building blocks			
**L-Valine 13C5,15N1**	Val $^{13}C_5$ $^{15}N_1$	202407-30-5	Cortecnet #CCN5000P01
**L-Leucine 13C6,15N1**	Leu $^{13}C_6$ $^{15}N_1$	202406-52-8	Cortecnet #CCN1600P01
**L-Isoleucine 13C6,15N1**	Ile $^{13}C_6$ $^{15}N_1$	202468-35-7	Cortecnet #CCN1300P01
**N-(tert-Butoxycarbonyl)glycine**	Boc-Gly-OH	4530-20-5	Sigma Aldrich #15420
Chemicals			
**Sodium carbonate**	Na_2CO_3	497-19-8	Acros Organics #424280025
***N*-(9-Fluorenylmethoxycarbonyloxy)succinimide**	FmocOSu	82911-69-1	Chem-Impex #00147
**Hydrochloric acid, 37%**	HCl	7647-01-0	Sigma Aldrich #258148
**Acetic acid**	AcOH	64-19-7	Sigma Aldrich #27225
**Sodium Chloride**	NaCl	7647-14-5	Sigma Aldrich #S9625
**Sodium sulfate**	Na_2SO_4	7757-82-6	Sigma Aldrich #239313
**Methyl 3-mercaptopropionate**		2935-90-2	Sigma Aldrich #108987
**4-(Dimethylamino)pyridine**	DMAP	1122-58-3	ChemImpex #00120
**1-Ethyl-3-(3-dimethylaminopropyl)carbodiimide hydrochloride**	EDC	25952-53-8	Combi blocks #SS-7536
**Sodium bicarbonate**	$NaHCO_3$	144-55-8	Acros #123360050
**Magnesium sulfate**	$MgSO_4$	22189-08-8	Acros # 196850010
**Hydrogen chloride, 4N solution in 1,4-dioxane**	4M HCl in 1,4-dioxane	7647-01-0	Acros # 388368000
Solvents			
**1,4-dioxane (AR)**		123-91-1	Biosolve #4240501
**Diethyl ether (AR)**	Et_2O	60-29-7	Biosolve #5280501
**Ethyl acetate (AR)**	EtOAc	141-78-6	VWR # 23880324

**n-Heptane (AR)**	Hept	142-82-5	VWR #24551324
**Dichloromethane (AR)**	DCM	75-09-2	VWR #23 366 327
**Dimethyl sulfoxide (AR)**	DMSO(AR)	67-68-5	Biosolve #4470501
**Acetonitrile (ULC-MS)**	CH_3CN (ULC-MS)	75-05-8	Biosolve #1204102
**Formic Acid (ULC-MS)**	FA (ULC-MS)	64-18-6	Biosolve #6914143
**Methanol-d4**	MeOD- d_4	811-98-3	Cortecnet #D024H
**DMSO-d6**	DMSO- d_6	2206-27-1	Cortecnet #D010H

Thin Layer Chromatography (TLC) analysis was performed on TLC plates from Merck (aluminum sheets precoated with Silica (SiO_2) Kieselgel 60 F_{254} neutral) and compounds were visualized by UV absorption (254 nm) and/or by using a solution of ninhydrin (15 g/L) and acetic acid (30 mL/L) in ethanol or a solution of $KMnO_4$ (7.5 g/L) and K_2CO_3 (50 g/L) in water followed by charring.

Flash column chromatography (FCC) purifications were purified by a Büchi Sepacore automatic flash chromatography system X10/X50. The Büchi Sepacore system was equipped with two Büchi Pump Modules C-605, a Büchi Control Unit C-620, a Büchi Fraction Collector C-660 and a Büchi UV Photometer C-640. The silica columns were purchased at BUCHI® (FlashPure EcoFlex Silica) and were packed with silica with an irregular particle size (40-63 µm) and pore size (55-75 Å). FCC purifications were performed with the indicated eluent.

Nuclear magnetic resonance (NMR) spectra were recorded on a Bruker UltraShield™ Avance II™ 300 (300 MHz for ^{1}H, 75 MHz for ^{13}C) at 298 K using the residual solvent as internal standard (^{1}H: δ 7.26 ppm for $CDCl_3$, 3.31 ppm for MeOD and 2.50 ppm for DMSO-d_6. ^{13}C: δ 77.16 ppm for $CDCl_3$, 49.00 ppm for MeOD and 39.52 ppm for DMSO-d_6). Chemical shifts (δ) are given in ppm and coupling constants (*J*) are quoted in hertz (Hz). Multiplicities are reported as a s (singlet), d (doublet), t (triplet), q (quartet), b (broad) and m (multiplet) or combinations thereof.

LC-MS analysis of crude reaction mixtures and pure products were performed on a Waters ACQUITY UPLC H-class System equipped with Waters ACQUITY Quaternary Solvent Manager (QSM), Waters ACQUITY UPLC Photodiode Array (PDA) eλ Detector (λ = 210-800 nm) and Waters ACQUITY UPLC Protein BEH C18 column (1.7 µm, 2.1 x 50 mm) (Column Temp = 40 °C) and LCT Premier Orthogonal Acceleration Time of Flight Mass Spectrometer (*m/z* = 100-1600) in ES+ mode. Samples were run with a 1.6 minute gradient (run time 3 min) using three mobile phases: 100% H_2O, 100% CH_3CN and 50% H_2O + 50% CH_3CN + 2.5% FA (flow rate = 0.5 mL/min).

**Time (min)**	**100% H_2O (%)**	**100% CH_3CN (%)**	**50% H_2O + 50% CH_3CN + 2.5% FA(%)**
**0.00**	94.0	2.0	4.0
**0.20**	94.0	2.0	4.0
**1.80**	0.0	96.0	4.0
**2.15**	0.0	96.0	4.0
**2.20**	94.0	2.0	4.0
**3.00**	94.0	2.0	4.0

Pure products were run with a 7 minute gradient (run time 10 min) using three mobile phases: 100% H_2O, 100% CH_3CN and 50% H_2O + 50% CH_3CN + 2.5% FA (flow rate = 0.5 mL/min).

Time (min)	100% H_2O (%)	100% CH_3CN (%)	50% H_2O + 50% CH_3CN + 2.5% FA(%)
**0.00**	94.0	2.0	4.0
**0.50**	94.0	2.0	4.0
**7.50**	0.0	96.0	4.0
**8.00**	0.0	96.0	4.0
**8.10**	94.0	2.0	4.0
**10.00**	94.0	2.0	4.0

Electrospray Ionization (ESI) high-resolution mass spectrometry (HR-MS) was carried on a Waters XEVO-G2 XS Q-TOF mass spectrometer equipped with an electrospray ion source in positive mode (capillary voltage: 3.0 kV, desolvation gas flow: 900 L h^{-1}, temperature: 60 °C) with a resolution R = 22,000 using 200 pg μL^{-1} Leu-Enk (m/z = 556.2771) as a "lock mass".

General procedure A: Fmoc protection of neutron-encoded amino acid
AA ($^{13}C_x$, $^{15}N_1$) (1 eq.) was dissolved in 10% Na_2CO_3 in H_2O (11 mL/mmol AA) and the solution was cooled to 0 °C. FmocOSu (1.2 eq.) was dissolved in 1,4-dioxane (7 mL/mmol FmocOSu). The FmocOSu solution was added dropwise to the cooled amino acid solution over the course of 2h. The reaction mixture was allowed to warm to room temperature and was stirred for 16 hours. H_2O (11 mL/mmol AA) was added to the reaction mixture resulting in a clear solution. The reaction solution was washed with Et_2O (3x 30 mL/mmol AA). The aqueous layer was acidified to pH ~1 with conc. HCl and extracted with EtOAc (2x 16mL/mmol AA and 1x 30 mL/mmol AA). The combined organic phase was washed with BRINE (2x 25 mL/ mmol AA), dried over Na_2SO_4 and concentrated *in vacuo*. The resulting residue was purified by Büchi flash column chromatography (100% n-Hept → 100% EtOAc) to yield the pure Fmoc-protected neutron-encoded amino acid.

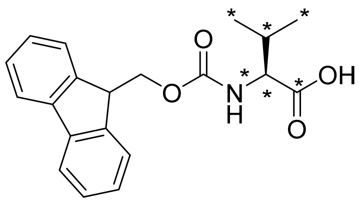

(((9*H*-fluoren-9-yl)methoxy)carbonyl)-L-valine-$^{13}C_5{}^{15}N_1$ (16)
Prepared according to general procedure A, starting from L-valine-$^{13}C_5{}^{15}N_1$ (250 mg; 2.03 mmol). The product was obtained as a white powder. (Yield: 576.9 mg, 1.67 mmol, 82.3%). TLC R_f =0.65 (60:40 EtOAc/n-Hept + 1 drop AcOH). ^1H-NMR (300 MHz, Methanol-*d4*): δ = 7.80 (d, *J* = 7.4 Hz, 2H, CH_{arom} Fmoc), 7.69 (dd, *J* = 7.5, 4.6 Hz, 2H, CH_{arom} Fmoc), 7.39 (t, *J* = 7.4 Hz, 2H, CH_{arom} Fmoc), 7.31 (td, *J* = 7.4, 1.2 Hz, 2H, CH_{arom} Fmoc), 4.36 (d, *J* = 7.4 Hz, 2H, CH_2 Fmoc), 4.24 (t, *J* = 7.0 Hz, 1H, CH Fmoc), 4.06 (d, *J* = 140.3 Hz, 1H, CαH), 2.16 (d, *J* =130.3 Hz, 1H, CβH), 1.23 – 0.58 (m, 6H, $C\gamma H_3$) ppm. ^{13}C-NMR (75 MHz, Methanol-*d4*): δ = 175.41 (d, *J* = 58.8 Hz, C=O), 145.38 (C_q), 145.20 (C_q), 142.59 (C_q), 128.77 (CH_{arom} Fmoc), 128.17 (CH_{arom} Fmoc), 128.14 (CH_{arom} Fmoc), 126.27 (CH_{arom} Fmoc), 120.90 (CH_{arom} Fmoc), 67.98 (CH_2 Fmoc), 61.01 (ddd, *J* = 58.8, 34.3, 12.6 Hz, Cα), 31.71 (q, *J* = 35.0 Hz, Cβ), 19.64 (dd, *J* = 35.5, 2.6 Hz, $C\gamma H_3$), 18.24 (d, *J* = 35.2 Hz, $C\gamma H_3$) ppm. LC-MS (4 → 98% ACN/H_2O); R_t = 5.09 min; calculated for [$M+H^+$] 346.11 ; found 346.15. HR-MS calculated for $^{13}C_5C_{15}H_{21}{}^{15}NO_4$ $[M+H]^+$ 346.1687; found 346.1687.

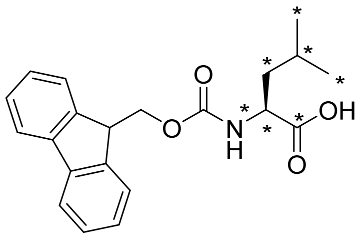

(((9*H*-fluoren-9-yl)methoxy)carbonyl)-L-leucine-$^{13}C_6{}^{15}N_1$ (17)

Prepared according to general procedure A, starting from L-Leucine-$^{13}C_6{}^{15}N_1$ (300 mg; 2.17 mmol). The product was obtained as a white powder. (Yield: 721.2 mg, 2.00 mmol, 92.2%). TLC R_f =0.68 (60:40 EtOAc/n-Hept + 1 drop AcOH). ^{1}H-NMR (300 MHz, Methanol-*d4*): δ = 7.80 (d, *J* = 7.4 Hz, 2H, CH_{arom} Fmoc), 7.68 (t, *J* = 6.5 Hz, 2H, CH_{arom} Fmoc), 7.39 (t, *J* =7.5 Hz, 2H, CH_{arom} Fmoc), 7.31 (t, *J* = 7.3 Hz, 2H, CH_{arom} Fmoc), 4.36 (d, *J* = 6.5 Hz, 2H, CH_2 Fmoc), 4.23 (t, *J* = 7.0 Hz, 1H, CH Fmoc), 4.18 (d, 2H, Cα+ …), 1.71 (d, *J* = 124.4 Hz, 1H, CγH), 1.61 (d, *J* = 127.4 Hz, 2H, $C\beta H_2$ 1.23-0.58 (m, 6H, $C\delta H_3$) ppm. ^{13}C-NMR (75 MHz, Methanol-*d4*): δ = 176.67 (dd, *J* = 59.1, 3.2 Hz, C=O), 145.39 (C_q), 145.17 (C_q), 142.59 (C_q), 128.75 (CH_{arom} Fmoc), 128.15 (CH_{arom} Fmoc), 128.12 (CH_{arom} Fmoc), 126.26 (CH_{arom} Fmoc), 120.88 (CH_{arom} Fmoc), 67.88 (CH_2 Fmoc), 53.77 (ddd, *J* = 58.9, 34.7, 12.5 Hz, Cα), 41.67 (t, *J* = 34.7 Hz, Cβ), 26.00 (qd, *J* = 34.8, 3.1 Hz, Cγ), 23.41 (dd, *J* = 35.0, 3.5 Hz, $C\delta H_3$), 21.69 (dd, *J* = 34.9, 2.1 Hz, $C\delta H_3$) ppm. LC-MS (4 → 98% ACN/H_2O); R_t = 5.38 min; calculated for [M+H$^+$] 361.12; found 361.17. HR-MS calculated for $^{13}C_6C_{15}H_{23}{}^{15}NO_4$ $[M+H]^+$ 361.1877; found 361.1877.

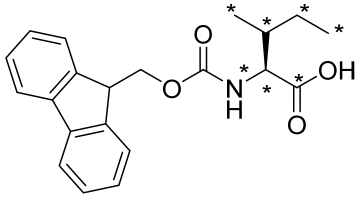

(((9*H*-fluoren-9-yl)methoxy)carbonyl)-L-isoleucine-$^{13}C_6{}^{15}N_1$ (18)

Prepared according to general procedure A, starting from L-Isoleucine-$^{13}C_6{}^{15}N_1$ (200 mg; 1.45 mmol). The product was obtained as a white powder. (Yield: 493.7 mg, 1.37 mmol, 94.5%). TLC R_f =0.70 (60:40 EtOAc/n-Hept + 1 drop AcOH). ^{1}H-NMR (300 MHz, MeOD): δ = 7.80 (d, *J* = 7.5 Hz, 2H, CH_{arom} Fmoc), 7.68 (dd, *J* = 7.4, 4.8 Hz, 2H, CH_{arom} Fmoc), 7.39 (t, *J* = 7.4 Hz, 2H, CH_{arom} Fmoc), 7.30 (td, J = 7.5, 1.3 Hz, 2H, CH_{arom} Fmoc), 4.36 (d, *J* = 7.0 Hz, 2H, CH_2 Fmoc), 4.23 (t, *J* = 7.0 Hz, 1H, CH Fmoc), 4.16 (d, *J* = 172.9 Hz, 1H, Cα), 1.77 (d, *J* = 191.4 Hz, 1H, CβH), 1.78-1.23 (m, 2H, $C\gamma H_2$), 1.23 – 0.56 (m, 6H, $C\gamma+\delta H_3$) ppm. ^{13}C-NMR (75 MHz, Methanol-*d4*): δ = 175.41 (d, *J* = 58.9 Hz), 145.38 (C_q), 145.19 (C_q), 142.59 (C_q), 128.76 (CH_{arom} Fmoc), 128.16 (CH_{arom} Fmoc), 128.13 (CH_{arom} Fmoc), 126.26 (CH_{arom} Fmoc), 120.89 (CH_{arom} Fmoc), 67.95 (CH_2 Fmoc), 60.12 (dddd, *J* = 58.9, 34.5, 12.5, 3.4 Hz, Cα), 38.34 (q, *J* = 35.0 Hz, Cβ), 26.13 (t, *J* = 34.9 Hz, $C\gamma H_2$), 16.09 (d, *J* = 35.5 Hz, $C\gamma H_3$), 11.81 (dt, *J* = 35.0, 2.5 Hz, $C\delta H_3$) ppm. LC-MS (4 → 98% ACN/H_2O); R_t = 5.38 min; calculated for [M+H$^+$] 361.12; found 361.18. HR-MS calculated for $^{13}C_6C_{15}H_{23}{}^{15}NO_4$ $[M+H]^+$ 361.1877; found 361.1873.

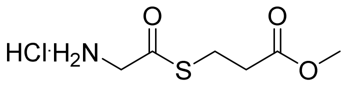

Methyl-3-(glycylthio)-propionate hydrochloride (26)

BocGlyOH (1.1 eq., 11 mmol, 1.93 g) was dissolved in DCM (50mL). Methyl 3-mercaptopropionate (1 eq, 10 mmol, 1.1mL), DMAP (0.1 eq., 1 mmol, 122 mg) and EDC (1.4 eq., 14 mmol, 2.6 g) were added and the mixture was stirred at RT overnight. EtOAc (100 mL) was added to the reaction mixture and washed with 1M HCl (aq.) (2x 100 mL), sat. $NaHCO_3$ (2x 100 mL) and BRINE (100 mL), dried over $MgSO_4$ and concentrated *in vacuo*. The crude product was obtained as a white powder. (Crude yield: 2,9 g, 10.46 mmol, 105%). The crude was dissolved in 4M HCl in dioxane (20mL) and stirred for 1h at RT. Et_2O (20 mL) was added to the reaction mixture and Gly-S(CH_2)CO_2Me precipitated from the solution. The product was dissolved in H_2O/ACN (50/50; v/v; 5 mL) and lyophilized. The product was obtained as a white solid. (Yield: 1.69 g, 7,9 mmol, 79%). ^{1}H-NMR (300 MHz, DMSO-*d6*): δ = 8.63 (s, 2H, NH_2), 4.04 (d, *J* = 1.0 Hz, 2H, $C\alpha H_2$), 3.36 (s, 3H, OCH_3), 3.15 (t, *J* = 6.8 Hz, 2H, CH_2), 2.66 (t, *J*=6.8 Hz, 2H, CH_2) ppm. LC-MS (4 → 98% ACN/H_2O); R_t = 0.51 min; calculated for [M+H$^+$] 178.05; found 178.05.

NMR Spectra

Supplementary Figure 12. ^{1}H and ^{13}C NMR spectra of Fmoc- L-Val-$^{13}C_5{}^{15}N_1$ (16)

Compound **16**: ^{1}H NMR (300.17 MHz, MeOD)

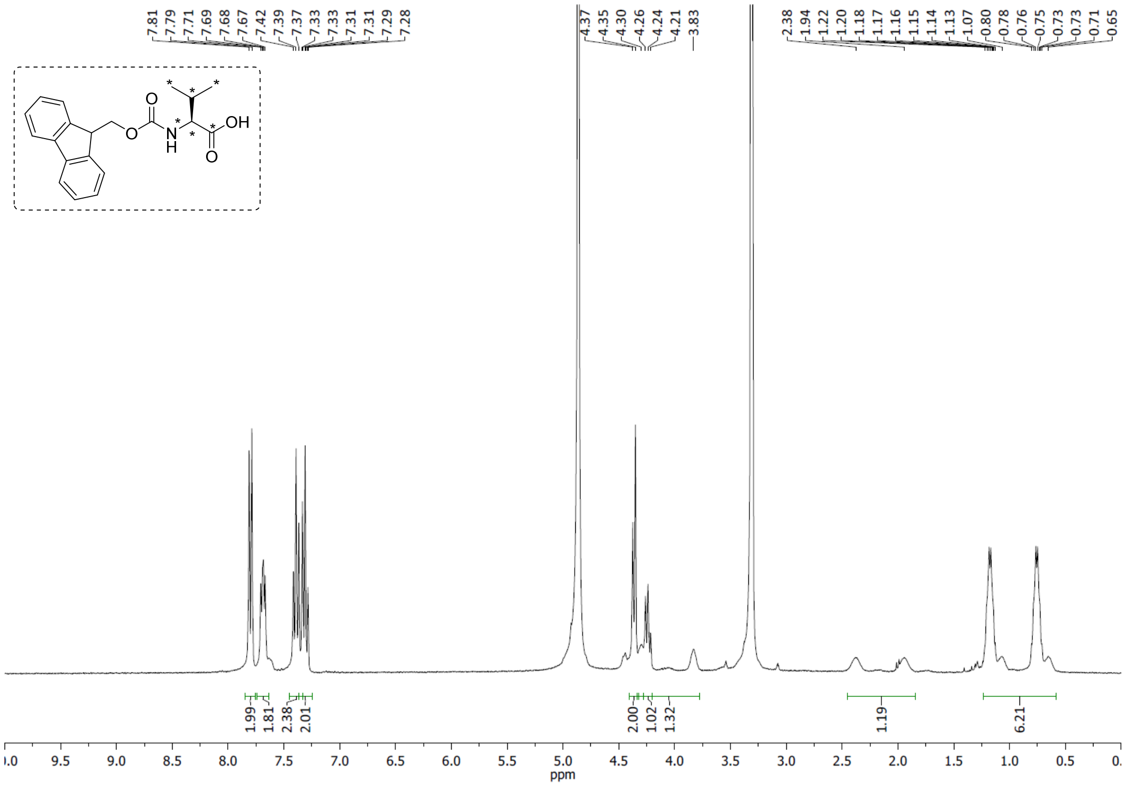

Compound **16**: ^{13}C NMR (75.47 mHz, MeOD)

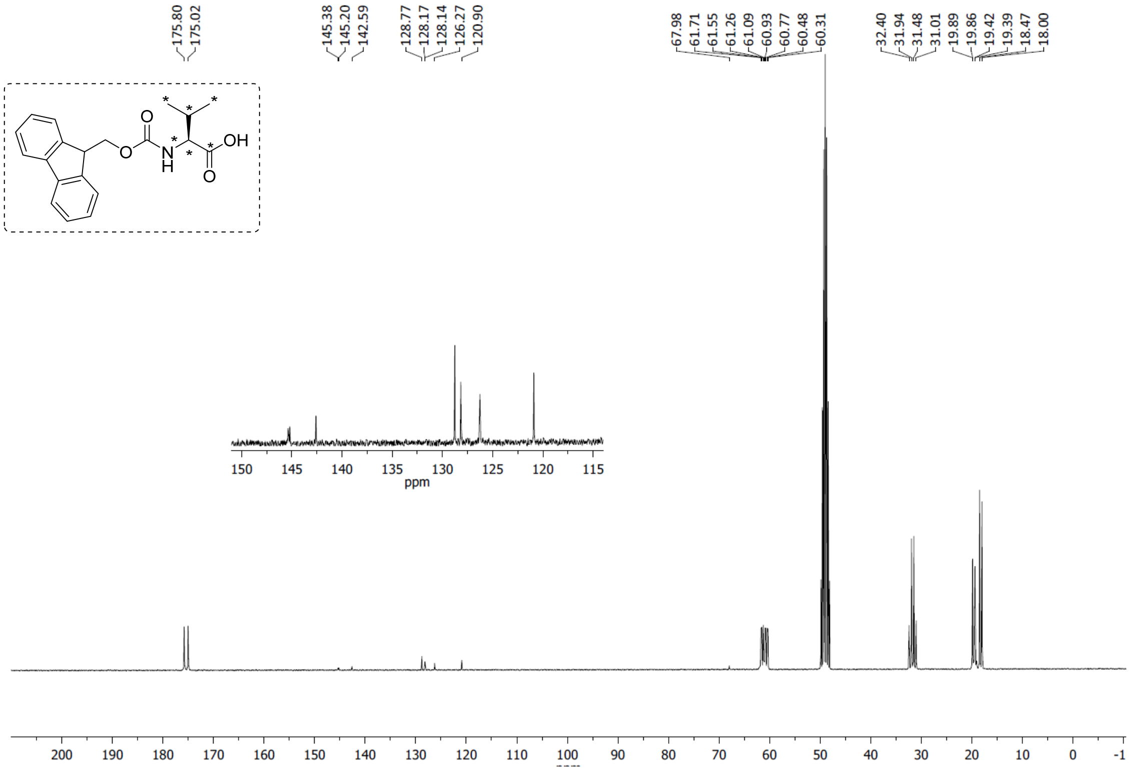

Supplementary Figure 13. ¹H and ¹³C NMR spectra of Fmoc- L-Leu-$^{13}C_6$$^{15}N_1$ (17)

Compound **17**: ^{1}H NMR (300.17 MHz, MeOD)

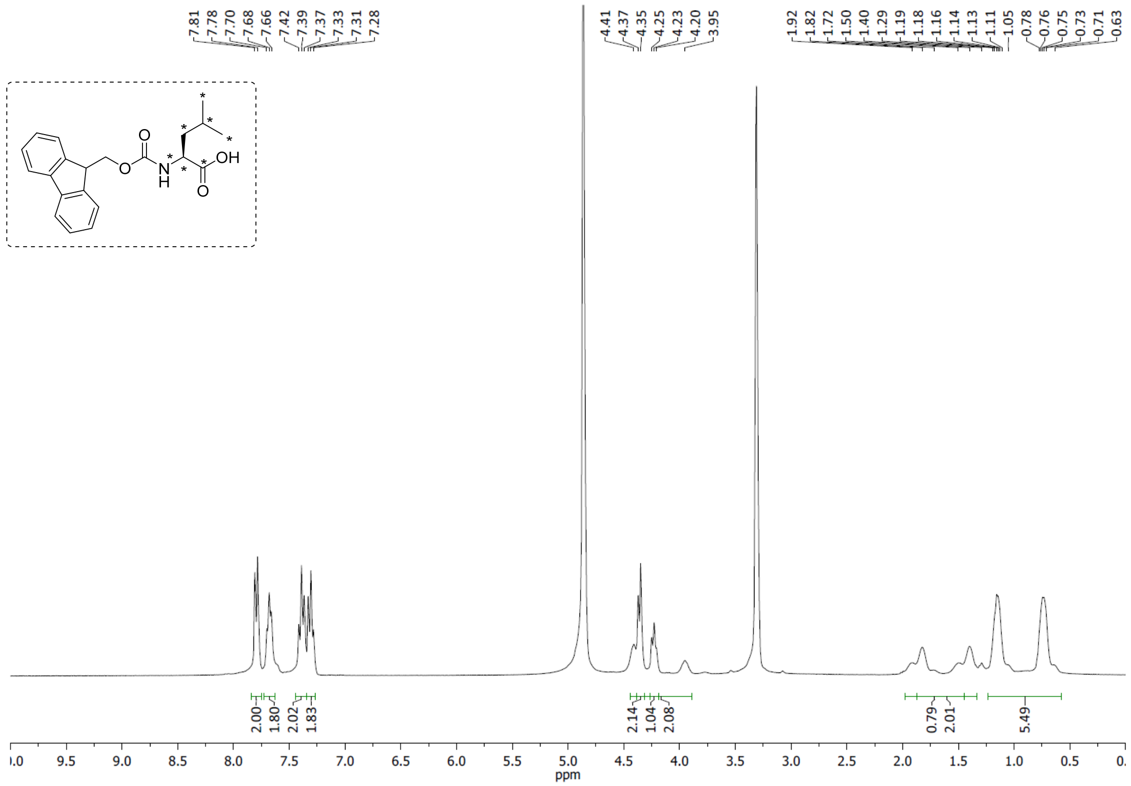

Compound **17**: ^{13}C NMR (75.47 mHz, MeOD)

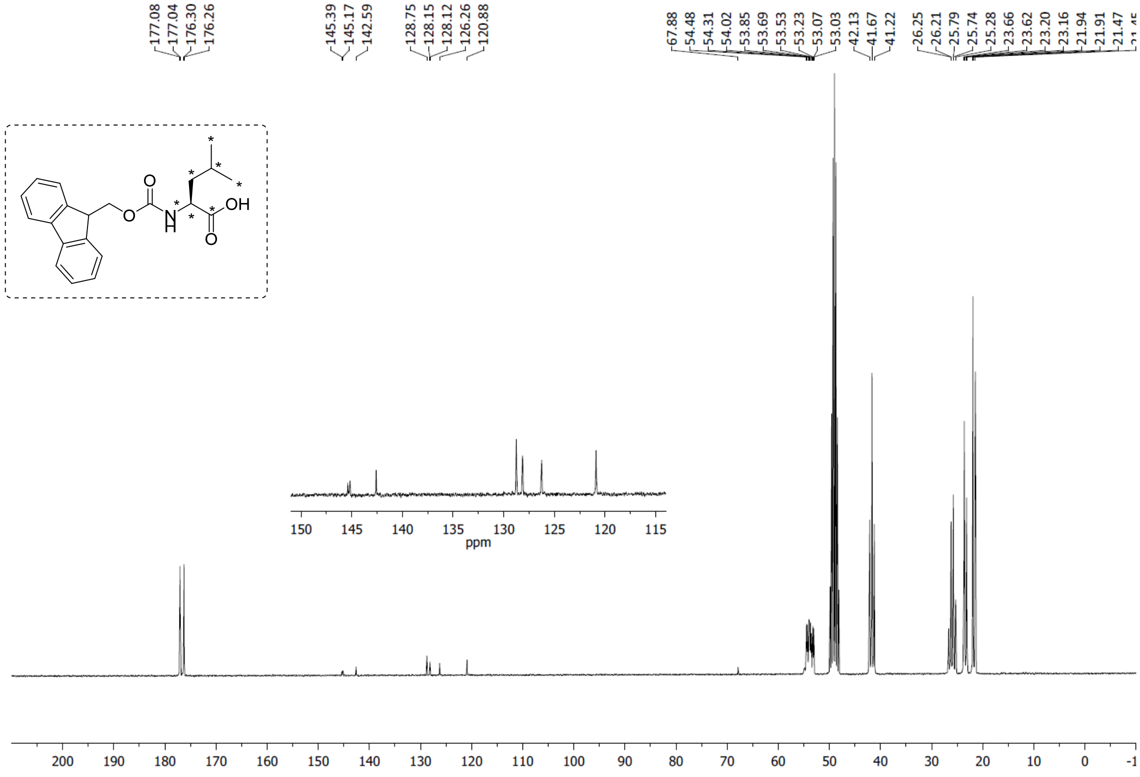

Supplementary Figure 14. ^{1}H and ^{13}C NMR spectra of Fmoc- L-Ile-$^{13}C_6{}^{15}N_1$ (18)

Compound **18**: ^{1}H NMR (300.17 MHz, MeOD)

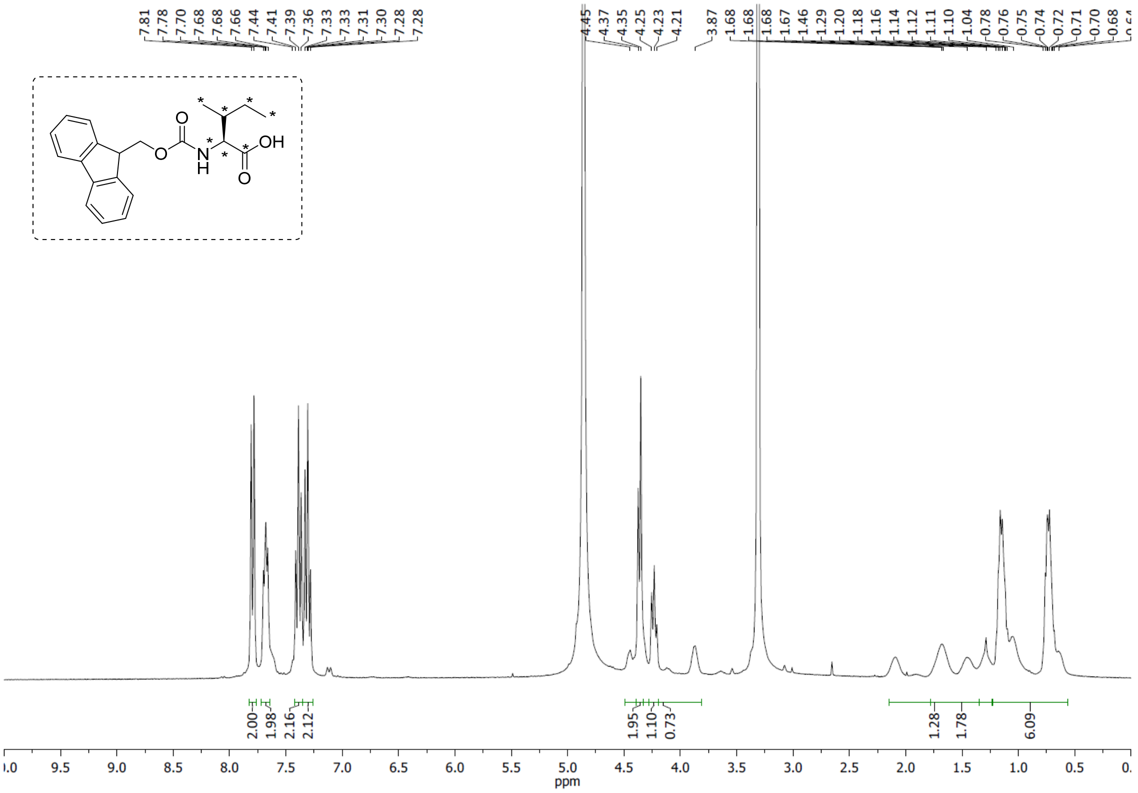

Compound **18**: ^{13}C NMR (75.47 mHz, MeOD)

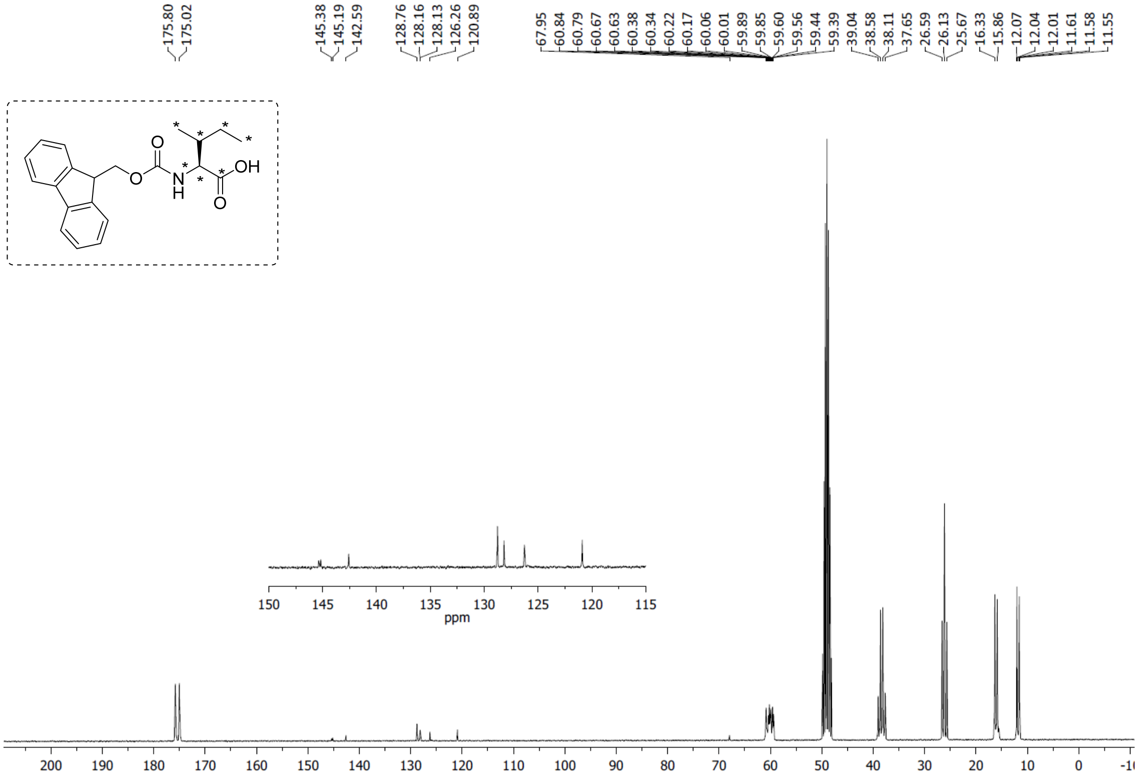

Supplementary Figure 15. ^{1}H spectra of methyl-3-(glycylthio)-propionate (26)
Compound **26**: ^{1}H NMR (300.17 MHz, DMSO)

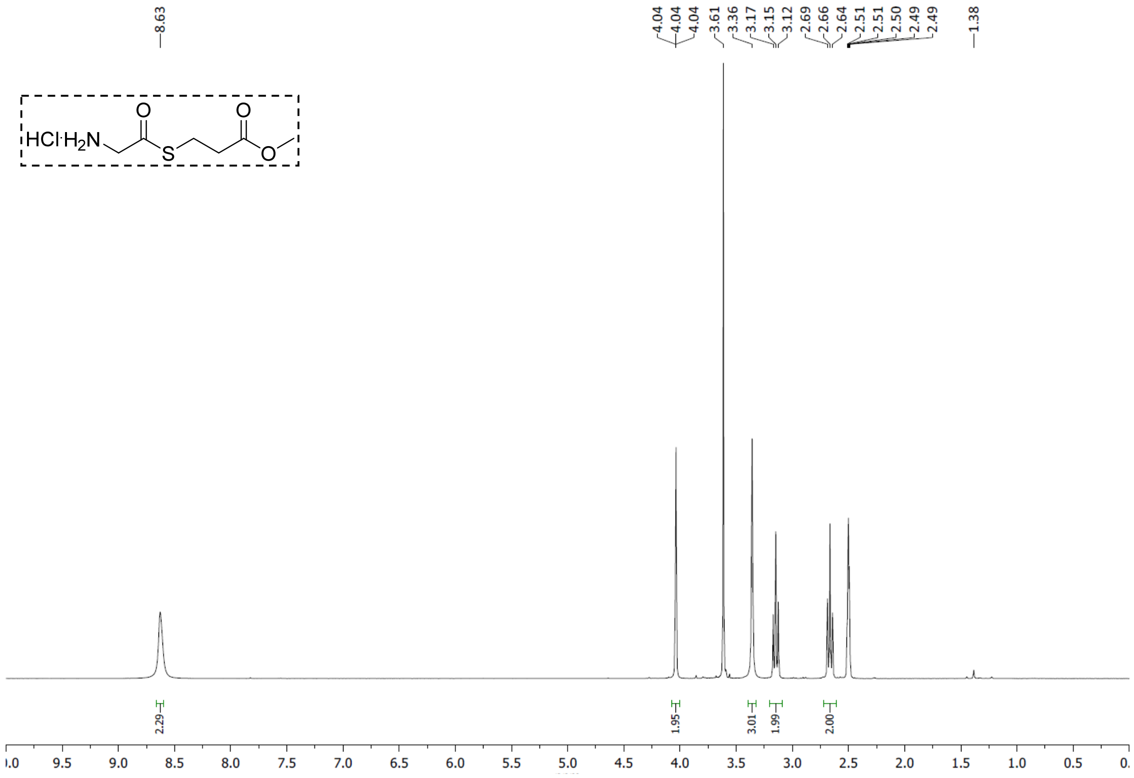

LC-MS

Analytical LC-MS data for neutron-encoded Fmoc-labelled AA's. Top panel: UV chromatogram (λ = 210-800 nm). Middle panel; TIC chromatogram (*m/z* = 100-1600). Bottom panel; Sum spectrum of mass spectra from main peak in chromatogram.

Supplementary Figure 16. Analytical LC-MS data for Fmoc-L-Val-$^{13}C_5{}^{15}N_1$ (16) ***System 1- Gradient 2***

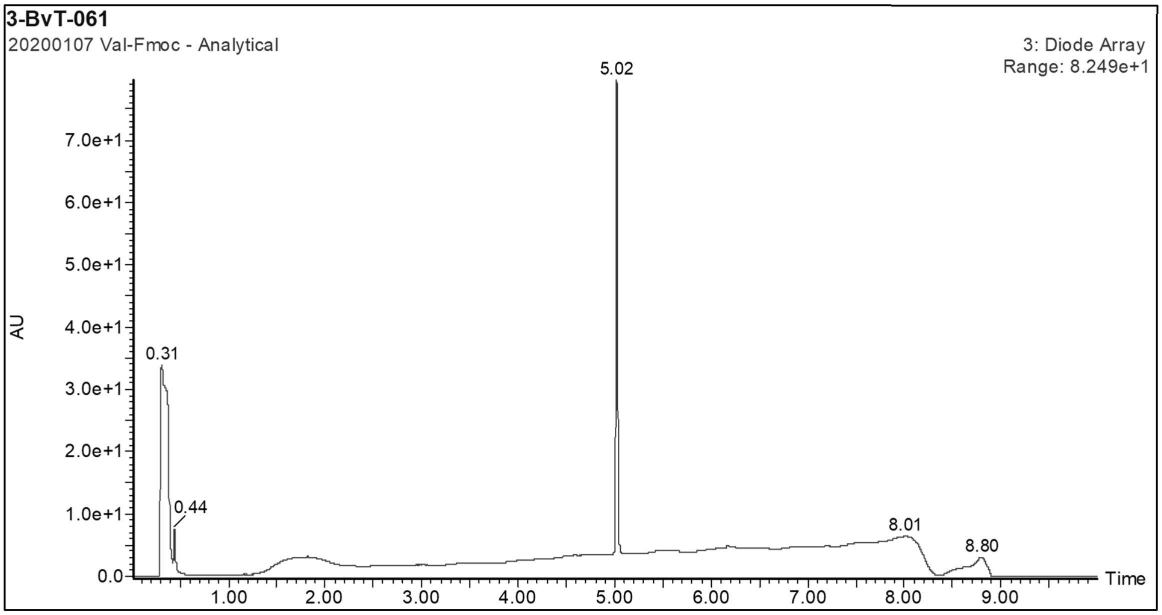

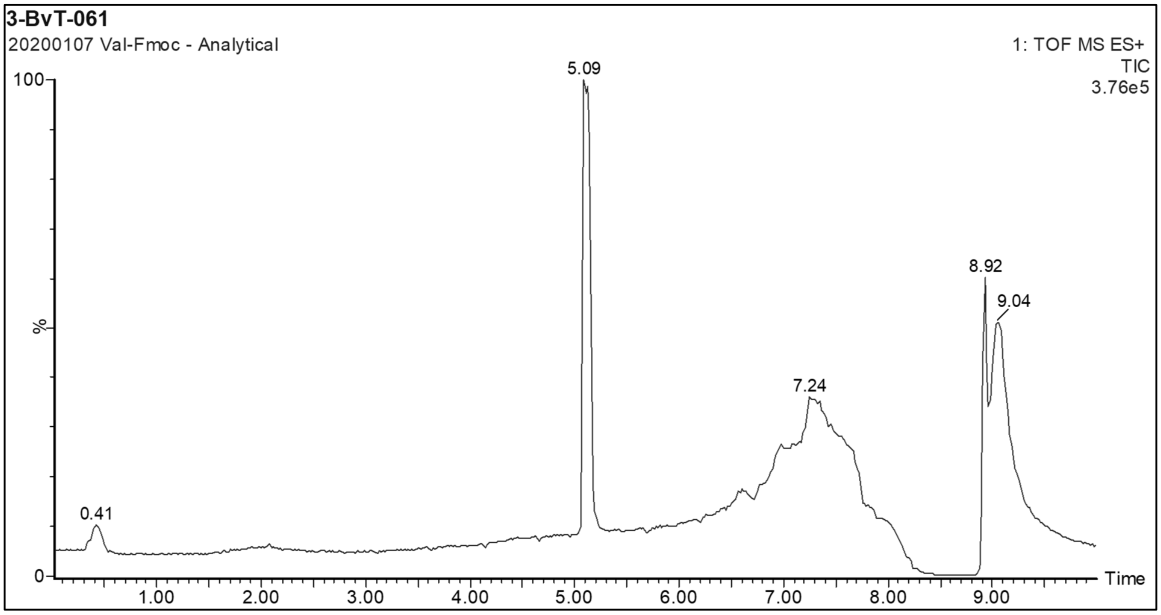

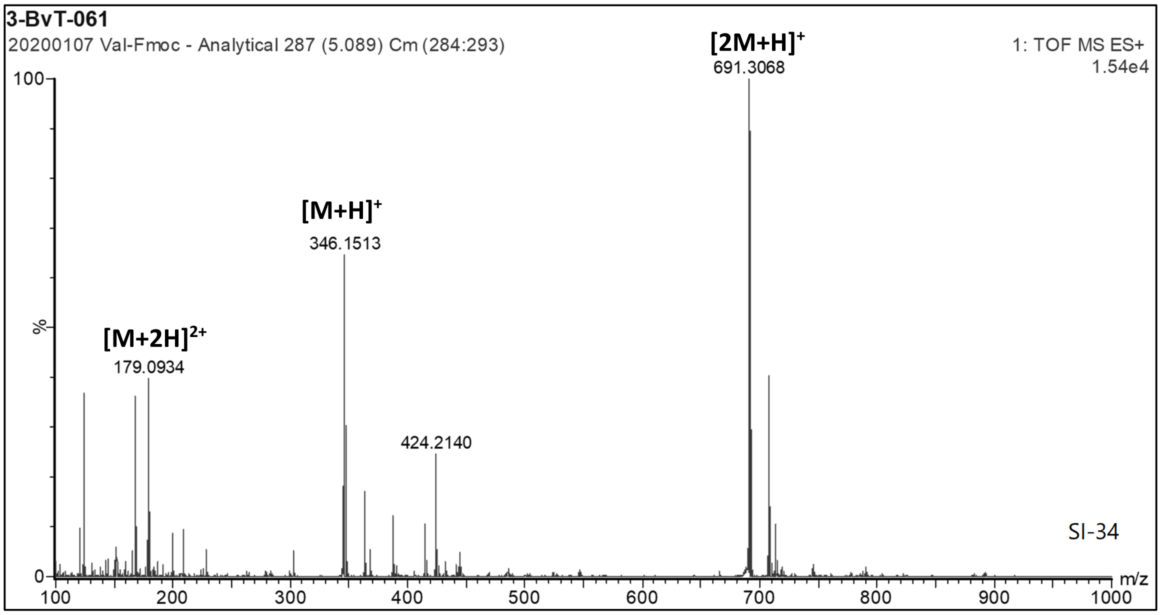

Supplementary Figure 17. Analytical LC-MS data for Fmoc-L-Leu-$^{13}C_6$$^{15}N_1$ (17) ***System 1- Gradient 2***

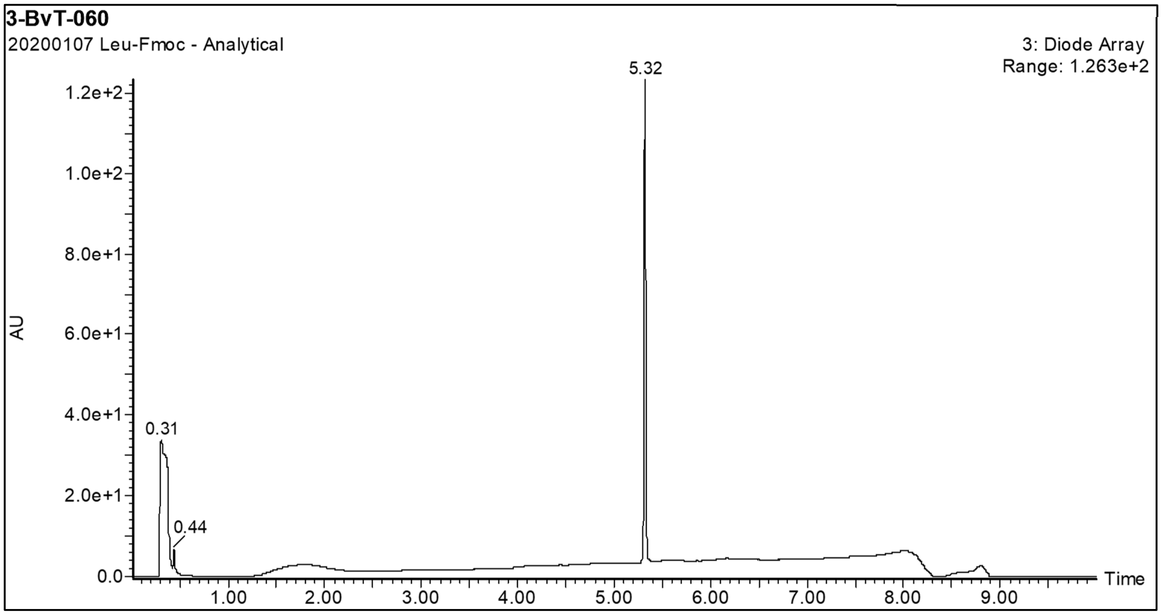

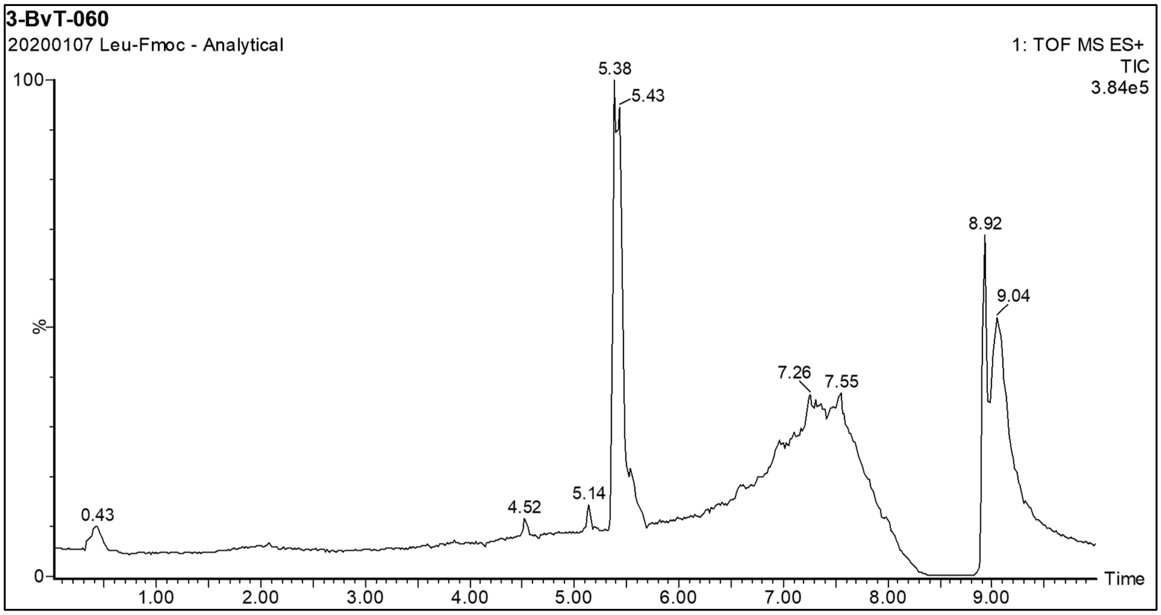

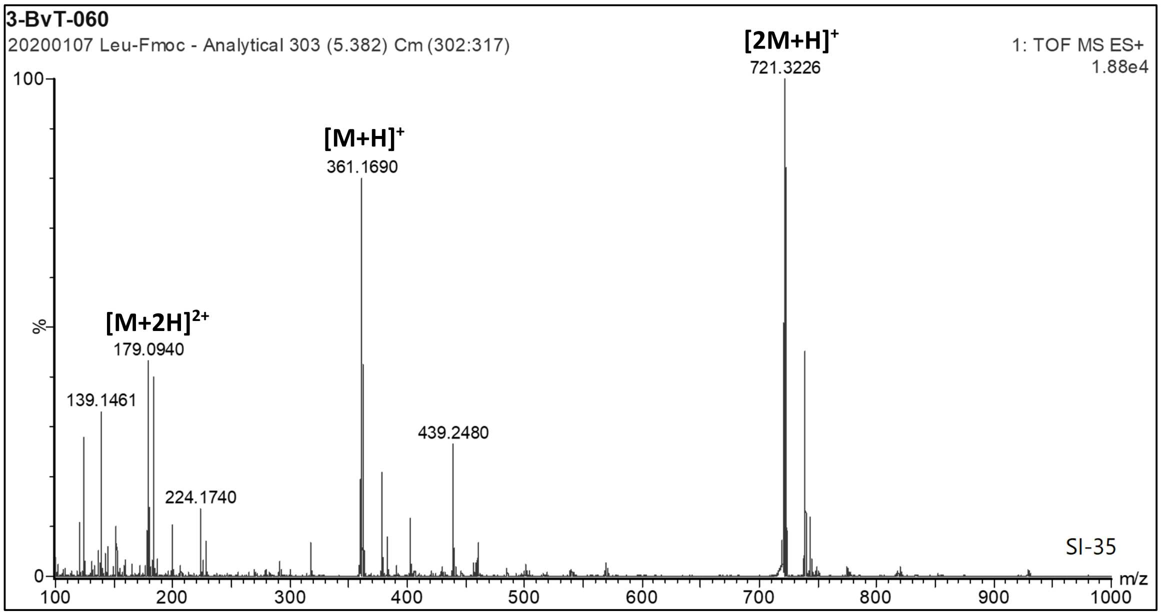

Supplementary Figure 18. Analytical LC-MS data for Fmoc-L-Ile-$^{13}C_6{}^{15}N_1$ (18) ***System 1- Gradient 2***

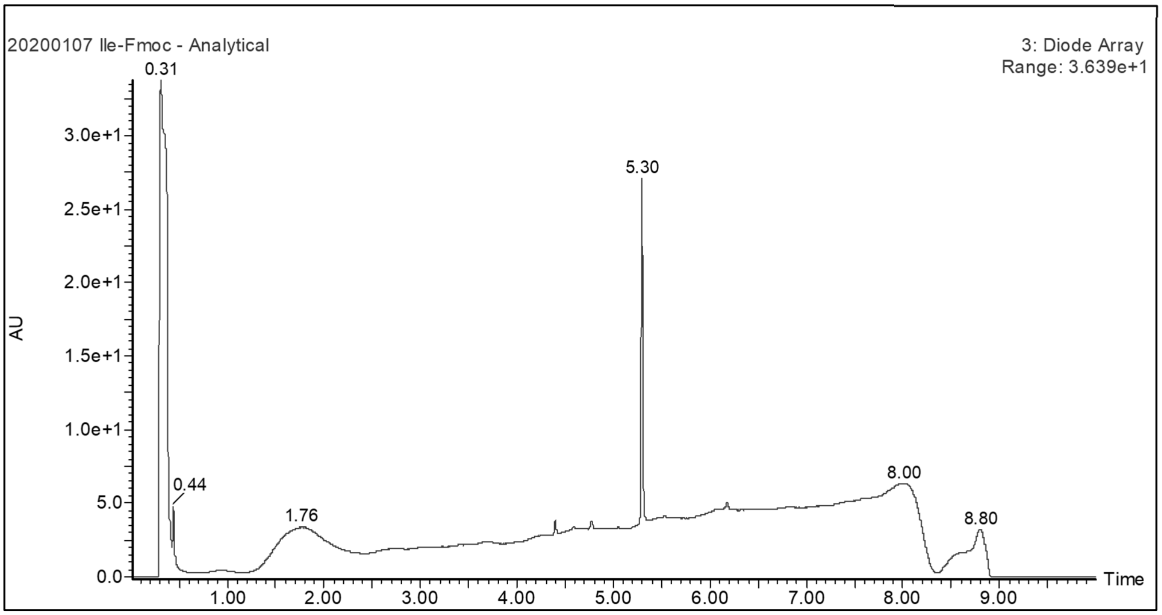

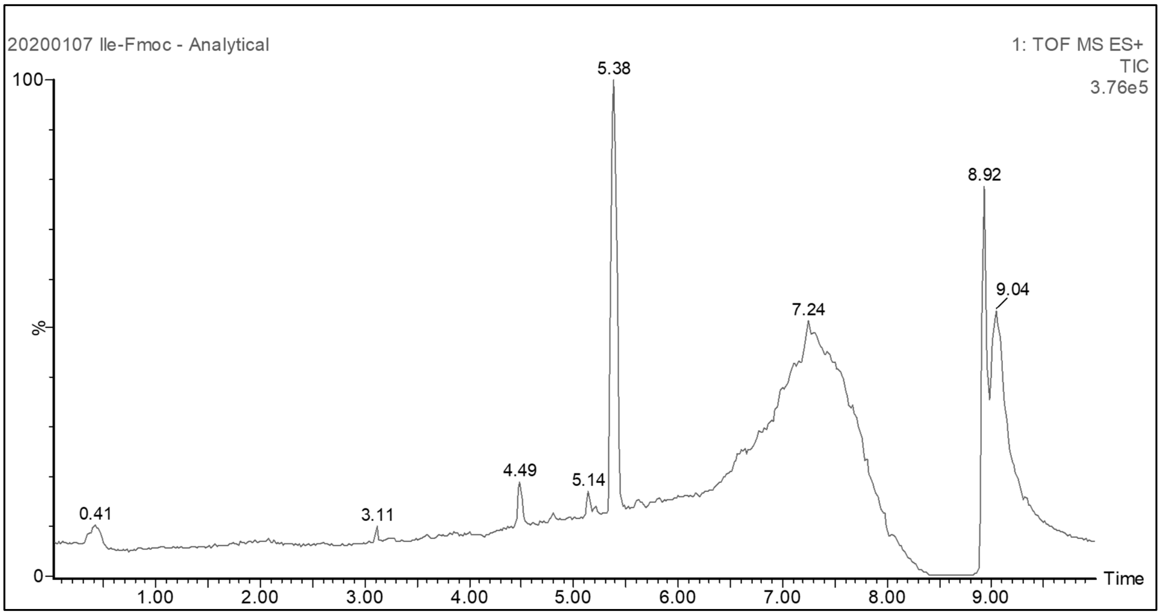

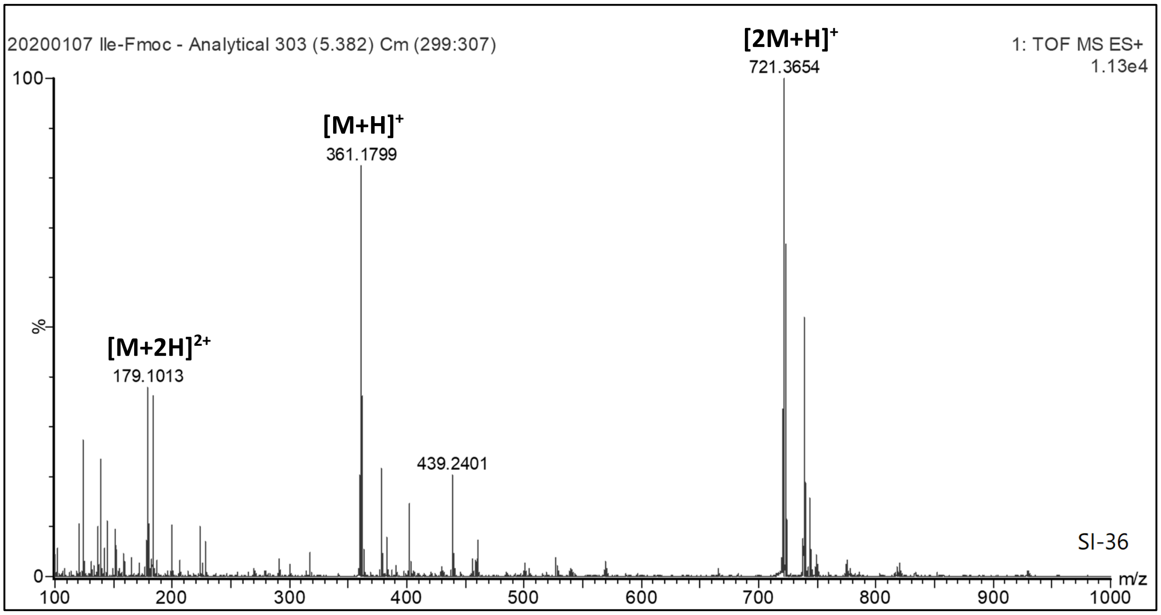

Protein Synthesis

General. Solvents and reagents for peptide synthesis were purchased from various suppliers (listed in Table 7) and were used as received. Linear solid phase peptide synthesis of Ub was performed on resin according to an established method described by our group.[13] LC-MS data processing was performed using Waters MassLynx Mass Spectrometry Software 4.2. Deconvoluted mass was obtained from the electrospray ionization mass spectrum envelope (average isotopes) with Maxent1 function. The calculated mass of Ub (derivatives) was obtained with ChemDraw Professional 20.0 (PerkinElmer Informatics, Inc.) by calculating the molecular weight of the complete structure.

Supplementary Table 7. Building blocks, reagents and solvents for diubiquitin synthesis.*

Compound	Abbreviation	CAS#	Source or reference
Building blocks			
**Fmoc Gly TentaGel® R Trt resin**	Gly-Trt	-	Rapp Polymere GmbH #RA1213
**Fmoc Gly TentaGel® R HMPA resin**	Gly-HMPA	-	Rapp Polymere GmbH #RA1513
**Fmoc Arg(pbf) TentaGel® R HMPA resin**	Arg-HMPA	-	Rapp Polymere GmbH #RA1502
**Fmoc-L-Valine-OH 13C5,15N1**	Fmoc-L-Val $^{13}C_5$ $^{15}N_1$	-	In-house synthesis, *this work*
**Fmoc-L-Leucine-OH 13C6,15N1**	Fmoc-L-Leu $^{13}C_6$ $^{15}N_1$	-	In-house synthesis, *this work*
**Fmoc-L-Isoleucine-OH 13C6,15N1**	Fmoc-L-Ile $^{13}C_6$ $^{15}N_1$	-	In-house synthesis, *this work*
**Fmoc- L-*S*^{t}Bu γ-thiolysine(Boc)-OH**	γ-thioLys	-	In-house synthesis, [Van der Heden van Noort, 2017][14]
**Boc-L-*S*^{t}Bu γ-thionorleucine-OH**	thioNle	-	In-house synthesis, [Xin, 2018][1]
**Methyl-3-(glycylthio)-propionate hydrochloride**	HCl·H-Gly-$S(CH_2)_2CO_2Me$	-	In-house synthesis, *this work*
Chemicals			
**Piperidine**	-	110-89-4	Carlo Erba Reagents #P0663516
**(Benzotriazol-1-yloxy)tripyrrolidino- phosphonium hexafluorophosphate)**	PyBOP	128625-52-5	NovaBiochem #851009
***N,N*-Diisopropylethylamine**	DiPEA	7087-68-5	VWR #84574.290
**Trifluoroacetic acid**	TFA	76-05-1	Biosolve #20233320
**2,2'-(Ethylenedioxy)diethanethiol**	DODt	14970-87-7	SigmaAldrich #465178
**Triisopropylsilane**	iPr_3SiH	6485-79-6	SigmaAldrich #233781
**Phenol**	PhOH	108-95-2	SigmaAldrich #328111
**Di-*tert*-butyldicarbonate**	Boc_2O	24424-99-5	Chem-Impex #00128-100G
**1,1,1,3,3,3-hexafluoroisopropylalcohol**	HFIP	920-66-1	Chem-Impex #00080

**1-Ethyl-3-(3-dimethylaminopropyl)carbodiimide hydrochloride**	EDC	25952-53-8	Combi blocks #SS-7536
**1-hydroxybenzotriazol hydradrate**	HOBt·H_2O	123333-53-9	Biosolve #081233
**Guanidine hydrochloride**	Gnd·HCl	50-01-1	Sigma Aldrich #50950-2.5 KG
**Sodium phosphate dibasic dihydrate**	Na_2HPO_4	10028-24-7	Fluka #71645-1 KG
**Tris(2-carboxyethyl)phosphine hydrochloride**	TCEP·HCl	51805-45-9	Combi blocks #OR-5119
**4-Mercaptophenylacetic acid**	MPAA	39161-84-7	Chem-Impex #28402
**Glutathione (reduced)**	GSH	70-18-8	Chem-Impex #00159
**2,2'-Azobis[2-(2-imidazolin-2-yl)propane] dihydrochloride**	VA-044	2997-92-4	Combi blocks #QC-7454
**Propargylamine**	PA	2450-71-7	SigmaAldrich #P50900
**Triethylamine**	TEA	121-44-8	SigmaAldrich #471283
**Urea**		57-13-6	Bio-Connect, #SC-29114B
**Copper(II) sulfate pentahydrate**	$CuSO_4$·$5H_2O$	7758-99-8	SigmaAldrich #61245
**Sodiumascorbate**		134-03-2	ChemImpex #01436
**2-amino-2-(hydroxymethyl)propaan-1,3-diol**	TRIS	77-86-1	Biosolve #20097789
**Sodium Chloride**	NaCl	7647-14-5	Sigma Aldrich #S9625
Solvents			
**Dichloromethane (AR)**	DCM	75-09-2	VWR #23 366 327
***N*-Methyl-pyrrolidone (PEPTIDE)**	NMP	872-50-4	VWR #84 572 320
**Diethyl ether (AR)**	Et_2O	60-29-7	Biosolve #5280501
***n*-Pentane**	Pentane	109-66-0	Biosolve #16050502
**Dimethyl sulfoxide (AR)**	DMSO(AR)	67-68-5	Biosolve #4470501
**Acetonitrile (ULC-MS)**	CH_3CN (ULC-MS)	75-05-8	Biosolve #1204102
**Formic Acid (ULC-MS)**	FA (ULC-MS)	64-18-6	Biosolve #6914143
**Acetonitrile (HPLC-R)**	CH_3CN (AR)	75-05-8	VWR #83 639 320
**Formic Acid (AR)**	FA (AR)	64-18-6	Fisher Scientific #147932500
**Methanol**	MeOH		VWR #20847307
**1,2-Dichloroethane**	DCE	107-06-2	Biosolve #4050602

* Peptide building blocks are listed in Supplementary Table 8 (SI-58).

Solid Phase Peptide Synthesis (SPPS)

SPPS was performed on a Syro II Multisyntech Automated Peptide synthesizer (SYRO robot; Part Nr: S002PS002; MultiSyntech GmbH, Germany) under inert gas (N_2) application, using standard 9-fluorenylmethoxycarbonyl (Fmoc) based solid phase peptide chemistry on a 10 or 25 µmol scale. A fourfold excess of amino acids relative to pre-loaded Fmoc amino acid trityl resin or HMPA resin (between 0.17 and 0.20 mmol/g, Rapp Polymere, Germany) was used. Ubiquitin variants on resin were prepared by linear SPPS as described previously[13]. Some optimizations were made in the synthesis protocol. Optimization of the procedure led to discarding of the capping step. Optimization also led to the equalizing of all cycles (equivalents reagents, time etc.) except for the coupling cycles

of isotope-labeled amino acids. Briefly, Fmoc-glycine-loaded TentaGel® R trityl resin (Rapp Polymere, Germany, #RA1213), Fmoc-glycine-loaded TentaGel® R HMPA resin (Rapp Polymere, Germany, #RA1513) or Fmoc-Arg(Pbf)-loaded TentaGel® R HMPA resin (Rapp Polymere, Germany, #RA1502) was washed with DCM (1x 5 mL) and swelled with NMP (1x 1250 µL) for 5 minutes prior to further modifications. Fmoc-protecting groups were removed by incubating three times with 20% piperidine/NMP (v/v) for 2, 2 and 5 minutes. Resin was washed with NMP (5x 1100 µL). Fmoc-protected amino acids to-be-coupled (4 eq.) were preactivated with PyBOP (4 eq.) and DIPEA (8 eq.) in NMP. Deprotected resin was incubated twice for 25 minutes with the preactivated mixture, washed with NMP (3x 1100 µL) after the second coupling step and Fmoc removal was performed as described above. This procedure was repeated for each amino acid coupling cycle, with a total of 69, 68 or 67 cycles. Coupling cycles with isotope-labelled amino acids were extended to two times 60 minutes and only three equivalents of Fmoc-protected amino acid was used, PyBOP (4eq.) and DIPEA (8eq.) stayed unchanged. Details on acid-labile side chain protecting groups (PG) and coupling of Fmoc-protected (di)peptide building blocks are provided in Supplementary Table 7 (SI-56) and Supplementary Table 8 (SI-58).

Supplementary Table 8. Fmoc protected L-amino acids and dipeptides

AA	Reagent	CAS#	Cat# *
**A**	Fmoc-L-Ala-OH	35661-39-3	852003
**R**	Fmoc-L-Arg(Pbf)-OH	154445-77-9	852067
**N**	Fmoc-L-Asn(Trt)-OH	132388-59-1	852044
**D**	Fmoc-L-Asp(OtBu)-OH	71989-14-5	852005
**Q**	Fmoc-L-Gln(Trt)-OH	132327-80-1	852045
**E**	Fmoc-L-Glu(OtBu)-OH	71989-18-9	852009
**G**	Fmoc-Gly-OH	29022-11-5	852001
**H**	Fmoc-L-His(Trt)-OH	109425-51-6	852032
**I**	Fmoc-L-Ile-OH	71989-23-6	852010
**L**	Fmoc-L-Leu-OH	35661-60-0	852011
**K**	Fmoc-L-Lys(Boc)-OH	71989-26-9	852012
**Nle**	Fmoc-L-Nle-OH	77284-32-3	852014
**F**	Fmoc-L-Phe-OH	35661-40-6	852016
**P**	Fmoc-L-Pro-OH	71989-31-6	852017
**S**	Fmoc-L-Ser(tBu)-OH	71989-33-8	852019
**T**	Fmoc-L-Thr(tBu)-OH	71989-35-0	852000
**Y**	Fmoc-L-Tyr(tBu)-OH	71989-38-3	852020
**V**	Fmoc-L-Val-OH	68858-20-8	852021
**AG**	Fmoc-L-Ala-(Dmb)Gly-OH	-	852108
**DG**	Fmoc-L-Asp(OtBu)- (Dmb)Gly-OH	900152-72-9	852115
**IT**	Fmoc-L-Ile-L-Thr($\Psi^{Me,Me}$pro)-OH	957780-52-8	852193
**LS**	Fmoc-L-Leu-L-Ser($\Psi^{Me,Me}$pro)-OH	339531-50-9	852179
**LT**	Fmoc-L-Leu-L-Thr($\Psi^{Me,Me}$pro)-OH	955048-89-2	852184
**ST**	Fmoc-L-Ser(tBu)-L-Thr($\Psi^{Me,Me}$pro)-OH	-	852192

* Brand: Novabiochem. Supplier: Merck

General procedure for trial cleavage

A small amount of resin was parted from the reaction mixture and washed with DCM and Et_2O. The resin was air-dried, and incubated with 'fast' trial cleavage mix (TFA/H_2O/DODT/*i*Pr_3SiH; 92.5/2.5/2.5/2.5; v/v/v/v; 100 µL) and shaken for 30 minutes at 37 °C. Samples were transferred to a filter tip and filtered. The reaction mixture (filtrate) was collected in cold Et_2O/*n*-pentane (3/1; v/v; 1.5 mL) to precipitate the product. The suspension was centrifuged and the supernatant was decanted. The precipitate was resuspended twice in cold Et_2O, centrifuged and Et_2O was decanted. The remaining Et_2O was removed by submitting to a gentle air flow. The solid material was dissolved in DMSO (50 µL), the DMSO solution (2 µL) was diluted in 0.1% aqueous formic acid (80 µL) and reaction progress was analysed by LC-MS. *System 1 – Gradient 1*.

LC-MS analysis of trial cleavages, crude reaction mixtures and purification fractions

LC-MS analysis of crude reaction mixtures and purification fractions were performed on a Waters ACQUITY UPLC H-class+ System equipped with Waters ACQUITY Quaternary Solvent Manager (QSM), Waters ACQUITY FTN-H AutoSampler and Waters ACQUITY UPLC Photodiode Array (PDA) eλ Detector (λ = 210-800 nm), Waters ACQUITY UPLC Protein BEH C4 Column (300 Å, 1.7 µm, 2.1 x 50 mm) and LCT Premier Orthogonal Acceleration Time of Flight Mass Spectrometer (*m/z* = 100-1600) in ES+ mode (System 1). Samples were run with a 1.6 minute gradient (run time 3 min) using three mobile phases; 100% H_2O, 100% CH_3CN and 50% H_2O + 50% CH_3CN + 2.5% FA (flow rate = 0.5 mL/min).

LC-MS - System 1 - Gradient 1:

**Time** (min)	**100% H_2O** (%)	**100% CH_3CN** (%)	**50% H_2O + 50% CH_3CN + 2.5% FA**(%)
**0.00**	94.0	2.0	4.0
**0.20**	94.0	2.0	4.0
**1.80**	0.0	96.0	4.0
**2.15**	0.0	96.0	4.0
**2.20**	94.0	2.0	4.0
**3.00**	94.0	2.0	4.0

Pure products were run with a 7 minute gradient (run time 10 min) using three mobile phases: 100% H_2O, 100% CH_3CN and 50% H_2O + 50% CH_3CN + 2.5% FA (flow rate = 0.5 mL/min).

LC-MS – System 1 - Gradient 2:

**Time** (min)	**100% H_2O** (%)	**100% CH_3CN** (%)	**50% H_2O + 50% CH_3CN + 2.5% FA**(%)
**0.00**	94.0	2.0	4.0
**0.50**	94.0	2.0	4.0
**7.50**	0.0	96.0	4.0
**8.00**	0.0	96.0	4.0
**8.10**	94.0	2.0	4.0
**10.00**	94.0	2.0	4.0

LC-MS analysis of purified mono- and diubiquitins

LC-MS analysis of purified mono- and diubiquitins and the described assay were performed on a Waters Acquitiy H-Class UPLC system equipped with a Waters ACQUITY Quaternary Solvent Manager (QSM) and Waters ACQUITY FTN AutoSampler. Separation was achieved on a Waters Acquity UPLC Protein BEH C4 column, 300Å, 1,7 uM (2.1 x 50 mm); flow rate = 0.6 mL/min , runtime = 4.55 min,

column T = 60°C using 2 mobile phases: A = 0,1% formic acid in water and B = 0,1% formic acid in CH_3CN. Mono- and diubiquitins were separated at baseline level and eluted using a shallow gradient focused from 26% → 30% B over 1 minute. The products were analysed by intact MS analysis (MS1) and masses were detected in a range from 550-2000 Da from 2.51 - 4.50 min and were recorded on a Waters XEVO-G2 XS Q-Tof mass spectrometer equipped with an electrospray ion source in positive mode (Capillary Voltage: 0.5 kV, desolvation gas flow: 900 L/h, desolvation gas temperature: 500°C, source temperature: 130 °C, probe angle: 9.5) with a resolution of R = 22,000 (System 2).

LC-MS – System 2 - Gradient 1: diubiquitin gradient

**Time** (min)	**Flowrate** (mL/min)	**100% H_2O** (%) **+0.1% FA**	**100% CH_3CN** (%) **+0.1% FA**
**0.00**	0.6	98.0	2.0
**0.20**	0.6	98.0	2.0
**0.70**	1.0	98.0	2.0
**1.80**	1.0	98.0	2.0
**2.30**	0.6	98.0	2.0
**2.50**	0.6	74.0	26.0
**2.83**	0.6	74.0	26.0
**3.83**	0.6	70.0	30.0
**3.90**	0.6	0.0	100.0
**4.30**	0.6	0.0	100.0
**4.35**	0.6	98.0	2.0
**4.55**	0.6	98.0	2.0

In between assay runs, the column was washed using a run with the same gradient without the column flush with a 1 minute gradient focused from 26% → 30% B (run time 2.20 min) using two mobile phases: 100% H_2O + 0.1% FA and 100% CH_3CN + 0.1% FA (flow rate = 0.6 mL/min) and masses were detected in a range from 550-2000 Da during the entire run time.

LC-MS – System 2 - Gradient 2: diubiquitin wash run

**Time** (min)	**Flowrate** (mL/min)	**100% H_2O** (%) **+0.1% FA**	**100% CH_3CN** (%) **+0.1% FA**
**0.00**	0.6	98.0	2.0
**0.05**	0.6	74.0	26.0
**0.33**	0.6	74.0	26.0
**1.33**	0.6	70.0	30.0
**1.40**	0.6	0.0	100.0
**1.80**	0.6	0.0	100.0
**1.85**	0.6	98	2.0
**2.20**	0.6	98	2.0

Pure products were run with a 7 minute gradient (run time 10 min) using three mobile phases two mobile phases: 100% H_2O + 0.1% FA and 100% CH_3CN + 0.1% FA (flow rate = 0.5 mL/min).

LC-MS – System 2 - Gradient 3: Analytical run

**Time** (min)	**Flowrate** (mL/min)	**100% H_2O** (%) **+0.1% FA**	**100% CH_3CN** (%) **+0.1% FA**
**0.00**	0.5	96.0	4.0
**0.50**	0.5	96.0	4.0
**7.50**	0.5	2.0	98.0
**8.00**	0.5	2.0	98.0
**8.10**	0.5	96.0	4.0
**10.00**	0.5	96.0	4.0

RP-HPLC purification

System 1. RP-HPLC purifications (max. 20 mL/run) were performed on a Waters HPLC equipped with a Waters 2489 UV/Vis detector, Waters fraction collector III and Waters XBridge BEH C18 OBD Prep Column (130 Å, 5 µm, 30 × 150 mm). Samples were run with a 13 or 23 minutes gradient detailed below (run time 25 or 35 minutes) at a flowrate = 37.5 mL/min. Mobile phase: A = H_2O, B = CH_3CN and C = 1% TFA in H_2O. Fraction collection was triggered by UV intensity (λ = 210 nm).

RP-HPLC – System 1 - Gradient 1:

**Time (min)**	**H_2O (%)**	**CH_3CN (%)**	**1% TFA in H_2O (%)**
**0.00**	90.0	5.0	5.0
**5.00**	90.0	5.0	5.0
**7.00**	75.0	20.0	5.0
**18.00**	50.0	45.0	5.0
**18.50**	0.0	95.0	5.0
**21.50**	0.0	95.0	5.0
**21.60**	90.0	5.0	5.0
**25.00**	90.0	5.0	5.0

RP-HPLC – System 1 - Gradient 2 (optimized):

**Time (min)**	**H_2O (%)**	**CH_3CN (%)**	**1% TFA in H_2O (%)**
**0.00**	90.0	5.0	5.0
**6.00**	90.0	5.0	5.0
**7.00**	85.0	10.0	5.0
**10.00**	70.0	25.0	5.0
**22.00**	60.0	35.0	5.0
**30.00**	25.0	70.0	5.0
**30.10**	0.0	95.0	5.0
**32.00**	0.0	95.0	5.0
**32.10**	90.0	5.0	5.0
**35.00**	90.0	5.0	5.0

System 2. RP-HPLC purifications (max. 5 mL/run) were performed on a Shimadzu LC-20AT HPLC system equipped with a Shimadzu SPD-20A UV/Vis detector, a Shimadzu FRC-10A fraction collector and a

Waters XBridge BEH C18 OBD Prep Column (130 Å, 5 µm, 10 × 150 mm) was used. Samples were run with a 15 or 23 minute gradient detailed below (run time 25 or 35 minutes) at a flowrate = 4.00 or 6.50 mL/min. Mobile phase: A = 0.05% TFA in H_2O and B = 0.05% TFA in CH_3CN. T = 40 °C.

RP-HPLC – System 2 - Gradient 1:

Time (min)	0.05% TFA in H_2O (%)	0.05% TFA in CH_3CN (%)	Flow rate (mL/min)
**0.00**	95.0	5.0	4.00
**6.00**	95.0	5.0	4.00
**7.00**	90.0	10.0	6.50
**10.00**	75.0	25.0	6.50
**22.00**	50.0	50.0	6.50
**22.10**	5.0	95.0	6.50
**24.00**	5.0	95.0	6.50
**24.10**	95.0	5.0	6.50
**25.00**	95.0	5.0	6.50

RP-HPLC – System 2 - Gradient 2:

Time (min)	0.05% TFA in H_2O (%)	0.05% TFA in CH_3CN (%)	Flow rate (mL/min)
**0.00**	95.0	5.0	4.00
**6.00**	95.0	5.0	4.00
**7.00**	90.0	10.0	6.50
**10.00**	75.0	25.0	6.50
**22.00**	65.0	35.0	6.50
**30.00**	30.0	70.0	6.50
**30.10**	5.0	95.0	6.50
**32.00**	5.0	95.0	6.50
**32.10**	95.0	5.0	6.50
**35.00**	95.0	5.0	6.50

Synthesis of Ub(1-76, Nle_1, γ-thioLysxx, xx V*, xx L*, xx I*) (**1 a-g**)

Step 1. SPPS

See SPPS procedure with extended coupling times.

Sequences neutron-encoded Ub1-76^{Met1Nle} (Kxx = γ-thioLys)

See table Supplementary Table 1 (SI-38).

69 cycles on Fmoc-Gly-loaded TentaGel® R HMPA resin (Rapp Polymere, Germany, #RA RA1513) on 10 µmol scale. To check the quality of the SPPS product a trial cleavage was performed.

Step 2. Global deprotection

Global deprotection was performed as described previously.[15] The resin-bound polypeptide Ub(1-76, Nle_1, γ-thioLysxx, xx V*, xx L*, xx I*)(PG) **6a-g** was deprotected and detached from the resin by treatment with TFA/H_2O/Phenol/iPr_3SiH (90.5/5/2.5/2; v/v/v/v; 7.5 mL) for 2.5-3.5 hours at room temperature under gentle shaking. The reaction mixture was filtered directly into ice-cold Et_2O/*n*-pentane (3/1; v/v; 35 mL) and the resin was washed with TFA (2x 4mL). The mixture of Et_2O/*n*-pentane and filtrate was centrifuged (1500 rpm, 5 min, 4 °C) and the supernatant was decanted. The pellet was washed three times by resuspension in Et_2O (20 mL), spinning down by centrifuge (1500 rpm, 5 min, 4 °C) and removal of the supernatant. The pellet was dissolved in H_2O/CH_3CN/formic acid (65/35/10; v/v/v; 15 mL) and lyophilized. The protein was subsequently purified using RP-HPLC.

RP-HPLC purification

The crude monoubiquitin was properly dissolved in a minimal amount of DMSO (max. 10 vol% of the final volume) while heated carefully. The DMSO was added dropwise into H_2O (10 to 20 mL). The pH was checked and should be below 7. The mixture was centrifuged (5 min @3800 rpm). The supernatant was filtered and purified by RP-HPLC.

See *RP-HPLC – System 2 – Gradient 1.*

Pure fractions (checked by LC-MS) were pooled and lyophilized to obtain the product as a white powder.

Ub(1-76, Nle_1, γ-thioLysxx, xx V*, xx L*, xx I*) (**1 a-g**)

The products were obtained as white solids. LC-MS analysis using *System 2 - Gradient 3*.

Yields:

Ub(1-76, Nle_1, γ-thioLys6, 1x V*, 1x L*) **1a** = 34.36 mg, 3.96 µmol, 39.6%. LC-MS: R_t 3.18 min: MS ES+ (amu) calculated: 8679,96 Da[M]; found: 8680 Da.

Ub(1-76, Nle_1, γ-thioLys11, 3x V*, 1x I*) **1b** = 21.51 mg, 2.47 µmol, 16.5%. LC-MS: R_t 3.18 min: MS ES+ (amu) calculated: 8691.87 Da[M]; found 8692 Da.

Ub(1-76, Nle_1, γ-thioLys27, 3x V*, 2x L*, 1x I*) **1c** = 17.99 mg, 2.07 µmol, 13.8%. LC-MS: R_t 3.28 min: MS ES+ (amu) calculated: 8705.77 Da[M]; found 8706 Da.

Ub(1-76, Nle_1, γ-thioLys29, 3x V*, 2x L*, 3x I*) **1d** = 22.28 mg, 2.55 µmol, 17.0%. LC-MS: R_t 3.25 min: MS ES+ (amu) calculated: 8719.66 Da[M]; found 8720 Da.

Ub(1-76, Nle_1, γ-thioLys33, 3x V*, 4x L*, 3x I*) **1e** = 23.31 mg, 2.67 µmol, 17.8%. LC-MS: R_t 3.22 min: MS ES+ (amu) calculated: 8766.56 Da[M]; found 8734 Da.

Ub(1-76, Nle_1, γ-thioLys48, 3x V*, 4x L*, 5x I*) **1f** = 23.5 mg, 3.53 µmol, 23.5%. LC-MS: R_t 3.19 min: MS ES+ (amu) calculated: 8747.45 Da[M]; found 8747 Da.

Ub(1-76, Nle_1, γ-thioLys63, 3x V*, 6x L*, 5x I*) **1g** = 27.90 mg, 3.18 µmol, 21.2%. LC-MS: R_t 3.22 min: MS ES+ (amu) calculated: 8761.34 Da[M]; found 8761 Da.

Synthesis of Ub(1-76, thioNle$_1$, 4x V*, 6x L*, 6x I*) (**2**)

Step 1. SPPS

See SPPS procedure with extended coupling times.

Sequences neutron-encoded Ub2-76

See Supplementary table 1 (SI-38).

68 cycles on Fmoc-Gly-loaded TentaGel® R HMPA resin (Rapp Polymere, Germany, #RA RA1513) on 10 µmol scale. To check the quality of the SPPS product a trial cleavage was performed.

Step 2. ThioNle coupling

PyBOP (4 eq., 20.9 mg, 40.12 µmol) was dissolved in NMP (100 µL). ThioNle (1.4 eq., ~14 mg, 39.82 µmol) was dissolved in NMP (200 µL). Both solutions were added to resin-bound Ub (2-76, 4x V*, 6x L*, 6x I*) **8**. DiPEA (8 eq., 10.3 mg, 79.79 µmol, 13.9 µL) was dissolved in NMP (60 µL) and this solution was also added to the resin. The reaction mixture was shaken overnight. To check the reaction progress by LC-MS a trial cleavage was performed. The reaction solution was filtered from the resin and the resin was washed three times with DCM and MeOH alternately, three times with DCM and Et_2O alternately and three times with Et_2O. The resin was either resubmitted to coupling conditions until reaction completion was achieved or submitted to global deprotection conditions.

Step 3. Global deprotection

The resin-bound polypeptide Ub(1-76, ThioNle1, 4x V*, 6x L*, 6x I*)(PG) **10** was deprotected and detached from the resin by treatment with TFA/H_2O/Phenol/iPr3SiH (90.5/5/2.5/2; v/v/v/v; 2 mL) for 2.5-3.5 hours at room temperature under gentle shaking. The reaction mixture was filtered directly into ice-cold Et_2O/n-pentane (3/1; v/v; 15 mL) and the resin was washed with TFA (2x 2 mL).

The mixture of Et_2O/*n*-pentane and filtrate was centrifuged (1500 rpm, 5 min, 4 °C) and the supernatant was removed. The pellet was washed with Et_2O (3 x 15 mL), the solution was vortexed, the suspension was centrifuged and the Et_2O was removed. Wash step was repeated twice. The pellet was dissolved in H_2O/CH_3CN/formic acid (65/25/10; v/v/v; 5 mL) and lyophilized. The protein was subsequently purified using RP-HPLC.

RP-HPLC purification

The crude monoubiquitin was properly dissolved in a minimal amount of DMSO (max. 10 vol% of the final volume) under careful heating. The DMSO was added dropwise into H_2O (10 to 20 mL). The pH was checked and should be below 7. The mixture was centrifuged (5 min @3800 rpm). The supernatant was filtered and purified by RP-HPLC.

RP-HPLC – System 2 – Gradient 1.

Pure fractions (checked by LC-MS) were pooled and lyophilized to obtain the product as a white powder.

Ub(1-76, ThioNle1, 4x V*, 6x L*, 6x I*) (**2**)

The product was obtained as white solid. LC-MS analysis using *System 2 - Gradient 3*.

Yield:

Ub(1-76, ThioNle1, 4x V*, 6x L*, 6x I*) **2 =** 5.52 mg, 0.63 µmol, 6.3%. LC-MS: R_t 3.24 min: MS ES+ (amu) calculated: 8774.246 Da[M]; found 8774 Da.

Synthesis of Ub(1-76, Nle_1)-$S(CH_2)_2CO_2Me$ **(3)**

Step 1. SPPS

See SPPS procedure.

Sequence Ub1-75^{Met1Nle}

(Nle)QIFVKTLTG KTITLEVEPS DTIENVKAKI QDKEGIPPDQ QRLIFAGKQL EDGRTLSDYN IQKESTLHLV LRLRG

68 cycles on Fmoc-Gly-loaded TentaGel® R trityl resin (Rapp Polymere, Germany, #RA RA1213) on 25 µmol scale. To check the quality of the SPPS product a trial cleavage was performed.

Step 2. Boc-protection

Resin-bound H-Ub(1-75, Nle_1)(PG) **7** was washed with DCM (3x 5 mL) and NMP (3x 5 mL) alternately and with DCM (3x 5 mL). Boc_2O (4 eq., 21.83 mg, 100 µmol, 23 µL) and DiPEA (15 eq., 48.47 mg, 375 µmol, 65.3 µL) were dissolved in DCM (1 mL). This solution was added to the resin-bound H-Ub(1-75, Nle_1) and the mixture was shaken for 3 hours at room temperature. After 3 hours, the liquid was removed and the resin was washed with NMP (3x 5 mL) and DCM (3x 5 mL) alternately followed by washing with DCM (3x 5 mL) and MeOH (3x 5 mL) alternately.

Step 3. Cleavage from resin

Resin cleavage was performed as described previously.[13] Resin-bound Boc-Ub(1-75, Nle_1)(PG) was cleaved from the trityl resin using a solution of hexafluoroisopropanol (HFIP) in DCM (1/4; v/v; 2.5 mL). The resin was treated twice for 20 minutes at room temperature and the filtrate was collected. The resin was rinsed two times with DCM in between HFIP treatments. All combined filtrates were concentrated under reduced pressure. Residual HFIP was removed by co-evaporation with DCE (3x 12 mL) (to prevent formation of HFIP ester in next steps) and the product lyophilized overnight.

Step 4. Gly-thioester coupling

The protected protein was dissolved in DCM (4 mL) and EDC (3 eq., 14.4 mg, 75 µmol), HOBt (3 eq., 10.1 mg, 75 µmol) and HCl·H-Gly-$S(CH_2)_2CO_2Me$ (3 eq., 16 mg, 75 µmol) were added to the solution. The reaction mixture was stirred for 16 hours. To evaluate the reaction progress by LC-MS, a trial cleavage was performed. The reaction mixture was concentrated under reduced pressure and the residue was either resubmitted to coupling conditions until reaction completion was achieved or submitted to global deprotection conditions.

Step 5. Global deprotection

Global deprotection was performed as described previously.[15] Ub(1-76, Nle_1)(PG)-$S(CH_2)_2CO_2Me$ **9** was treated with a freshly prepared solution of TFA/H_2O/Phenol/iPr_3SiH (90.5/5/2.5/2; v/v/v/v; 5 mL) for 2.5-3.5 hours at room temperature to remove all protecting groups from all amino acid sidechains. The cleavage mixture was added dropwise to an ice-cold Et_2O/*n*-pentane (3/1; v/v; 20 mL) mixture to precipitate the protein. The suspension was centrifuged (1500 rpm, 5 min, 4 °C) and the Et_2O/ *n*-pentane (supernatant) was removed. The pellet was resuspended in Et_2O (20 mL), the suspension was vortexed, centrifuged (1500 rpm, 5 min, 4 °C) and the Et_2O was removed. The wash step was repeated twice. The remaining solvent was removed by a gentle N_2 flow over the pellet. The solid crude material was dissolved in H_2O/CH_3CN/formic acid (65/25/10; v/v/v; 10 mL) and lyophilized. The crude material was subsequently purified using preparative RP-HPLC.

RP-HPLC purification

The crude monoubiquitin was properly dissolved in a minimal amount of DMSO (max. 10 vol% of the final volume) under careful heating. The DMSO solution was added dropwise into H_2O (10 to 20 mL). The pH was checked and should be below 7. The mixture was centrifuged (5 min @3800 rpm). The supernatant was filtered and purified by RP-HPLC.

See *RP-HPLC – System 1 - Gradient 1.*

Pure fractions (>95%, checked by LC-MS) were pooled and lyophilized to obtain the product as a white powder.

Ub(1-76, Nle_1)-$S(CH_2)_2CO_2Me$ (**3**)

The product was obtained as white solid. LC-MS analysis using *System 2 - Gradient 3*.

Yield:
Ub(1-76, Nle_1)-$S(CH_2)_2CO_2Me$ **3** = 57.53 mg, 6.65 µmol, 26.6%. LC-MS: R_t 3.13 min: MS ES+ (amu) calculated: 8649.0 Da[M]; found 8649 Da.

Native Chemical Ligation of Ub(1-76, Nle_1)-$S(CH_2)_2CO_2Me$ (**3**) and Ub(1-76, Nle_1, γ-thioLysxx, xx V*, xx L*, xx I*) (**1a-g**)

Native Chemical Ligation

Ub(1-76, Nle_1, γ-thioLysxx, xx V*, xx L*, xx I*) (**1 a-g**, 1 eq., 10.0 mg, 1.10 µmol) was dissolved in DMSO (50 µL) and added dropwise to aqueous 8 M Gnd·HCl and 0.2 M Na_2HPO_4 pH 7.55 (175 µL). 1 M aqueous TCEP solution pH 7.0 (25 µL) was added. This solution was pre-incubated for 180 min. and the disulfide bond cleavage was monitored by LC-MS analysis (Program 1). Ub(1-76, Nle_1)-$S(CH_2)_2CO_2Me$ (**3**, 1.5 eq., 15.0 mg, 1.73 µmol) was dissolved in DMSO (37.5 µL) and added dropwise to aqueous 8 M Gnd·HCl and 0.2 M Na_2HPO_4 pH 7.55 (130 µL). 1 M aqueous MPAA solution pH 7.0 (137.5 µL) was added. The solution was pre-incubated for 5 minutes. The thioLys **1a-g** and the thioester **3** containing solutions were properly mixed and the pH of the reaction mixture was adjusted to pH 7.33 by the addition of 35 µL 10% Na_2CO_3 in H_2O solution. The reaction mixture was flushed with argon and shaken for 16h at 37 °C. The reaction progress was checked by LC-MS analysis (Program 1). The formed diUb was purified by RP-HPLC.

RP-HPLC purification

To prepare the sample, the reaction mixture was added dropwise to 3.76 mL aqueous buffer containing 6.0 M Gnd·HCl and 0.15 M Na_2HPO_4. This solution was diluted with water to 14 mL. 1 M aqueous TCEP solution pH 7.0 (250 µL) was added. The pH was checked and adjusted below 7 with 1 M aqueous HCl solution. The mixture was centrifuged (5 min @ 3800 rpm), filtered and purified by RP-HPLC.

See *RP-HPLC – System 2 - Gradient 2.*

Pure fractions (>95%, checked by LC-MS) were pooled and lyophilized, dissolved in H_2O/CH_3CN/formic acid (65/25/10; v/v/v; 15 mL) and lyophilized again. The product was obtained as white powder.

Native Chemical Ligation of Ub(1-76, Nle_1)-$S(CH_2)_2CO_2Me$ (**3**) and Ub(1-76, ThioNle1, 4x V*, 6x L*, 6x I*) (**2**)

Native Chemical Ligation

The Native Chemical Ligation reaction to create a linear diUb molecule was described previously.[1] Briefly, Ub(1-76, ThioNle1, 4x V*, 6x L*, 6x I*) (**2**) (5 mg) was dissolved in an aqueous buffer containing 8 M Gnd·HCl and 0.2 M Na_2HPO_4 pH 7.55 (100 µL). 1 M aqueous TCEP solution pH 7.0 (12.5 µL) was added to the solution to reduce the disulfide bond on the ThioNle side chain. This solution was pre-incubated for 1h and disulfide bond reduction was monitored by LC-MS. Ub(1-76, Nle_1)-S-$(CH_2)_2CO_2Me$ (**3**) (1.5 eq., 7.5 mg) was dissolved in 151.9 µL of aqueous 8 M Gnd·HCl and 0.2 M Na_2HPO_4 pH 7.55 and 46.9 µL of 1 M MPAA solution was added. This solution was pre-incubated for 5 minutes. Both solutions were properly mixed and the pH of the reaction mixture was adjust to pH 7.45 by the addition of 22 µL 10% Na_2CO_3 in H_2O solution. The reaction mixture was shaken overnight at 37

°C. The progress of the reaction was checked by LC-MS analysis. The formed diUb was purified by RP-HPLC.

RP-HPLC purification

To prepare the sample, the reaction mixture was added dropwise to 2.5 mL aqueous buffer containing 6 M Gnd·HCl and 0.15 M Na_2HPO_4. This solution was diluted with water to 10 mL. 1 M aqueous TCEP solution pH 7.0 (125 µL) was added. The pH was checked and adjusted below 7. The mixture was centrifuged (5 min @ 3800 rpm), filtered and purified by RP-HPLC on the Shimadzu. Pure fractions (checked by LC-MS) were pooled and lyophilized, dissolved in H_2O/CH_3CN/formic acid (65/25/10; v/v/v; 15 mL) and lyophilized again. The product was obtained as white powder.

See *RP-HPLC – System 2 - Gradient 2.*

Thiol-containing linear diUb (4x V*, 6x L*, 6x I*) (**12**)

The product was obtained as white solid. LC-MS analysis using *System 1 - Gradient 1*.

Yield:

Thiol-containing linear diUb (4x V*, 6x L*, 6x I*) **12** = 3.89 mg, 0.22 µmol, 38.9%. LC-MS: R_t 2.00 min: MS ES+ (amu) calculated: 17215 Da[M]; found 17214 Da.

Desulfurization

Desulfurization

The desulfurization reaction was described previously.[1,15] Briefly, thiol-containing diUb was dissolved in aqueous buffer containing 6 M Gnd·HCl, 0.15 M Na_2HPO_4 and 0.25 M TCEP at pH 7.0 to a concentration of 1 mg/mL protein. Reduced glutathione (GSH) was added to the solution to a concentration of 100 mM. The pH of the solution was adjust to 7.20 by the addition of 400 µL of 10% Na_2CO_3 solution. VA-044 was added to the solution to a final concentration of 75 mM. The reaction mixture was flushed with argon and shaken overnight at 37 °C. The progress of the reaction was checked by LC-MS analysis (Program 1). The desulfurized diUb was purified by RP-HPLC.

RP-HPLC purification

The reaction mixture was diluted with water (same amount as the reaction volume) and 1 M NaOAc/AcOH buffer (40 vol% of the reaction volume). The sample diluted with MQ to 10 mL, the pH was checked and adjusted below 7, the sample was centrifuged (5 min @ 3800 rpm) and filtered, before it was purified by RP-HPLC on the Shimadzu HPLC. Pure fractions (checked by LC-MS) were pooled and lyophilized, dissolved in H_2O/CH_3CN/formic acid (65/25/10; v/v/v; 15 mL) and lyophilized again. The product was obtained as white powder.

See *RP-HPLC – System 2 - Gradient 2.*

Size exclusion

The products were purified by gel filtration using a Biorad NGC Chromotography system on a size exclusion HiLoad® 16/600 Superdex® 75 pg GE healthcare column with a volume bed of 120 mL and 3-70 kDa separation range using a filtered aqueous buffer containing 50 mM TRIS·HCl and 20 mM NaCl at pH 7.55 at a flowrate of 1 mL/min. The sample was prepared by dissolving the product in DMSO (250 µL), dropwise addition of this solution to MilliQ (2450 µL) and dropwise addition of 10x TRIS buffer (300 µL). The mixture was centrifuged for 5 min @3500 rpm. The fractions were analysed by SDS-PAGE analysis and LC-MS and pure fractions were pooled. The products were obtained as colorless solutions containing 50 mM TRIS·HCl and 20 mM NaCl buffer at pH 7.55. LC-MS analysis (*System 2 - Gradient 3*) was done to check the purity. Pure fractions were combined and concentrated using 3 kDa MWCO Amicon Ultra spin filters.

Concentration determination

To determine the concentration of the solutions (and the yield), these solutions were together with a concentration series of monoubiquitin (0.5 µg, 1 µg, 2 µg, 4 µg per lane), resolved by gel electrophoreses, stained with InstantBlue™ Staining and scanned. The concentration of the solution was determined by quantification of the bands using a GE Healthcare Amersham Imager 600 with ImageQuant TL 8.1 GE Healthcare lifesciences software.

Protein	Stock concentration (mg/mL)	Stock concentration (µM)	Amount
**Neutron-encoded K6 diUb (4a)**	0.44	25.75	1.5 mL
**Neutron-encoded K11 diUb (4b)**	0.45	26.32	1.6 mL
**Neutron-encoded K27 diUb (4c)**	0.94	54.92	1.65 mL
**Neutron-encoded K29 diUb (4d)**	0.88	51.38	1.85 mL
**Neutron-encoded K33 diUb (4e)**	0.36	21.00	1.65 mL
**Neutron-encoded K48 diUb (4f)**	0.26	15.15	1.95 mL
**Neutron-encoded K63 diUb (4g)**	0.20	11.65	1.5 mL
**Neutron-encoded M1 diUb (5)**	0.46	26.77	0.55 mL

<u>Isopeptide-linked diUb (xx V*, xx L*, xx I*) (**4a-g**)</u>

The products were obtained as solutions. LC-MS analysis using System 2 – *Gradient 3*.

Yields:

Lys6 linked neutron-encoded diUb (1x V*, 1x L*) **4a** = 0.66 mg, 0.039 µmol, 3.3% (over two steps) . LC-MS: R_t 3.22 min: MS ES+ (amu) calculated: 17089 Da[M]; found 17089 Da.

Lys11 linked neutron-encoded diUb (3x V*, 1x I*) **4b** = 0.72 mg, 0.042 µmol, 3.6% (over two steps). LC-MS: R_t 3.25 min: MS ES+ (amu) calculated: 17100 Da[M]; found 17100 Da.

Lys27 linked neutron-encoded diUb (3x V*, 2x L*, 1x I*) **4c** = 1.55 mg, 0.090 µmol, 7.75% (over two steps). LC-MS: R_t 3.24 min: MS ES+ (amu) calculated: 17115 Da[M]; found 17115 Da.

Lys29 linked neutron-encoded diUb (3x V*, 2x L*, 3x I*) **4d** = 1.63 mg, 0.095 µmol, 8.15% (over two steps). LC-MS: R_t 3.23 min: MS ES+ (amu) calculated: 17129 Da[M]; found 17129 Da.

Lys33 linked neutron-encoded diUb (3x V*, 4x L*, 3x I*) **4e** = 0.59 mg, 0.034 µmol, 2.95% (over two steps). LC-MS: R_t 3.23 min: MS ES+ (amu) calculated: 17143 Da[M]; found 17143 Da.

Lys48 linked neutron-encoded diUb (3x V*, 4x L*, 5x I*) **4f** = 0.51 mg, 0.029 µmol, 2.55% (over two steps). LC-MS: R_t 3.21 min: MS ES+ (amu) calculated: 17157 Da[M]; found 17156 Da.

Lys63 linked neutron-encoded diUb (3x V*, 6x L*, 5x I*) **4g** = 0.3 mg, 0.017 µmol, 1.5% (over two steps). LC-MS: R_t 3.22 min: MS ES+ (amu) calculated: 17171 Da[M]; found 17171 Da.

<u>Linear diUb (4x V*, 6x L*, 6x I*) (**5**)</u>

The product was obtained as a solution. LC-MS analysis using System 2 – *Gradient 3*.

Yield:

Linear neutron-encoded diUb (4x V*, 6x L*, 6x I*) **5** = 0.25 mg, 0.15 µmol, 11.8%. LC-MS: R_t 3.32 min: MS ES+ (amu) calculated: 17183 Da[M]; found 17184 Da.

Extra attention was given to obtaining a good purity of the diUb molecules since impurities will have a detrimental effect on the MS-based quantification during the envisioned assay. Based on the LC-MS data, the purity of all linkages was sufficient for the designed assay.

<u>Synthesis of Ub(1-74, Nle_1)</u>

Step 1. SPPS

See SPPS procedure.

Sequence Ub1-74^{Met1Nle}

(Nle)QIFVKTLTG KTITLEVEPS DTIENVKAKI QDKEGIPPDQ QRLIFAGKQL EDGRTLSDYN IQKESTLHLV LRLR

67 cycles on Fmoc-Arg(Pbf)-loaded TentaGel® R HMPA resin (Rapp Polymere, Germany, #RA1502)

25 µmol scale – ~12.5 µmol used for next step. To check the quality of the SPPS product a trial cleavage was performed.

Step 2. Global deprotection

Global deprotection was performed as described previously.[15] The resin-bound polypeptide Ub(1-74, Nle_1) was deprotected and detached from the resin by treatment with a freshly prepared solution of TFA/H_2O/Phenol/iPr$_3$SiH (90.5/5/2.5/2; v/v/v/v; 2.5 mL) for 2.5-3.5 hours at room temperature to remove all protecting groups from all amino acid sidechains. The reaction mixture was filtered directly into ice-cold Et_2O/*n*-pentane (3/1; v/v; 10 mL) and the resin was washed with TFA (2x 4mL). The mixture of Et_2O/*n*-pentane and filtrate was centrifuged (1500 rpm, 5 min, 4 °C) and the Et_2O/ *n*-pentane (supernatant) was removed. The pellet was resuspended in Et_2O (12 mL), the suspension was vortexed, centrifuged (1500 rpm, 5 min, 4 °C) and the Et_2O was removed. The wash step was repeated twice. The remaining solvent was removed by a N_2 flow over the pellet. The solid crude material was dissolved in H_2O/CH_3CN/formic acid (65/25/10; v/v/v; 15 mL) and lyophilized. The crude material was subsequently purified using preparative RP-HPLC (*RP-HPLC – System 1 – Gradient 1).* Pure fractions (>95%, checked by LC-MS) were pooled and lyophilized to obtain the product as a white powder.

<u>Ub(1-74, Nle_1)</u>

The product was obtained as white solid. LC-MS analysis using Program 2.

Yield:

Ub(1-74, Nle_1) = 32.85 mg, 3.89 µmol, 31.1%. LC-MS: R_t 3.13 min: MS ES+ (amu) calculated: 8432.7 Da[M]; found 8433 Da.

The products was purified by gel filtration using a Biorad NGC Chromotography system on a size exclusion HiLoad® 16/600 Superdex® 75 pg GE healthcare column. See **Size Exclusion** (above). The product was obtained as colorless solution containing 50 mM TRIS·HCl and 20 mM NaCl buffer at pH 7.55. LC-MS analysis (LC-MS – System 2 – Gradient 2) was done to check the purity. Pure fractions were combined and concentrated using a 3 kDa MWCO Amicon Ultra spin filters. Yielding a stock concentration of 2.5 mg/mL or 296.5 µM.

<u>Synthesis of Ub(1-75, Nle_1)-PA (**21**)</u>

Step 1. SPPS

See SPPS procedure.

Sequence Ub1-75^{Met1Nle}

(Nle)QIFVKTLTG KTITLEVEPS DTIENVKAKI QDKEGIPPDQ QRLIFAGKQL EDGRTLSDYN IQKESTLHLV LRLRG

68 cycles on Fmoc-Gly-loaded TentaGel® R trityl resin (Rapp Polymere, Germany, #RA RA1213)

25 µmol scale. To check the quality of the SPPS product a trial cleavage was performed.

Step 2,3 and 4. Cleavage from resin, PA coupling and Global deprotection

Ub(1-75, Nle_1)-PA (**21**) was prepared as described previously.[16] Briefly, after SPPS and release from the resin using HFIP/DCM (1/4; v/v) the protected polypeptide Ub(1-75, Nle_1) (25 µmol) was dissolved in DCM (1 mL/ 5 µmol), and PyBOP (5 eq., 125 µmol), triethylamine (5 eq., 125 µmol) and propargylamine (10 eq., 250 µmol) were added to the solution. The reaction was stirred for 16 hours at RT. The reaction mixture was concentrated and deprotected using TFA/H_2O/Phenol/iPr_3SiH (90.5/5/2.5/2; v/v/v/v) for 2.5 hours. The crude polypeptide was collected after precipitation from ice-cold Et_2O/*n*-pentane (3/1; v/v), centrifugation (1500 rpm, 5 min, 4 °C) and the Et_2O/ *n*-pentane (supernatant) was removed. The pellet was resuspended in Et_2O (20 mL), the suspension was vortexed, centrifuged (1500 rpm, 5 min, 4 °C) and the Et_2O was removed. The crude polypeptide was purified using preparative RP-HPLC (Method A; Gradient 1). Pure fractions (> 95%, checked by LC-MS) were pooled and lyophilized to obtain the product as a white powder.

Synthesis of Ub(1-75, Nle_1, L-azido-$ornithine_{48}$) (**22**)

Step 1. SPPS

See SPPS procedure.

Sequence Ub1-75 ^{Met1Nle} (K48 = L-azido-ornithine)

(Nle)QIFVKTLTG KTITLEVEPS DTIENVKAKI QDKEGIPPDQ QRLIFAG(L-azido-ornithine)QL EDGRTLSDYN IQKESTLHLV LRLRG

Step 2. Global deprotection

Global deprotection was performed as described previously.[15] The resin-bound polypeptide Ub(1-75, Nle_1, L-azido-$ornithine_{48}$) was deprotected and detached from the resin by treatment with a freshly prepared solution of TFA/H_2O/Phenol/iPr_3SiH (90.5/5/2.5/2; v/v/v/v; 5 mL) for 2.5-3.5 hours at room temperature to remove all protecting groups from all amino acid sidechains. The reaction mixture was filtered directly into ice-cold Et_2O/*n*-pentane (3/1; v/v; 35 mL) and the resin was washed with TFA (2x 4mL). The mixture of Et_2O/*n*-pentane and filtrate was centrifuged (1500 rpm, 5 min, 4 °C) and the Et_2O/ *n*-pentane (supernatant) was removed. The pellet was resuspended in Et_2O (20 mL), the suspension was vortexed, centrifuged (1500 rpm, 5 min, 4 °C) and the Et_2O was removed. The wash step was repeated twice. The remaining solvent was removed by a gentle N_2 flow over the pellet. The solid crude material was dissolved in H_2O/CH_3CN/formic acid (65/25/10; v/v/v; 15 mL) and lyophilized. The crude material was subsequently purified using preparative RP-HPLC (*RP-HPLC – System 2 – Gradient 1*). Pure fractions (checked by LC-MS) were pooled and lyophilized to obtain the product as a white powder.

Synthesis of non-hydrolysable clicked Lys48 diubiquitin (**23**)

Copper-catalysed alkyne-azide cycloaddition (CuAAC) – "Click reaction"

The click reaction was performed as described previously.[17] Briefly, the CuAAC reaction was performed under denaturing conditions in 8 M Urea, 100 mM phosphate buffer pH 7. Ub(1-75, Nle_1)-PA (**21**) (11.5 mg) was dissolved in warm DMSO (100 µL) and Ub(1-75, Nle_1, L-azido-$ornithine_{48}$) (**22**) (10.25 mg) was dissolved in warm DMSO (100 µL). Both DMSO solutions were added to an aqueous buffer containing 8 M Urea and 100 mM phosphate, pH 7 (2 mL). To the solution 210 µL of catalyst solution containing 25 mg/mL $CuSO_4 \cdot 5H_2O$ in H_2O, 120 mg/mL sodium ascorbate in H_2O and 52 mg/mL TBTA-analogue[3] in CH_3CN (1/1/1; v/v/v) was added. The reaction was gently shaken at room temperature. Extra catalyst solution was added after 1h (90 µL) and fresh catalyst solution was added after 16h (210 µL). After reaction was finished, as judged by LC-MS (~ 24 hour), the reaction was quenched by the addition of 34 µL of 0.5 M EDTA (1 eq. compared to $CuSO_4 \cdot 5H_2O$), pH 7.0. The crude material was subsequently purified using preparative RP-HPLC (*RP-HPLC – System 2 – Gradient 2*). Pure fractions (checked by LC-MS) were pooled and lyophilized to obtain the product as a white powder.

The products were purified by gel filtration using a Biorad NGC Chromotography system on a size exclusion HiLoad® 16/600 Superdex® 75 pg GE healthcare column. See **Size Exclusion** (above). The product was obtained as colorless solution containing 50 mM TRIS·HCl and 20 mM NaCl buffer at pH 7.55. LC-MS analysis (LC-MS – System 2 – Gradient 2) was done to check the purity. Pure fractions were combined and concentrated using a 3 kDa MWCO Amicon Ultra spin filters. Yielding a stock concentration of 1.52 mg/mL or 89.25 µM.

Supplementary References

1. Xin, B. T., Van Tol, B. D. M., Ovaa, H. & Geurink, P. P. Native chemical ligation at methionine bioisostere norleucine allows for N-terminal chemical protein ligation. *Org. Biomol. Chem.* **16**, 6306–6315 (2018).
2. Wan, Q. & Danishefsky, S. J. Free-radical-based, specific desulfurization of cysteine: A powerful advance in the synthesis of polypeptides and glycopolypeptides. *Angew. Chem. Int. Ed.* **46**, 9248–9252 (2007).
3. Zhou, Z. & Fahrni, C. J. A Fluorogenic Probe for the Copper(I)-Catalyzed Azide−Alkyne Ligation Reaction: Modulation of the Fluorescence Emission via 3 (n,π*) − (π,π*) Inversion. *J. Am. Chem. Soc.* **126**, 8862–8863 (2004).
4. Kim, R. Q. *et al.* Kinetic analysis of multistep USP7 mechanism shows critical role for target protein in activity. *Nat. Commun.* **10**, 231 (2019).
5. Paudel, P. *et al.* Crystal structure and activity-based labeling reveal the mechanisms for linkage-specific substrate recognition by deubiquitinase USP9X. *Proc. Natl. Acad. Sci.* **116**, 7288–7297 (2019).
6. Luna-Vargas, M. P. A. *et al.* Enabling high-throughput ligation-independent cloning and protein expression for the family of ubiquitin specific proteases. *J. Struct. Biol.* **175**, 113–119 (2011).
7. Mons, E. *et al.* Exploring the Versatility of the Covalent Thiol-Alkyne Reaction with Substituted Propargyl Warheads: A Deciding Role for the Cysteine Protease. *J. Am. Chem. Soc.* **143**, 6423–6433 (2021).
8. Ye, Y. *et al.* Polyubiquitin binding and cross-reactivity in the USP domain deubiquitinase USP21. *EMBO Rep.* **12**, 350–357 (2011).
9. Sapmaz, A. *et al.* USP32 regulates late endosomal transport and recycling through deubiquitylation of Rab7. *Nat. Commun.* **10**, 1454 (2019).
10. Mevissen, T. E. T. *et al.* OTU Deubiquitinases Reveal Mechanisms of Linkage Specificity and Enable Ubiquitin Chain Restriction Analysis. *Cell* **154**, 169–184 (2013).
11. Nanao, M. H., Tcherniuk, S. O., Chroboczek, J., Dideberg, O. & Balakirev, M. Y. *EMBO Rep.* **5**, 783–788 (2004).
12. Hameed, D. S. *et al.* Development of Ubiquitin-Based Probe for Metalloprotease Deubiquitinases. *Angew. Chem. Int. Ed.* **58**, 14477–14482 (2019).
13. El Oualid, F. *et al.* Chemical synthesis of ubiquitin, ubiquitin-based probes, and diubiquitin. *Angew. Chem. Int. Ed.* **49**, 10149–10153 (2010).
14. Van Der Heden Van Noort, G. J., Kooij, R., Elliott, P. R., Komander, D. & Ovaa, H. Synthesis of Poly-Ubiquitin Chains Using a Bifunctional Ubiquitin Monomer. *Org. Lett.* **19**, 6490–6493 (2017).
15. Geurink, P. P. *et al.* Development of Diubiquitin-Based FRET Probes To Quantify Ubiquitin Linkage Specificity of Deubiquitinating Enzymes. *ChemBioChem* **17**, 816–820 (2016).
16. Ekkebus, R. *et al.* On Terminal Alkynes That Can React with Active-Site Cysteine Nucleophiles in Proteases. *J. Am. Chem. Soc.* **135**, 2867–2870 (2013).
17. Flierman, D. *et al.* Non-hydrolyzable Diubiquitin Probes Reveal Linkage-Specific Reactivity of Deubiquitylating Enzymes Mediated by S2 Pockets. *Cell Chem. Biol.* **23**, 472–482 (2016).
