# Supplementary material for: Neutron-encoded diubiquitins to profile linkage selectivity of deubiquitinating enzymes
Source: Nat Commun. 2023 Mar 25;14:1661. doi: 10.1038/s41467-023-37363-6 (PMC10039891; doi:10.1038/s41467-023-37363-6)
Supplement: Supplementary file 3 — Description of Additional Supplementary Files [file 41467_2023_37363_MOESM3_ESM.docx]

**Description of Additional Supplementary Files**

File Name: Supplementary Data 1

Description: LC-MS analysis of all synthesized mono- and diubiquitins.

File Name: Supplementary Data 2

Description: Determinations of the linkage specificities of 22 DUBs. Curves showing the quantified assay results of diUb consumption and monoUb formation.
