# Supplementary material for: Neutron-encoded diubiquitins to profile linkage selectivity of deubiquitinating enzymes
Source: Nat Commun. 2023 Mar 25;14:1661. doi: 10.1038/s41467-023-37363-6 (PMC10039891; doi:10.1038/s41467-023-37363-6)

Neutron-encoded K6-linked diUb (4a) **System 2- Gradient 3**

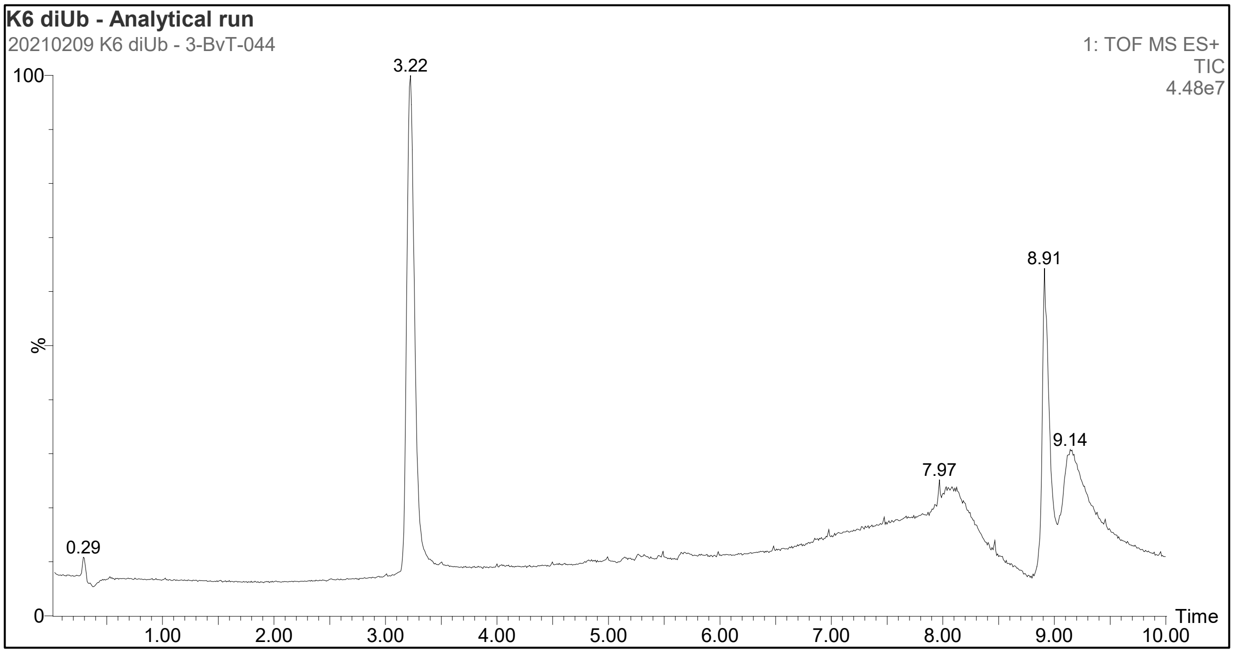

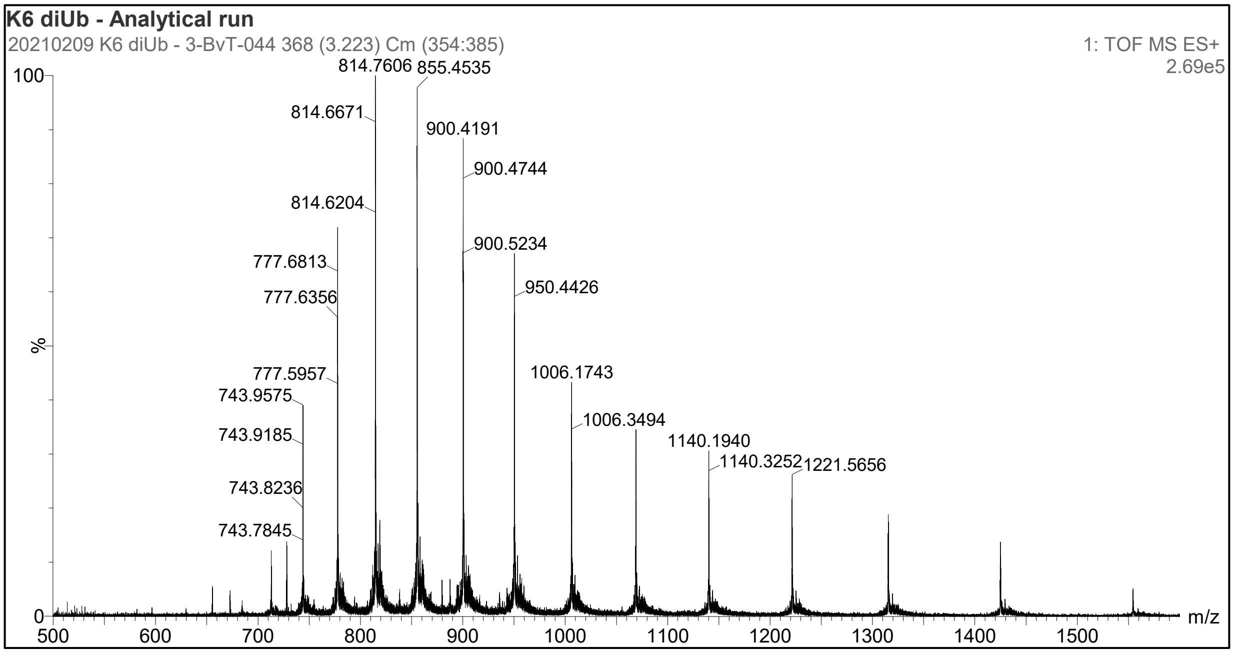

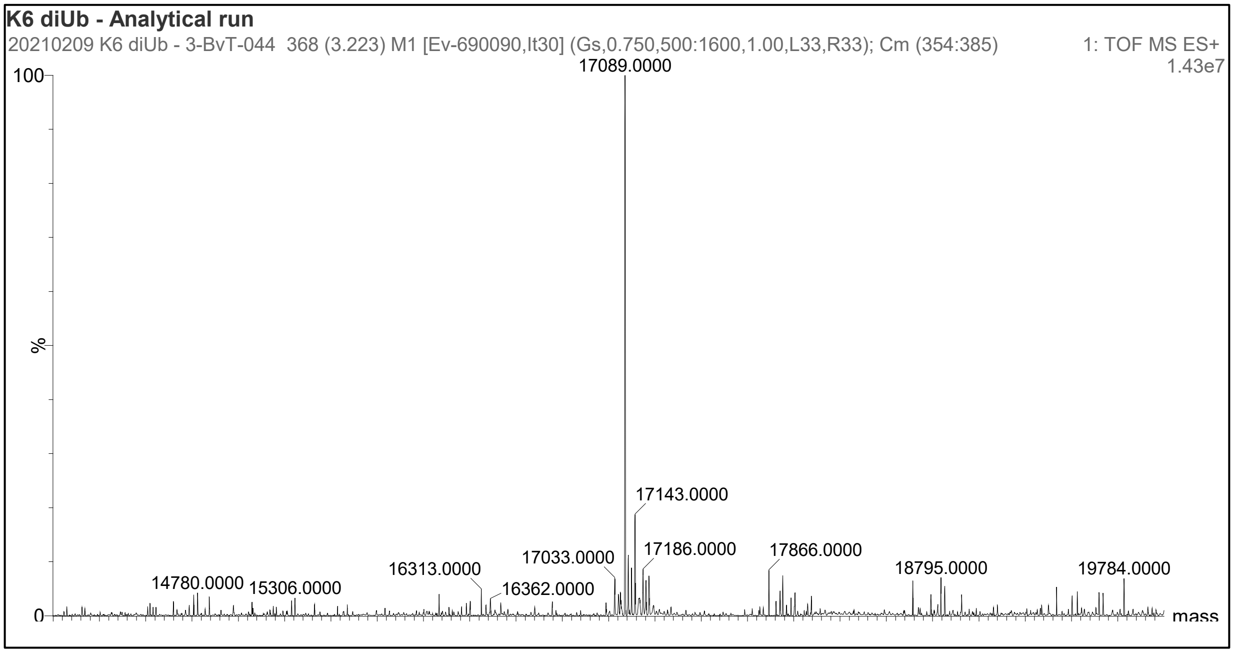

Neutron-encoded K11-linked diUb (4b) **System 2- Gradient 3**

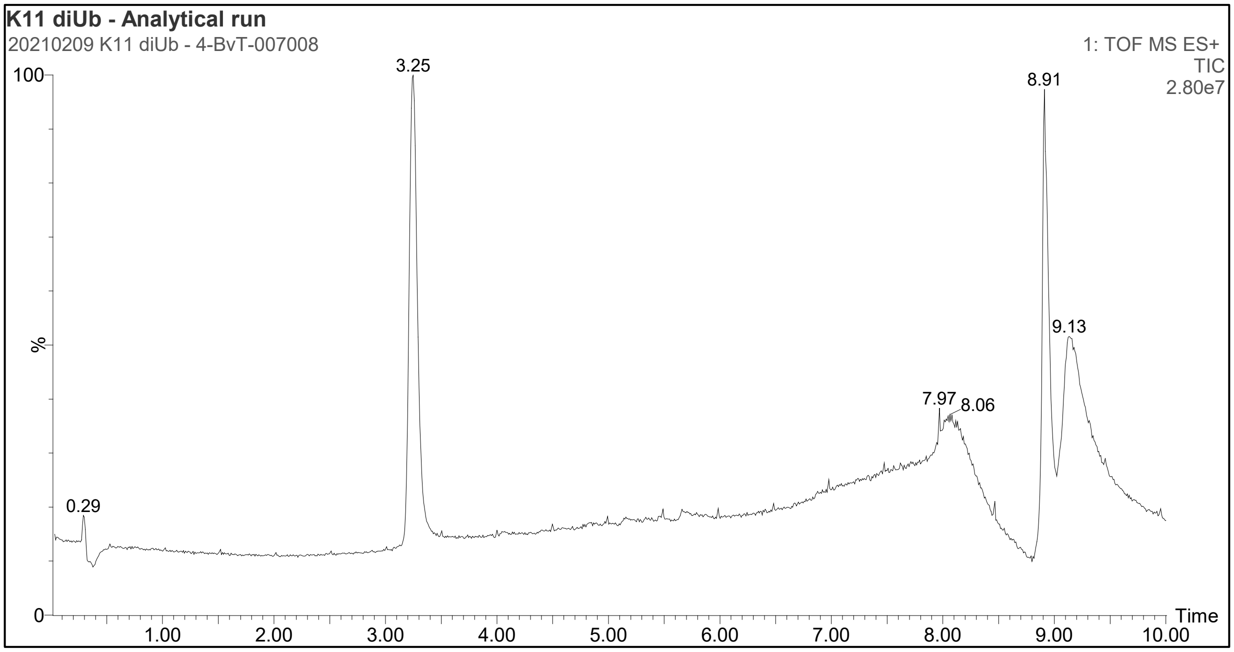

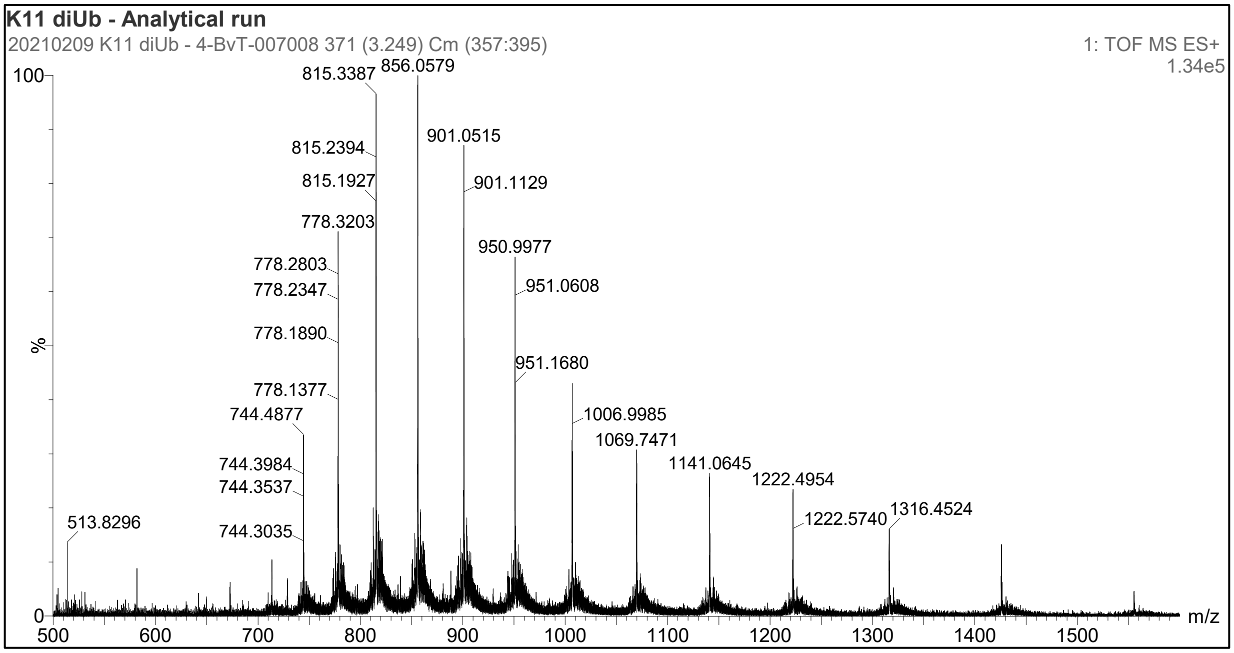

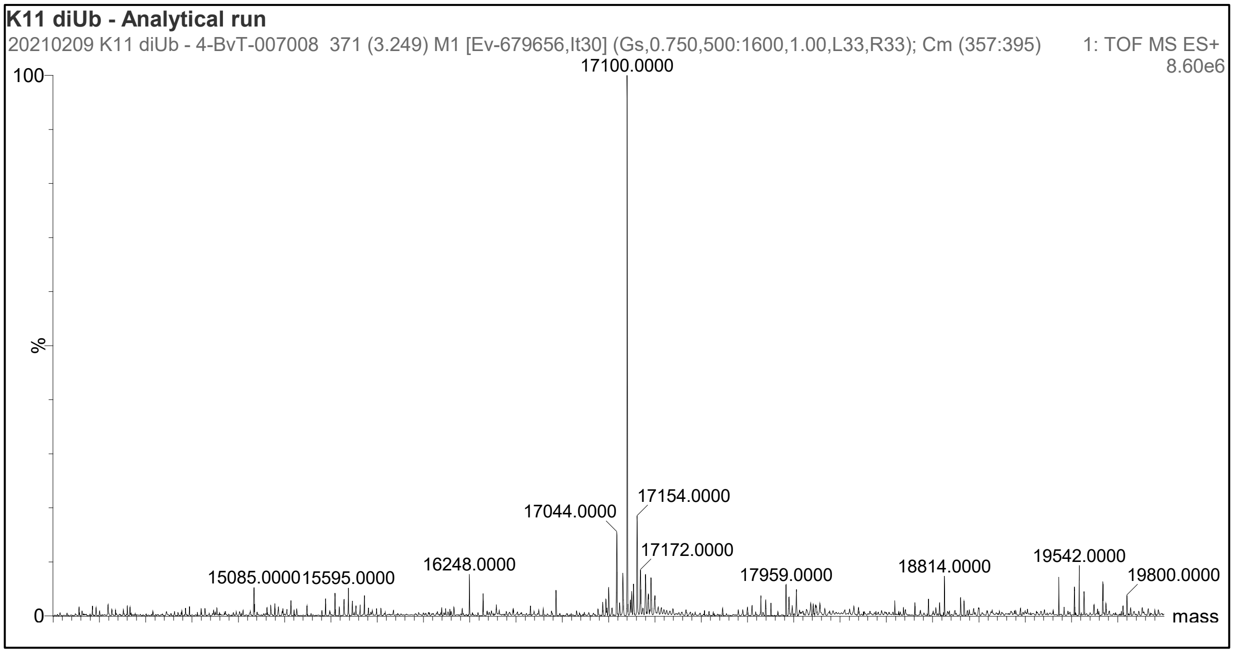

Neutron-encoded K27-linked diUb (4c) **System 2- Gradient 3**

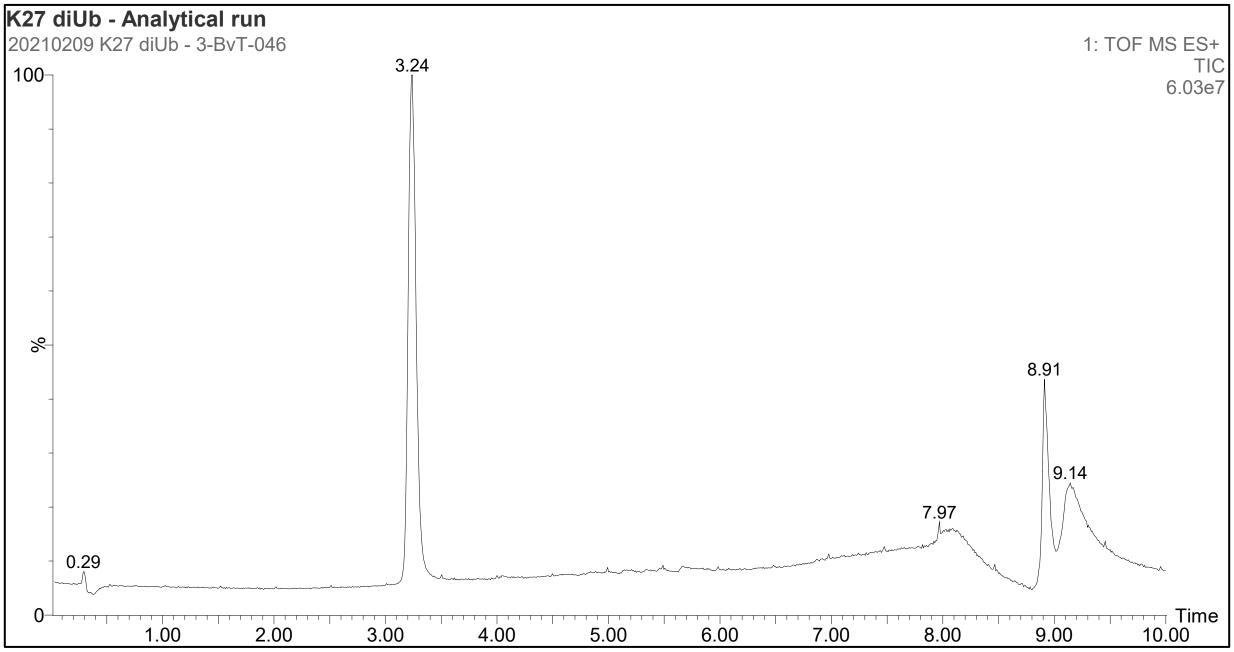

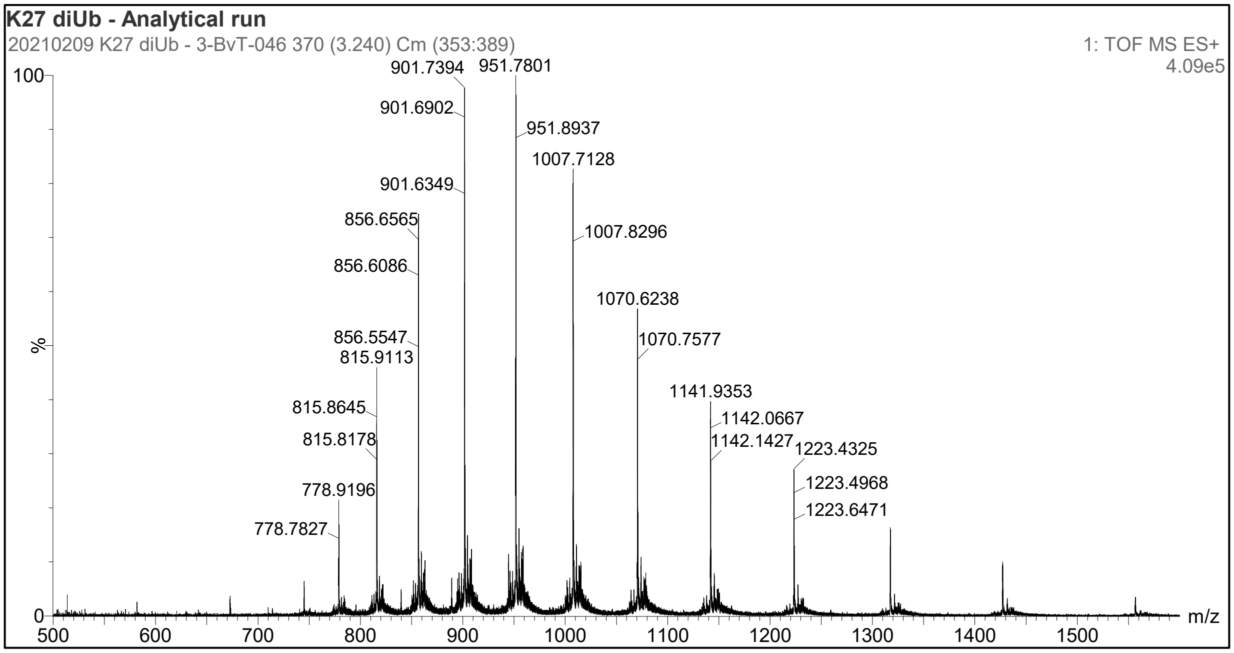

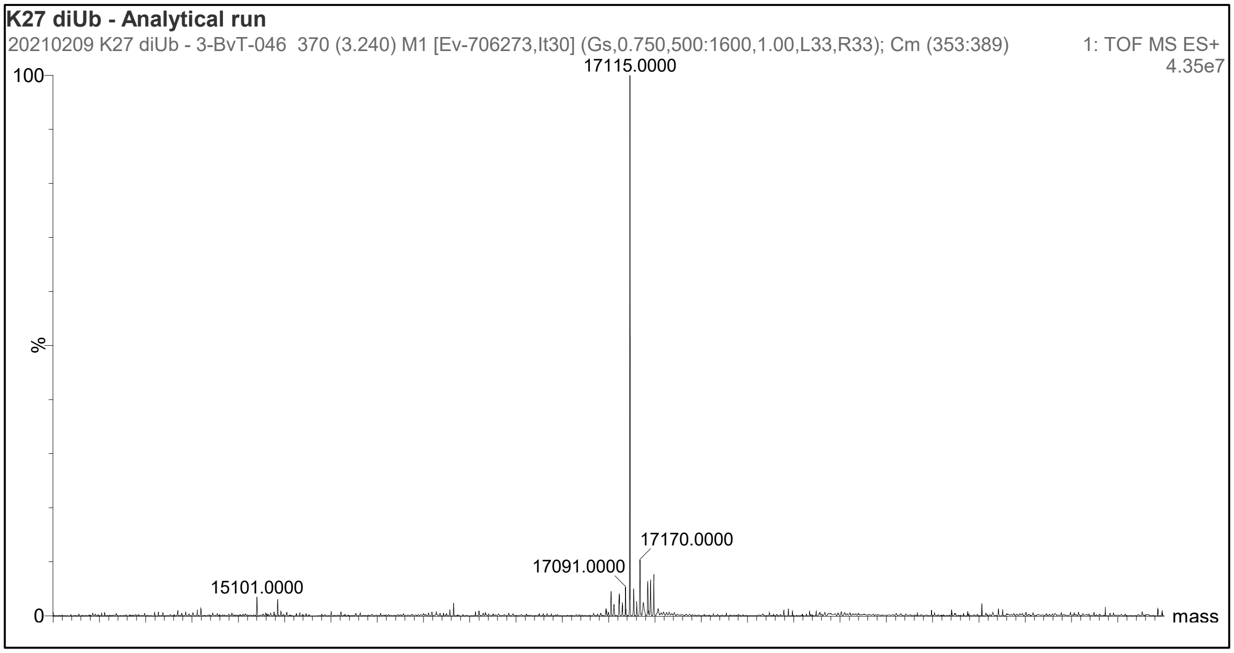

Neutron-encoded K29-linked diUb (4d) **System 2- Gradient 3**

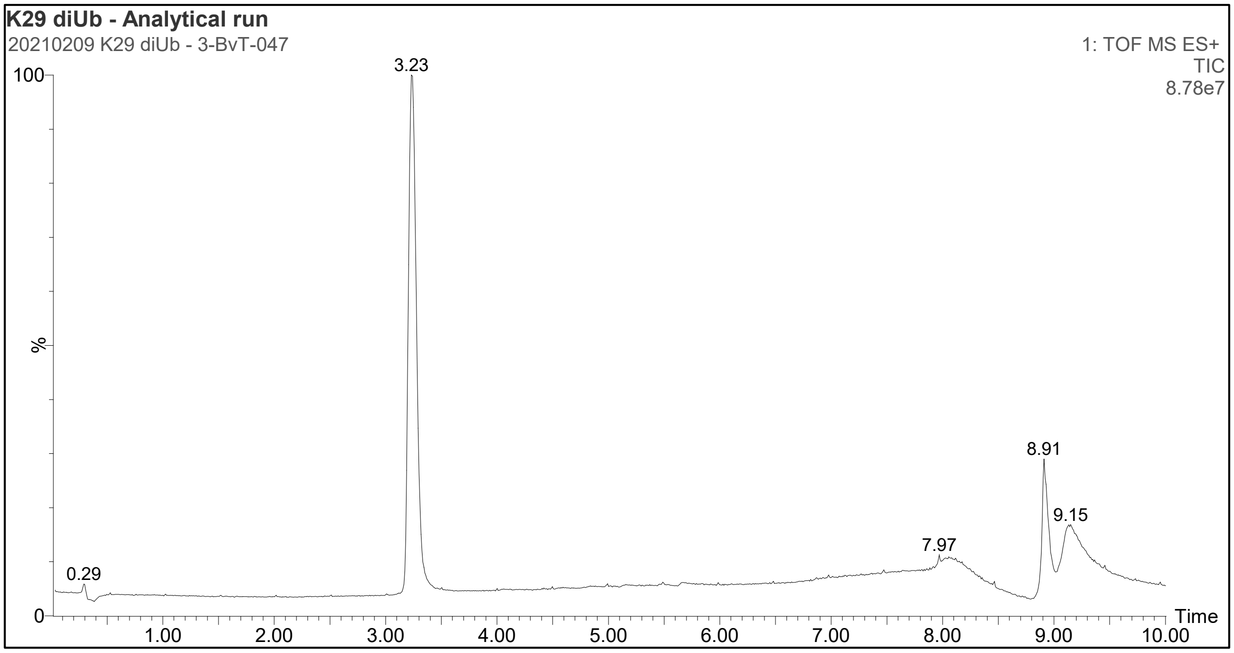

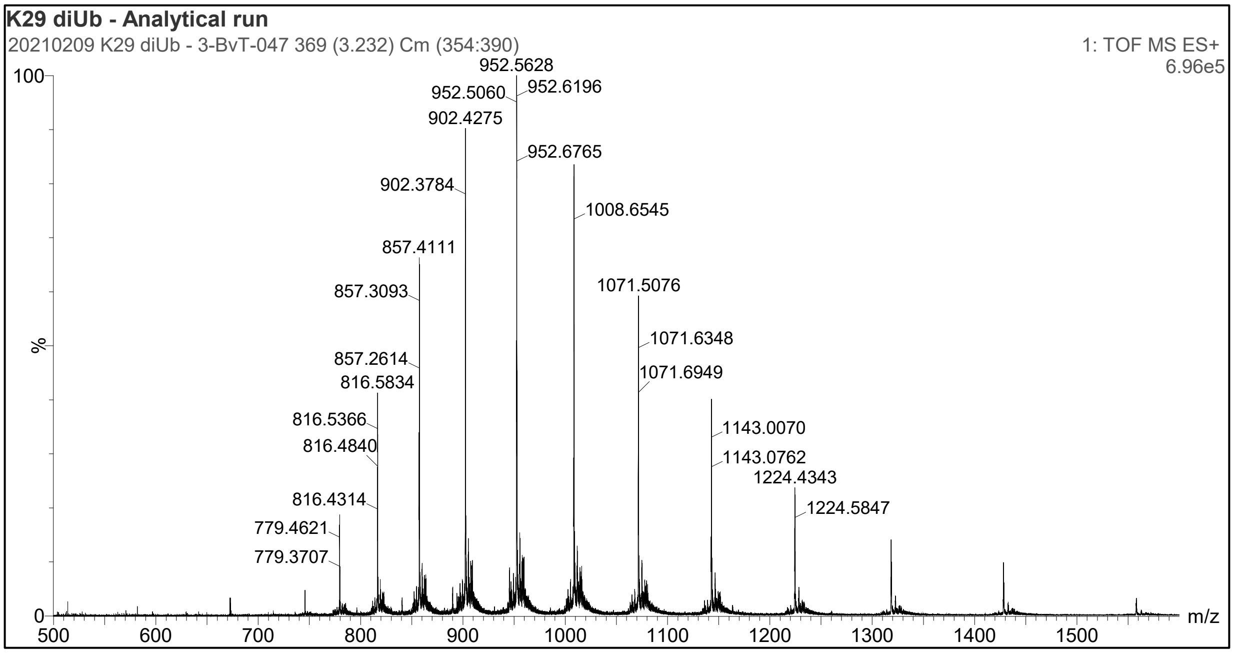

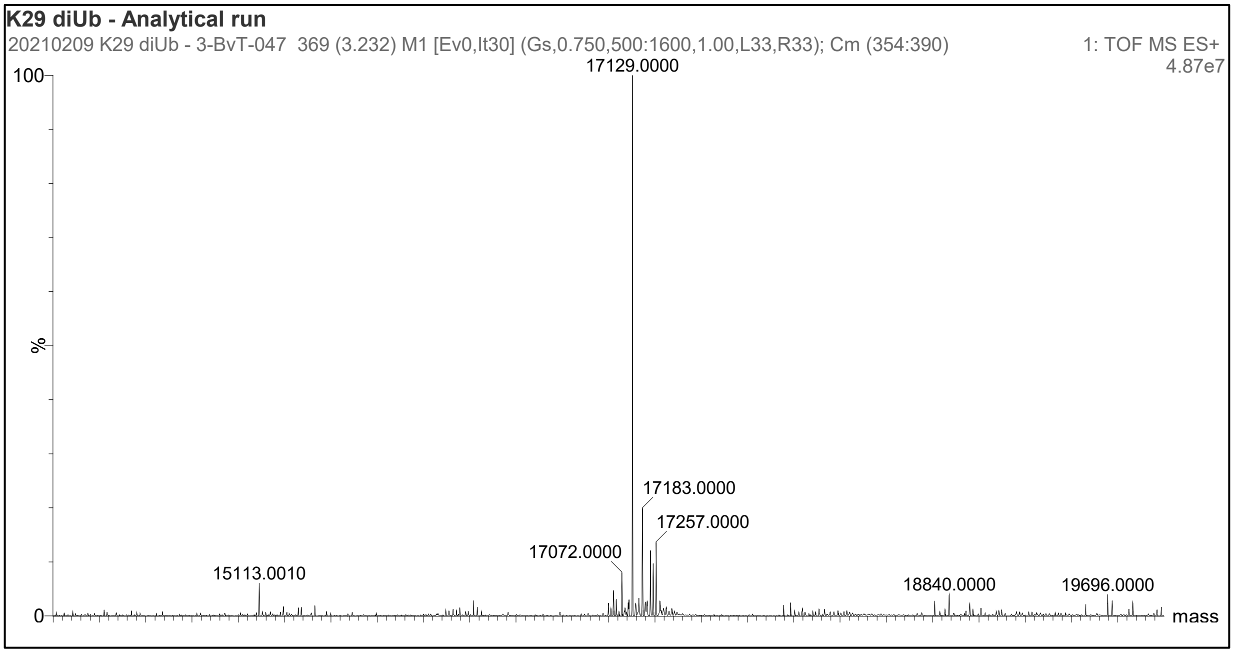

Neutron-encoded K33-linked diUb (4e) **System 2- Gradient 3**

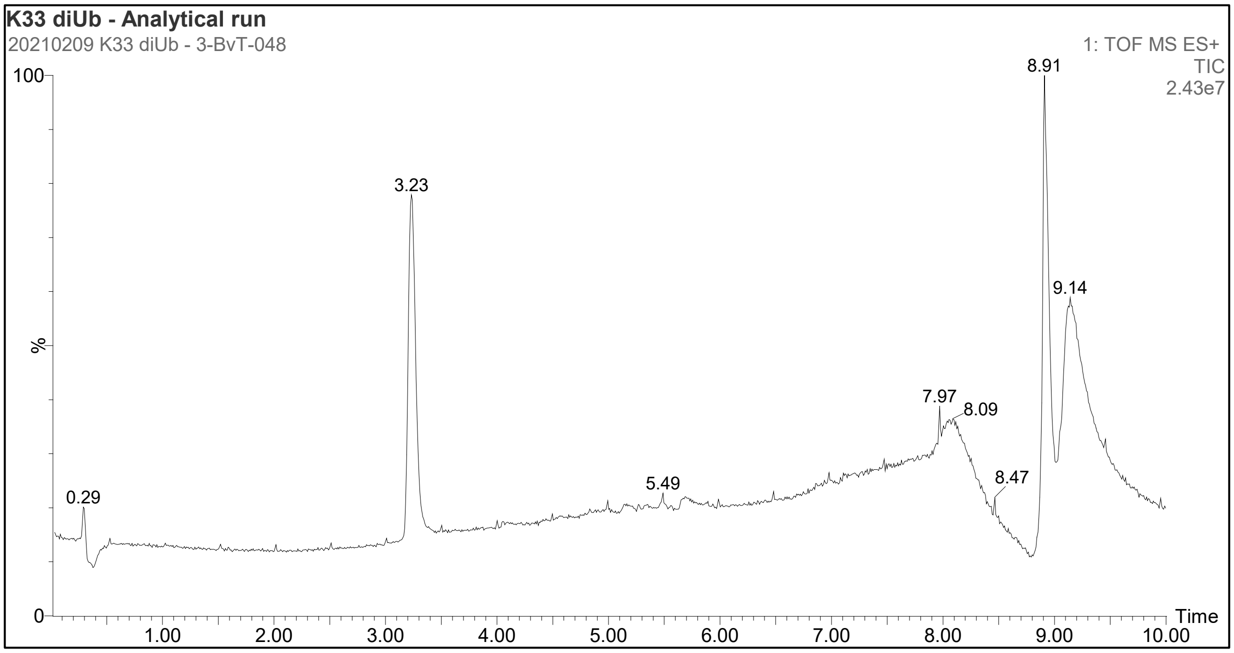

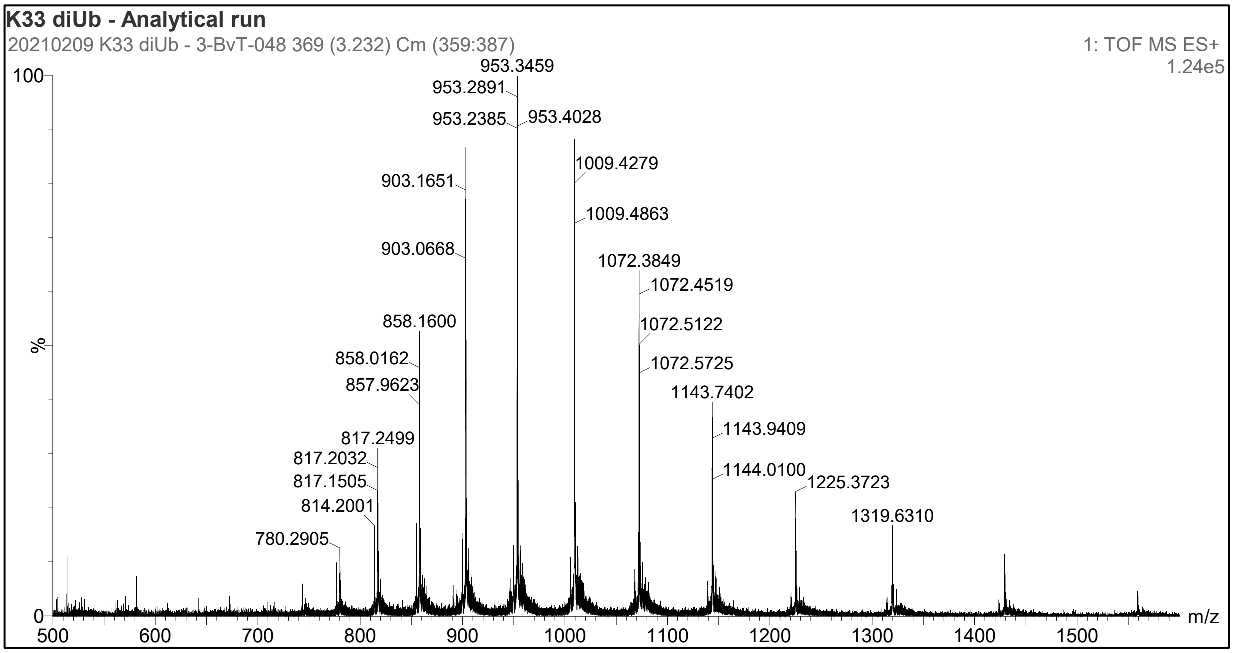

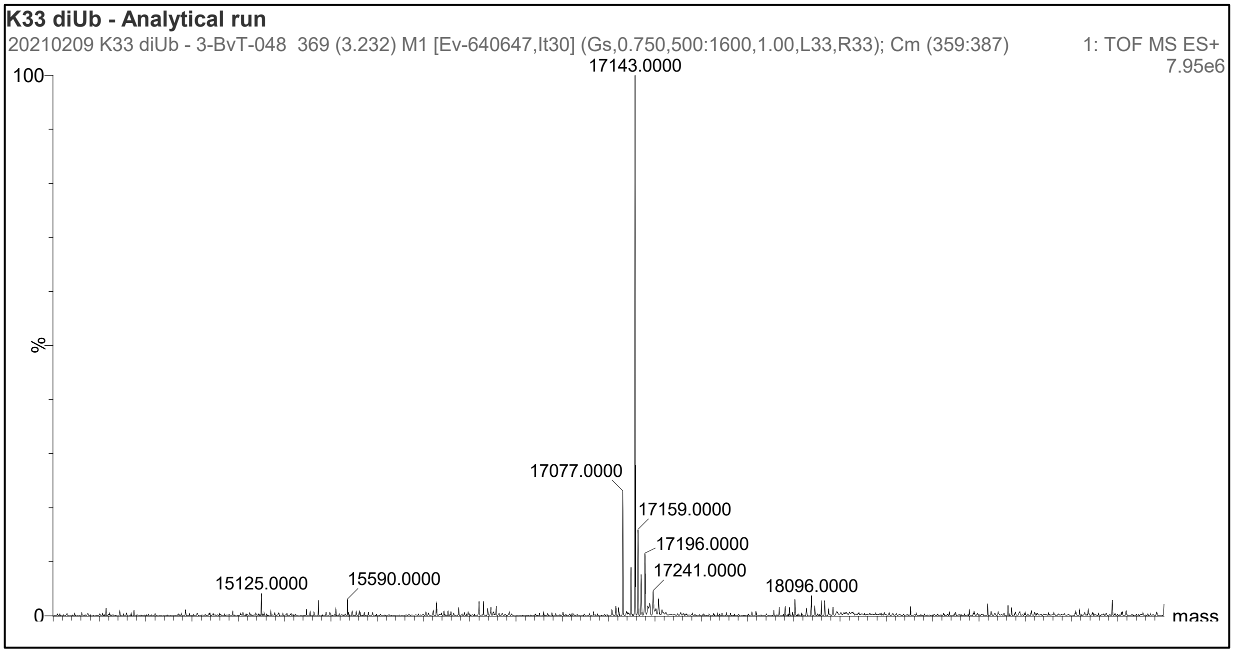

Neutron-encoded K48-linked diUb (4f) **System 2- Gradient 3**

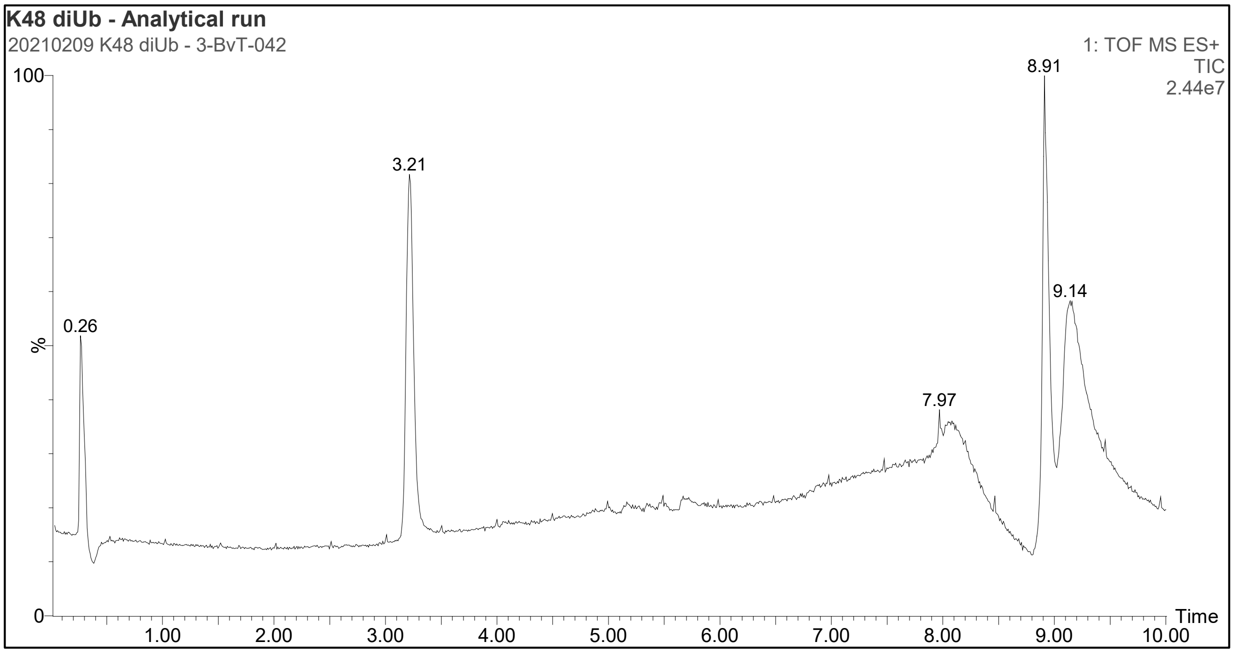

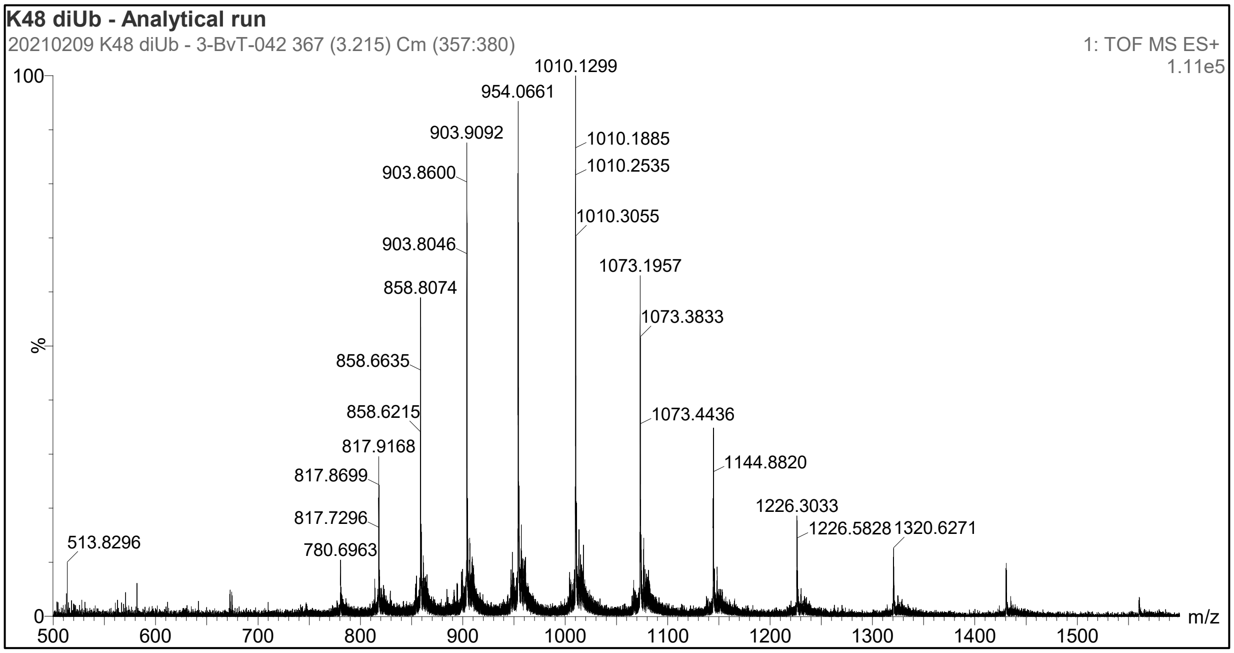

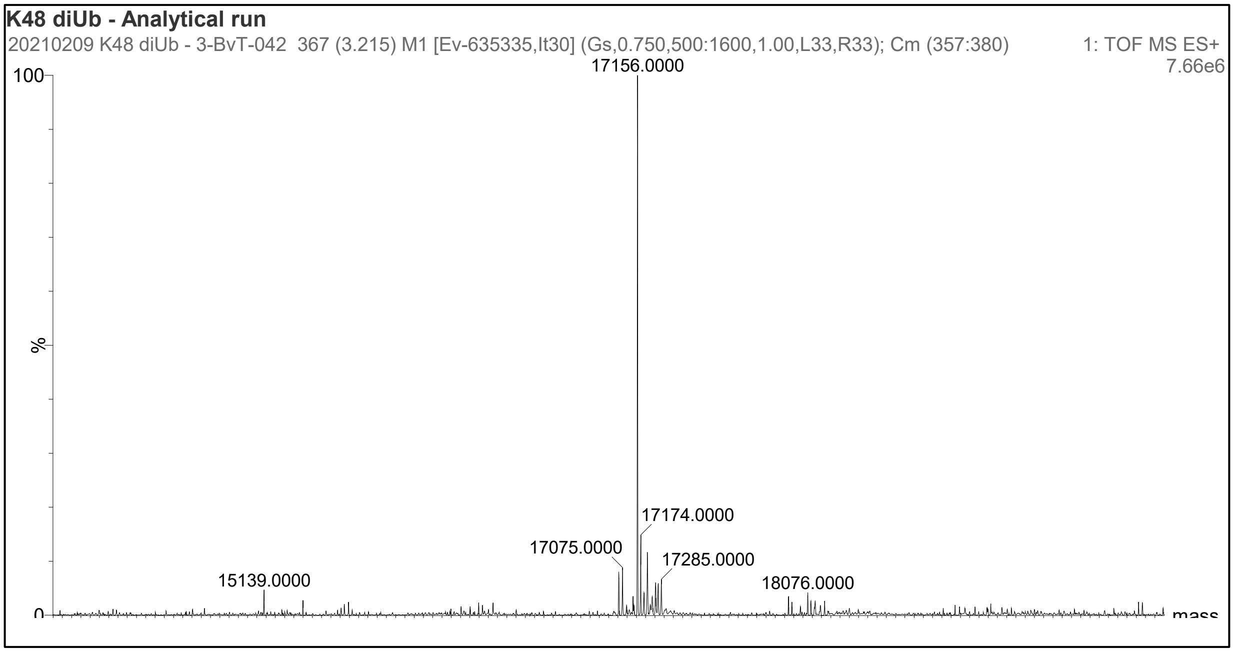

Neutron-encoded K63-linked diUb (4g) **System 2- Gradient 3**

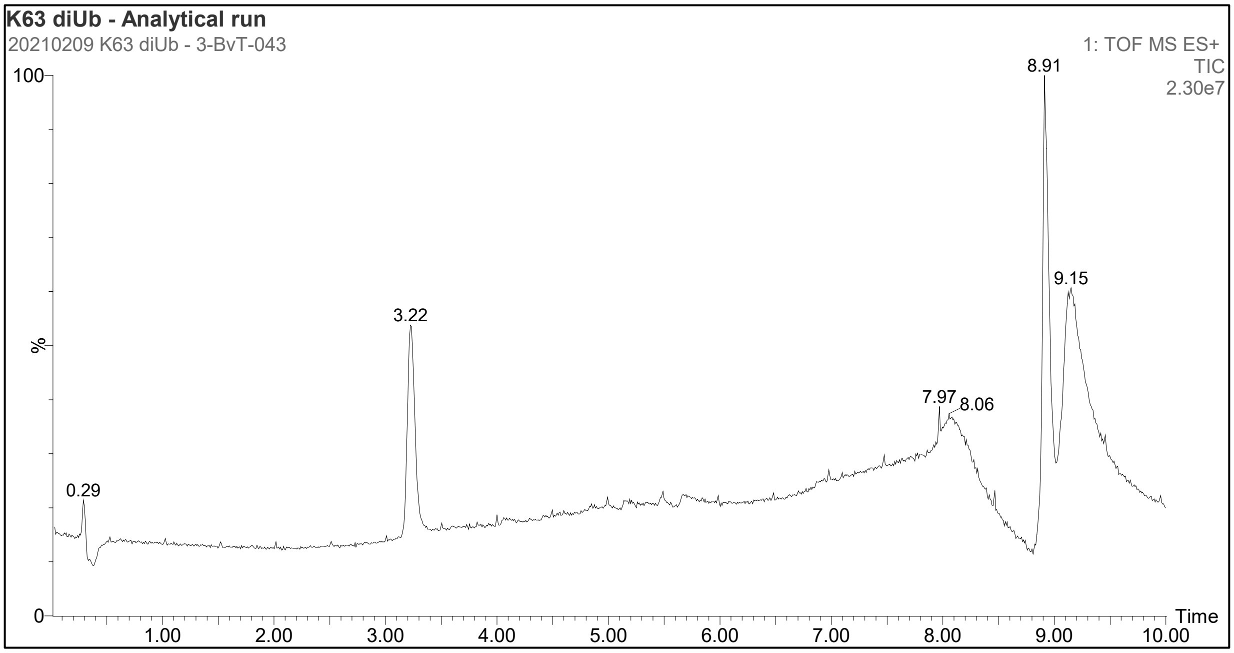

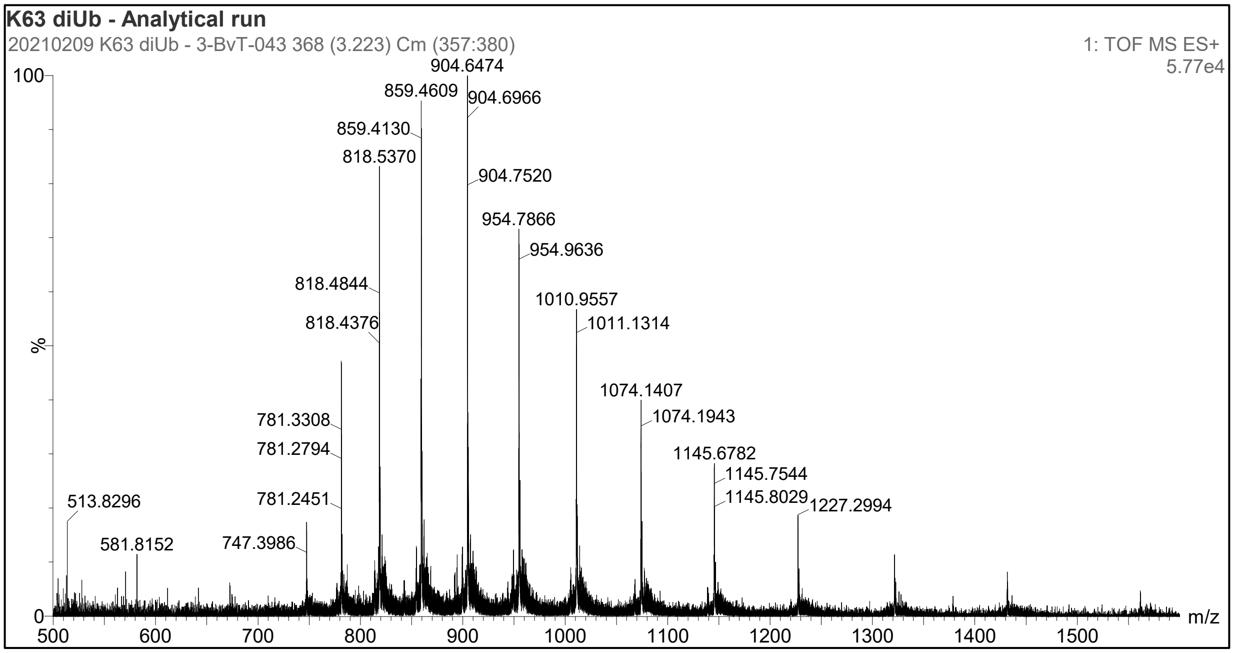

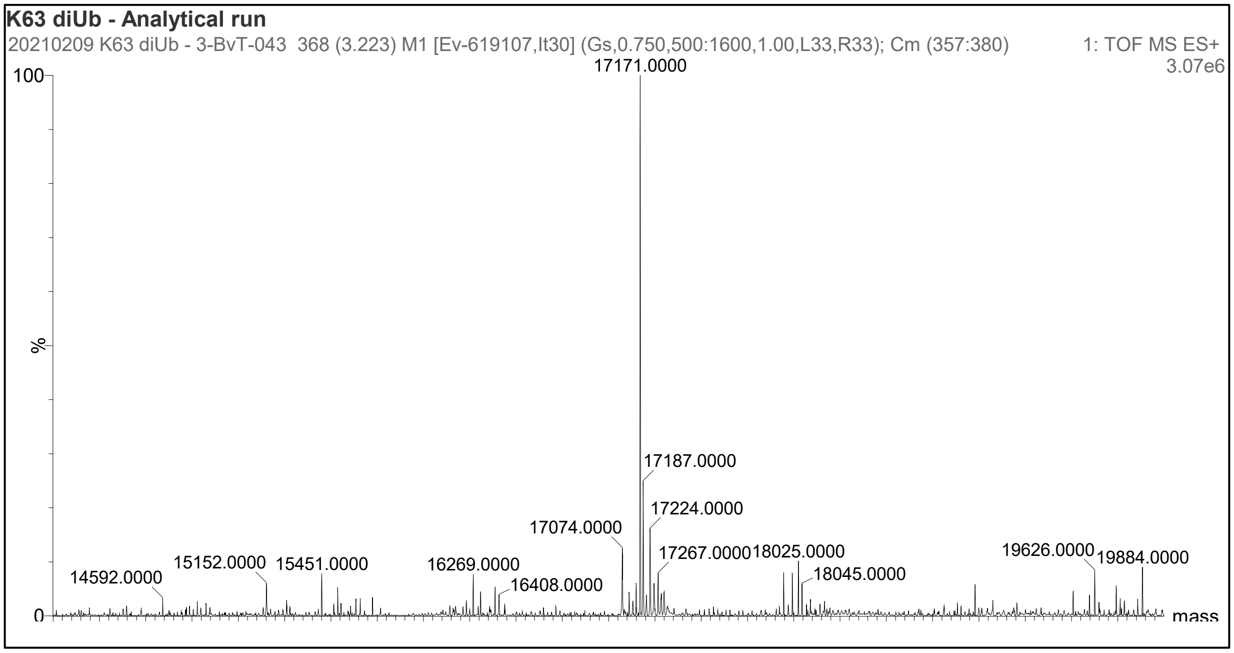

Neutron-encoded M1-linked diUb (5) **System 2- Gradient 3**

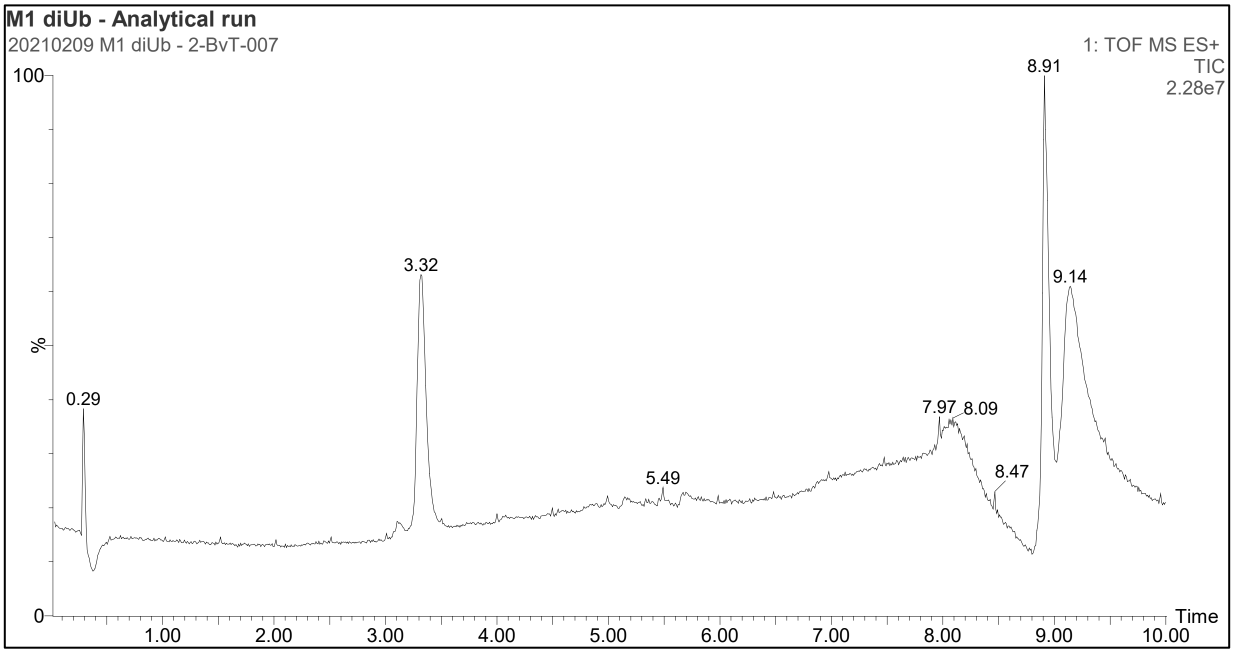

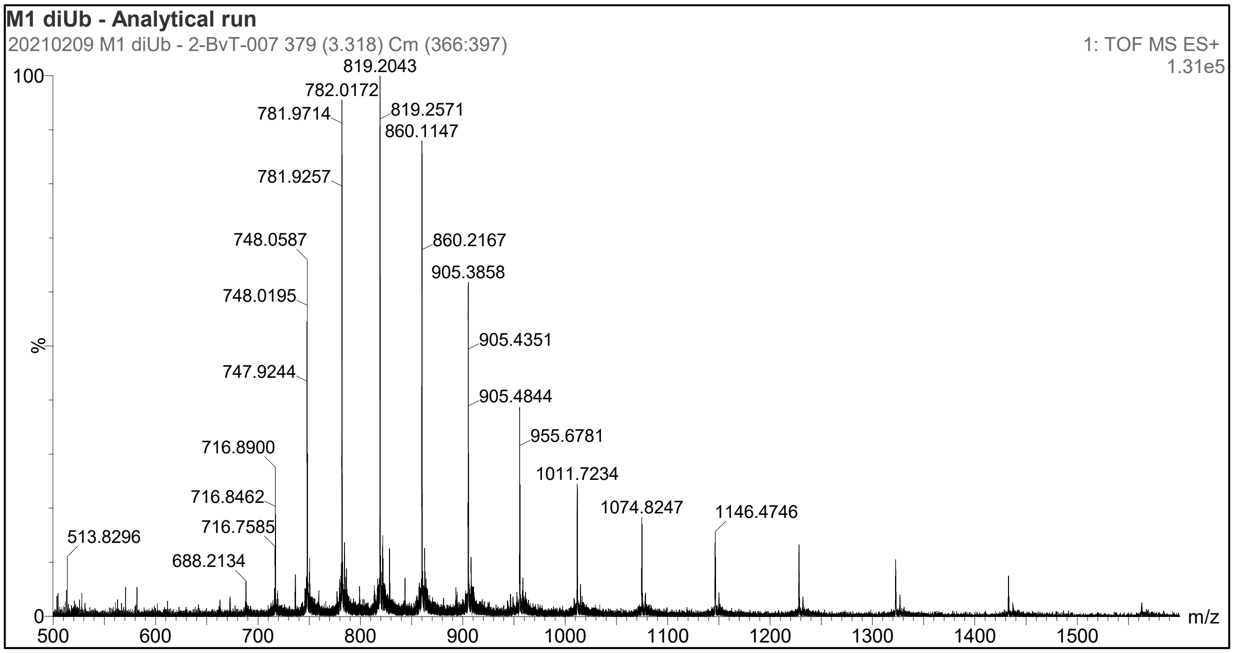

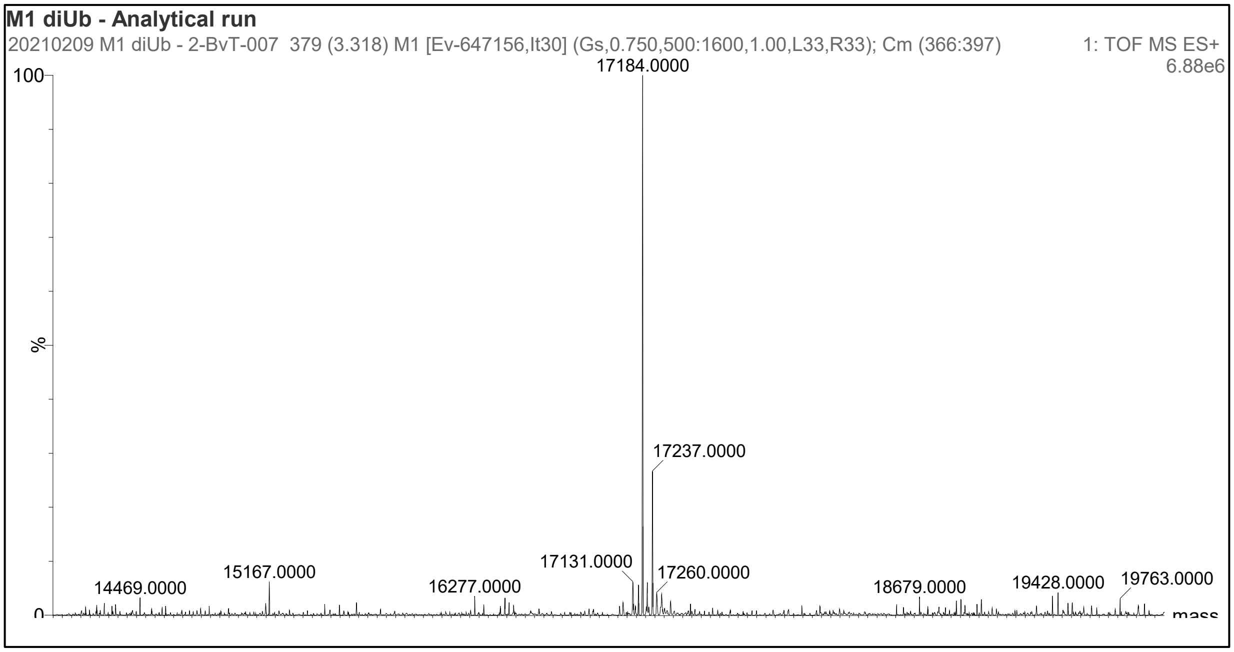

Internal standard – Ub_{1-74} **System 2- Gradient 3**

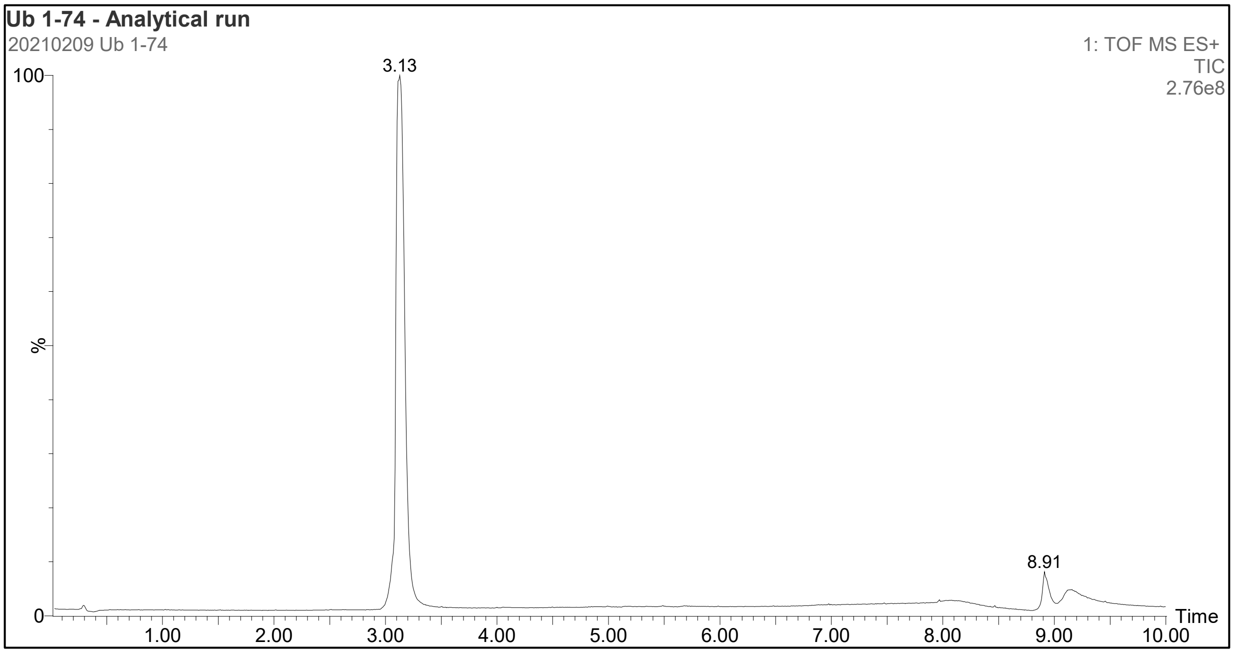

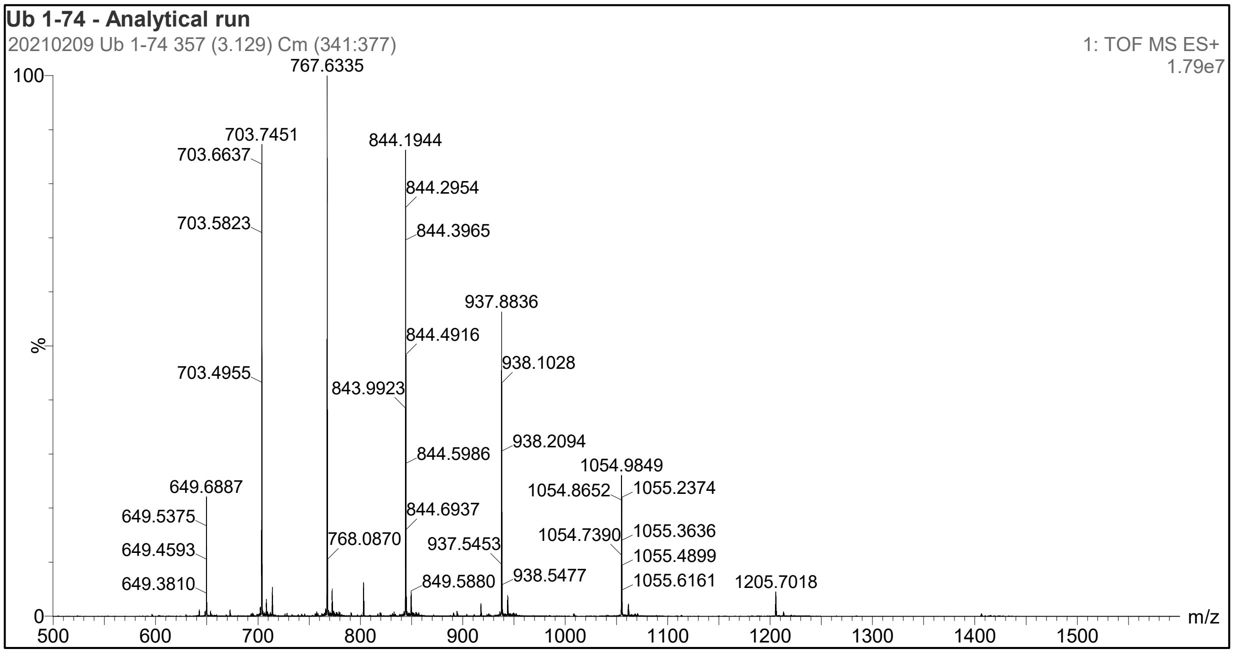

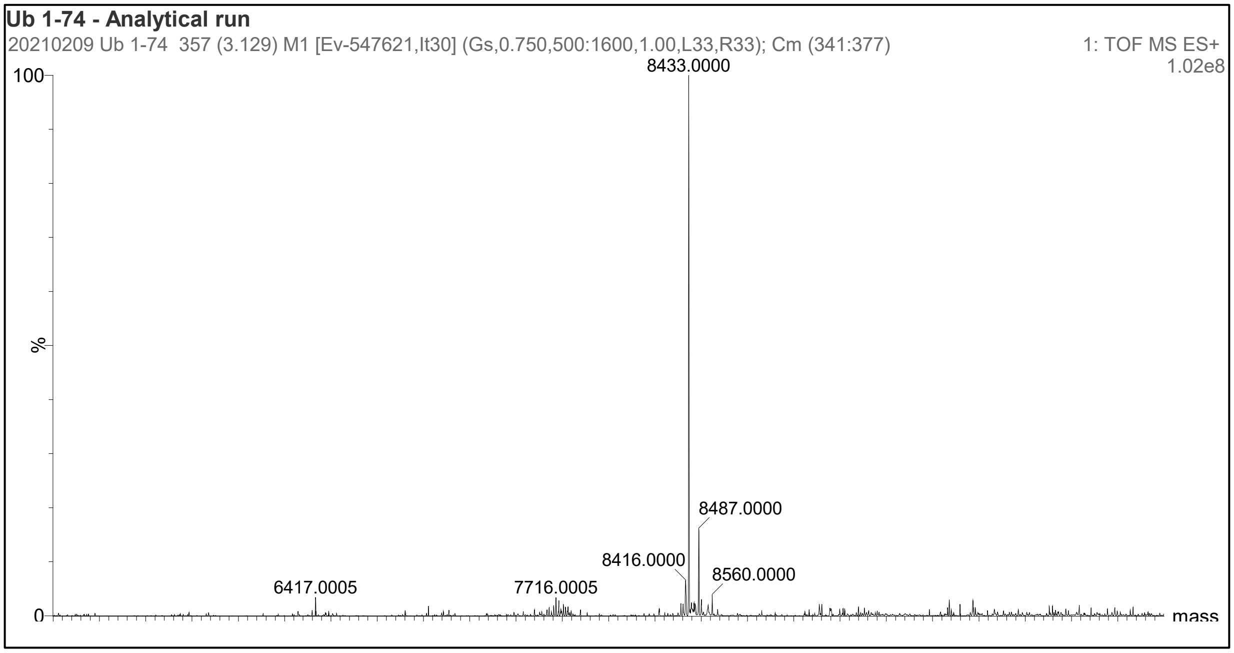

Internal standard – Non-hydrolysable clicked Lys48 linked diUb (23) **System 2- Gradient 3**

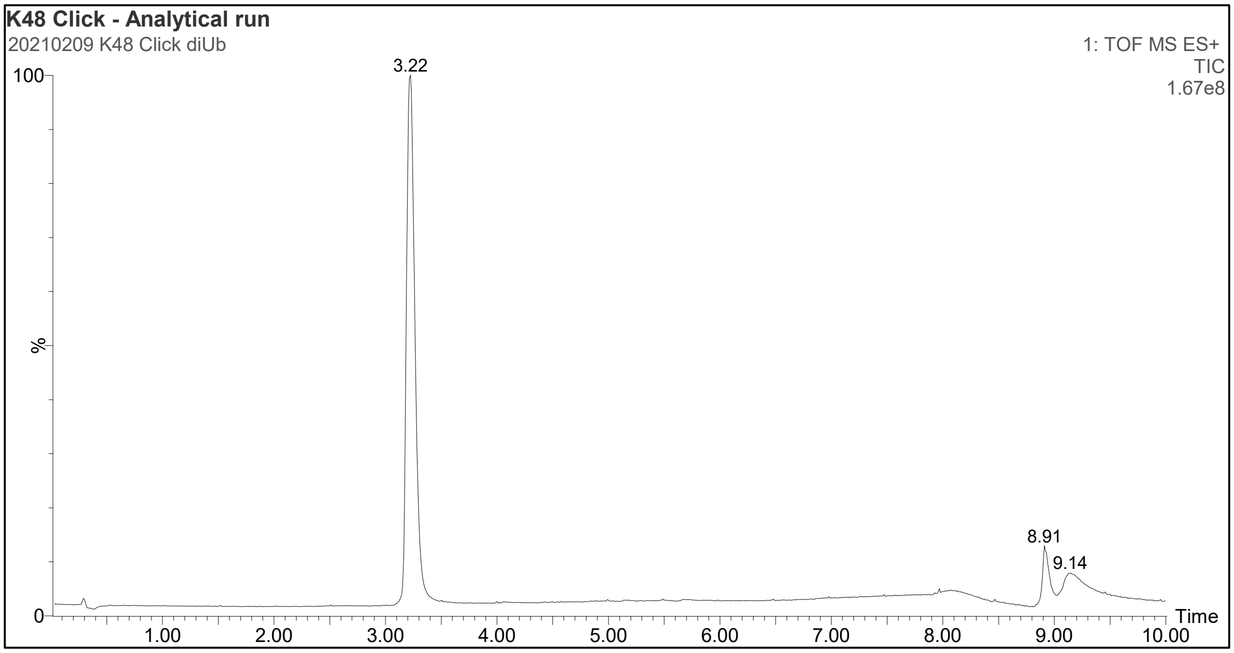

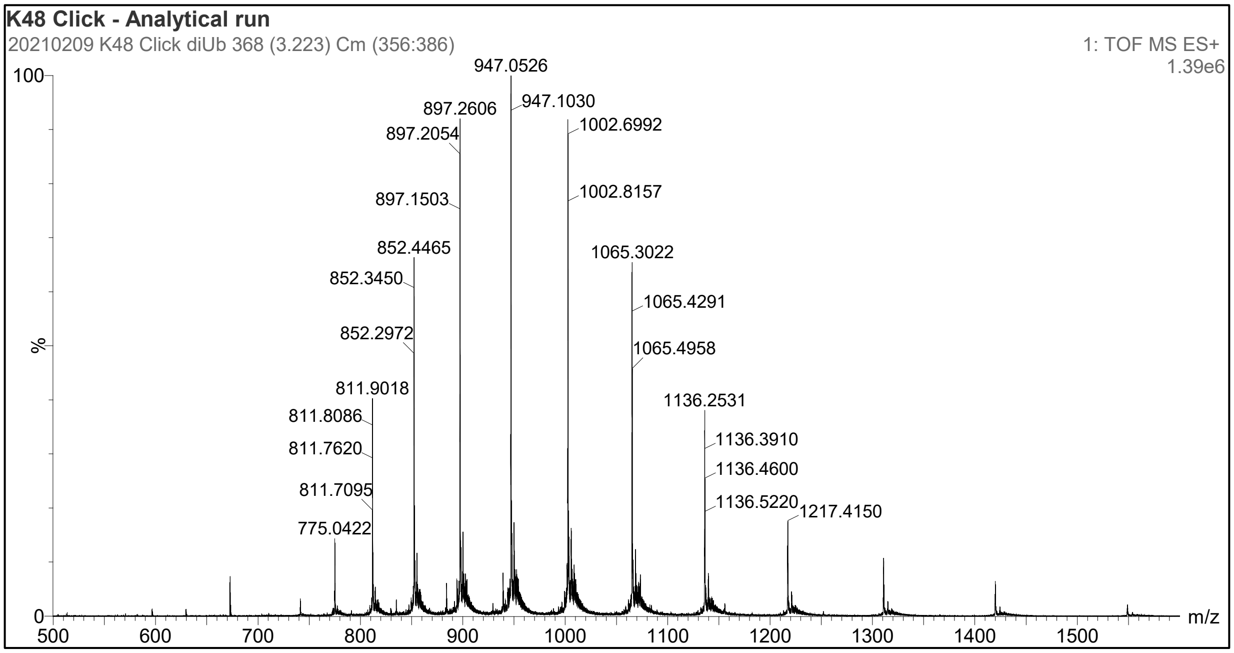

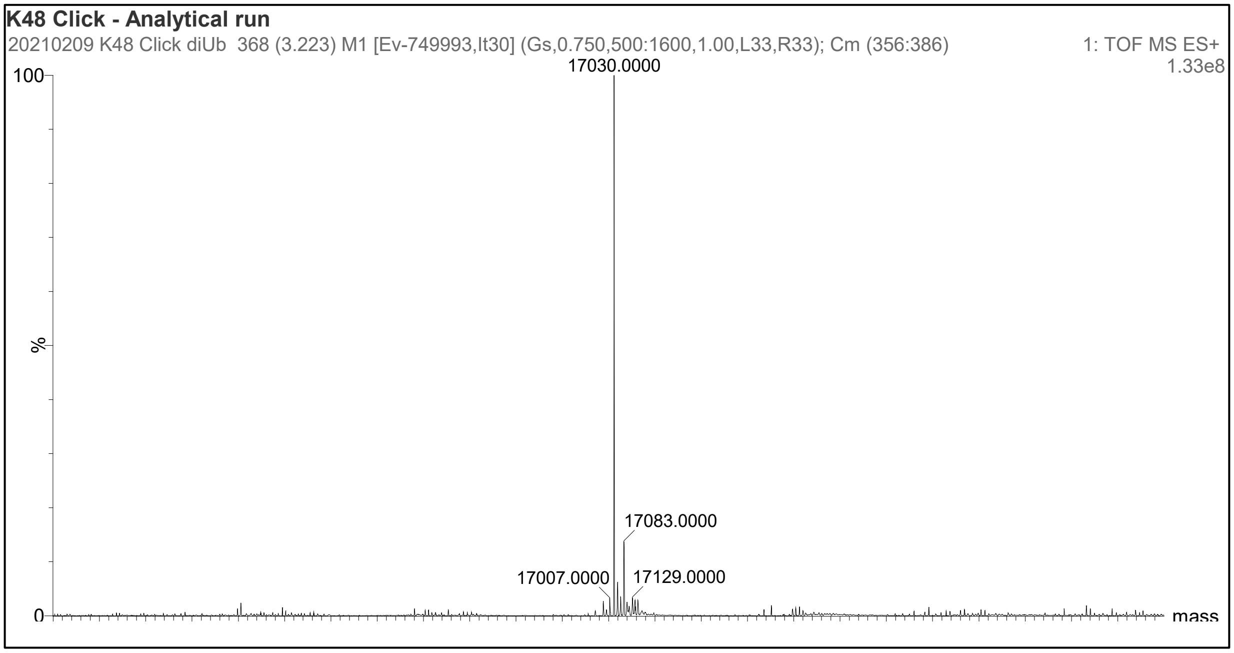

Neutron-encoded monoUb K6 TLys(1a) **System 2- Gradient 3**

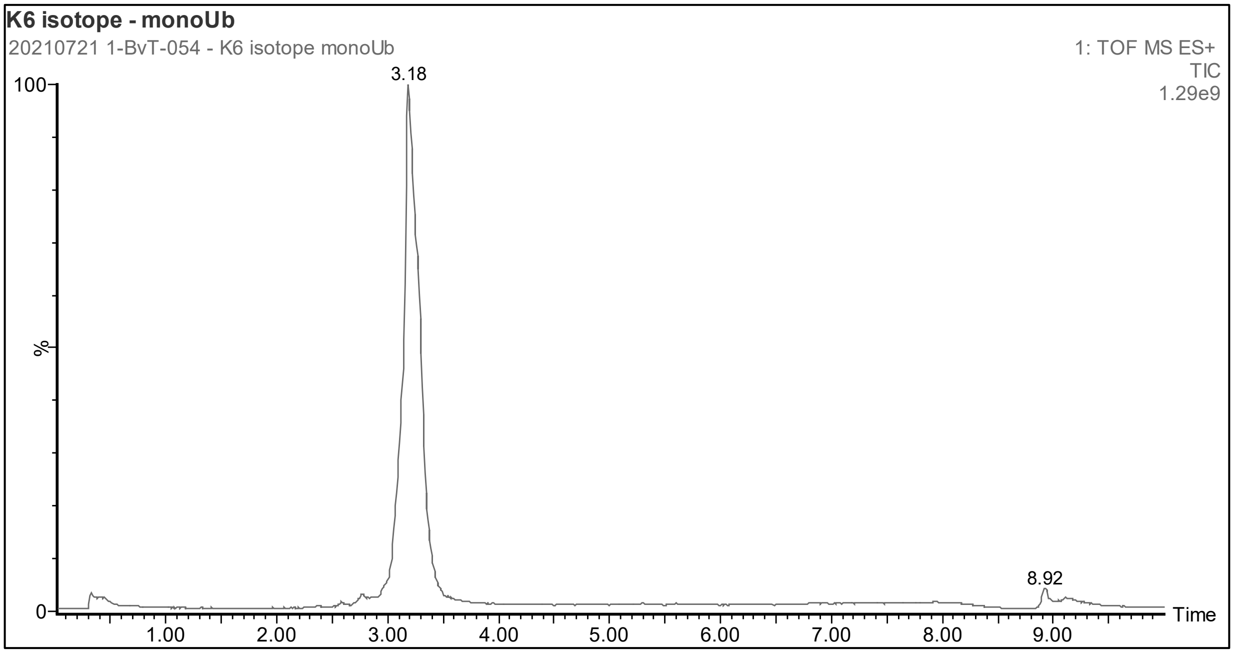

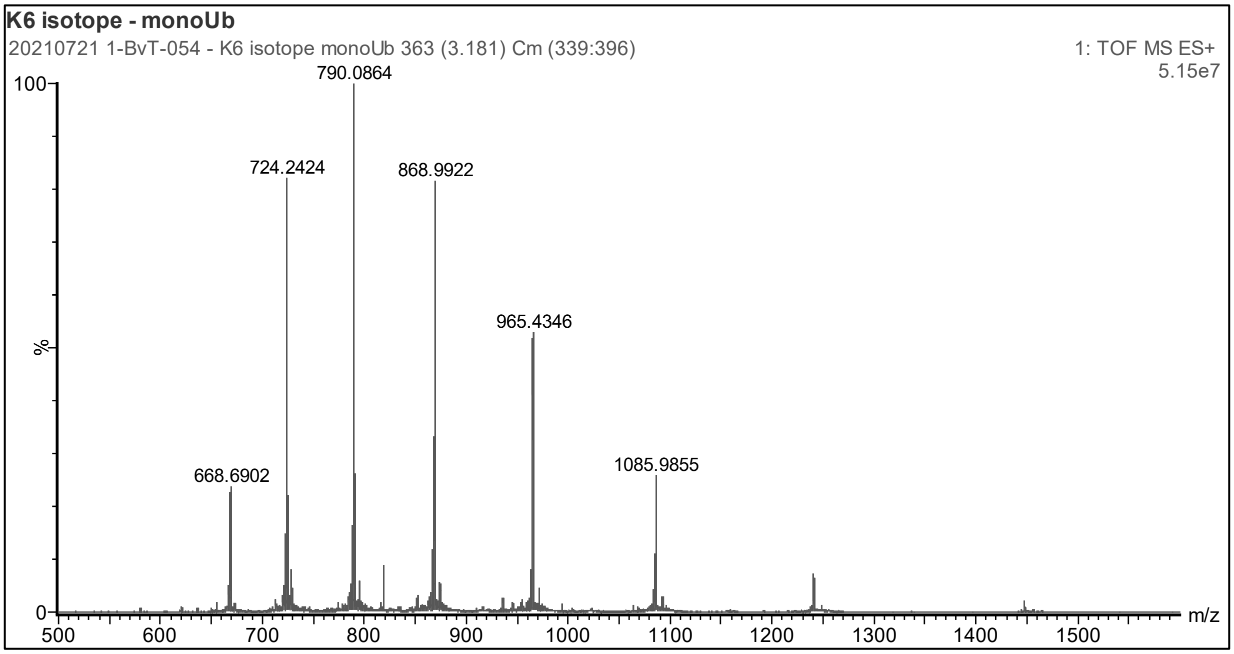

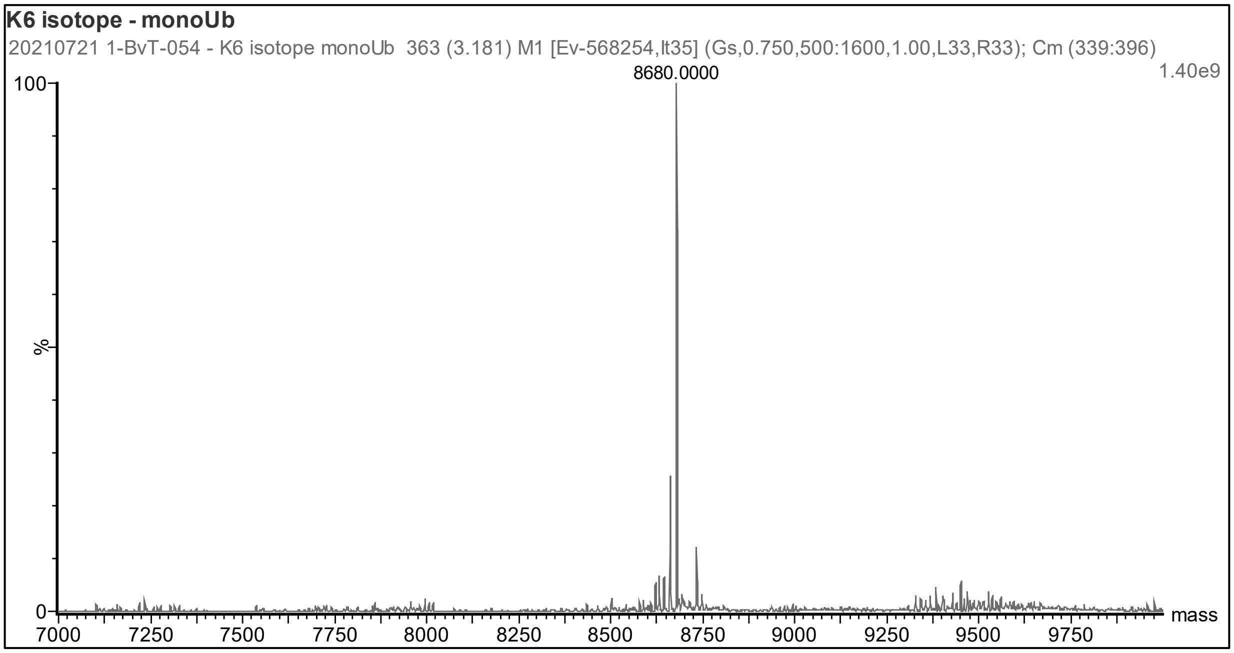

Neutron-encoded monoUb K11 TLys(1b) **System 2- Gradient 3**

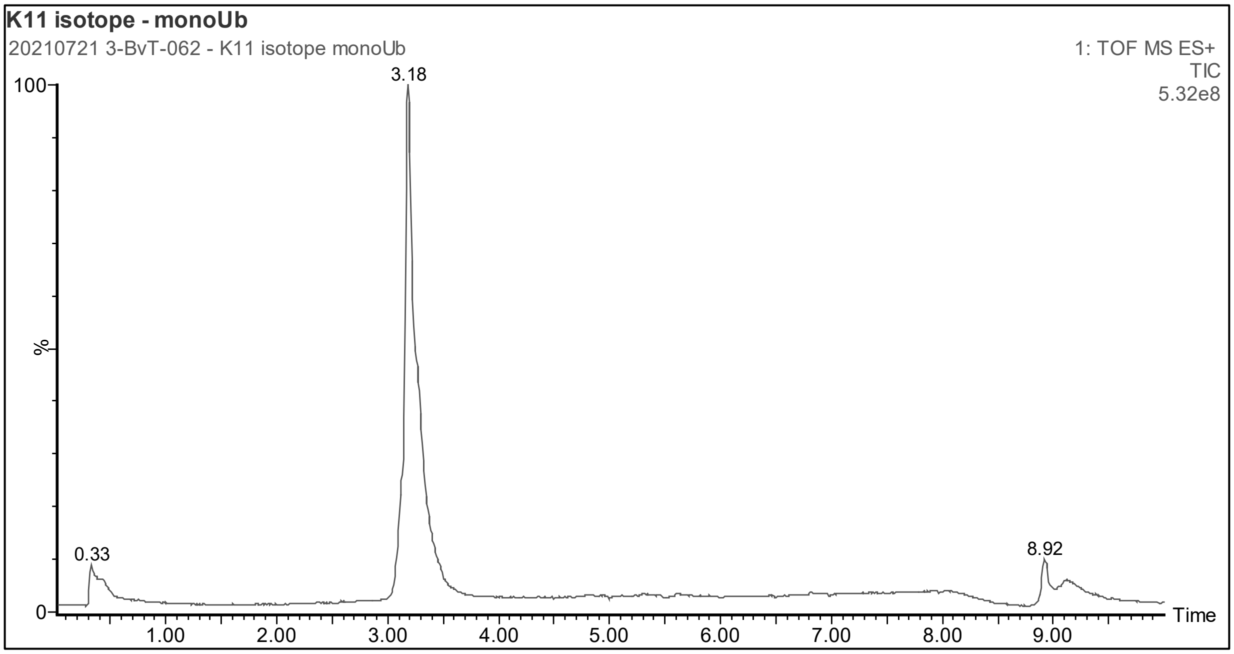

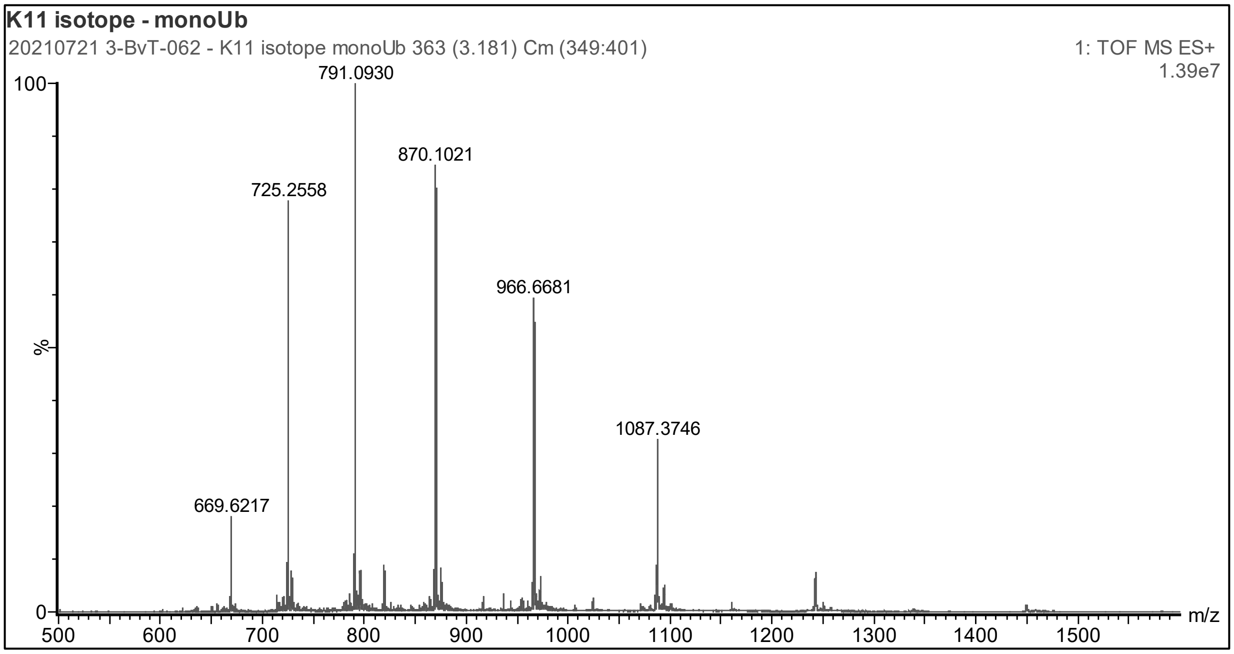

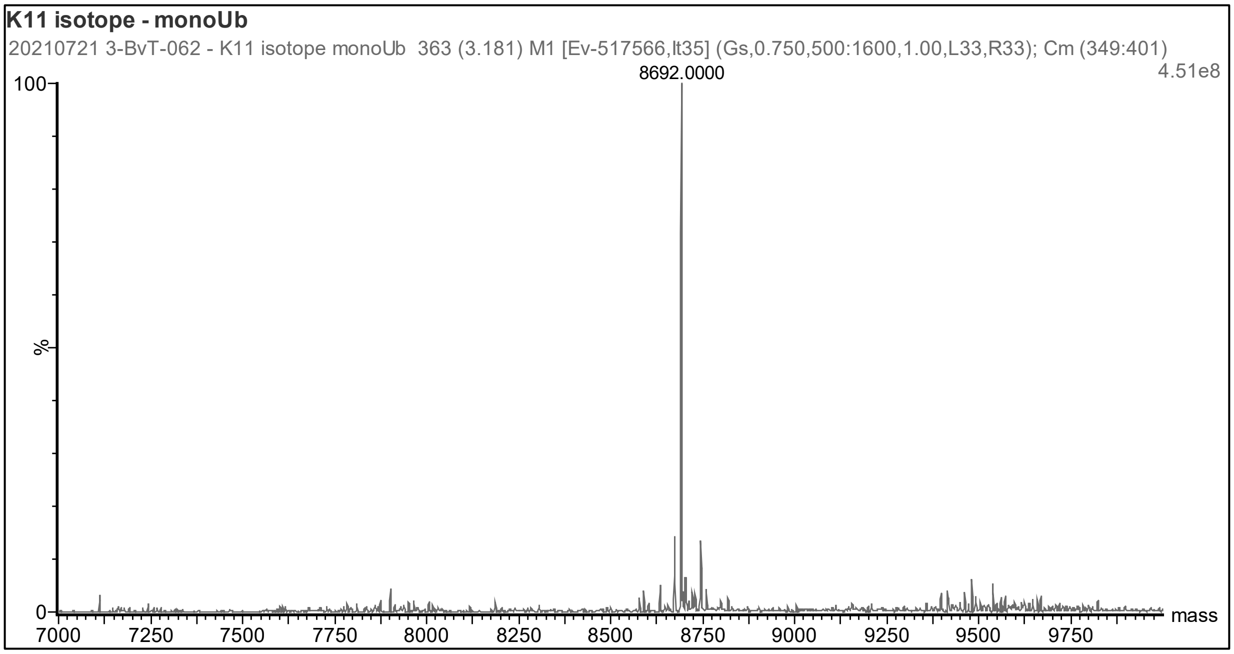

Neutron-encoded monoUb K27 TLys(1c) **System 2- Gradient 3**

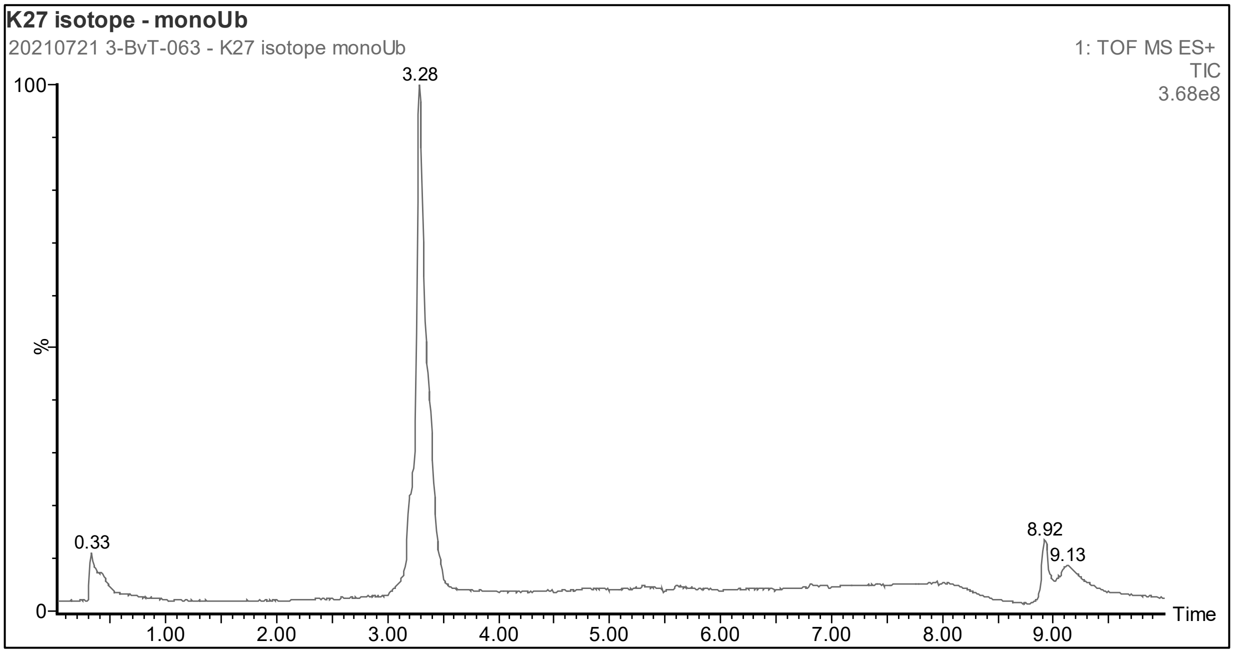

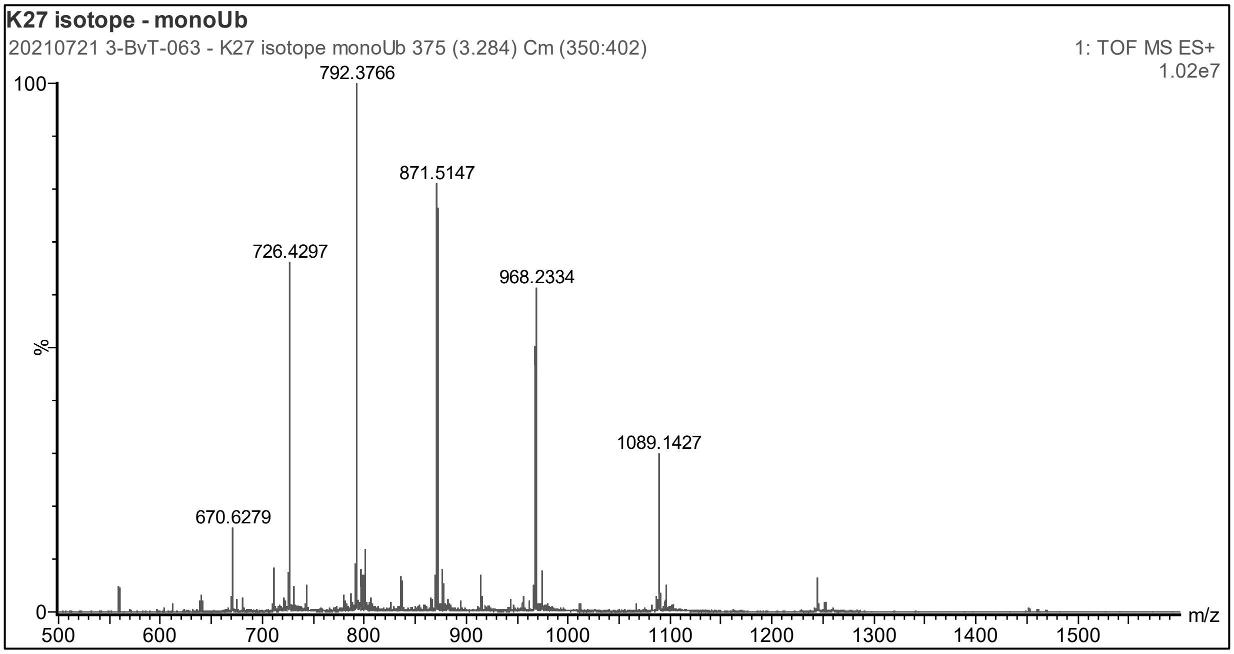

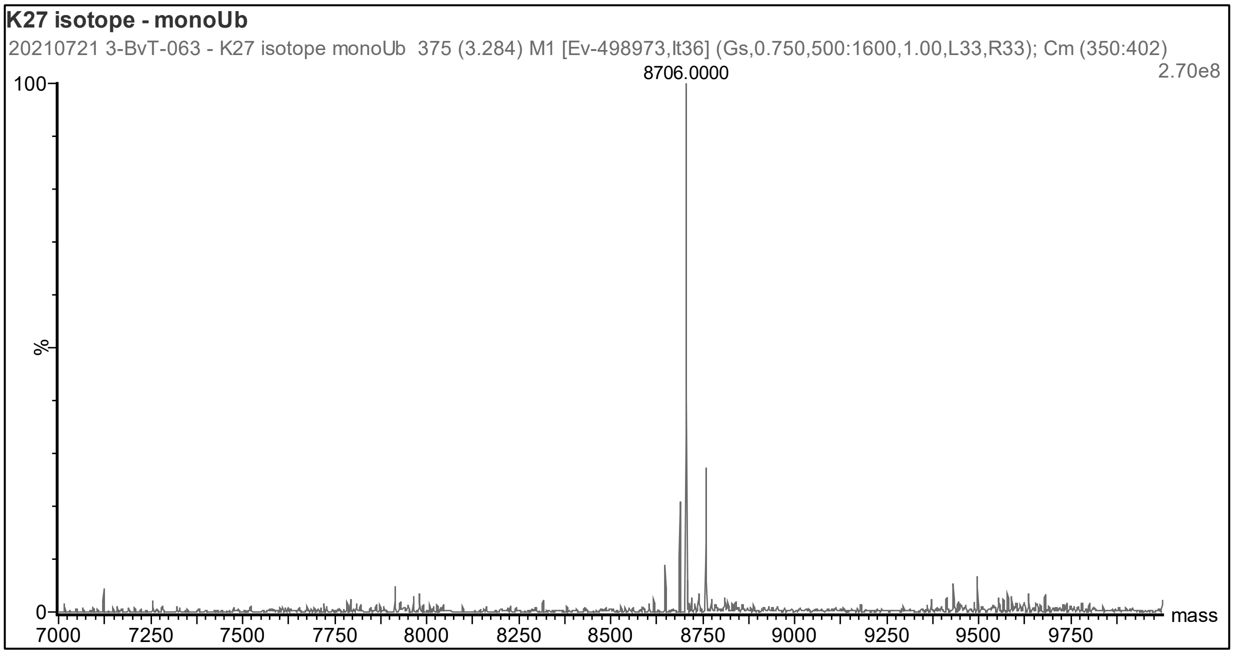

Neutron-encoded monoUb K29 TLys(1d) **System 2- Gradient 3**

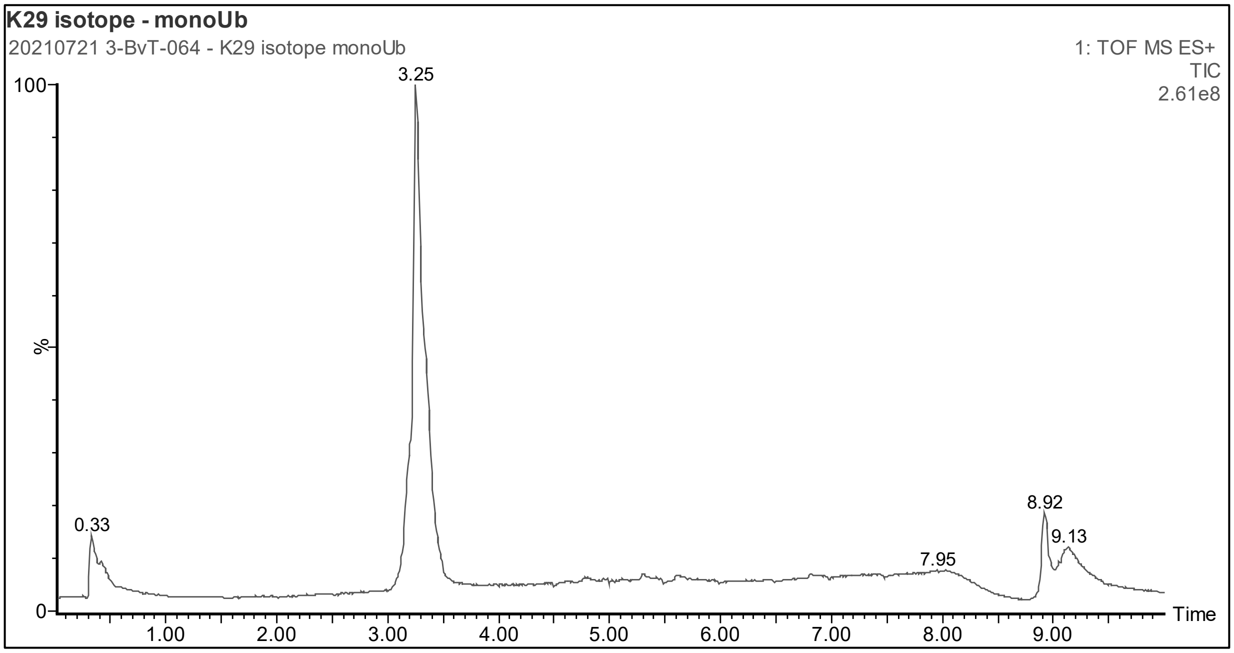

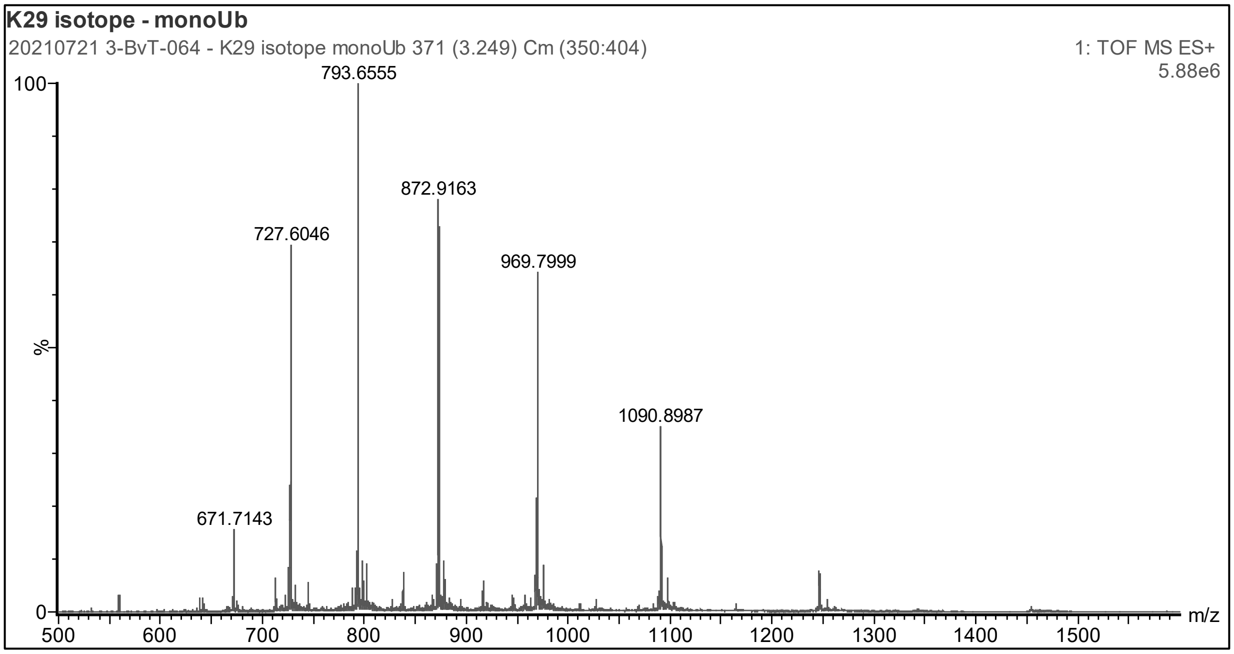

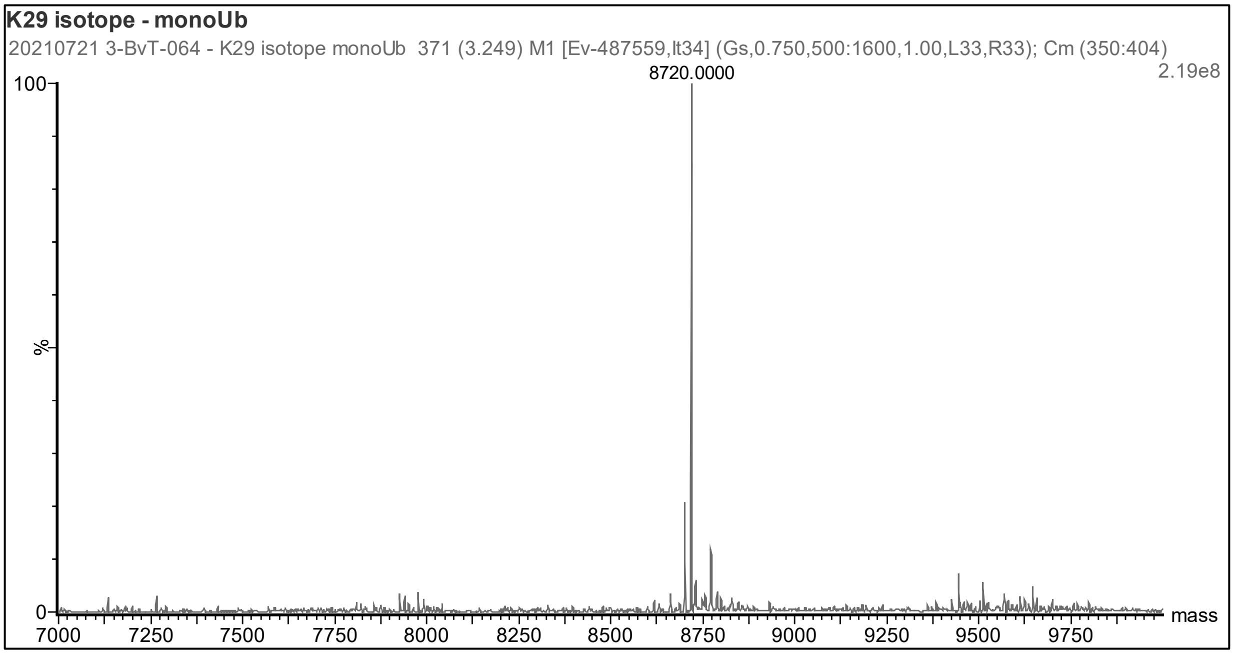

Neutron-encoded monoUb K33 TLys(1e) **System 2- Gradient 3**

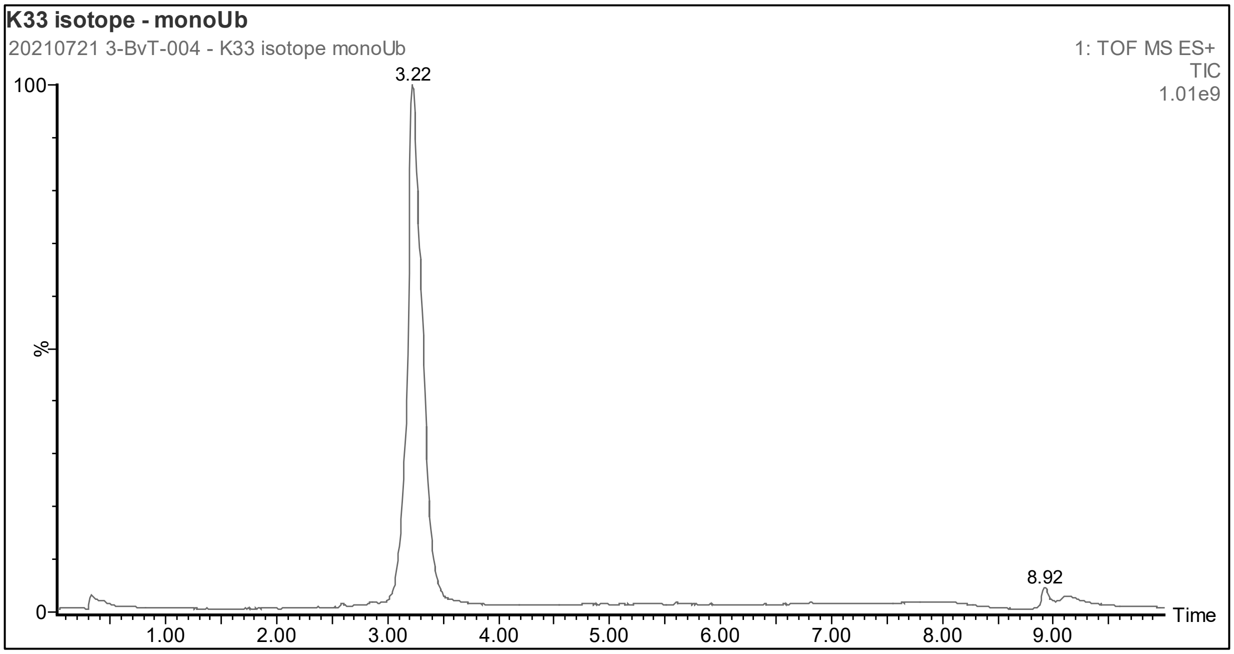

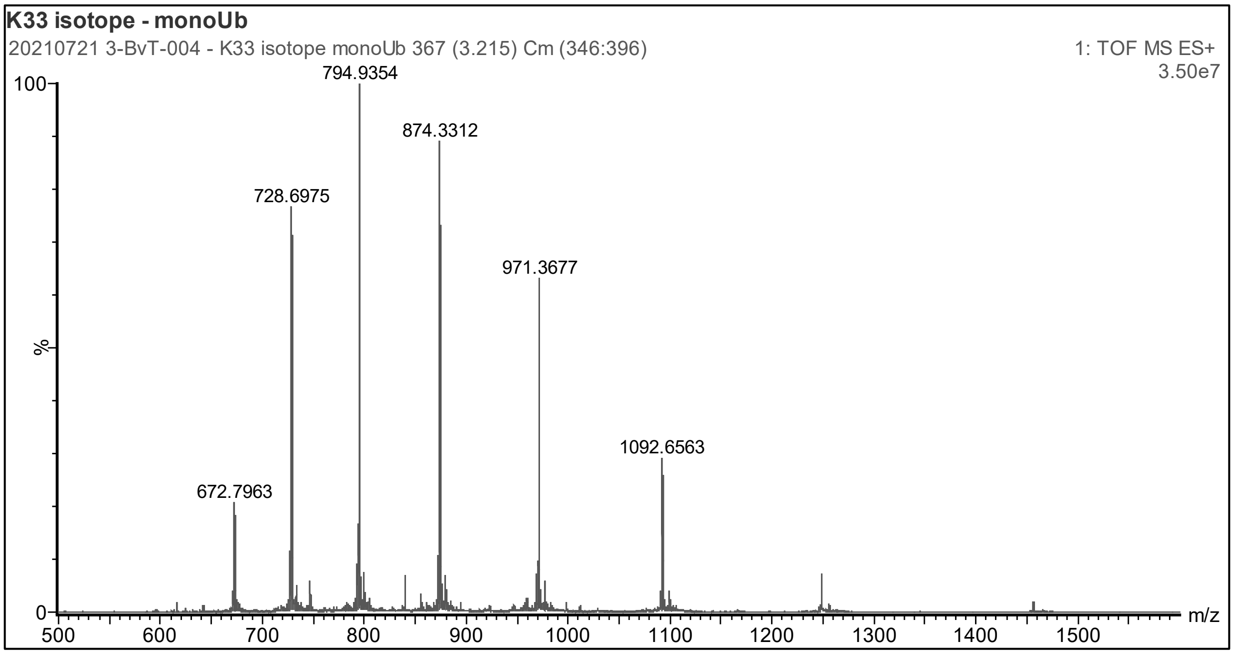

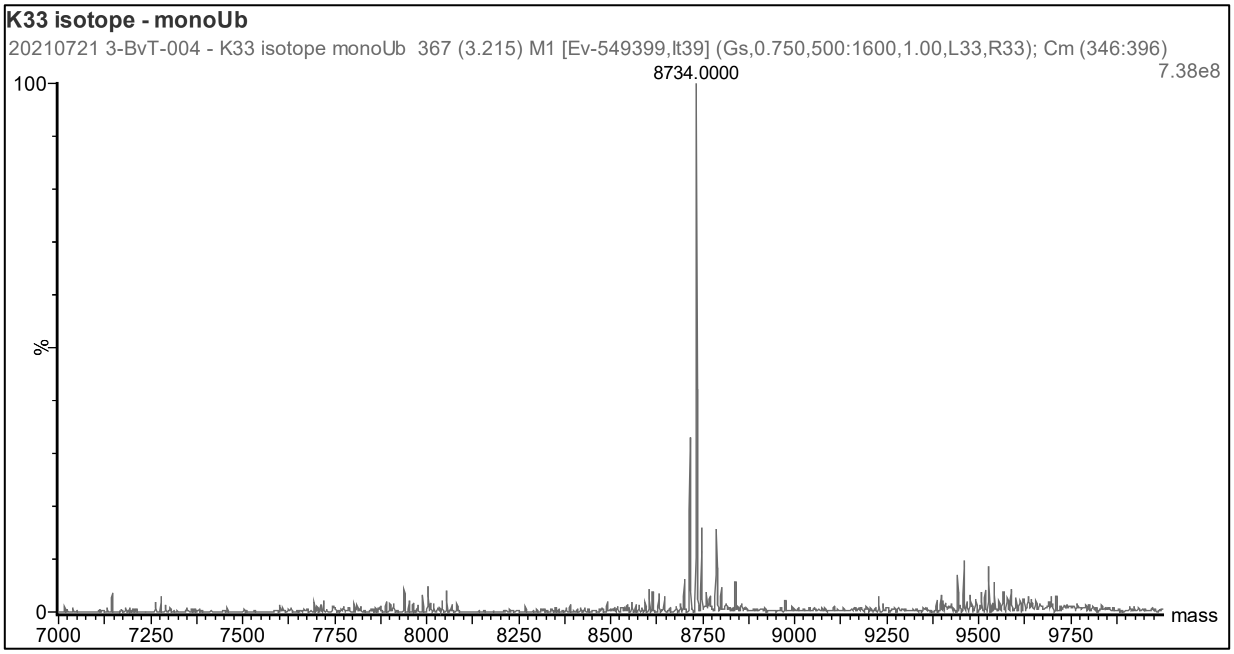

Neutron-encoded monoUb K48 TLys(1f) **System 2- Gradient 3**

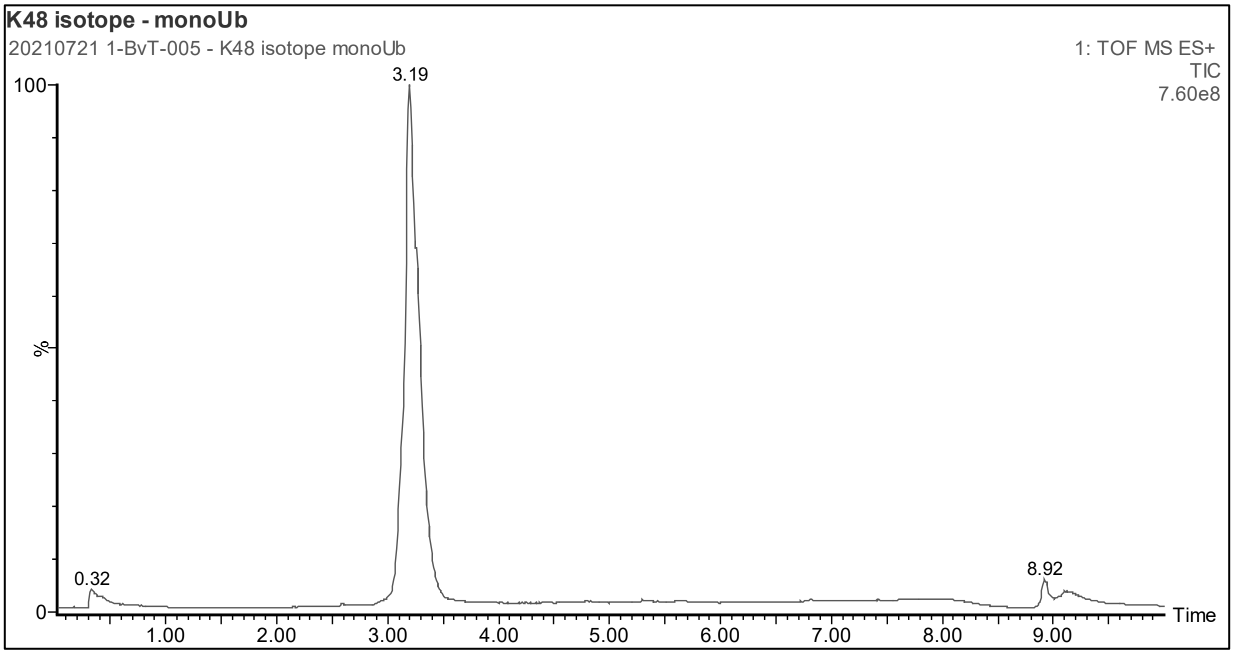

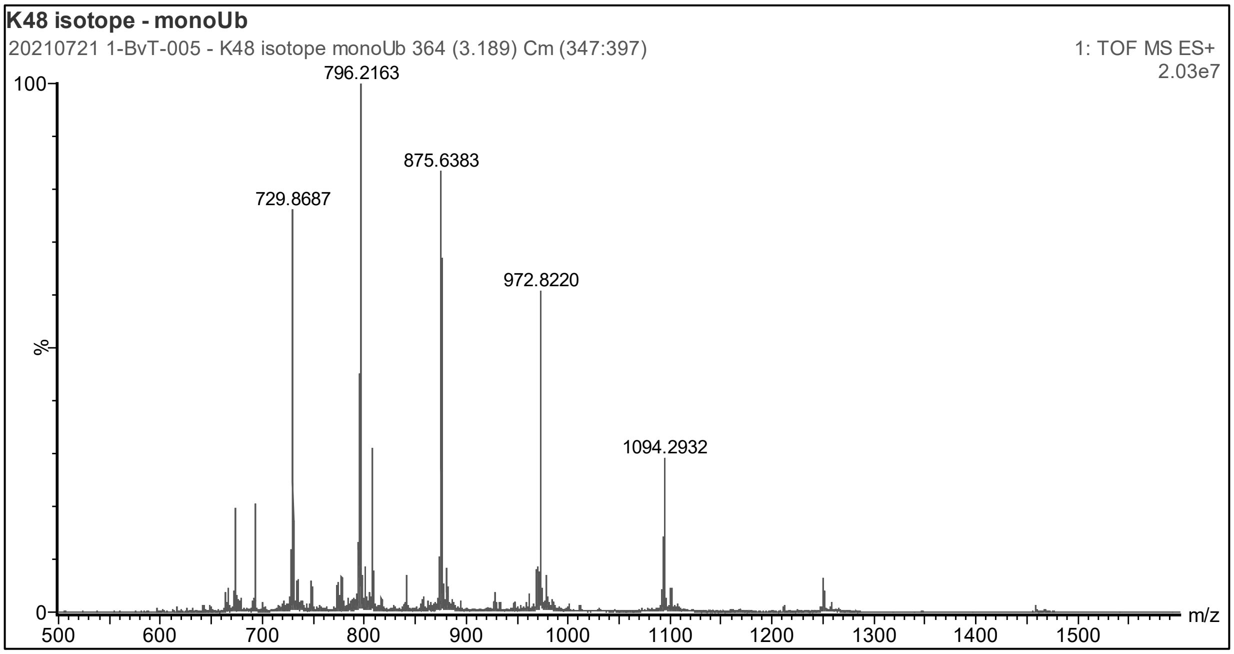

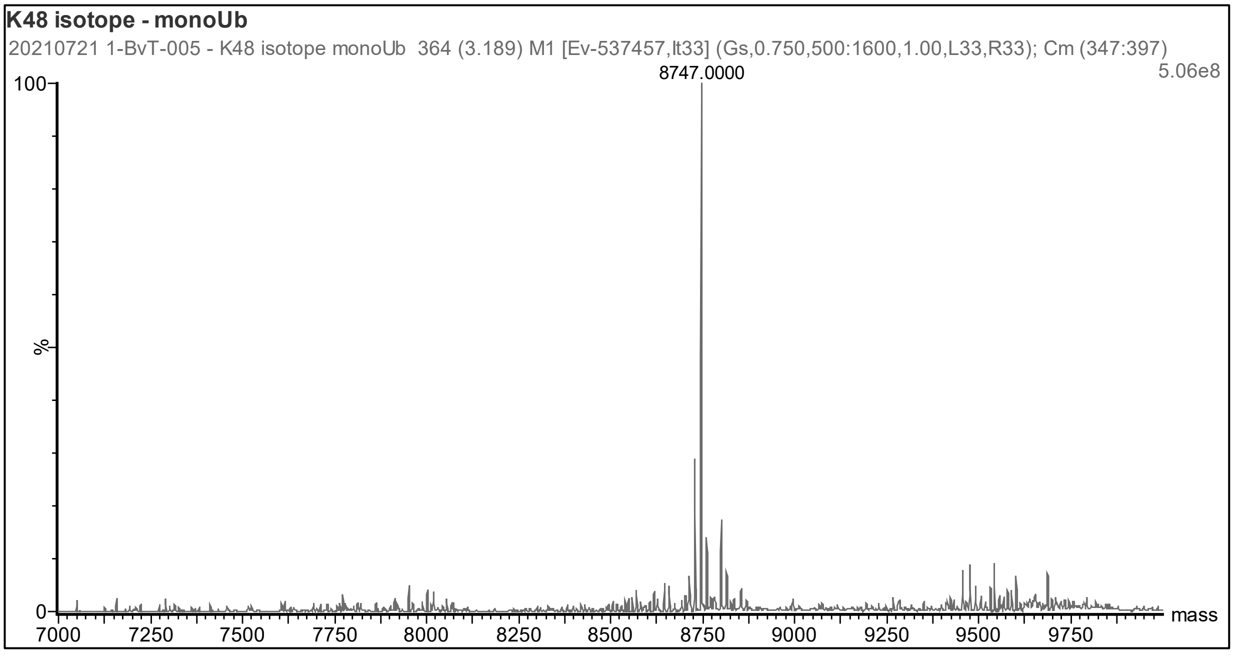

Neutron-encoded monoUb K63 TLys(1g) **System 2- Gradient 3**

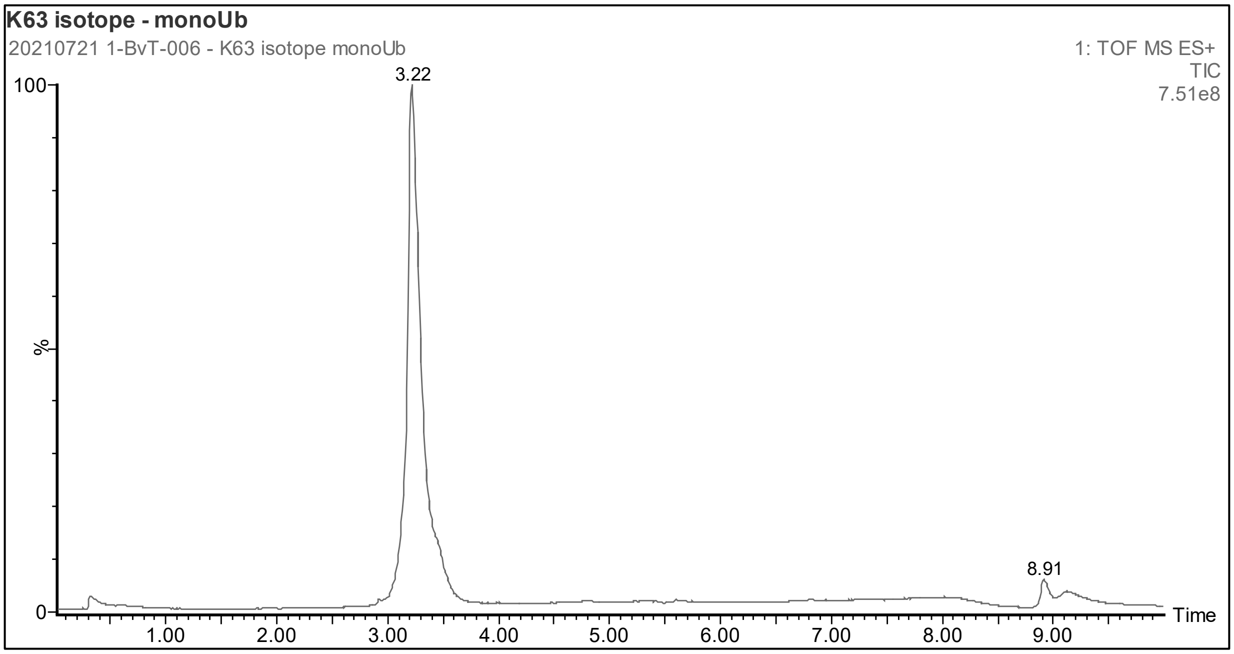

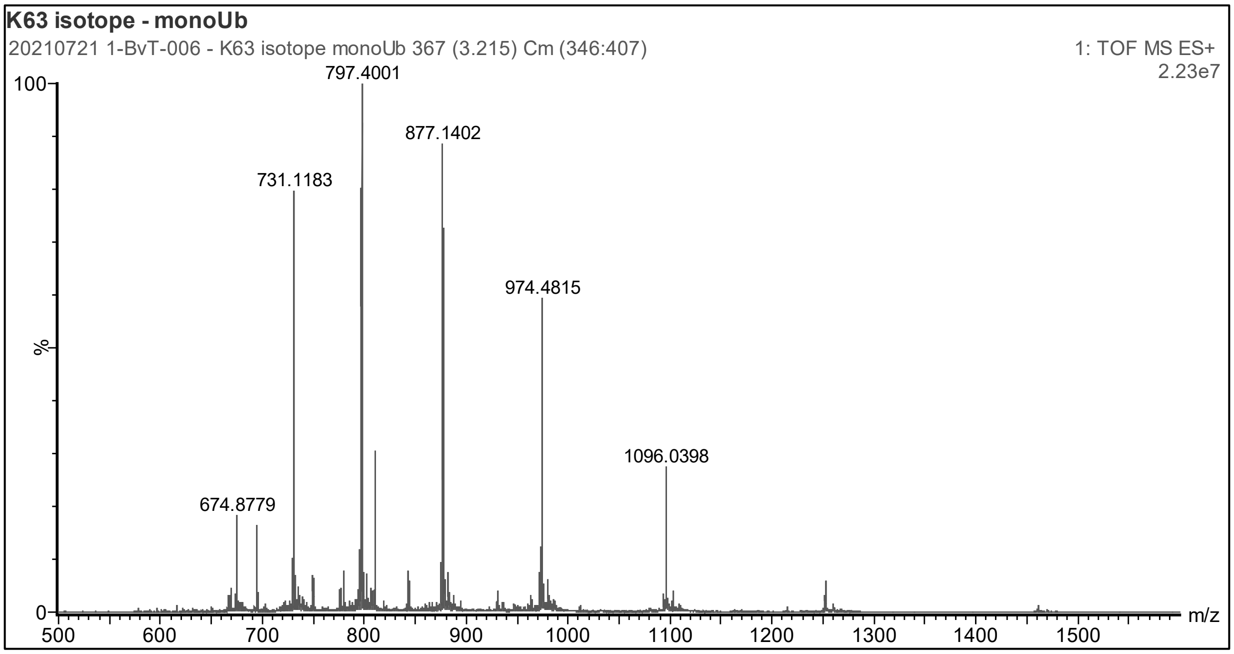

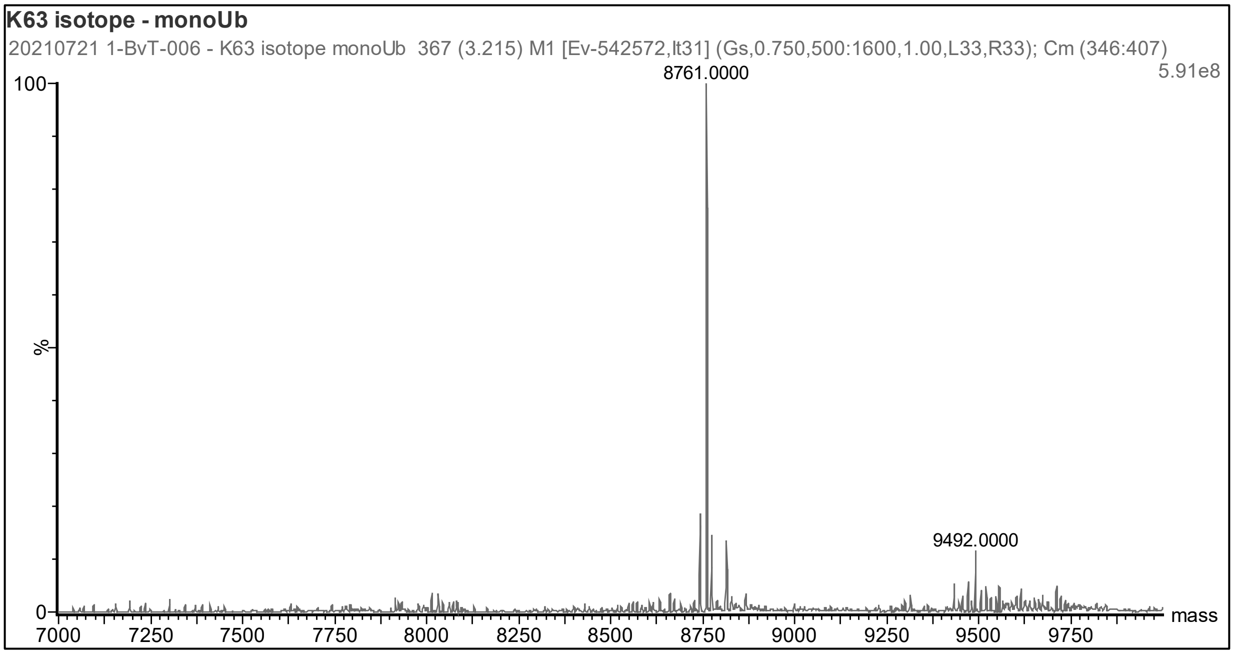

Neutron-encoded monoUb M1 ThioNle(2) **System 2- Gradient 3**

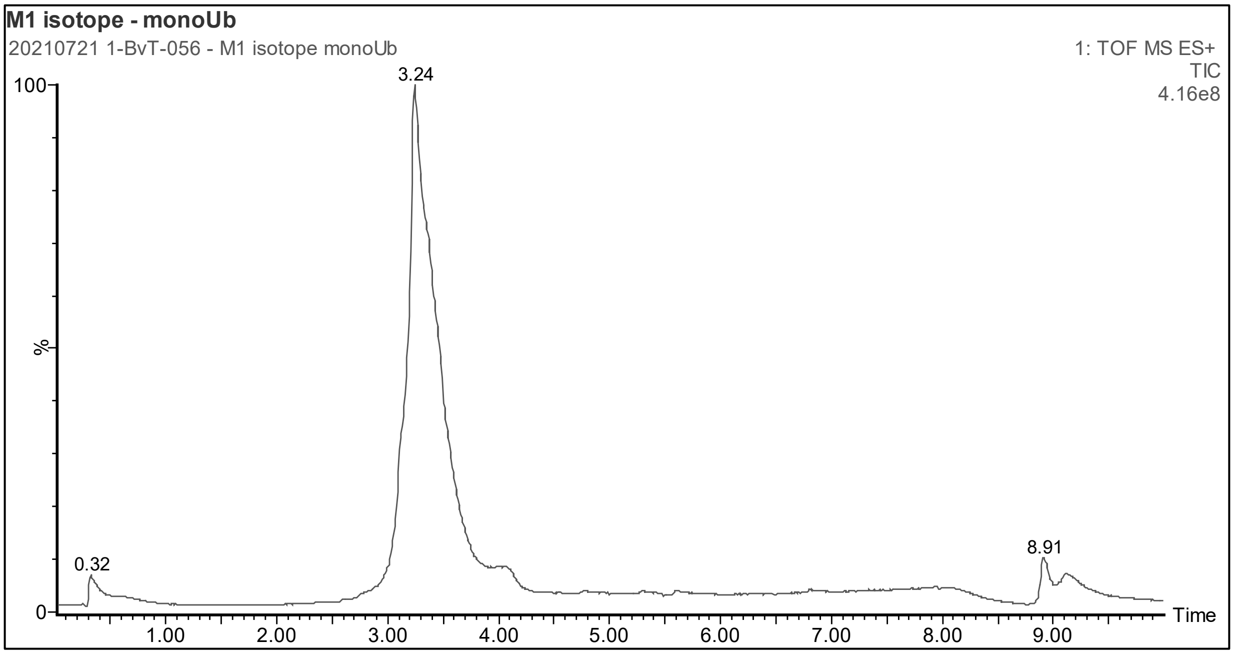

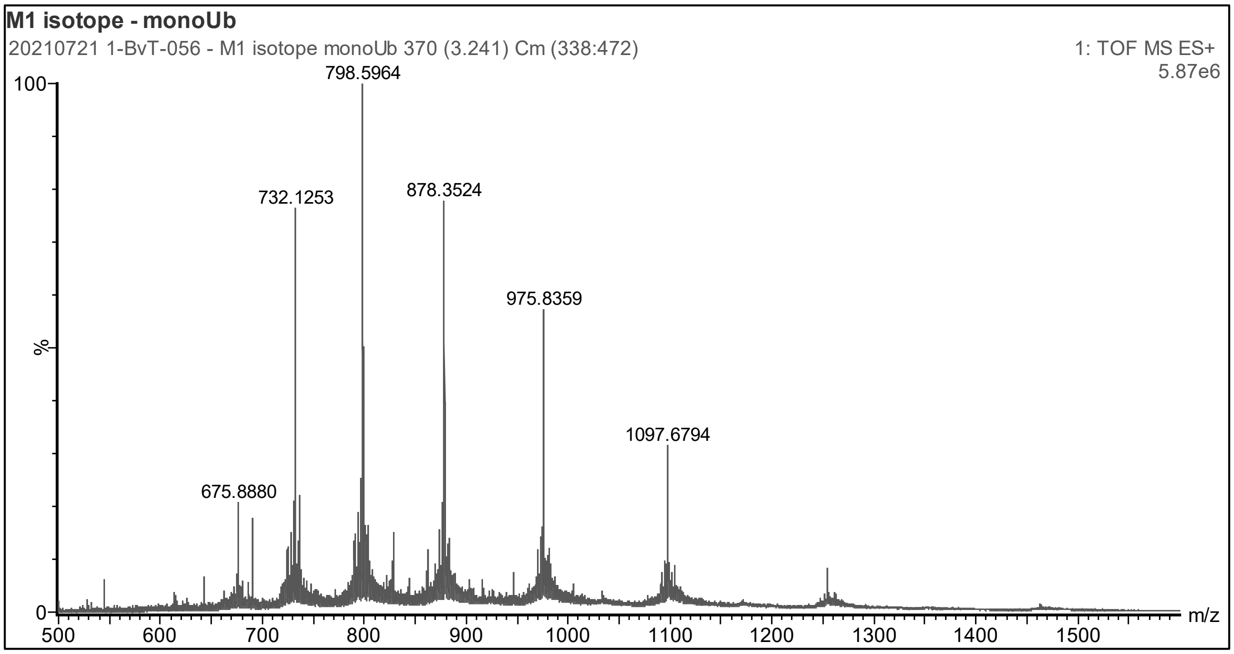

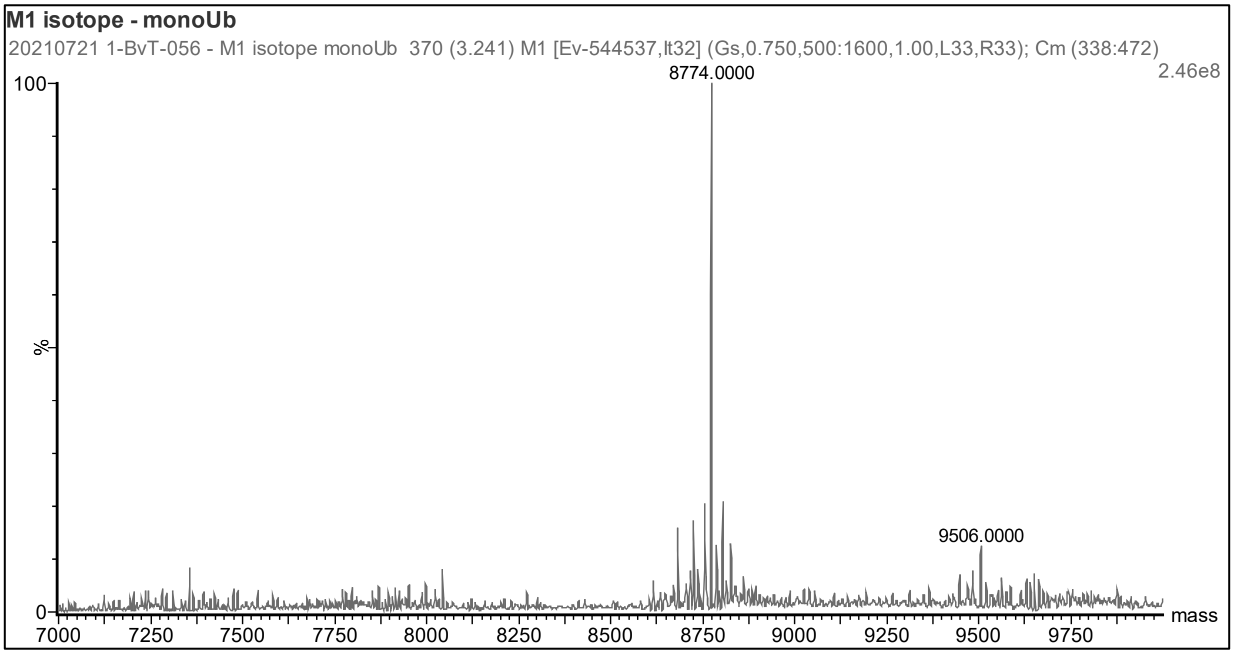

Ub(1-76, Nle$_1$)-S(CH$_2$)$_2$CO$_2$Me (3) **System 2- Gradient 3**

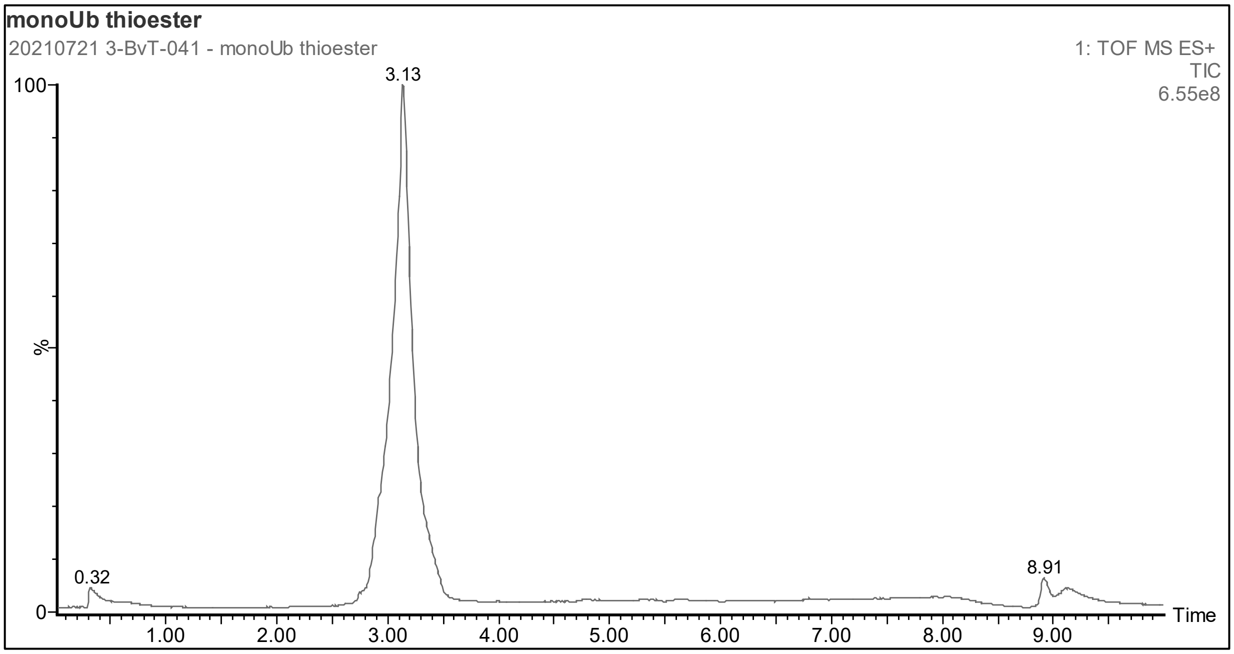

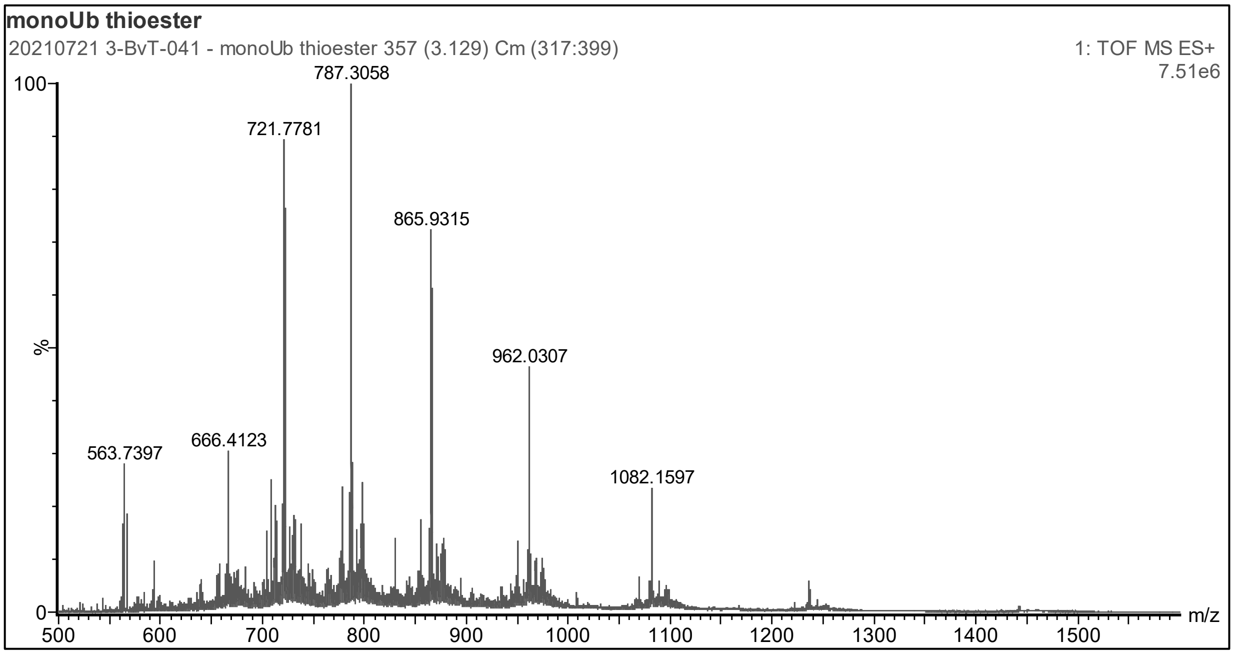

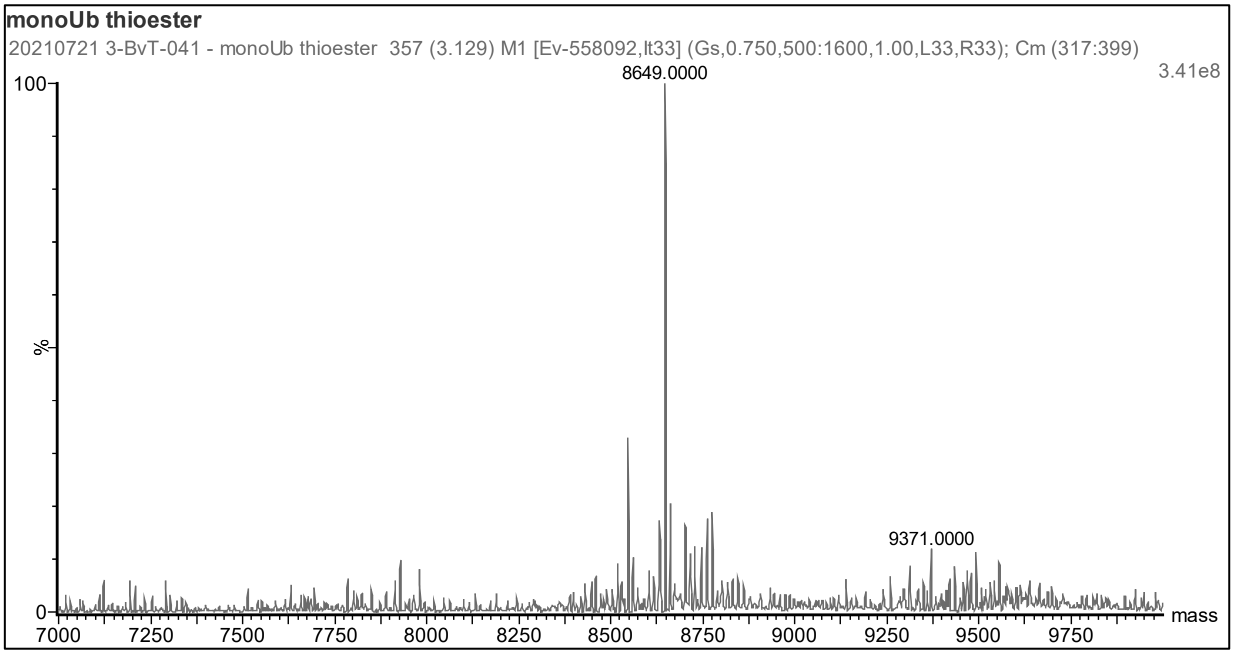

Supplement: Supplementary file 4 — Supplementary Data 1 [file 41467_2023_37363_MOESM4_ESM.pdf]
