# Supplementary material for: Neutron-encoded diubiquitins to profile linkage selectivity of deubiquitinating enzymes
Source: Nat Commun. 2023 Mar 25;14:1661. doi: 10.1038/s41467-023-37363-6 (PMC10039891; doi:10.1038/s41467-023-37363-6)
Supplement: Supplementary file 5 — Supplementary Data 2 [file 41467_2023_37363_MOESM5_ESM.pdf]

Supplementary Data 2. Determination of the linkage specificities of 22 DUBs. The quantified assay results of the DUBs belonging to **a,** the UbiCRest DUBs **b,** literature USPs and **c,** different DUB families DUBs are shown in two or three different graphs. The first graph shows the remaining % of diUb normalized to the internal standard non-hydrolysable Lys48 linked diUb and calculated starting from 100% remaining at t=0. The second graph shows the amount of monoUb formed (µM), normalized to the internal standard Ub_{1-74} and calculated using the concentration of the internal standard as reference. The third graph is a zoom-in or zoom-out of panel two, for clarification purposes (n=1).

a, UbiCRest DUBs

OTUB1

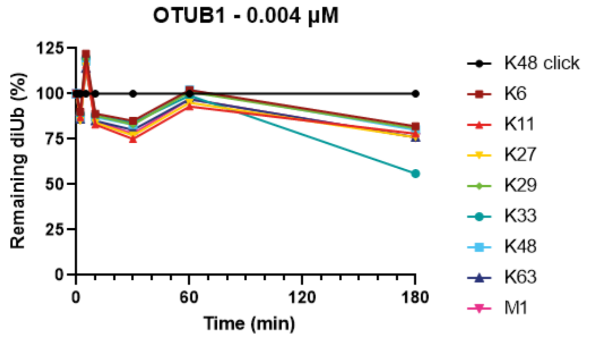

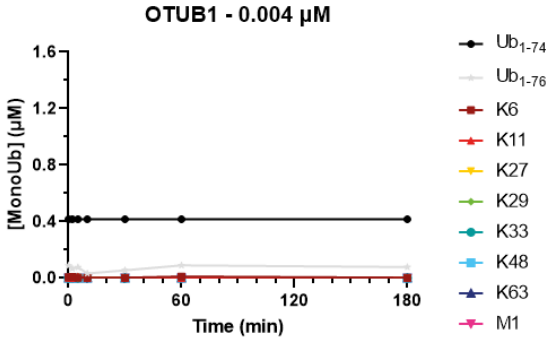

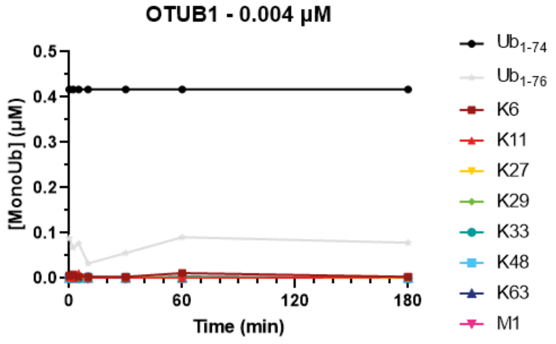

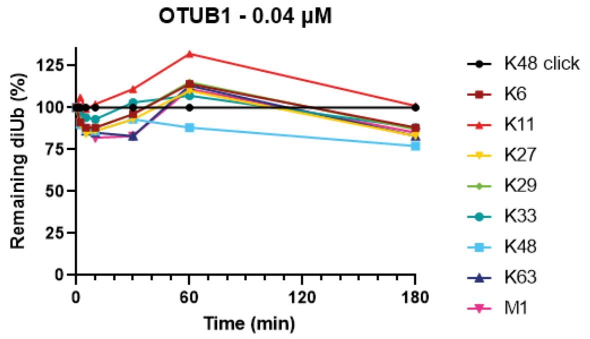

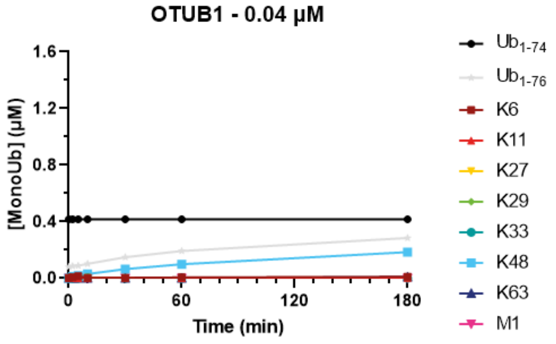

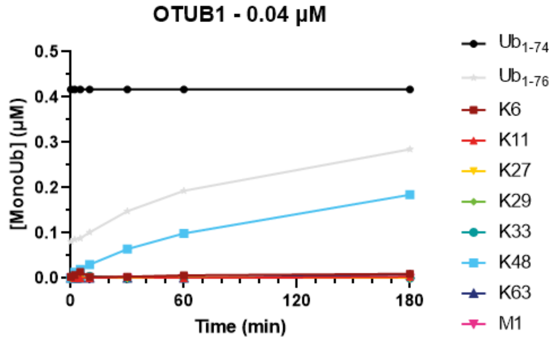

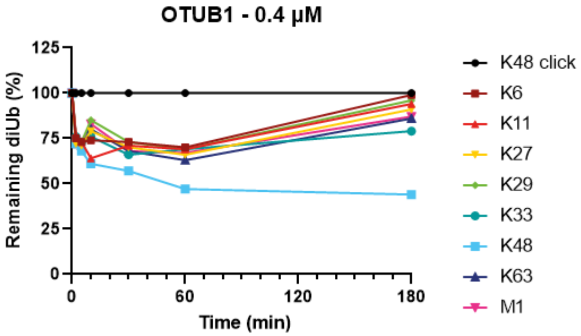
OTUB1 - 0.4 µM
Remaining diUb (%)
Time (min)
K48 click
K6
K11
K27
K29
K33
K48
K63
M1

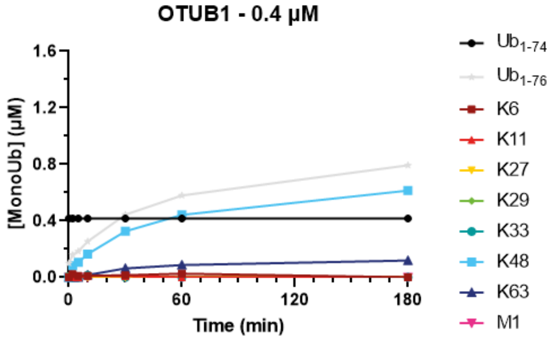
OTUB1 - 0.4 µM
[MonoUb] (µM)
Time (min)
Ub_{1-74}
Ub_{1-76}
K6
K11
K27
K29
K33
K48
K63
M1

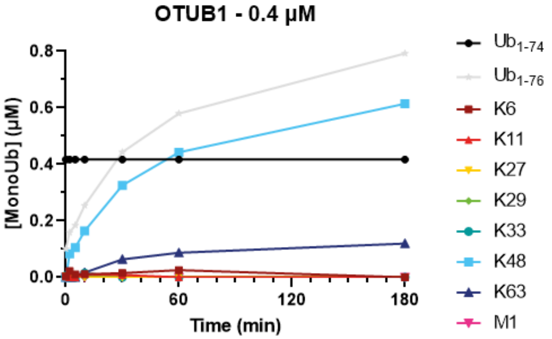
OTUB1 - 0.4 µM
[MonoUb] (µM)
Time (min)
Ub_{1-74}
Ub_{1-76}
K6
K11
K27
K29
K33
K48
K63
M1

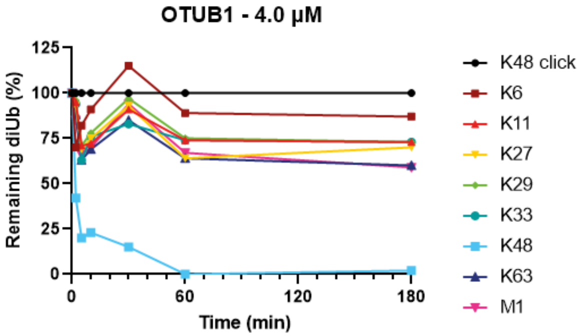
OTUB1 - 4.0 µM
Remaining diUb (%)
Time (min)
K48 click
K6
K11
K27
K29
K33
K48
K63
M1

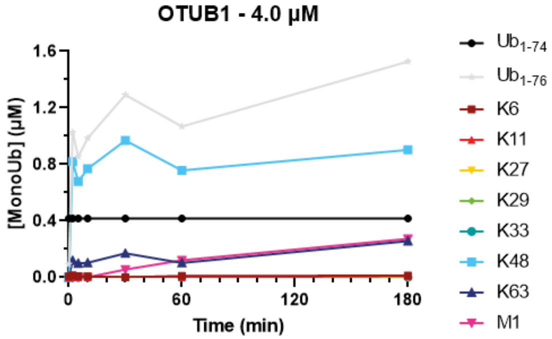
OTUB1 - 4.0 µM
[MonoUb] (µM)
Time (min)
Ub_{1-74}
Ub_{1-76}
K6
K11
K27
K29
K33
K48
K63
M1

OTUB2

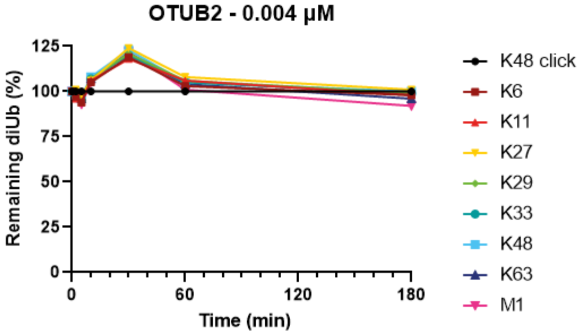
OTUB2 - 0.004 µM
Remaining diUb (%)
Time (min)
K48 click
K6
K11
K27
K29
K33
K48
K63
M1

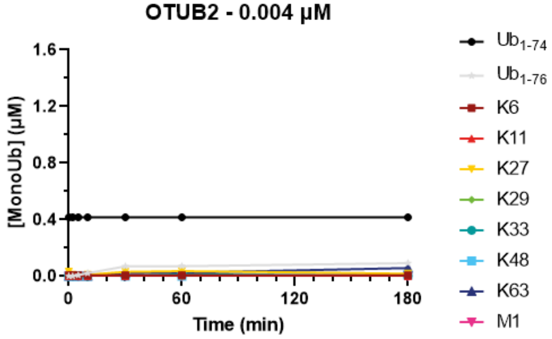
OTUB2 - 0.004 µM
[MonoUb] (µM)
Time (min)
Ub_{1-74}
Ub_{1-76}
K6
K11
K27
K29
K33
K48
K63
M1

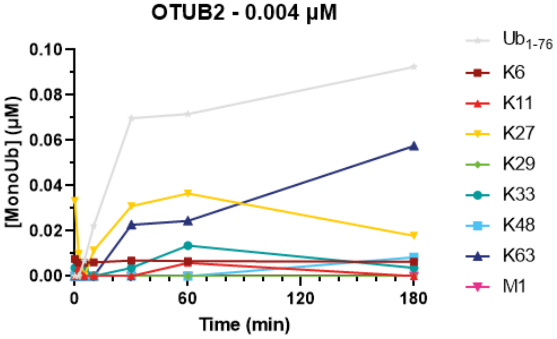
OTUB2 - 0.004 µM
[MonoUb] (µM)
Time (min)
Ub_{1-76}
K6
K11
K27
K29
K33
K48
K63
M1

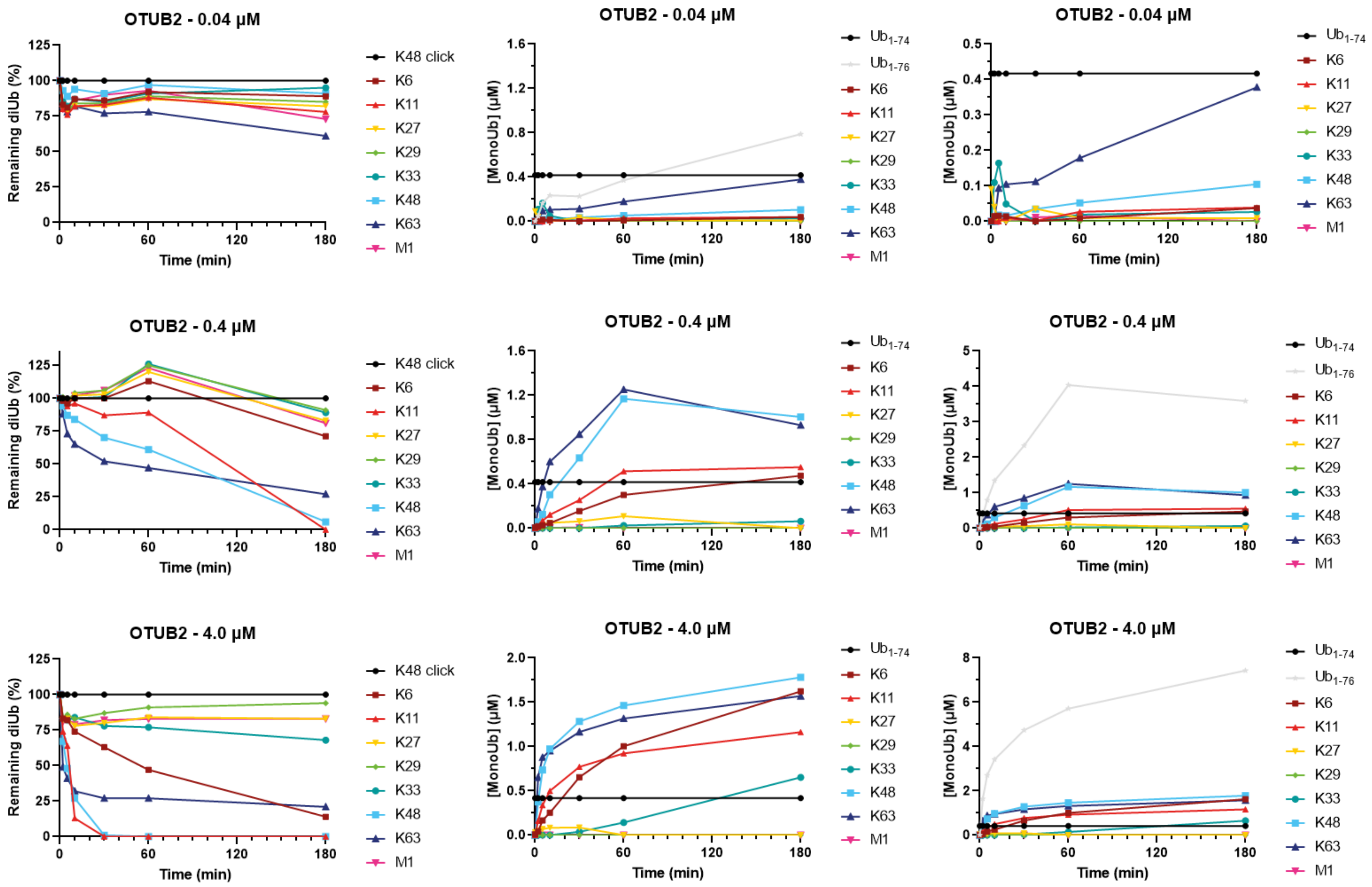
OTUB2 - 0.04 µM
Remaining diUb (%)
Time (min)
K48 click
K6
K11
K27
K29
K33
K48
K63
M1
OTUB2 - 0.04 µM
[MonoUb] (µM)
Time (min)
Ub1-74
Ub1-76
K6
K11
K27
K29
K33
K48
K63
M1
OTUB2 - 0.04 µM
[MonoUb] (µM)
Time (min)
Ub1-74
K6
K11
K27
K29
K33
K48
K63
M1
OTUB2 - 0.4 µM
Remaining diUb (%)
Time (min)
K48 click
K6
K11
K27
K29
K33
K48
K63
M1
OTUB2 - 0.4 µM
[MonoUb] (µM)
Time (min)
Ub1-74
K6
K11
K27
K29
K33
K48
K63
M1
OTUB2 - 0.4 µM
[MonoUb] (µM)
Time (min)
Ub1-74
Ub1-76
K6
K11
K27
K29
K33
K48
K63
M1
OTUB2 - 4.0 µM
Remaining diUb (%)
Time (min)
K48 click
K6
K11
K27
K29
K33
K48
K63
M1
OTUB2 - 4.0 µM
[MonoUb] (µM)
Time (min)
Ub1-74
K6
K11
K27
K29
K33
K48
K63
M1
OTUB2 - 4.0 µM
[MonoUb] (µM)
Time (min)
Ub1-74
Ub1-76
K6
K11
K27
K29
K33
K48
K63
M1

OTUD2

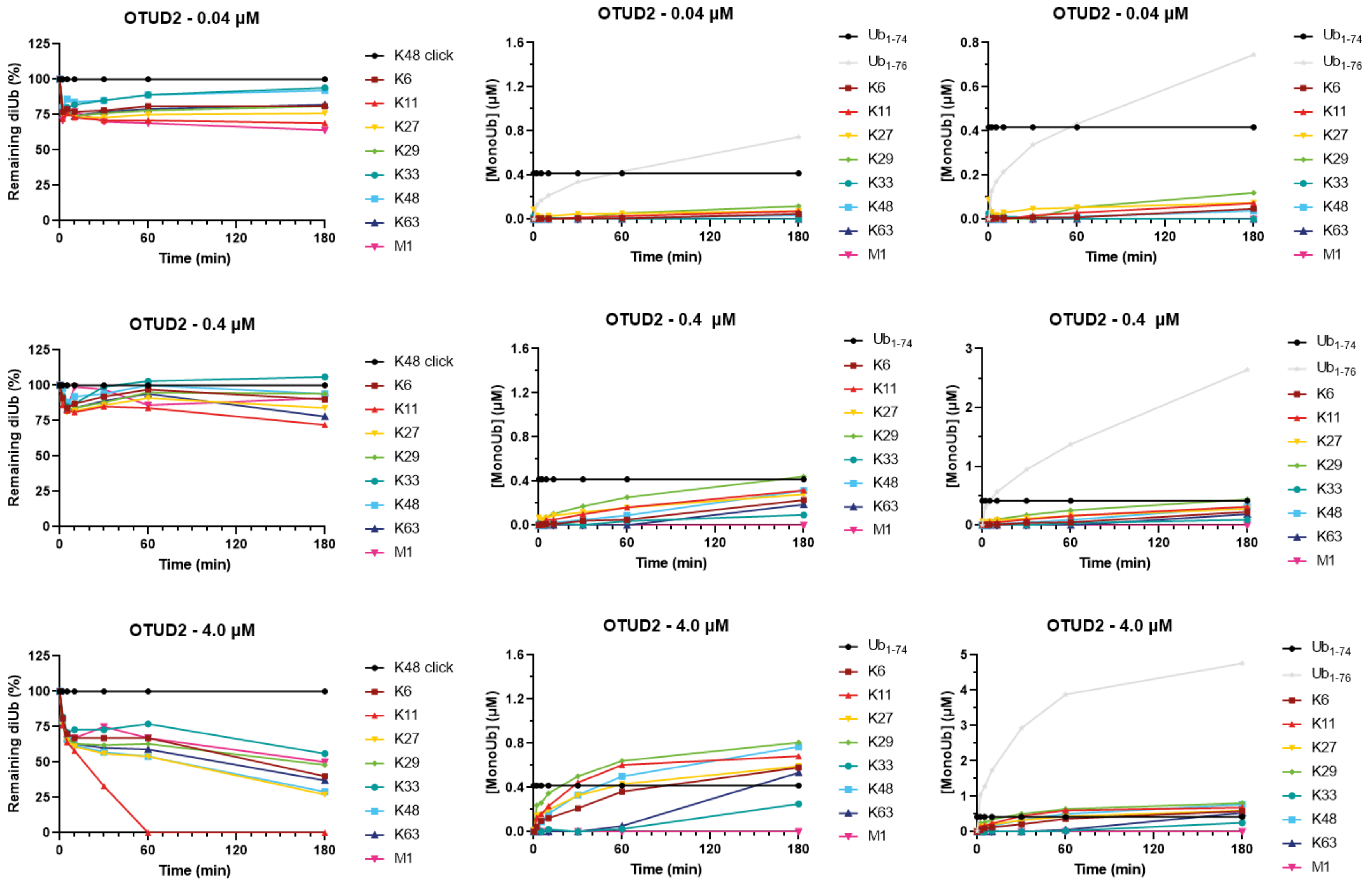
OTUD2 - 0.04 µM
Remaining diUb (%)
Time (min)
K48 click
K6
K11
K27
K29
K33
K48
K63
M1
OTUD2 - 0.04 µM
[MonoUb] (µM)
Time (min)
Ub_{1-74}
Ub_{1-76}
K6
K11
K27
K29
K33
K48
K63
M1
OTUD2 - 0.04 µM
[MonoUb] (µM)
Time (min)
Ub_{1-74}
Ub_{1-76}
K6
K11
K27
K29
K33
K48
K63
M1
OTUD2 - 0.4 µM
Remaining diUb (%)
Time (min)
K48 click
K6
K11
K27
K29
K33
K48
K63
M1
OTUD2 - 0.4 µM
[MonoUb] (µM)
Time (min)
Ub_{1-74}
K6
K11
K27
K29
K33
K48
K63
M1
OTUD2 - 0.4 µM
[MonoUb] (µM)
Time (min)
Ub_{1-74}
Ub_{1-76}
K6
K11
K27
K29
K33
K48
K63
M1
OTUD2 - 4.0 µM
Remaining diUb (%)
Time (min)
K48 click
K6
K11
K27
K29
K33
K48
K63
M1
OTUD2 - 4.0 µM
[MonoUb] (µM)
Time (min)
Ub_{1-74}
K6
K11
K27
K29
K33
K48
K63
M1
OTUD2 - 4.0 µM
[MonoUb] (µM)
Time (min)
Ub_{1-74}
Ub_{1-76}
K6
K11
K27
K29
K33
K48
K63
M1

OTUD3

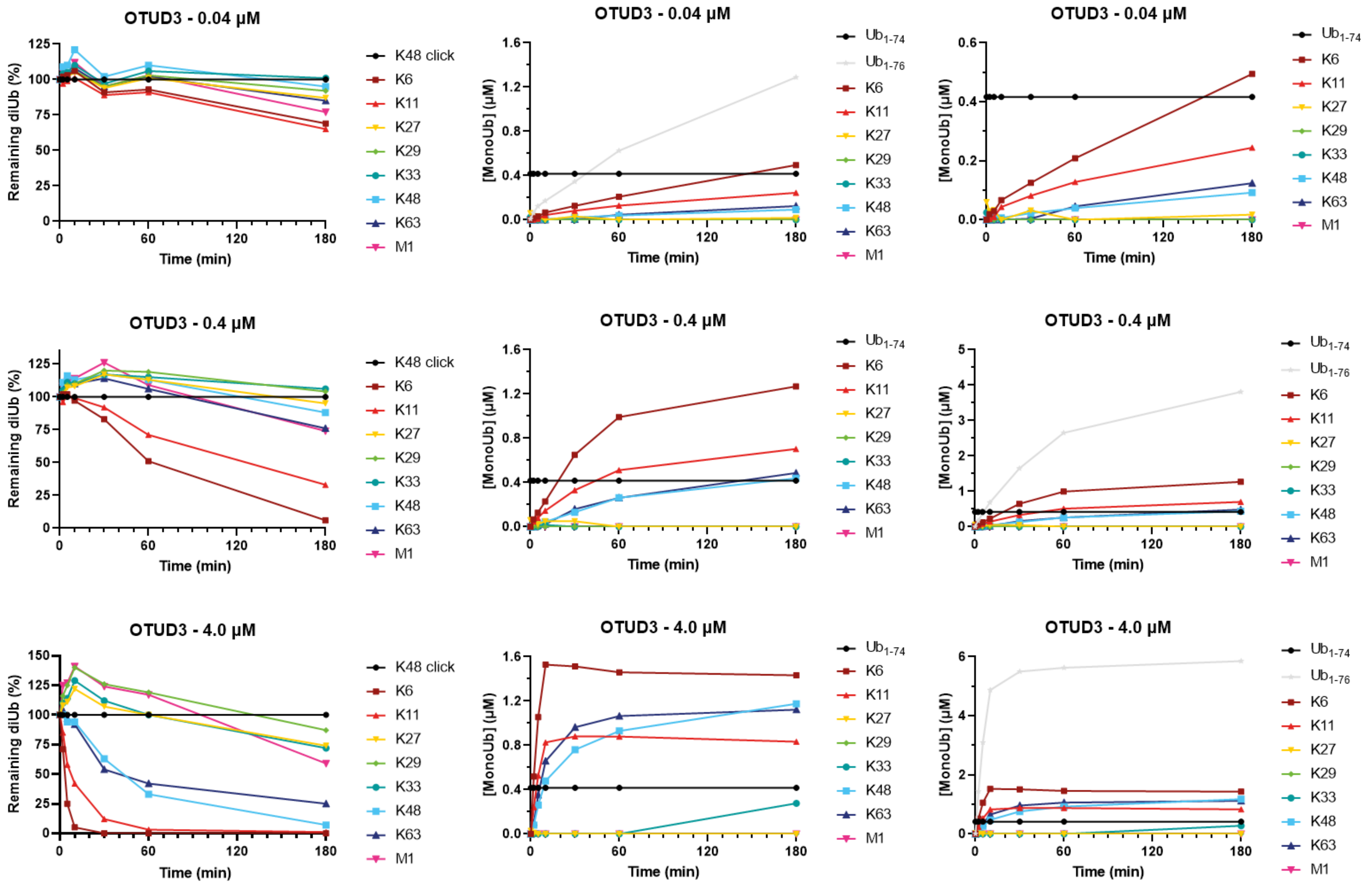

OTUD3 - 0.04 μM
Remaining diUb (%)
Time (min)
K48 click
K6
K11
K27
K29
K33
K48
K63
M1
OTUD3 - 0.04 μM
[MonoUb] (μM)
Time (min)
Ub1-74
Ub1-76
K6
K11
K27
K29
K33
K48
K63
M1
OTUD3 - 0.04 μM
[MonoUb] (μM)
Time (min)
Ub1-74
K6
K11
K27
K29
K33
K48
K63
M1
OTUD3 - 0.4 μM
Remaining diUb (%)
Time (min)
OTUD3 - 0.4 μM
[MonoUb] (μM)
Time (min)
Ub1-74
K6
K11
K27
K29
K33
K48
K63
M1
OTUD3 - 0.4 μM
[MonoUb] (μM)
Time (min)
Ub1-74
Ub1-76
OTUD3 - 4.0 μM
Remaining diUb (%)
Time (min)
OTUD3 - 4.0 μM
[MonoUb] (μM)
Time (min)
OTUD3 - 4.0 μM
[MonoUb] (μM)
Time (min)

OTULIN

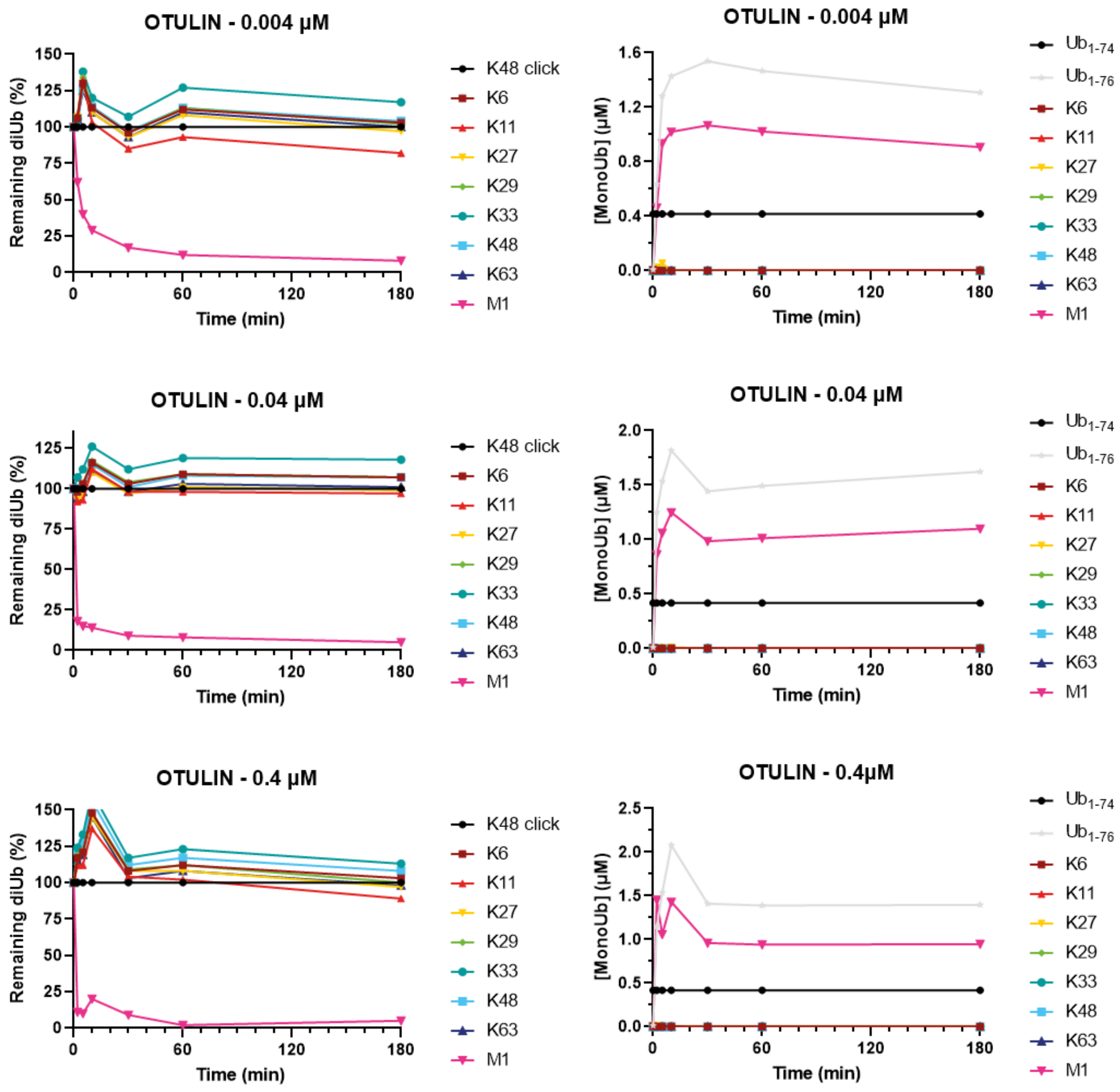
OTULIN - 0.004 µM
Remaining diUb (%)
Time (min)
OTULIN - 0.004 µM
[MonoUb] (µM)
Time (min)
OTULIN - 0.04 µM
Remaining diUb (%)
Time (min)
OTULIN - 0.04 µM
[MonoUb] (µM)
Time (min)
OTULIN - 0.4 µM
Remaining diUb (%)
Time (min)
OTULIN - 0.4µM
[MonoUb] (µM)
Time (min)
K48 click
K6
K11
K27
K29
K33
K48
K63
M1
Ub_{1-74}
Ub_{1-76}

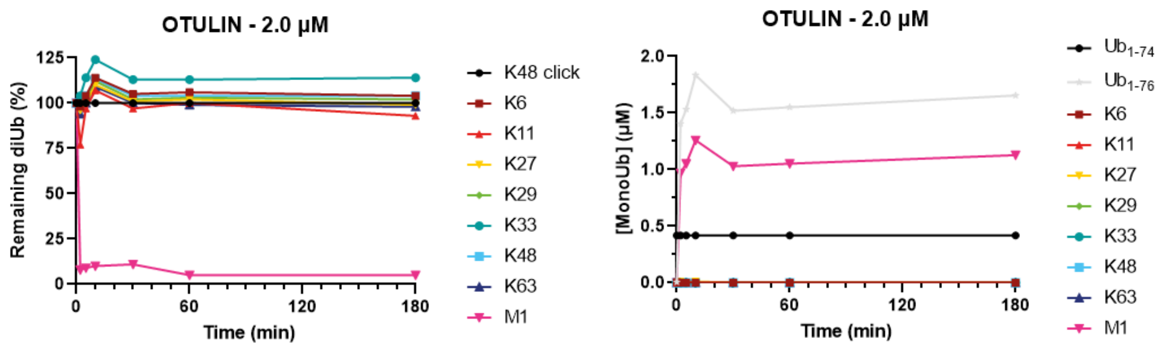
OTULIN - 2.0 µM
Remaining diUb (%)
Time (min)
K48 click
K6
K11
K27
K29
K33
K48
K63
M1
OTULIN - 2.0 µM
[MonoUb] (µM)
Time (min)
Ub1-74
Ub1-76
K6
K11
K27
K29
K33
K48
K63
M1

Cezanne

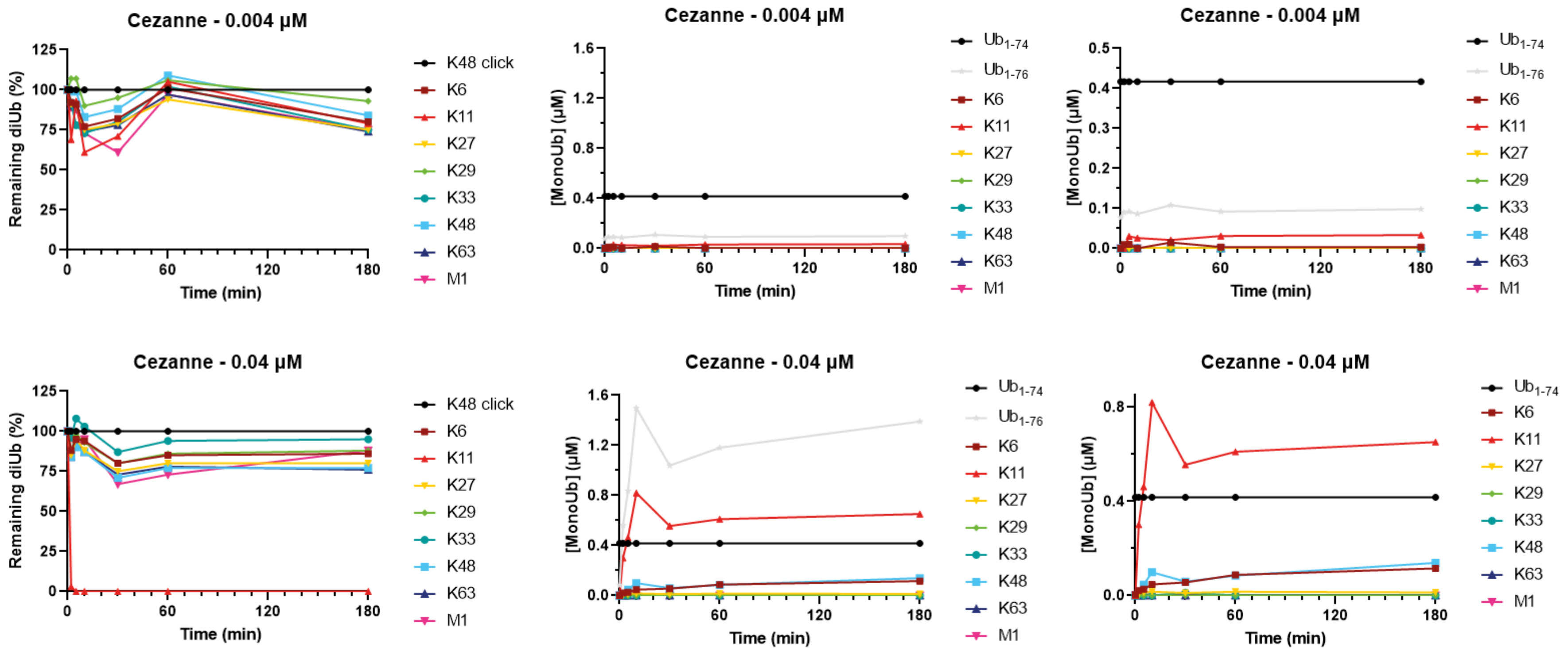
Cezanne - 0.004 µM
Remaining diUb (%)
Time (min)
K48 click
K6
K11
K27
K29
K33
K48
K63
M1
Cezanne - 0.004 µM
[MonoUb] (µM)
Time (min)
Ub1-74
Ub1-76
K6
K11
K27
K29
K33
K48
K63
M1
Cezanne - 0.004 µM
[MonoUb] (µM)
Time (min)
Cezanne - 0.04 µM
Remaining diUb (%)
Time (min)
Cezanne - 0.04 µM
[MonoUb] (µM)
Time (min)
Cezanne - 0.04 µM
[MonoUb] (µM)
Time (min)

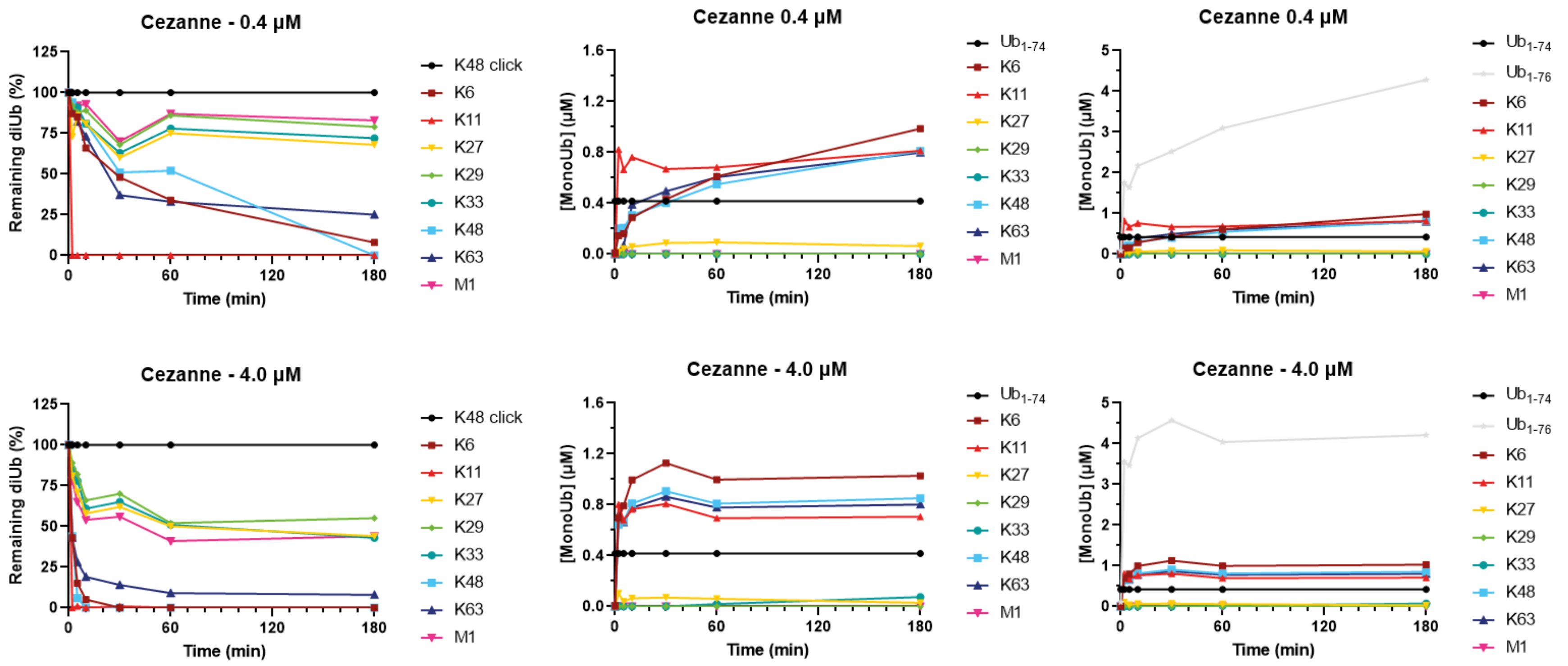
Cezanne - 0.4 µM
Remaining diUb (%)
Time (min)
K48 click
K6
K11
K27
K29
K33
K48
K63
M1
Cezanne 0.4 µM
[MonoUb] (µM)
Time (min)
Ub1-74
K6
K11
K27
K29
K33
K48
K63
M1
Cezanne 0.4 µM
[MonoUb] (µM)
Time (min)
Ub1-74
Ub1-76
K6
K11
K27
K29
K33
K48
K63
M1
Cezanne - 4.0 µM
Remaining diUb (%)
Time (min)
K48 click
K6
K11
K27
K29
K33
K48
K63
M1
Cezanne - 4.0 µM
[MonoUb] (µM)
Time (min)
Ub1-74
K6
K11
K27
K29
K33
K48
K63
M1
Cezanne - 4.0 µM
[MonoUb] (µM)
Time (min)
Ub1-74
Ub1-76
K6
K11
K27
K29
K33
K48
K63
M1

USP21

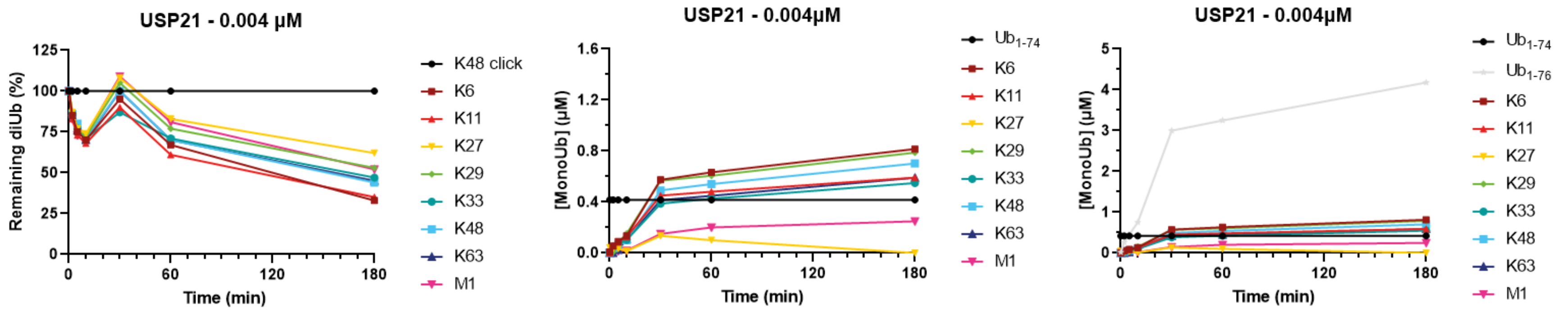
USP21 - 0.004 µM
Remaining diUb (%)
Time (min)
K48 click
K6
K11
K27
K29
K33
K48
K63
M1
USP21 - 0.004µM
[MonoUb] (µM)
Time (min)
Ub1-74
K6
K11
K27
K29
K33
K48
K63
M1
USP21 - 0.004µM
[MonoUb] (µM)
Time (min)
Ub1-74
Ub1-76
K6
K11
K27
K29
K33
K48
K63
M1

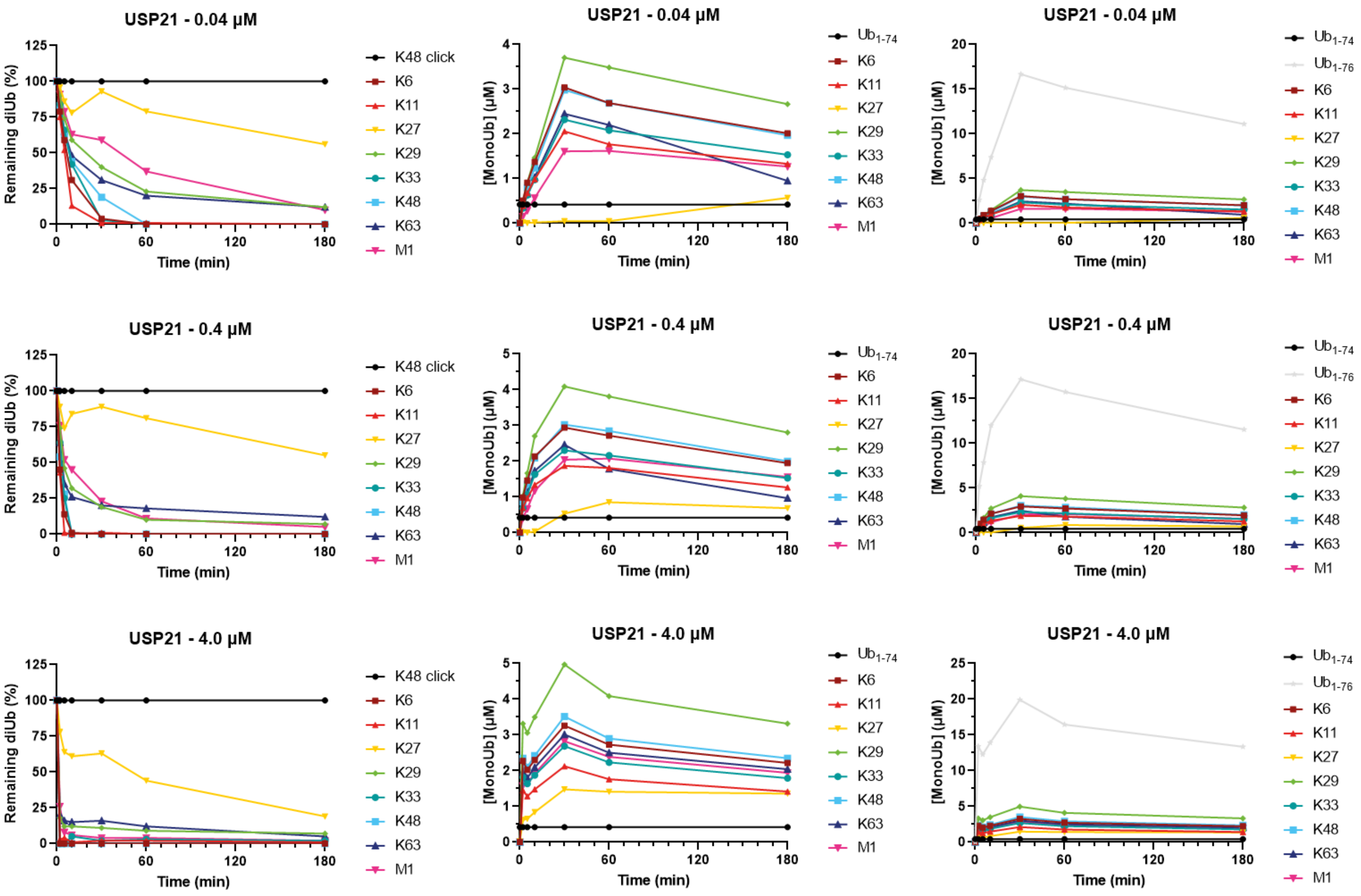

USP21 - 0.04 μM
Remaining diUb (%)
Time (min)
K48 click
K6
K11
K27
K29
K33
K48
K63
M1
[MonoUb] (μM)
Ub1-74
Ub1-76
USP21 - 0.4 μM
USP21 - 4.0 μM

b, Literature USPs

USP2

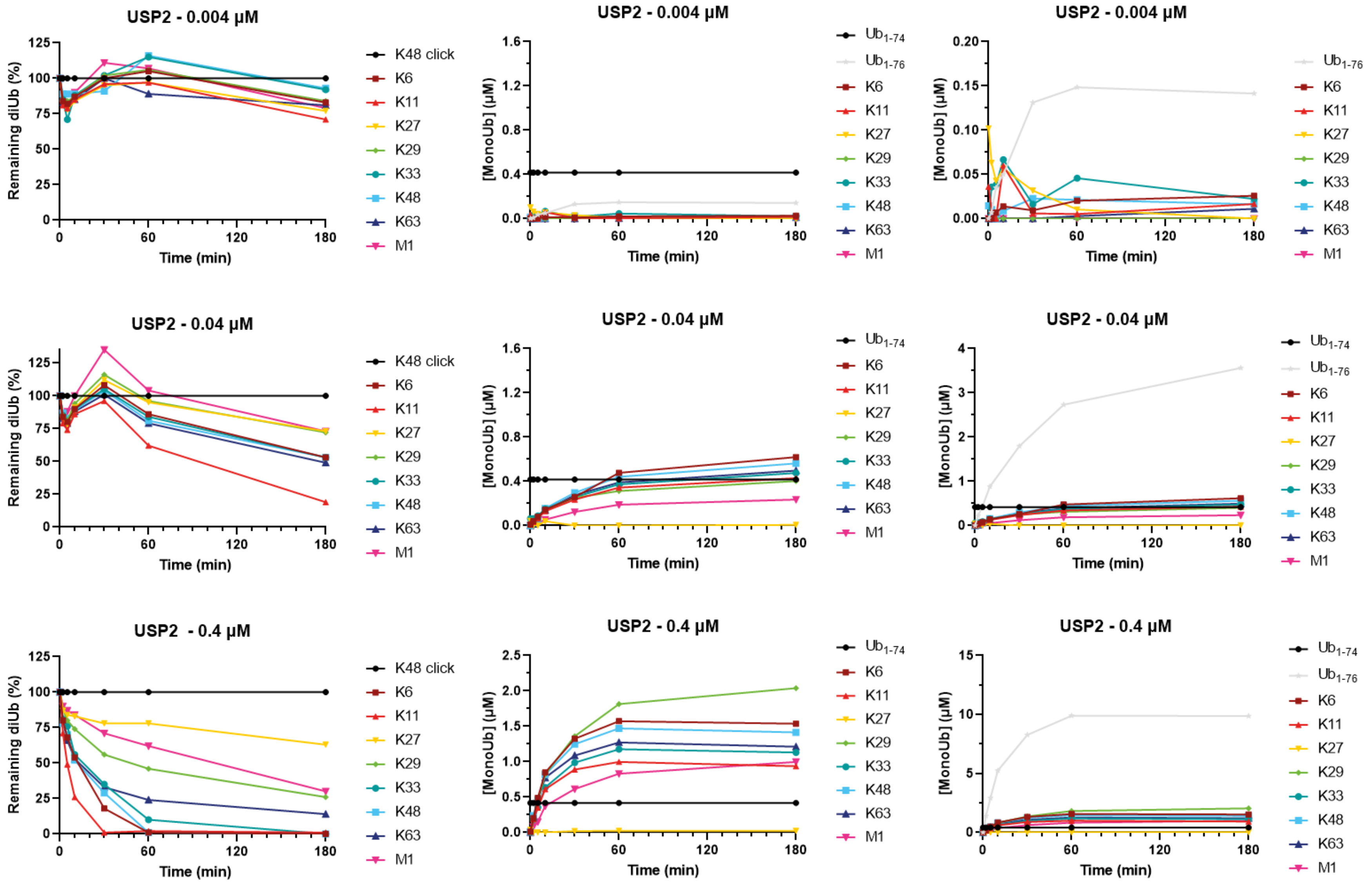

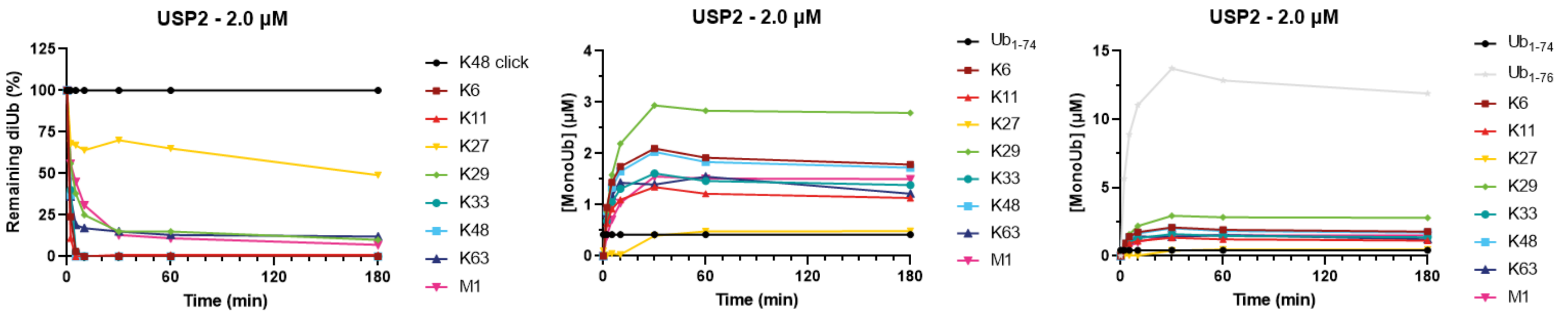
USP2 - 2.0 μM
Remaining diUb (%)
Time (min)
K48 click
K6
K11
K27
K29
K33
K48
K63
M1
USP2 - 2.0 μM
[MonoUb] (μM)
Time (min)
Ub1-74
K6
K11
K27
K29
K33
K48
K63
M1
USP2 - 2.0 μM
[MonoUb] (μM)
Time (min)
Ub1-74
Ub1-76
K6
K11
K27
K29
K33
K48
K63
M1

USP7

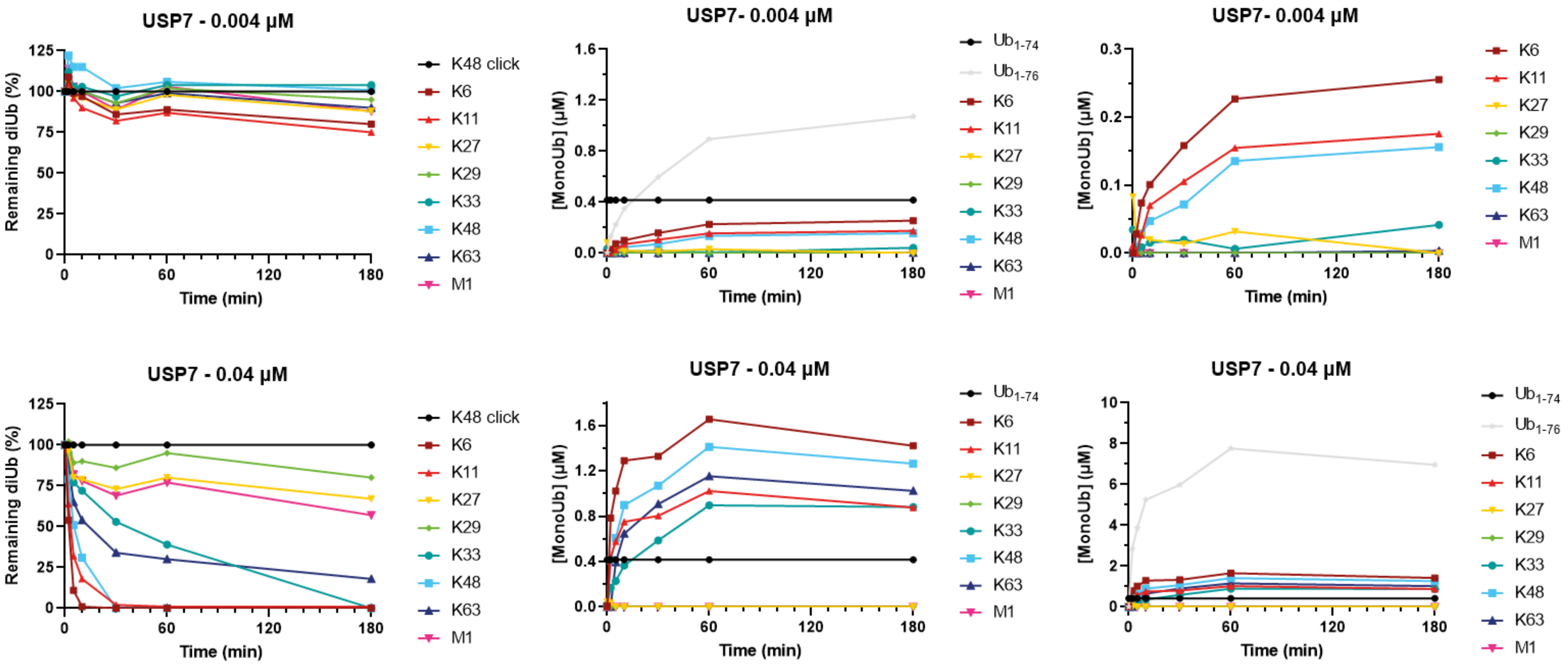
USP7 - 0.004 μM
Remaining diUb (%)
Time (min)
K48 click
K6
K11
K27
K29
K33
K48
K63
M1
USP7- 0.004 μM
[MonoUb] (μM)
Time (min)
Ub1-74
Ub1-76
K6
K11
K27
K29
K33
K48
K63
M1
USP7- 0.004 μM
[MonoUb] (μM)
Time (min)
K6
K11
K27
K29
K33
K48
K63
M1
USP7 - 0.04 μM
Remaining diUb (%)
Time (min)
K48 click
K6
K11
K27
K29
K33
K48
K63
M1
USP7 - 0.04 μM
[MonoUb] (μM)
Time (min)
Ub1-74
K6
K11
K27
K29
K33
K48
K63
M1
USP7 - 0.04 μM
[MonoUb] (μM)
Time (min)
Ub1-74
Ub1-76
K6
K11
K27
K29
K33
K48
K63
M1

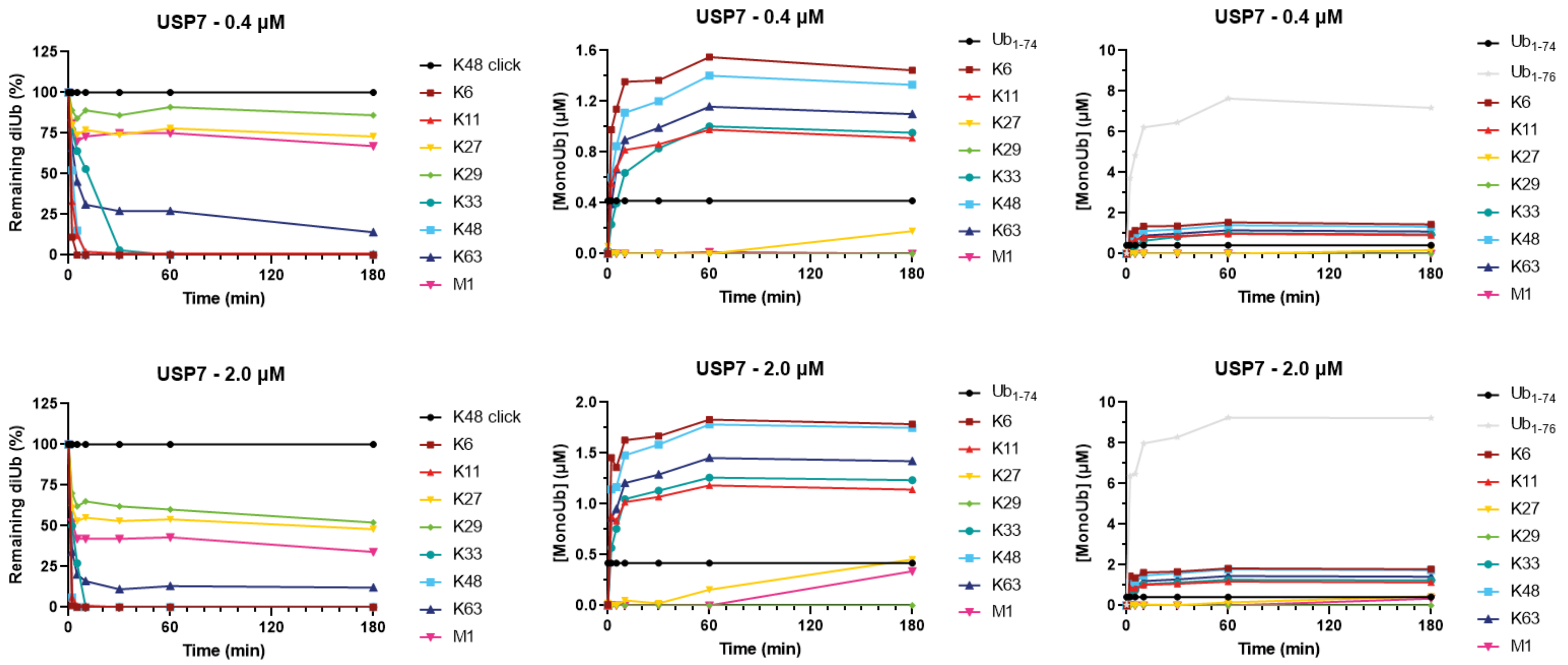
USP7 - 0.4 µM
Remaining diUb (%)
Time (min)
K48 click
K6
K11
K27
K29
K33
K48
K63
M1
USP7 - 0.4 µM
[MonoUb] (µM)
Time (min)
Ub1-74
K6
K11
K27
K29
K33
K48
K63
M1
USP7 - 0.4 µM
[MonoUb] (µM)
Time (min)
Ub1-74
Ub1-76
K6
K11
K27
K29
K33
K48
K63
M1
USP7 - 2.0 µM
Remaining diUb (%)
Time (min)
K48 click
K6
K11
K27
K29
K33
K48
K63
M1
USP7 - 2.0 µM
[MonoUb] (µM)
Time (min)
Ub1-74
K6
K11
K27
K29
K33
K48
K63
M1
USP7 - 2.0 µM
[MonoUb] (µM)
Time (min)
Ub1-74
Ub1-76
K6
K11
K27
K29
K33
K48
K63
M1

USP8

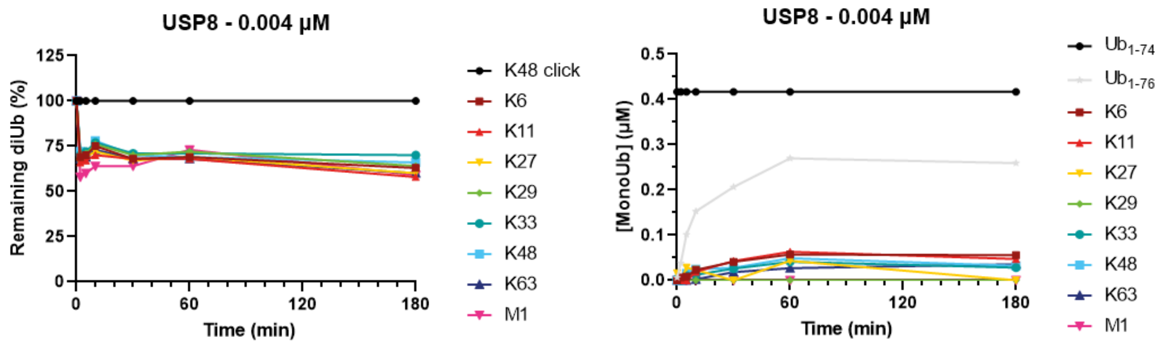
USP8 - 0.004 µM
Remaining diUb (%)
Time (min)
K48 click
K6
K11
K27
K29
K33
K48
K63
M1
USP8 - 0.004 µM
[MonoUb] (µM)
Time (min)
Ub1-74
Ub1-76
K6
K11
K27
K29
K33
K48
K63
M1

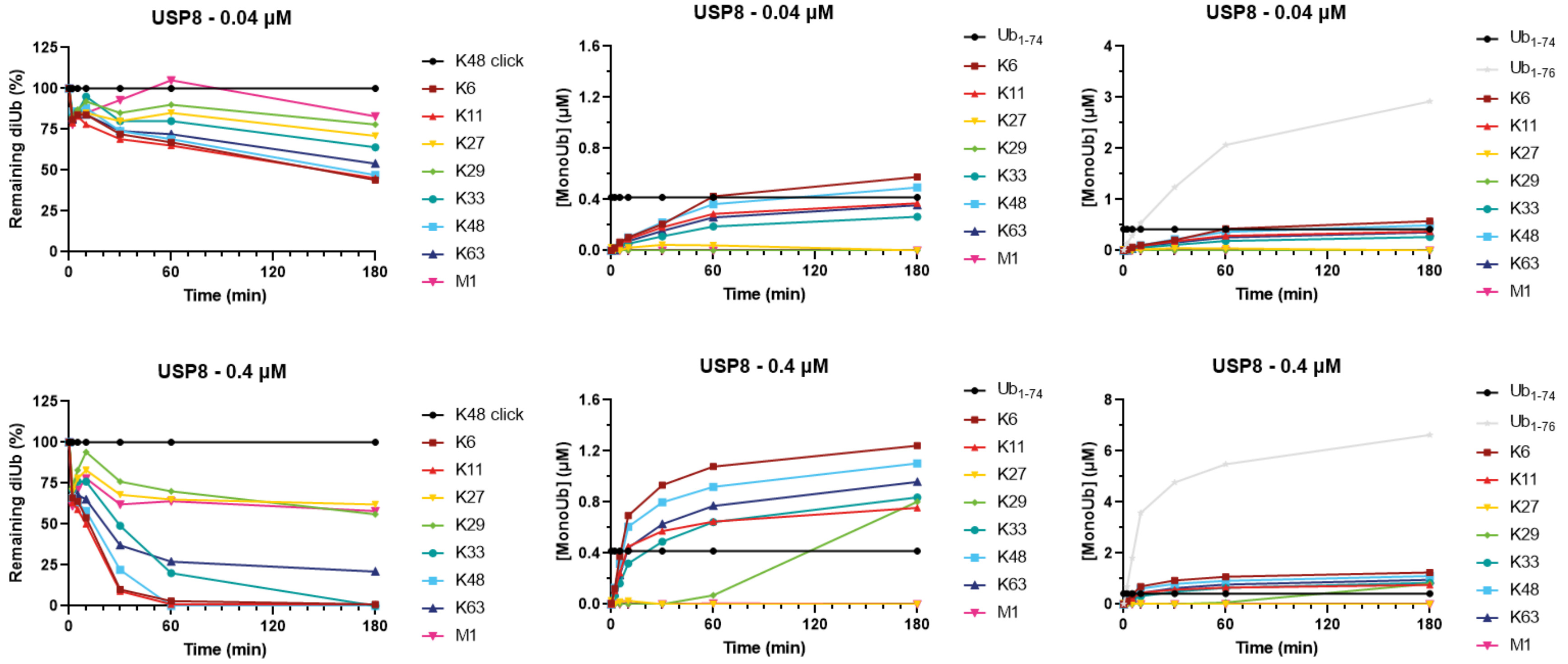
USP8 - 0.04 µM
Remaining diUb (%)
Time (min)
K48 click
K6
K11
K27
K29
K33
K48
K63
M1
USP8 - 0.04 µM
[MonoUb] (µM)
Time (min)
Ub1-74
K6
K11
K27
K29
K33
K48
K63
M1
USP8 - 0.04 µM
[MonoUb] (µM)
Time (min)
Ub1-74
Ub1-76
K6
K11
K27
K29
K33
K48
K63
M1
USP8 - 0.4 µM
Remaining diUb (%)
Time (min)
K48 click
K6
K11
K27
K29
K33
K48
K63
M1
USP8 - 0.4 µM
[MonoUb] (µM)
Time (min)
Ub1-74
K6
K11
K27
K29
K33
K48
K63
M1
USP8 - 0.4 µM
[MonoUb] (µM)
Time (min)
Ub1-74
Ub1-76
K6
K11
K27
K29
K33
K48
K63
M1

USP9x

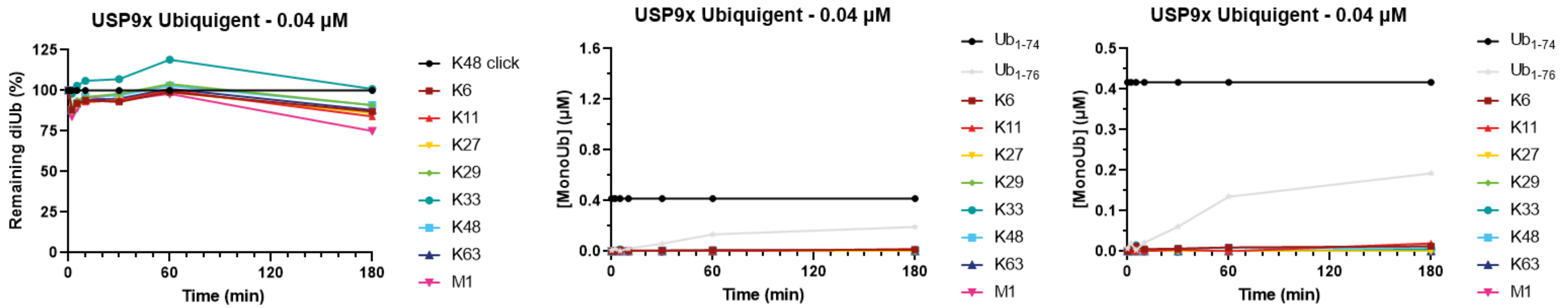
USP9x Ubiquigent - 0.04 µM
Remaining diUb (%)
Time (min)
K48 click
K6
K11
K27
K29
K33
K48
K63
M1
USP9x Ubiquigent - 0.04 µM
[MonoUb] (µM)
Time (min)
Ub1-74
Ub1-76
K6
K11
K27
K29
K33
K48
K63
M1
USP9x Ubiquigent - 0.04 µM
[MonoUb] (µM)
Time (min)
Ub1-74
Ub1-76
K6
K11
K27
K29
K33
K48
K63
M1

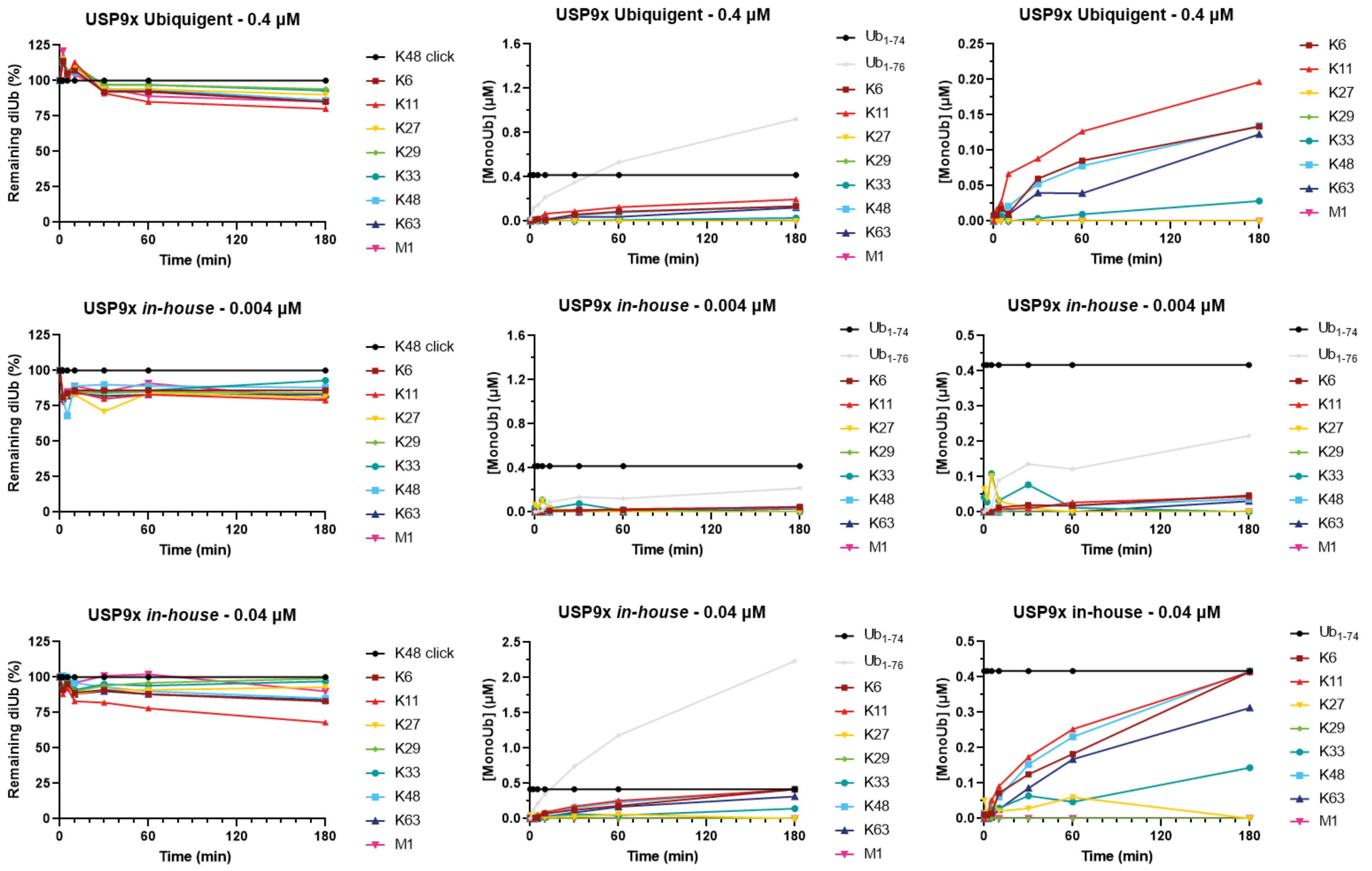

USP9x Ubiquigent - 0.4 µM
Remaining diUb (%)
Time (min)
K48 click
K6
K11
K27
K29
K33
K48
K63
M1
USP9x Ubiquigent - 0.4 µM
[MonoUb] (µM)
Time (min)
Ub_{1-74}
Ub_{1-76}
K6
K11
K27
K29
K33
K48
K63
M1
USP9x Ubiquigent - 0.4 µM
[MonoUb] (µM)
Time (min)
K6
K11
K27
K29
K33
K48
K63
M1
USP9x *in-house* - 0.004 µM
Remaining diUb (%)
Time (min)
K48 click
K6
K11
K27
K29
K33
K48
K63
M1
USP9x *in-house* - 0.004 µM
[MonoUb] (µM)
Time (min)
Ub_{1-74}
Ub_{1-76}
K6
K11
K27
K29
K33
K48
K63
M1
USP9x *in-house* - 0.004 µM
[MonoUb] (µM)
Time (min)
Ub_{1-74}
Ub_{1-76}
K6
K11
K27
K29
K33
K48
K63
M1
USP9x *in-house* - 0.04 µM
Remaining diUb (%)
Time (min)
K48 click
K6
K11
K27
K29
K33
K48
K63
M1
USP9x *in-house* - 0.04 µM
[MonoUb] (µM)
Time (min)
Ub_{1-74}
Ub_{1-76}
K6
K11
K27
K29
K33
K48
K63
M1
USP9x in-house - 0.04 µM
[MonoUb] (µM)
Time (min)
Ub_{1-74}
K6
K11
K27
K29
K33
K48
K63
M1

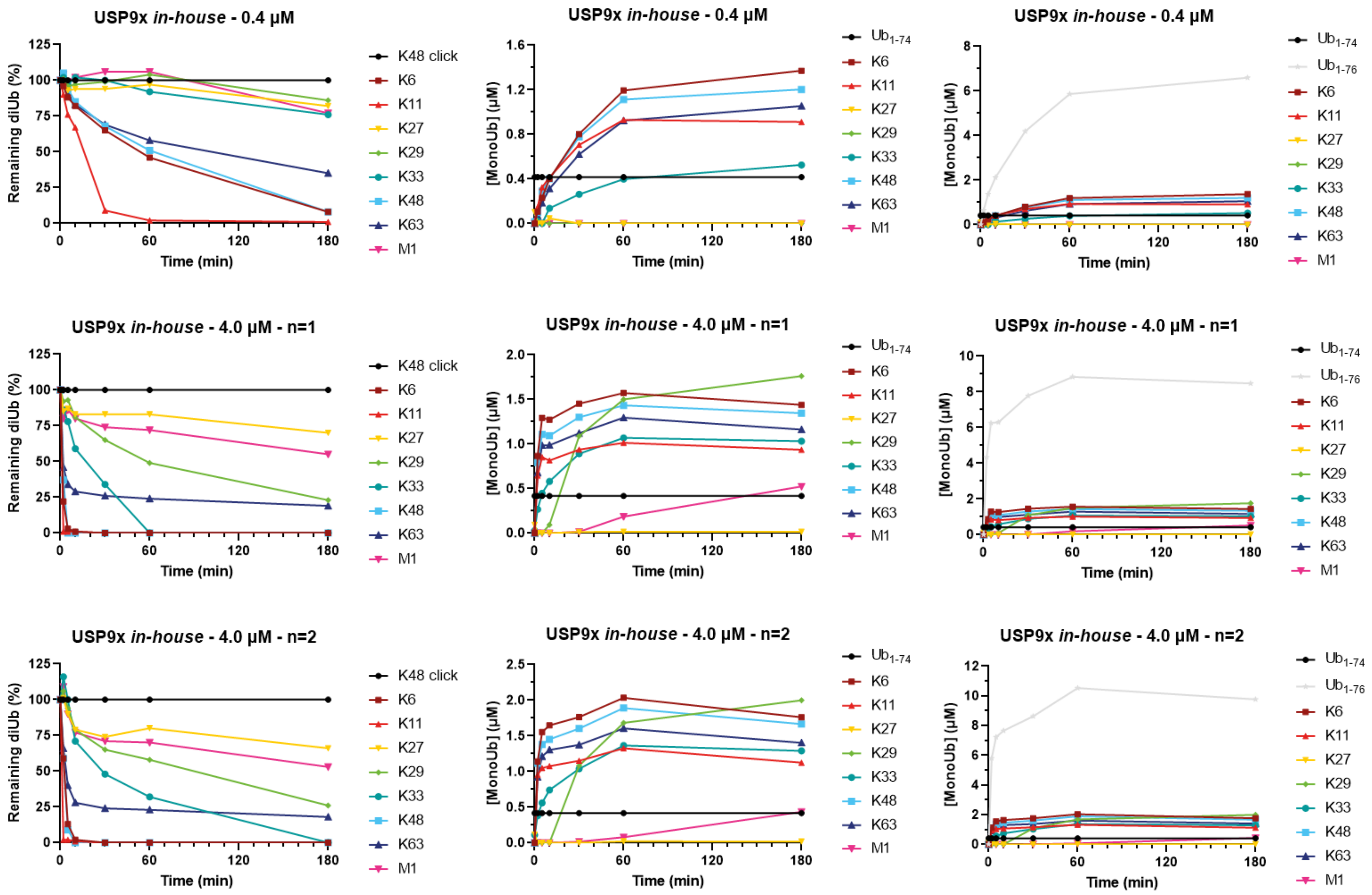
USP9x in-house - 0.4 µM
Remaining diUb (%)
Time (min)
K48 click
K6
K11
K27
K29
K33
K48
K63
M1
USP9x in-house - 0.4 µM
[MonoUb] (µM)
Time (min)
Ub_{1-74}
K6
K11
K27
K29
K33
K48
K63
M1
USP9x in-house - 0.4 µM
[MonoUb] (µM)
Time (min)
Ub_{1-74}
Ub_{1-76}
K6
K11
K27
K29
K33
K48
K63
M1
USP9x in-house - 4.0 µM - n=1
Remaining diUb (%)
Time (min)
USP9x in-house - 4.0 µM - n=1
[MonoUb] (µM)
Time (min)
USP9x in-house - 4.0 µM - n=1
[MonoUb] (µM)
Time (min)
USP9x in-house - 4.0 µM - n=2
Remaining diUb (%)
Time (min)
USP9x in-house - 4.0 µM - n=2
[MonoUb] (µM)
Time (min)
USP9x in-house - 4.0 µM - n=2
[MonoUb] (µM)
Time (min)

USP10

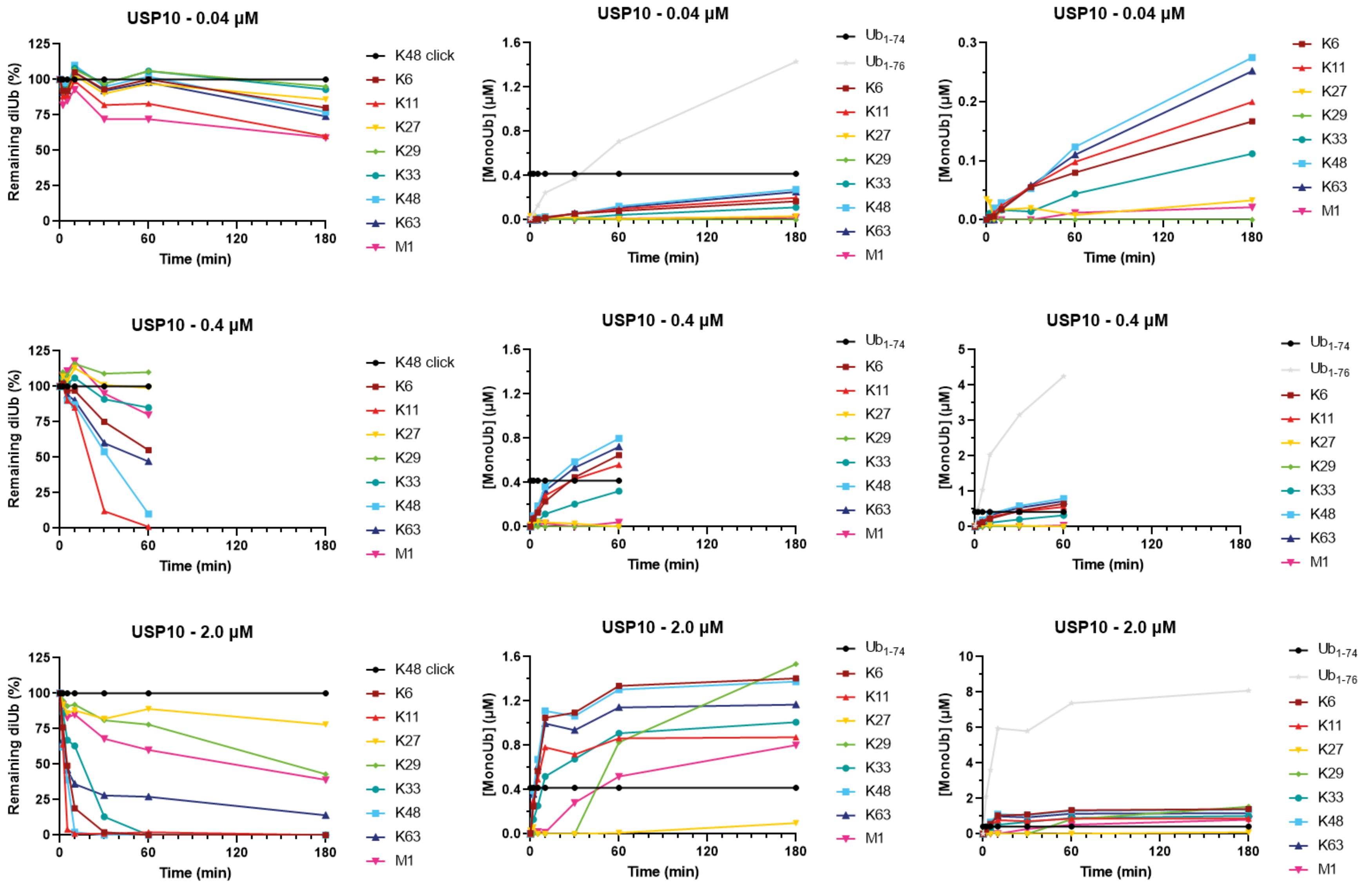
USP10 - 0.04 µM
Remaining diUb (%)
Time (min)
K48 click
K6
K11
K27
K29
K33
K48
K63
M1
[MonoUb] (µM)
Ub1-74
Ub1-76
USP10 - 0.4 µM
USP10 - 2.0 µM

USP16

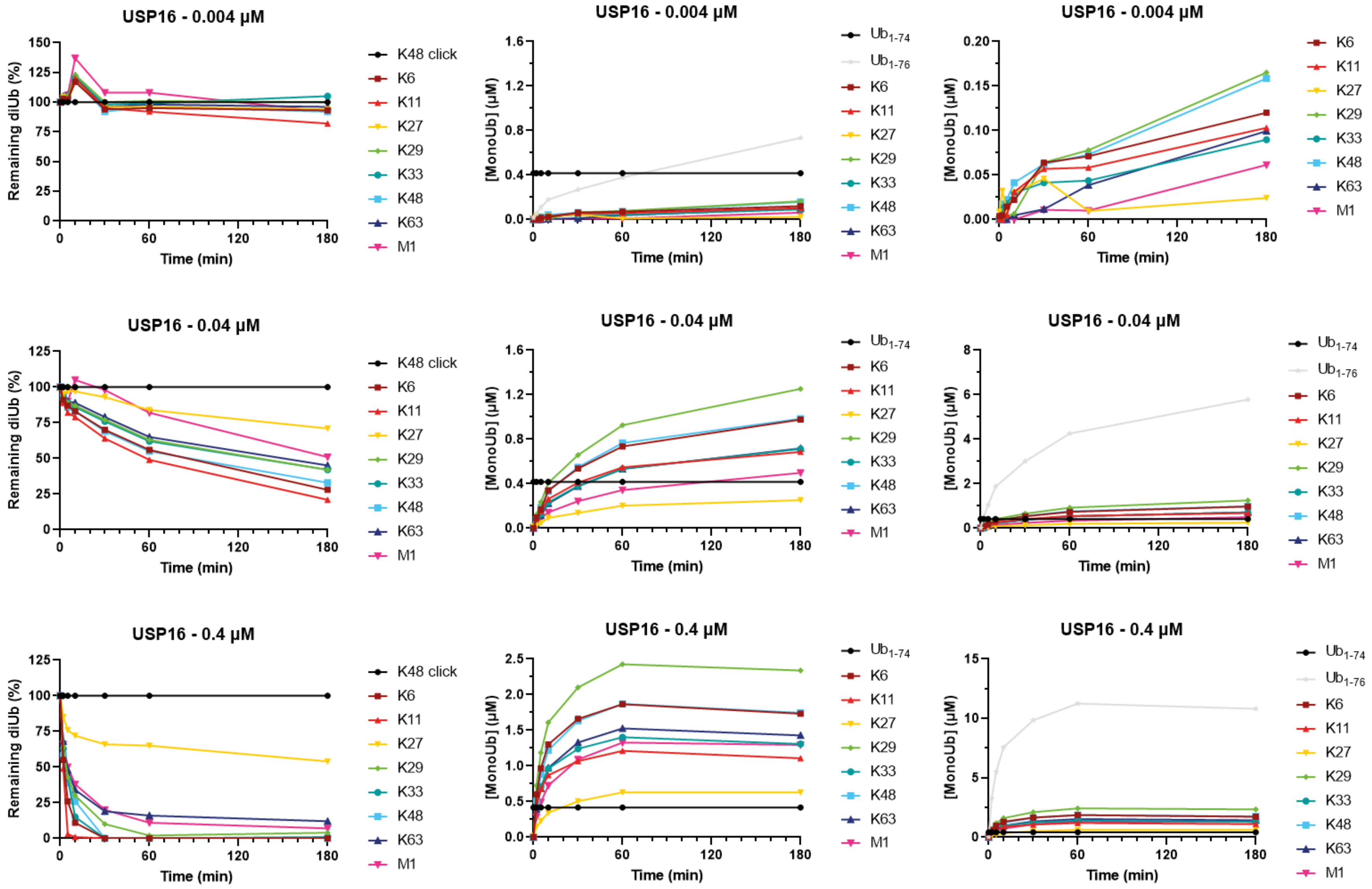

USP16 - 0.004 µM
USP16 - 0.04 µM
USP16 - 0.4 µM
Remaining diUb (%)
[MonoUb] (µM)
Time (min)
K48 click
Ub1-74
Ub1-76
K6
K11
K27
K29
K33
K48
K63
M1

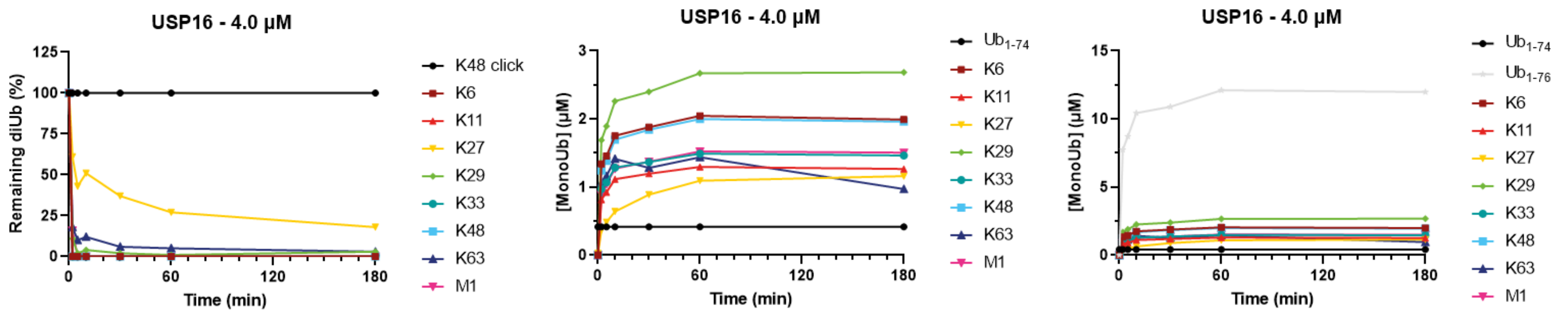

c, Different DUB families DUBs

AMSH-LP

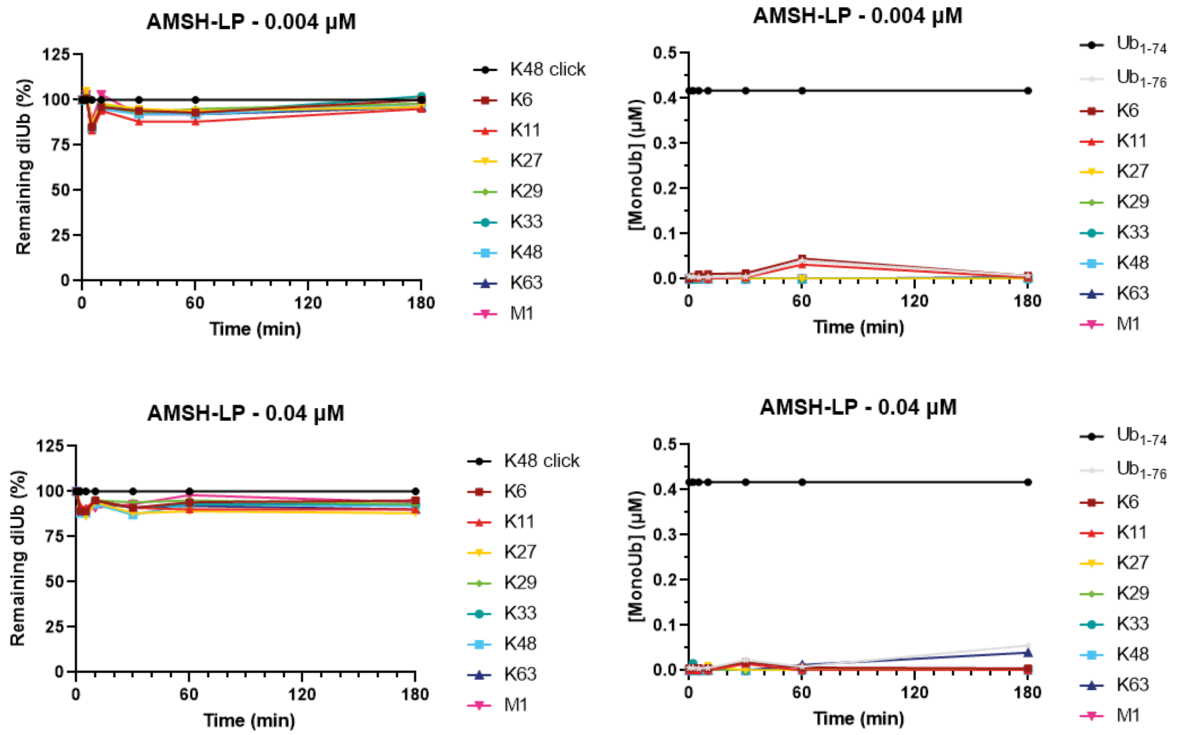

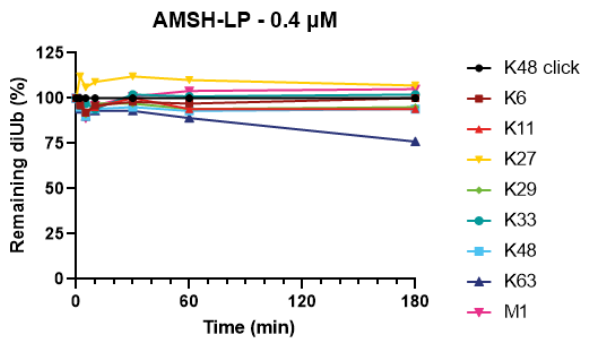
AMSH-LP - 0.4 µM
Remaining diUb (%)
Time (min)
K48 click
K6
K11
K27
K29
K33
K48
K63
M1

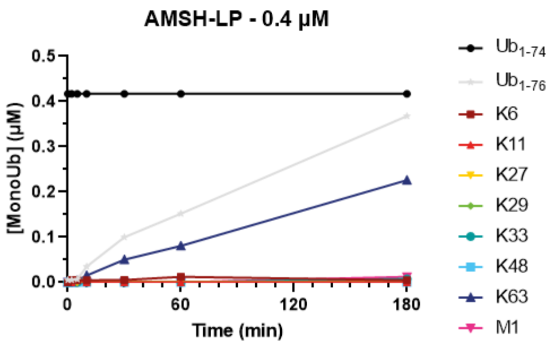
AMSH-LP - 0.4 µM
[MonoUb] (µM)
Time (min)
Ub_{1-74}
Ub_{1-76}
K6
K11
K27
K29
K33
K48
K63
M1

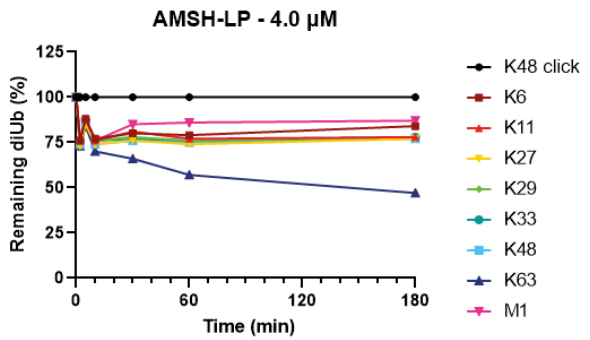
AMSH-LP - 4.0 µM
Remaining diUb (%)
Time (min)
K48 click
K6
K11
K27
K29
K33
K48
K63
M1

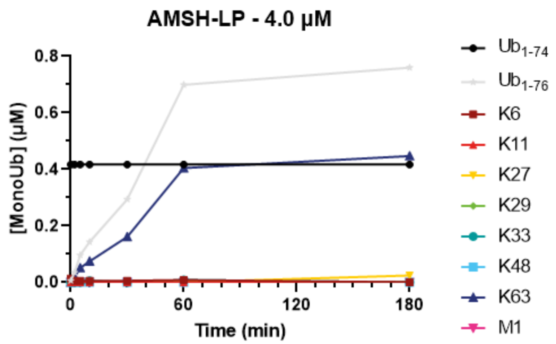
AMSH-LP - 4.0 µM
[MonoUb] (µM)
Time (min)
Ub_{1-74}
Ub_{1-76}
K6
K11
K27
K29
K33
K48
K63
M1

AMSH

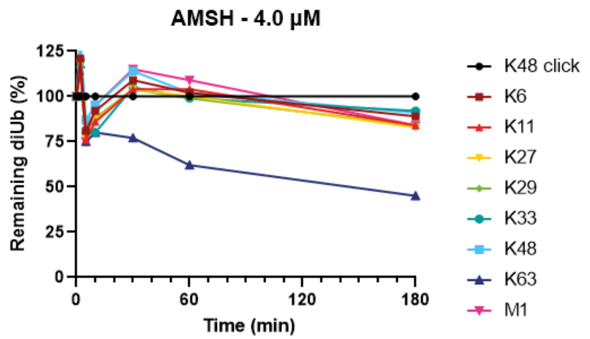
AMSH - 4.0 µM
Remaining diUb (%)
Time (min)
K48 click
K6
K11
K27
K29
K33
K48
K63
M1

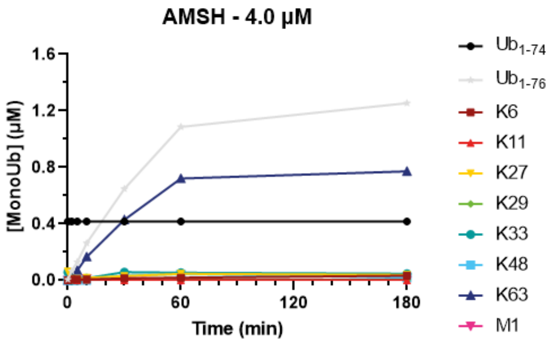
AMSH - 4.0 µM
[MonoUb] (µM)
Time (min)
Ub_{1-74}
Ub_{1-76}
K6
K11
K27
K29
K33
K48
K63
M1

RPN11/RPN8

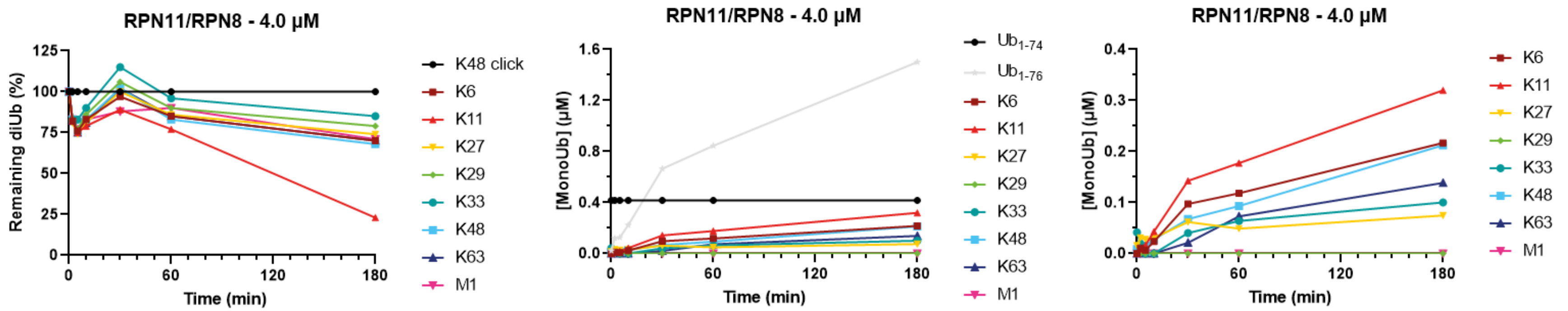

USP11

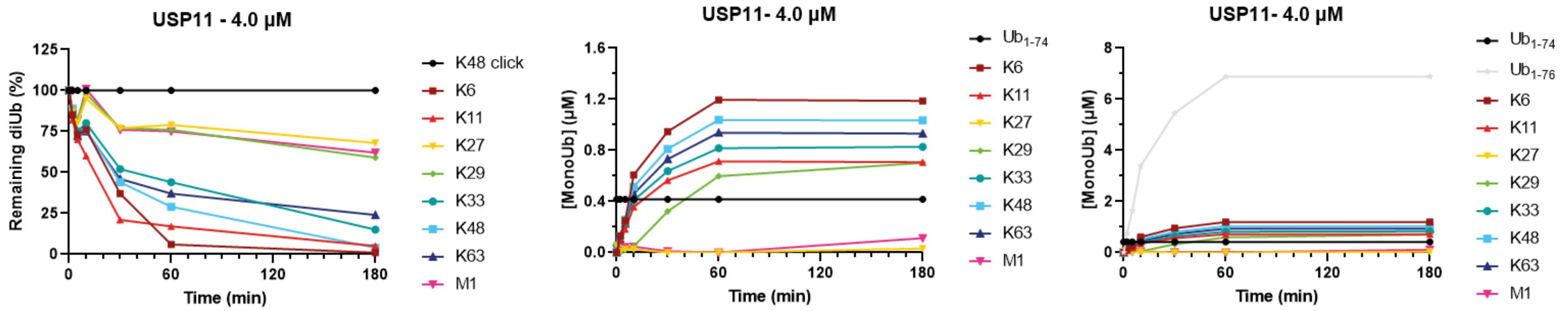

USP32

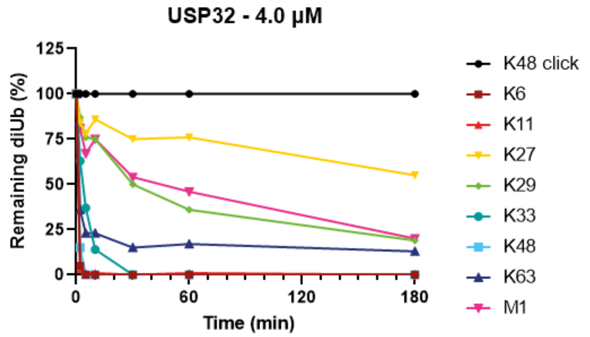
USP32 - 4.0 µM
Remaining diUb (%)
125
100
75
50
25
0
0
60
120
180
Time (min)
K48 click
K6
K11
K27
K29
K33
K48
K63
M1

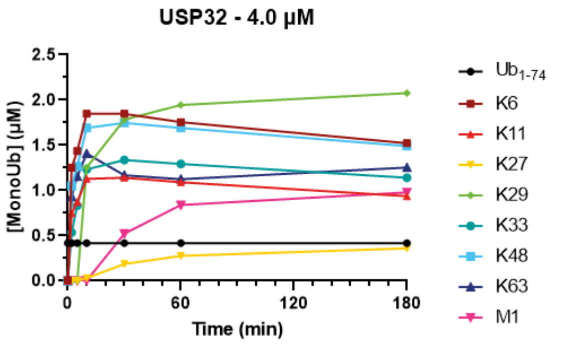
USP32 - 4.0 µM
[MonoUb] (µM)
2.5
2.0
1.5
1.0
0.5
0.0
0
60
120
180
Time (min)
Ub1-74
K6
K11
K27
K29
K33
K48
K63
M1

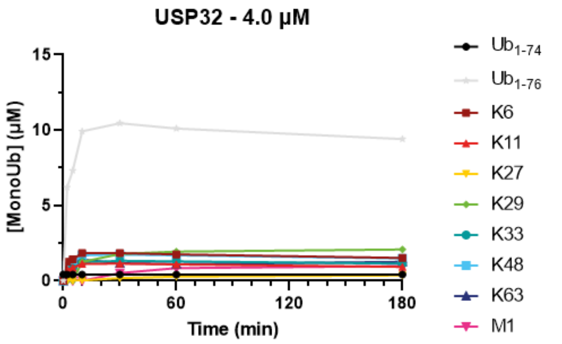
USP32 - 4.0 µM
[MonoUb] (µM)
15
10
5
0
0
60
120
180
Time (min)
Ub1-74
Ub1-76
K6
K11
K27
K29
K33
K48
K63
M1

USP34

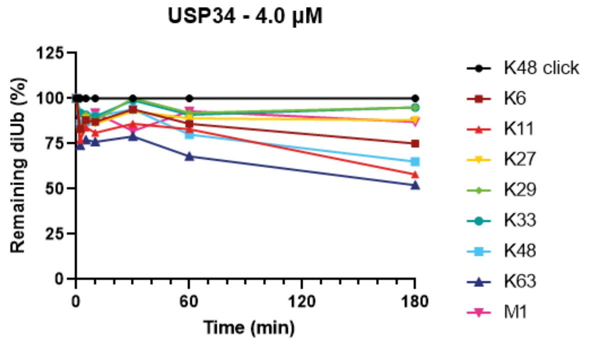
USP34 - 4.0 µM
Remaining diUb (%)
125
100
75
50
25
0
0
60
120
180
Time (min)
K48 click
K6
K11
K27
K29
K33
K48
K63
M1

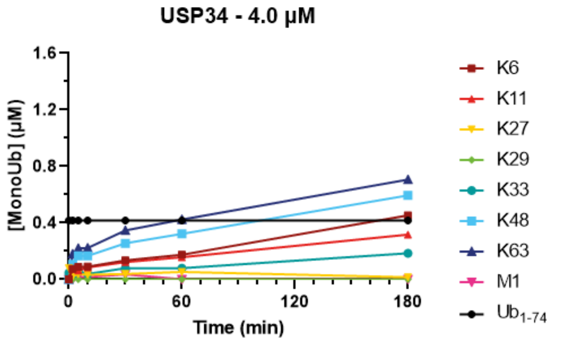
USP34 - 4.0 µM
[MonoUb] (µM)
1.6
1.2
0.8
0.4
0.0
0
60
120
180
Time (min)
K6
K11
K27
K29
K33
K48
K63
M1
Ub1-74

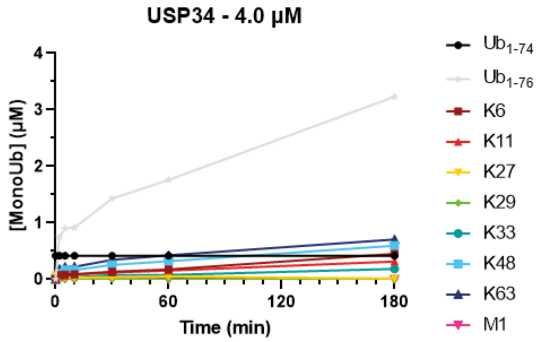
USP34 - 4.0 µM
[MonoUb] (µM)
4
3
2
1
0
0
60
120
180
Time (min)
Ub1-74
Ub1-76
K6
K11
K27
K29
K33
K48
K63
M1

ATAXIN-L3

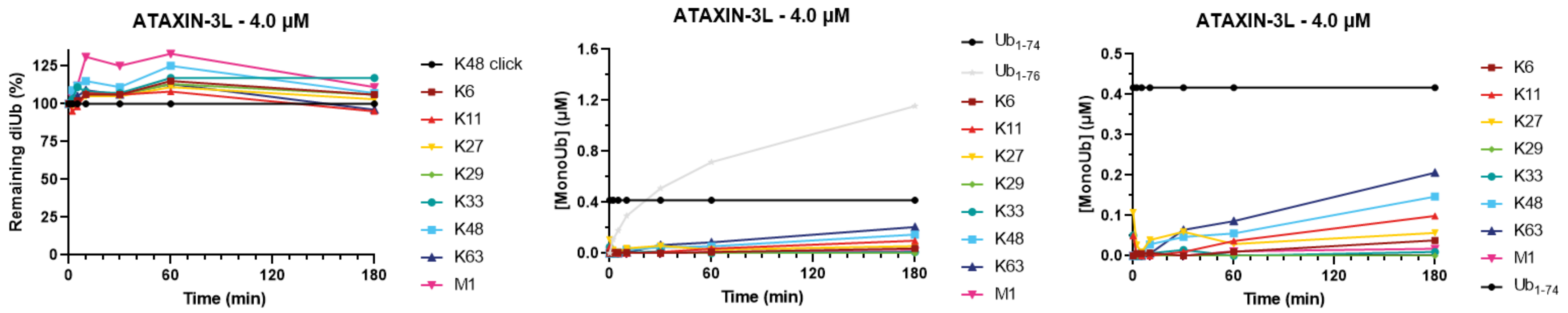

JOSD1

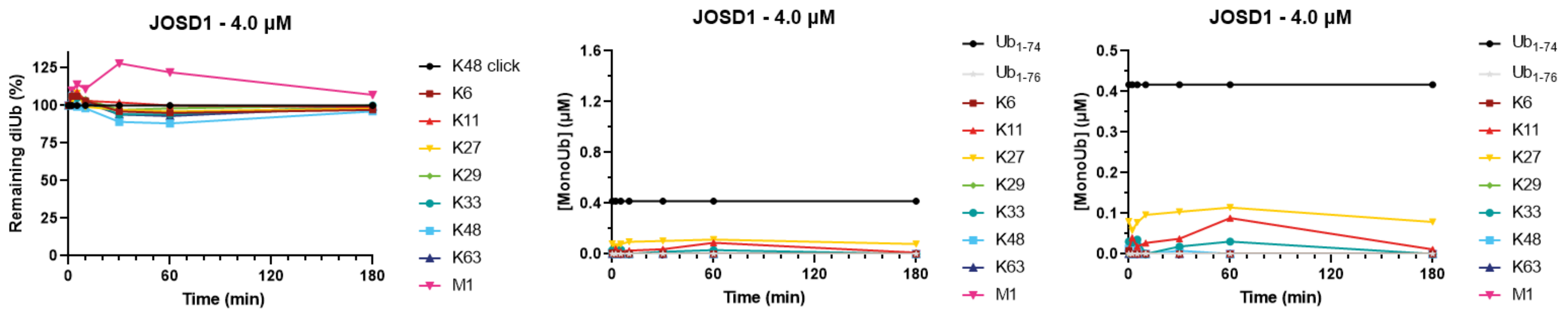

JOSD2

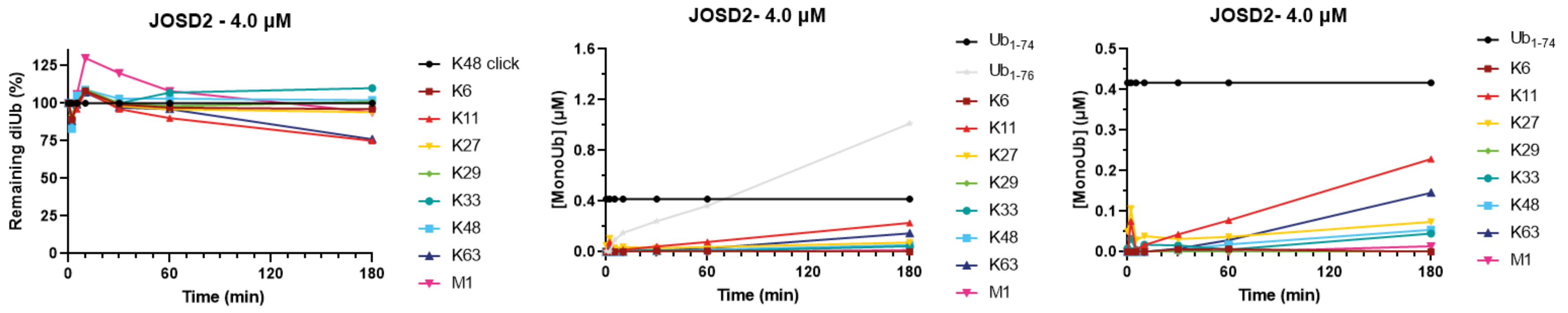
JOSD2 - 4.0 µM
Remaining diUb (%)
Time (min)
K48 click
K6
K11
K27
K29
K33
K48
K63
M1
JOSD2- 4.0 µM
[MonoUb] (µM)
Time (min)
Ub1-74
Ub1-76
K6
K11
K27
K29
K33
K48
K63
M1
JOSD2- 4.0 µM
[MonoUb] (µM)
Time (min)
Ub1-74
K6
K11
K27
K29
K33
K48
K63
M1
