# Supplementary material for: Neutron-encoded diubiquitins to profile linkage selectivity of deubiquitinating enzymes
Source: Nat Commun. 2023 Mar 25;14:1661. doi: 10.1038/s41467-023-37363-6 (PMC10039891; doi:10.1038/s41467-023-37363-6)
Supplement: Supplementary file 6 — Reporting Summary [file 41467_2023_37363_MOESM6_ESM.pdf]

nature portfolio

Corresponding author(s): Paul P. Geurink

Last updated by author(s): Feb 23, 2023

Reporting Summary

Nature Portfolio wishes to improve the reproducibility of the work that we publish. This form provides structure for consistency and transparency in reporting. For further information on Nature Portfolio policies, see our Editorial Policies and the Editorial Policy Checklist.

Statistics

For all statistical analyses, confirm that the following items are present in the figure legend, table legend, main text, or Methods section.

n/a	Confirmed	
☐	☒	The exact sample size (*n*) for each experimental group/condition, given as a discrete number and unit of measurement
☐	☒	A statement on whether measurements were taken from distinct samples or whether the same sample was measured repeatedly
☒	☐	The statistical test(s) used AND whether they are one- or two-sided<br>*Only common tests should be described solely by name; describe more complex techniques in the Methods section.*
☒	☐	A description of all covariates tested
☒	☐	A description of any assumptions or corrections, such as tests of normality and adjustment for multiple comparisons
☒	☐	A full description of the statistical parameters including central tendency (e.g. means) or other basic estimates (e.g. regression coefficient) AND variation (e.g. standard deviation) or associated estimates of uncertainty (e.g. confidence intervals)
☒	☐	For null hypothesis testing, the test statistic (e.g. *F*, *t*, *r*) with confidence intervals, effect sizes, degrees of freedom and *P* value noted<br>*Give P values as exact values whenever suitable.*
☒	☐	For Bayesian analysis, information on the choice of priors and Markov chain Monte Carlo settings
☒	☐	For hierarchical and complex designs, identification of the appropriate level for tests and full reporting of outcomes
☒	☐	Estimates of effect sizes (e.g. Cohen's *d*, Pearson's *r*), indicating how they were calculated

Our web collection on statistics for biologists contains articles on many of the points above.

Software and code

Policy information about availability of computer code

Data collection	MS data was collected using MassLynx software V4.2 SCN966 (Waters Inc.)
Data analysis	Proteowizard 3.0.20274 was used to convert raw data files to the mzXML file format. mzXML files were further processed using LaCyTools version 22.04.29. LaCyTools is freely available for download at https://github.com/Tarskin/LaCyTools. The LaCyTools output file (Summary.txt) was further processed in Microsoft Excel 365. Data was plotted using GraphPadPrism 9.3.0. Chemical structures were drawn in Chemdraw 22.0.0.

For manuscripts utilizing custom algorithms or software that are central to the research but not yet described in published literature, software must be made available to editors and reviewers. We strongly encourage code deposition in a community repository (e.g. GitHub). See the Nature Portfolio guidelines for submitting code & software for further information.

Data

Policy information about availability of data

All manuscripts must include a data availability statement. This statement should provide the following information, where applicable:

- Accession codes, unique identifiers, or web links for publicly available datasets
- A description of any restrictions on data availability
- For clinical datasets or third party data, please ensure that the statement adheres to our policy

The experiment data that support the findings of this study are available from the corresponding author upon reasonable request. All MS datasets generated and/or

analysed during the current study have been deposited with the ProteomeXchange Consortium via the MassIVE partner repository with data set identifier MSV000090455 [https://massive.ucsd.edu/ProteoSAFe/dataset.jsp?task=896ad6c6a30b444daf4bba82aeca2470]. Additional source data are provided with this paper.

Human research participants

Policy information about studies involving human research participants and Sex and Gender in Research.

Reporting on sex and gender	No human research participants
Population characteristics	No human research participants
Recruitment	No human research participants
Ethics oversight	No human research participants

Note that full information on the approval of the study protocol must also be provided in the manuscript.

Field-specific reporting

Please select the one below that is the best fit for your research. If you are not sure, read the appropriate sections before making your selection.

☒ Life sciences ☐ Behavioural & social sciences ☐ Ecological, evolutionary & environmental sciences

For a reference copy of the document with all sections, see nature.com/documents/nr-reporting-summary-flat.pdf

Life sciences study design

All studies must disclose on these points even when the disclosure is negative.

Sample size	For the assay window/linearity determination experiments (Supplementary Figure 7), n=3 individual experiments to enable the calculation of the SEM. Each diUb cleavage assay was performed once for each DUB at 4 different concentrations.
Data exclusions	For the following DUBs the lowest enzyme concentration (0.004 microM) was excluded because no enzyme activity was observed: OTUD2, OTUD3 (Figure 5a and Supplementary Data 2).
Replication	The experiment for USP9x in-house - 4 uM was performed twice and was successful.
Randomization	The study involves the biochemical analysis of enzyme activity/selectivity by mass spectrometry. Randomization was not applicable to this study, as no studies on advanced organisms were performed. Randomising the order of sample analysis on the mass spectrometer would not impact the results of the measurements as blanco samples were measured before or after each sample to verify absence of background signal coming from earlier measurements.
Blinding	The study involves the biochemical analysis of enzyme activity/selectivity by mass spectrometry. No blinding techniques were used in this study, experiments were carried out with labeled compounds. Blinding is not relevant as the mass spectrometer is not influenced or affected by earlier measurements.

Reporting for specific materials, systems and methods

We require information from authors about some types of materials, experimental systems and methods used in many studies. Here, indicate whether each material, system or method listed is relevant to your study. If you are not sure if a list item applies to your research, read the appropriate section before selecting a response.

Materials & experimental systems

n/a	Involved in the study	
☒	☐	Antibodies
☒	☐	Eukaryotic cell lines
☒	☐	Palaeontology and archaeology
☒	☐	Animals and other organisms
☒	☐	Clinical data
☒	☐	Dual use research of concern

Methods

n/a	Involved in the study	
☒	☐	ChIP-seq
☒	☐	Flow cytometry
☒	☐	MRI-based neuroimaging
