# Supplementary figures and images for: Neutron-encoded diubiquitins to profile linkage selectivity of deubiquitinating enzymes
Source: Nat Commun. 2023 Mar 25;14:1661. doi: 10.1038/s41467-023-37363-6 (PMC10039891; doi:10.1038/s41467-023-37363-6)

## Slide 1
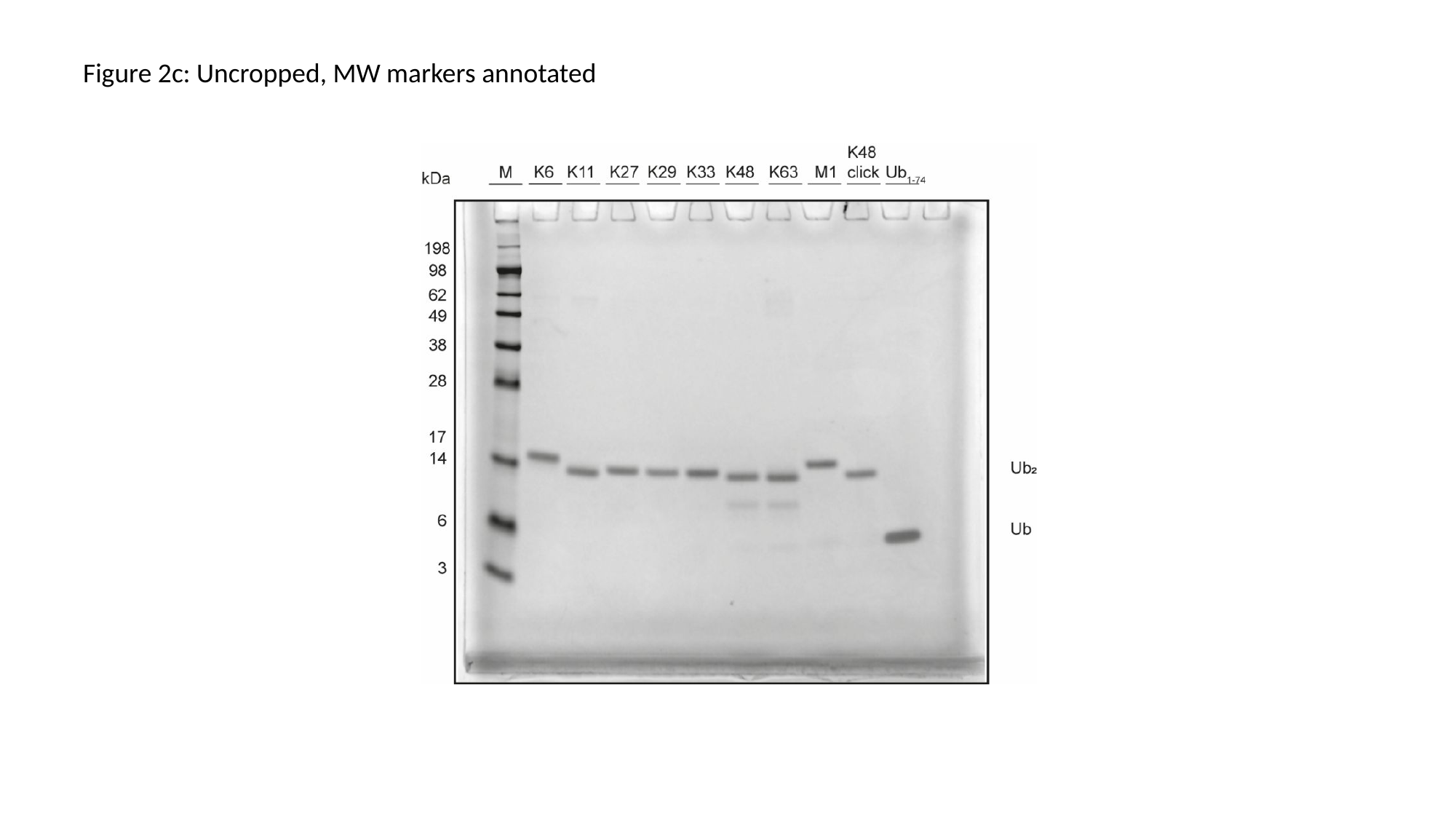

Figure 2c: Uncropped, MW markers annotated

Supplement: Supplementary file 7 — Source Data [file 41467_2023_37363_MOESM7_ESM.zip › Uncropped gel images/Figure 2c.pptx]

## Slide 1
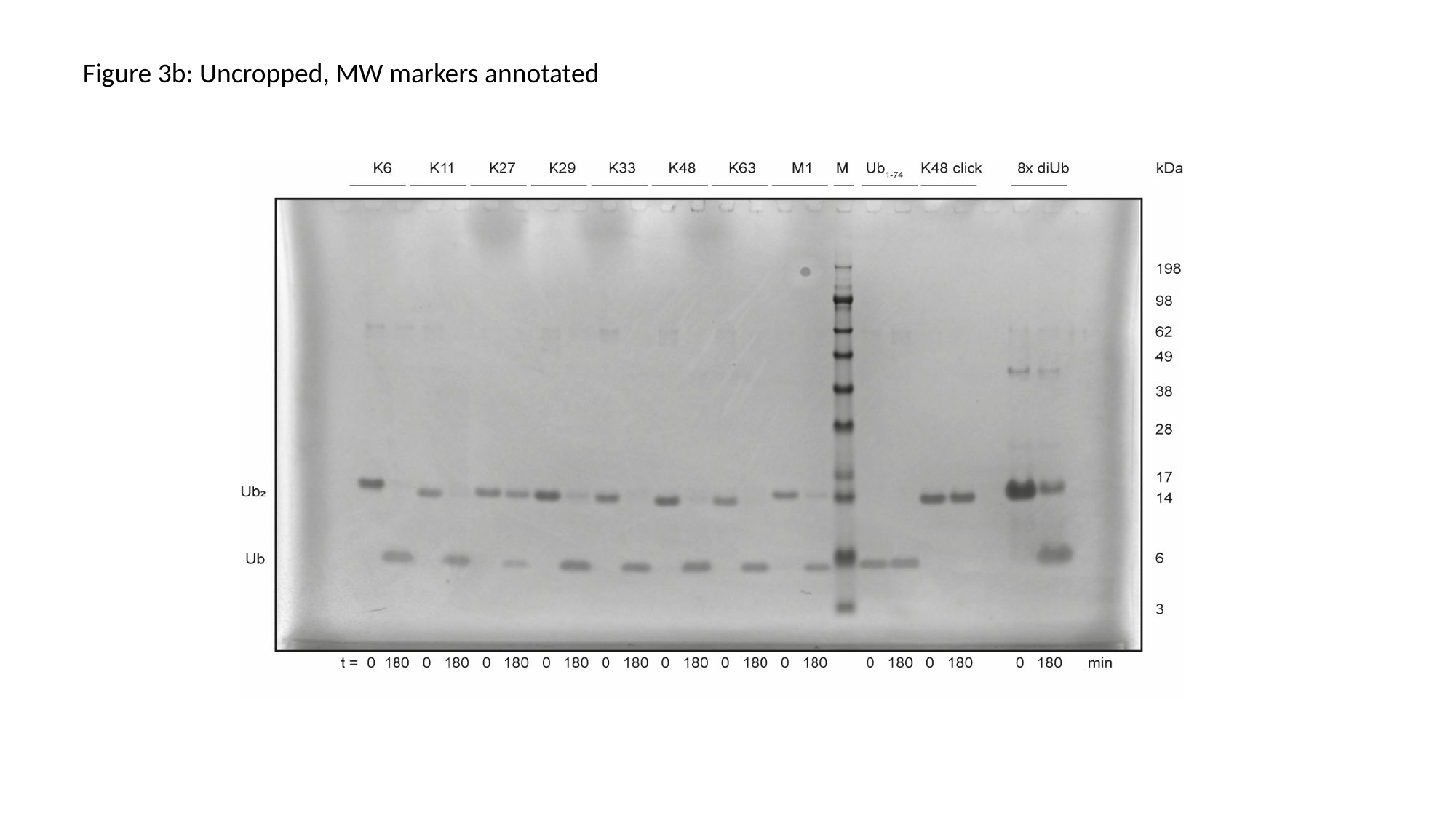

Figure 3b: Uncropped, MW markers annotated

Supplement: Supplementary file 7 — Source Data [file 41467_2023_37363_MOESM7_ESM.zip › Uncropped gel images/Figure 3b.pptx]

## Slide 1
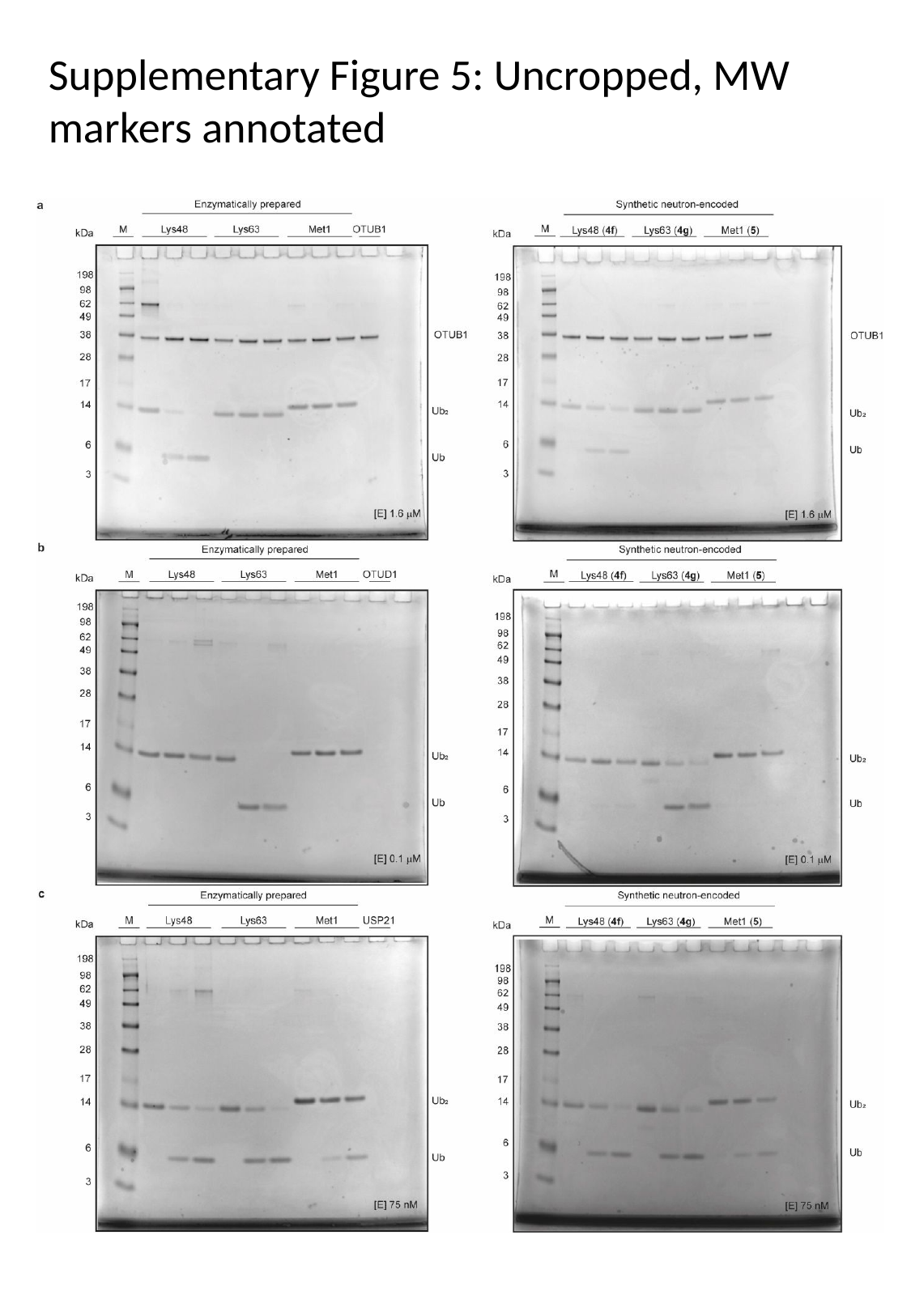

Supplementary Figure 5: Uncropped, MW markers annotated

Supplement: Supplementary file 7 — Source Data [file 41467_2023_37363_MOESM7_ESM.zip › Uncropped gel images/Supplementary Figure 5.pptx]
